# Development of Transiently Strainable Benzocycloheptenes for Catalyst-Free, Visible-Light-Mediated [3 + 2]-Cycloadditions

**DOI:** 10.1021/acs.bioconjchem.4c00595

**Published:** 2025-02-04

**Authors:** Shivangi Kharbanda, Osaid Alkhamayseh, Georgia Eastham, Jimmie D. Weaver

**Affiliations:** Department of Chemistry, Oklahoma State University, Stillwater, Oklahoma 74078, United States

## Abstract

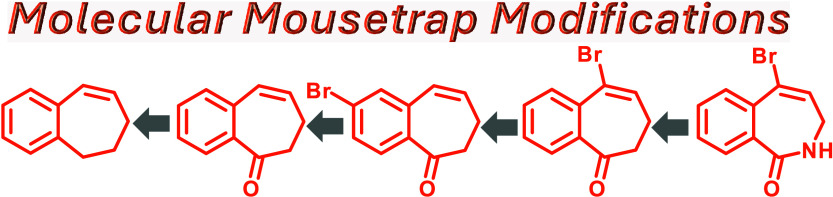

Dynamic photogeneration of ephemeral and reactive species is enabling for chemical reactions, providing spatial and temporal control. A previous study from our group established the ability of 6,7-dihydro-5H-benzo[7]annulene, benzocycloheptene (**BC7**), to convert photochemical energy into ring strain, enabling the rapid cycloaddition of alkyl azides with the reversibly formed and transient *trans*-isomer, affording versatile nonaromatic triazolines. Despite the conceptual advances of the previous study, some challenges remained: the fragility of the triazoline products, the low regioselectivity for the cycloaddition, a need for an iridium-based photosensitizer and organic-based solvents, and a lack of convenient linchpin functional group handles. Herein, we communicate the development of a second generation of **BC7** molecules that overcome the issues of the first generation. A method to convert fragile triazoline products to stable triazoles was developed. The alkene component was polarized with a carbonyl group, dramatically improving the regioselectivity while simultaneously red-shifting the absorbance of the cycloalkene into the visible region, which was expected to facilitate direct excitation and eliminate the need for photocatalysts. However, experiments indicated that the cycloaddition involved passage through a triplet manifold, complicating the direct excitation strategy. This was successfully overcome by attaching a bromine atom directly to the alkene moiety, which accelerated singlet-to-triplet intersystem crossing by the heavy atom effect. Further exploration identified sites of substitution that can increase the water solubility and provide a handle for the loading of chemical tools and probes.

Substituted 1,2,3-triazoles have gained renown as molecular unions that can be orthogonally “clicked” together under a wide array of conditions. The technology dates back to Huisgen’s pioneering work in the 1960s on cycloaddition reactions^1^ and culminated in the 2022 Nobel Prize in Chemistry.^2^ Initially, limitations in regioselectivity and the need for high temperatures hindered their widespread use. Development of the copper(I)-catalyzed azide–alkyne cycloaddition by Sharpless^3^ and application by Meldal^4^ groups offered mild reaction conditions, rapid reaction rates, and exceptional regioselectivity compared to thermal reactions, which triggered a rapid expansion of its applications beyond synthetic chemistry to fields such as drug discovery, chemical biology, and materials science.^5^ However, some applications involving living cells are intolerant of Cu(I) catalysts.^6^ To address this issue, Bertozzi^7^ introduced strain-promoted azide–alkyne cycloaddition (SPAAC) in 2004, which relied on the elevated ground-state energies of the contorted cyclooctynes to reduce the reaction barriers, eliminating the need for copper catalysts.

A notable advancement in SPAAC is the development of phototriggered alkyne–azide cycloaddition, which offers some level of user-dictated spatial and temporal control of the reaction. Several iterations with unique advantages have been reported.^8^ For instance, a kinetically stable cyclopropenone derivative serves as a masked precursor of the cyclooctyne, which, upon photoirradiation with 350 nm light, undergoes irreversible decarbonylation, forming the reactive cyclooctyne.^9^ While such methods provide precise control of the release of the dipolarophile, they can face the same challenges of homocoupling and off-site reactivity observed in the SPAAC.^10^ An additional challenge arises from the poor solubility introduced by the extended conjugation which is needed to red-shift the absorption.^9a^

We have been investigating visible-light-induced strainable ring systems, characterized by dynamic thermal reversibility.^11^ Such systems act as molecular transducers, efficiently converting the potential energy from electronically excited sensitizers into strain energy in elevated ground-state cycloalkene stereoisomers, which undergo strain-promoted cycloadditions, among other transformations. This approach offers superior spatial and temporal control by virtue of the brief lifetime of the strained isomers, which rapidly revert to their dormant forms in the absence of azide. We reported our first efforts on the subject in 2018 (Scheme 1),^12^ which proved the benzocycloheptene derivative (**BC7-B**) is capable of rapid (15 min) insulin conjugation with blue light irradiation at ambient conditions and open to air.

**Scheme 1:**
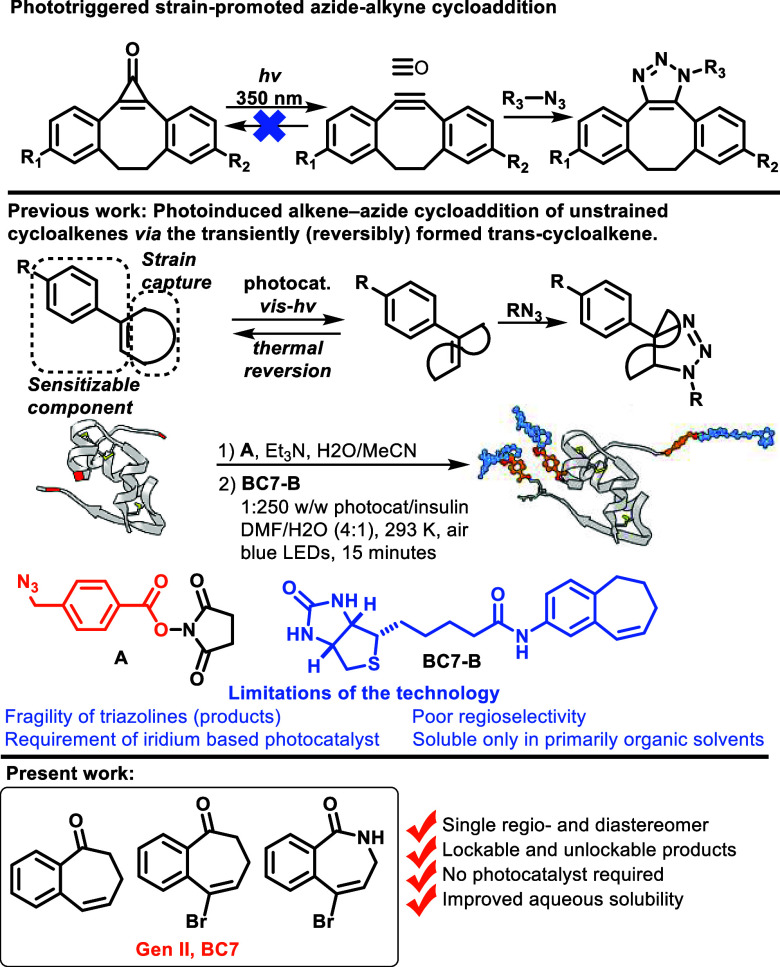
Photoinduced Cycloaddition of Unstrained Molecules via a Transiently Strained Coupling Partner

While this study introduced a new potentially useful motif for conjugation applications, it was not without some limitations, which included the fragility of the nonaromatic triazoline products, the modest regioselectivity, the requirement of an external sensitizer, and the relatively low solubility in aqueous systems of both the substrate and the photosensitizer. Unlike robust aromatic triazoles, triazoline products can be intentionally decomposed under moderately acidic conditions (acids with p*K*_a_ < 3) to give α-tetralone and the corresponding amine.^12^ While the catch and release strategy can be useful, for example, in a protein pull-down experiment, there are scenarios in which this linkage needs to be robust, and we also wanted to explore methods that produce robust triazoles directly from the triazolines.

To obtain the triazoles, we began by oxidizing a 4:1 mixture of triazolines **1d** and **1d’** (Scheme 2) as our model substrates, which were synthesized based on the previously reported conditions by our group.^12^ The best results were observed with tert-butyl hydroperoxide and catalytic KI.^13^ This oxidation system proved to be critical in the selective oxidation of the triazoline substituted benzylic site over the other benzylic sites. While we observed that the major triazoline cleanly converted to the triazole (**2d**), we observed that the minor regioisomer formed triazole **2d’** in lower yields, along with other unidentified products (Scheme 2).

**Scheme 2:**
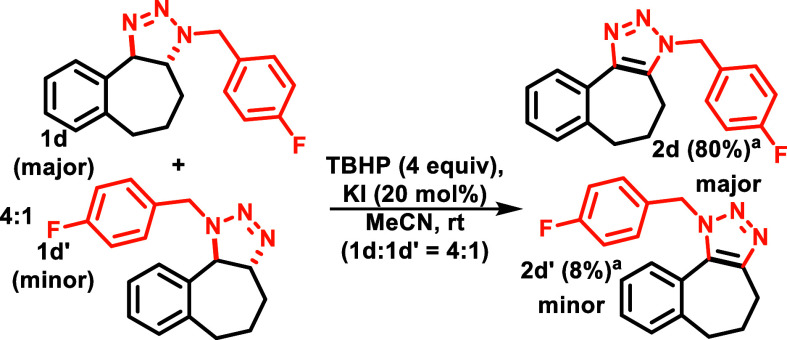
Oxidation on Triazoline Regioisomers Yields are based on ^19^F NMR with the addition of fluorobenzene as the internal standard.

During our efforts, we found that we could intentionally and selectively decompose the minor triazoline (**1d’**) by exposure to silica gel, which would leave the major triazoline (**1d**) unchanged and serve to facilitate isolation of the major triazole. Therefore, the minor triazoline was deliberately decomposed, and the purified major triazoline was subsequently subjected to oxidation conditions, resulting in the formation of a singular triazole product with excellent yields (Scheme 3).

**Scheme 3:**
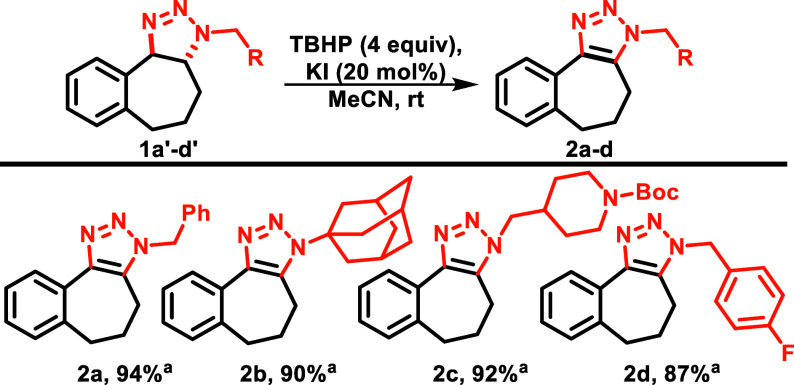
Triazoline Oxidation Isolated yields.

While the TBHP oxidation conditions facilitated the isolation of the major regioisomer, they came at the cost of the minor isomer. Huisgen had suggested that the relative distribution of regioisomers could be influenced by electronic factors,^1b,14^ suggesting that modulating the polarity of our alkene could potentially enhance regioselectivity. In addition, in 1975, Hart^15^ demonstrated a series of photoirradiation experiments involving benzocycloheptenone derivatives and furan. The observed stereochemistry of the [4 + 2] adduct was consistent with the reaction of a *trans*-alkene. Hence, on the basis of these two literary hints, we explored the [3 + 2] cycloaddition of azides with **oxo-BC7** (**3**, Scheme 4).

**Scheme 4:**
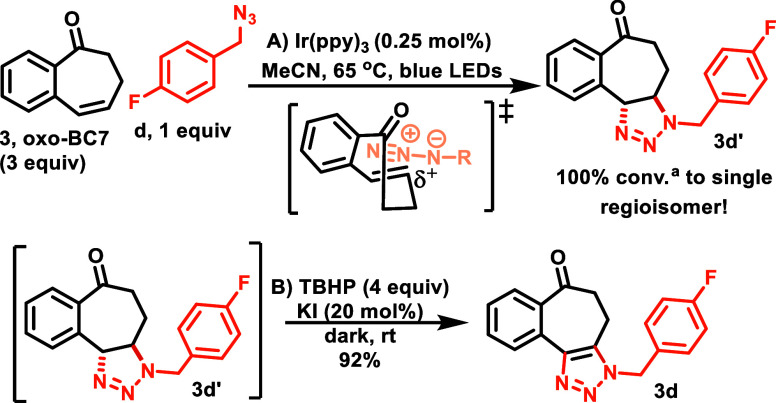
Sensitized [3 + 2]-Cycloaddition and Telescoped Oxidation of **oxo-BC7** Conv. based on ^1^H NMR.

When **oxo-BC7** (**3 equiv**)^16^ was subjected to photocycloaddition with para-fluorobenzyl azide (1 equiv), we were pleased to see the full conversion of azide **d** to a single regioisomer and diastereomer (**3d’**) (see the SI for details). Importantly, this result showed that **oxo-BC7** (1) could be sensitized using visible light, (2) was capable of undergoing [3 + 2]-cycloadditions with azides, and (3) did result in high regioselectivity, showing that the polarization of the alkene was an effective tool to enhance regioselectivity.

Attempts to purify the triazoline **3d’** via column chromatography proved unsuccessful due to its instability to acid. A telescoped one-pot approach circumvented the issue by directly oxidizing the reaction mixture. After the azide was consumed, catalytic KI and stoichiometric TBHP were added, which yielded the desired, silica-stable triazoles (Scheme 4).

With the proof of concept in hand, we moved to the systematic study of this cycloaddition reaction. Methyl 4-(azidomethyl)benzoate (**f**) was chosen as the model substrate along with **oxo-BC7** for this study. We screened different photocatalysts, solvents, photocatalyst loading and reaction concentrations, and the impact of air on the reaction (see the SI for details on the optimization study). *fac*-Ir(Fppy)_3_ at 0.25 mol % loading in MeCN solvent (0.2 M) provided the best yield, with minimum reaction times. More readily available *fac*-Ir(ppy)_3_ also provided similar yields with extended reaction times and was used throughout this study. We also observed that open, unsparged reactions still worked, albeit with slightly diminished rates, likely stemming from competitive and unproductive quenching by O_2_. We next explored the scope of this reaction (Scheme 5). All azides (**a**–**j**) yielded single regioisomers, which were subsequently oxidized to the triazoles with excellent telescoped yields. Addition of more TBHP and an extended reaction time cleanly led to the formation of the fully aromatic tricyclic motifs (**3a”**, **3e”**, **3g”**, and **3j”**), which appeared to fluoresce under UV light, which is noteworthy, as blue-emitting compounds remain of great interest.^17^ In nearly all cases, we achieved highly comparable yields using either Ir(Fppy)_3_ or Ir(ppy)_3_, simply by extending the reaction times for triazoline formation.

**Scheme 5:**
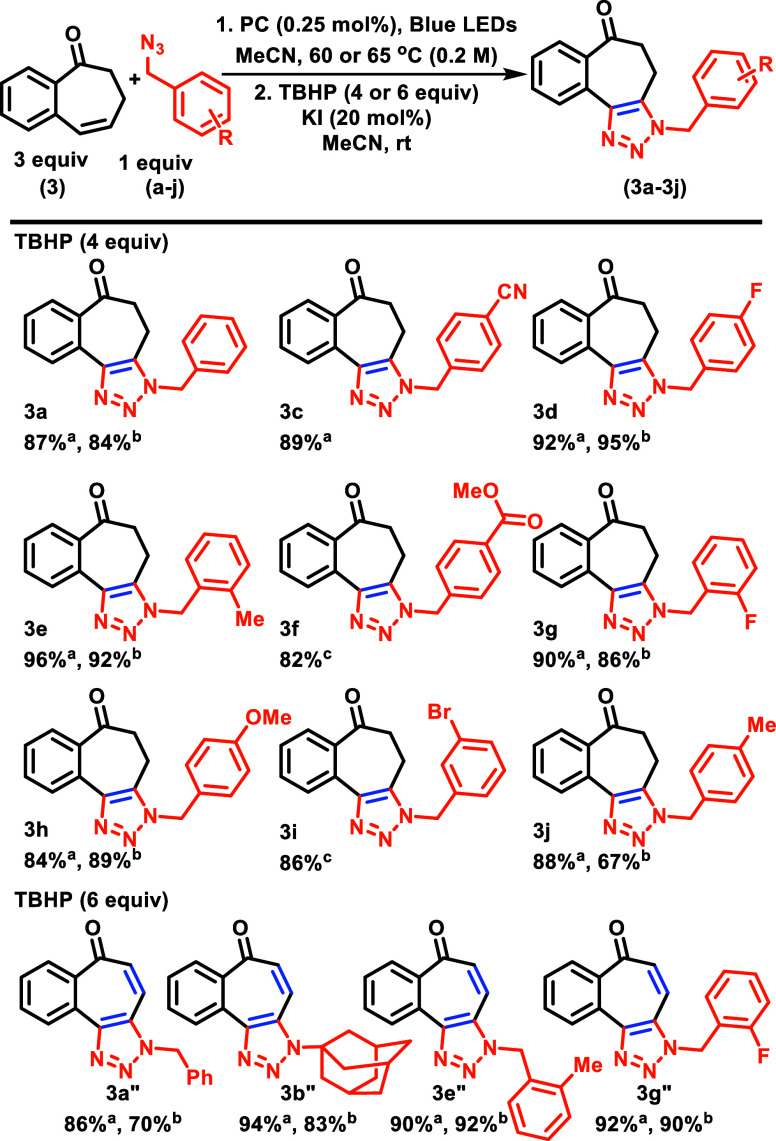
Sensitized [3 + 2]-Reaction and Telescoped Oxidation with **oxo-BC7** and Azides Isolated yield with Ir(ppy)_3_ at 65 °C. NMR yield with Ir(Fppy)_3_ at 60 °C. Isolated yield with Ir(Fppy)_3_ at 60 °C.

While our efforts had solved the issues of regioselectivity and product stability, the reaction still required a photosensitizer. Given the similarity to acetophenone, which is a known triplet sensitizer,^18^ we wondered whether **oxo-BC7** could be directly excited, undergo ISC to the triplet, followed by ISC to yield a highly reactive *trans*-cycloheptenone. If so, this would enable photocycloaddition simply by irradiation with light at sufficient energy. To gain a more comprehensive understanding of the system, we performed UV–vis absorption spectroscopy on **oxo-BC7** (Figure 1). This revealed a strong absorption at 358 nm in MeCN that tailed into the visible range. By moving from blue light to violet or UV, we hoped that we might directly excite **oxo-BC7**, which might proceed to undergo ISC to the excited triplet, and ultimately give the strained *trans***-oxo-BC7**. Our hopes were bolstered by a recent study by Tang^19^ in which the authors were able to synthesize triazoles by direct UV-irradiation of cycloheptenones and azides in air.

**Figure 1:**
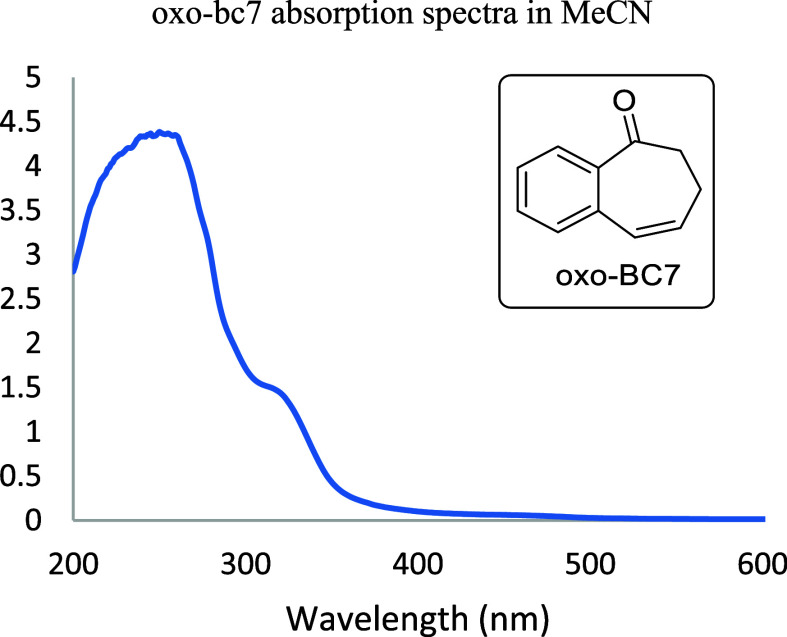
UV–vis absorption spectra of **oxo-BC7** in MeCN.

However, when we conducted the reaction without an Ir photocatalyst, but while irradiating with blue-, violet-, or UV (370 nm)-LED lights (Scheme 6), we failed to observe the desired triazoline product and the starting material remained unchanged, indicating perhaps that the formation of the *trans***-oxo-BC7** does not happen in the absence of an Ir photocatalyst,^20^ which is a well-established triplet sensitizer.^21^ Therefore, we suspected that **oxo-BC7** does not undergo intersystem crossing (ISC) or serve as a self-sensitizer for the photocycloaddition reaction and instead requires an external photosensitizer. A potential explanation could be due to the greater spin population of S_1_ being located primarily on the alkene rather than the carbonyl (where the ISC would be accelerated by the orthogonal frontier orbitals).

**Scheme 6:**
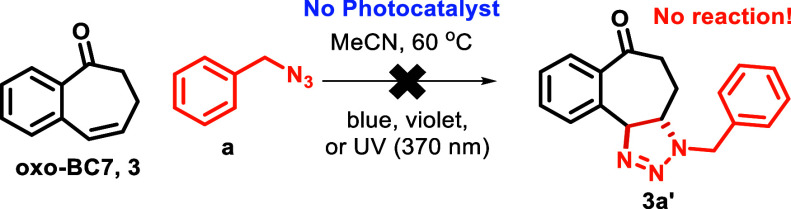
Failed Sensitizer-Free [3 + 2]-Cycloaddition Reaction of **oxo-BC7**

Based on its absorption spectrum (Figure 1), we believe that **oxo-BC7** was being excited to the singlet (*S*_1_), but that the *S*_1_ must undergo a nonradiative decay process(es) faster than ISC to the triplet (*T*_1_), relaxation, ISC to the ground state (*S*_0_), where it is expected to arrive at the transition state for rotation about the double bond, and from here, relax to the *trans***-oxo-BC7**, from which subsequent cycloaddition is expected to occur (Figure 2). We postulated, then, that accessing the *T*_1_ state was critical for productive cycloaddition, which leads to the strained, twisted alkene followed by cycloaddition.

**Figure 2:**
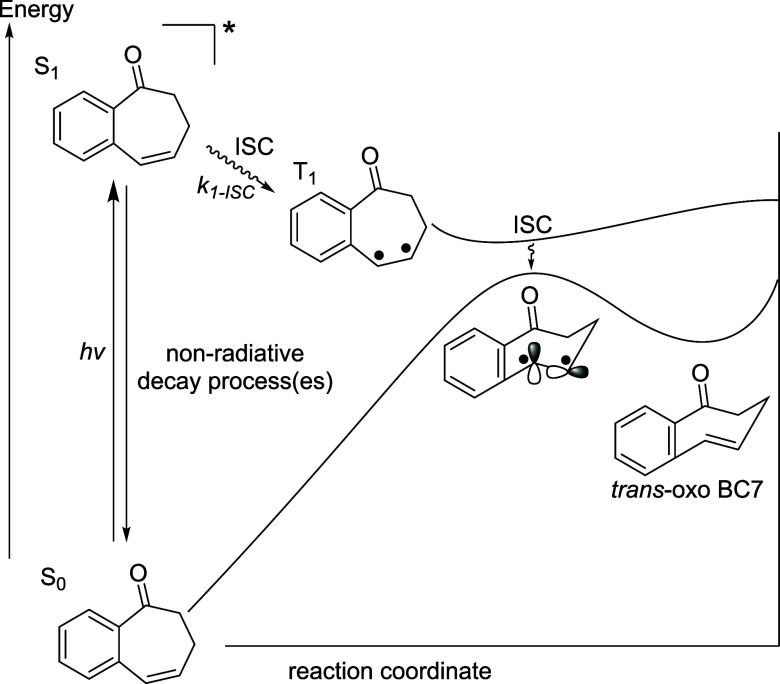
Energy profile for the *trans*-**oxo-BC7**.

Stated differently, we believe that the *S*_1_ of **oxo-BC7** is relatively slow to undergo ISC to the triplet, *T*_1_(*k*_1-isc_) (Figure 2). To address this challenge, we attempted to exploit the heavy atom effect.^22^ By introducing a heavy atom into **oxo-BC7** such as bromine, we aimed to facilitate ISC from the singlet to the triplet excited state. We then successfully synthesized **Broxo-BC7** (**4**, Scheme 7), in which a bromine atom was installed on the aromatic ring at the 3-position.^23^ The 3-position was selected primarily out of convenience as it could be easily synthesized via a Sandmeyer reaction from the corresponding 3-amino compound, which we had previously synthesized.^12^

**Scheme 7:**
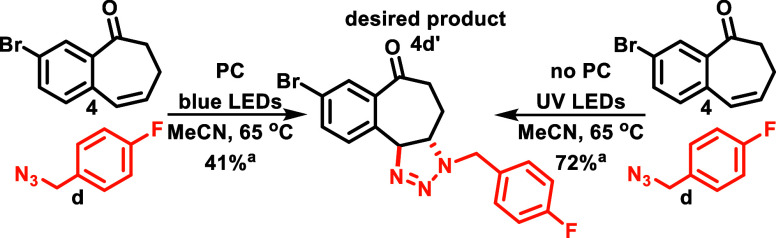
Initial Results in the Sensitizer-Free [3 + 2]-Cycloaddition NMR conv. for the triazoline formation is based on ^1^H NMR of the crude reaction mixture. PC implies 0.25 mol % Ir(ppy)_3_ was added to the reaction, and no PC implies no photocatalyst was added; see the SI for details.

In our initial attempts, we conducted the cycloaddition reaction using Ir(ppy)_3_ as the photocatalyst and blue LEDs as the light source (Scheme 7). This resulted in the formation of the desired triazoline product (**4d’**) with a conversion of 41% based on alkene as determined by 1H NMR of the crude reaction mixture. However, when we utilized UV-LEDs (370 ± 5 nm) without an external photosensitizer, we achieved a conversion of 72% to the same triazoline product within a 12 h time frame. This result supports our hypothesis about a competitive decay process from the *S*_1_ state in nonbrominated **3**.

Recognizing the importance of incorporating a heavy atom into the **oxo-BC7** motif to accelerate ISC to the triplet, *T*_1_, we synthesized^23^ another bromine substituted **BC7** variant with the bromine atom attached directly to the alkene (**Broxo-BC7, 5**). We hoped that if **5** was successful in the cycloaddition reaction, it would eliminate the need for a subsequent oxidation to obtain the triazole as the final product as the carbon would be preoxidized. When we subjected **5** (Scheme 8) to UV-LEDs (370 nm), only trace amounts of **3d’** were detected. Since aromatization would take place by the elimination of HBr to form the triazole, we speculated that the HBr was serving to destroy the triazoline intermediate, consistent with our earlier observations of acid sensitivity of the triazolines.^12^ Indeed, the addition of a base proved essential for this reaction, and we screened several bases. While stronger bases such as DBU and tetramethylguanidine failed, tertiary aliphatic- and pyridine-based amines performed well, with collidine giving the optimal results (see the SI).

**Scheme 8:**
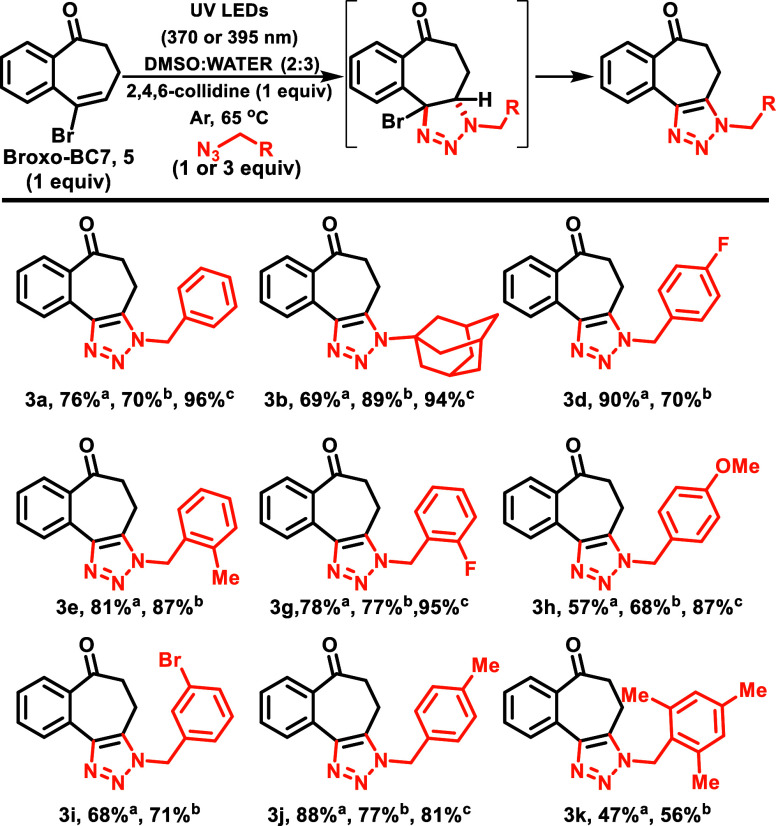
Substrate Scope with Broxo-BC7 (**5**) Isolated yields, alkene:azide (1:3). NMR yields, alkene:azide (1:1). NMR yields at 395 nm after 72 h.

Next, we evaluated different solvents,^24^ and we determined that DMSO provided the best results (88% NMR yield)^24^ and found that the reaction worked in other solvents (such as MeOH) that did not previously work in the photosensitized reaction of **oxo-BC7**.

Wanting to move toward aqueous reaction media, we subsequently investigated the impact of water addition on the reaction. Fortuitously, the inclusion of water expedited the reaction rate, leading to a nearly 4-fold decrease in the reaction time. As we progressively increased the amount of water, the reaction mixture became increasingly heterogeneous. We determined that the DMSO:H_2_O ratio of 2:3 yielded the most favorable outcomes, resulting in a 96% NMR yield on the worked-up material.^24^ Importantly, like the photosensitized cycloaddition of **oxo-BC7** (Scheme 4), we still observed only a single regioisomer. Thus, we began to explore the scope of direct photocycloaddition of **5** with various azides. We were also interested in assessing the outcome when azide and alkene were near-stoichiometric. Thus, in some cases, we explored the scope using either a 1:1 ratio of alkene:azide (conditions b, Scheme 8) or with an excess of azide (conditions a). In both cases, generally good to excellent yields were achieved. We also observed good yields at 395 nm, although it did require a longer reaction time (72 h), compared to 24 h at 370 nm. Importantly, based on the absorption spectrum, we anticipate the photonic efficiency decreases at longer wavelengths. However, the photon flux is not normalized; therefore, the reaction times are not comparable.

We next sought to broaden the scope of **Broxo-BC7** and enhance its versatility by incorporating additional cargo attachment points and improving its pharmacokinetic profile. To this end, we embarked on synthesizing a lactam-based alkene derivative **Br-lactam-BC7** (**8**, Scheme 10), which can be succinctly synthesized from alpha-tetralone.^23^ If successful, in future applications, this modification would be expected to serve as a convenient functional group handle for attaching a wide range of bioprobes and tools to **BC7**-derivatives.

We first investigated the **des-Bromo lactam BC7s** (**6** and **7**), which failed to give any reactivity with or without the photocatalyst, and at different wavelengths (Scheme 9). Given that **6** and **7** have reasonable absorption at lower wavelengths, it is safe to assume that excitation to *S*_1_ was occurring. The lack of reactivity may be explained by the poor ISC of **6** and **7** to the *T*_1_ or the slow quenching of the photocatalyst when present.^25^

**Scheme 9:**
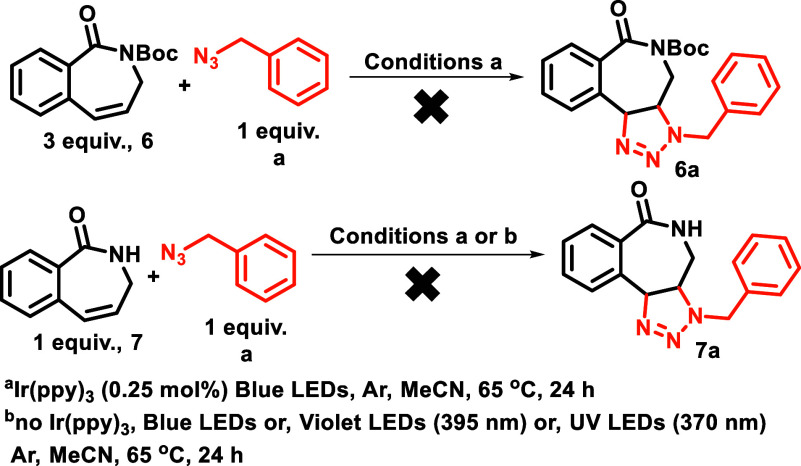
Cycloaddition with des-Bromo Lactam BC7s (**6** and **7**) IR(ppy)3 (0.25 mol %) blue LEDs, Ar, MeCN, 65 °C, 24 h. No Ir(ppy)3, blue LEDs or violet LEDs, (395 nm), or UV-LEDs (370 nm) Ar, MeCN, 65 °C, 24 h.

Next, we investigated whether **Br-lactam-BC7** could work and if it required a sensitizer. We were pleased to see that it could operate in the absence of an external sensitizer. Similar to **Broxo-BC7** (**5**), which could function with or without a sensitizer, **Br-lactam-BC7** (**8**) also worked with or without the sensitizer (Scheme 10). Given the anticipated differences in solubilities, we reassessed the reaction solvent with the **Br-lactam-BC7** and 4-fluoro benzyl azide. Based on the ^1^H NMR analysis of the crude reaction mixture, it was determined that MeOH demonstrated the highest conversion (56%). Subsequently, three different azides were reacted, and good isolated yields of the triazoles were obtained. Also, we observed great NMR yields at 395 nm, which were irradiated for 72 h.

**Scheme 10:**
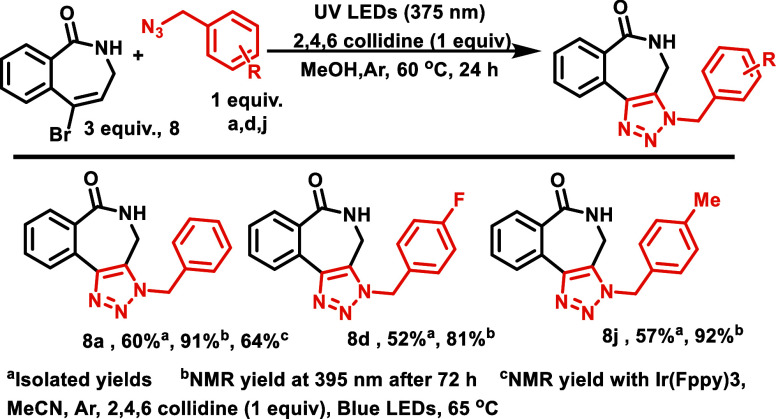
Substrate Scope with Br-Lactam-BC7 (**8**) Isolated yields. NMR yields at 395 nm after 72 h. NMR yields with IR(Fppy)3, MeCN, Ar, 2,4,6 collidine (1 equiv), blue LEDs, 65 °C.

In this study, we have investigated a second generation of benzocycloheptene derivatives that can successfully convert photochemical energy into ring strain, which facilitates [3 + 2]-cycloadditions with azides. The strained *trans*-cycloalkenes display vastly enhanced reactivity toward azides when compared to their unstrained isomers. The use of light to induce reactivity may result in enhanced spatial and temporal control^26^ that accompanies light-triggered reactions. These molecules add to the relatively small but growing group of conjugation strategies that can be made transiently reactive using light. We have also demonstrated a strategy for locking fragile triazoline intermediates into stable triazole products, which complements the previously demonstrated ability to decompose the triazoline.^12^ Additionally, we have shown that polarizing the alkene eliminates regioselectivity issues. Furthermore, in an effort to simplify the conditions and make them more broadly useful, we have made strides in eliminating the precious metal photocatalyst by both red-shifting the alkene’s absorbance to the near-visible by extended conjugation and by incorporating a bromine atom, which facilitates ISC from the singlet to the triplet. Furthermore, we have shown that the bromine can be located directly on the alkene (**Broxo-BC7**), which not only allows for photocatalyst-free conjugation but also serves to preserve this conjugation by in situ elimination and rearomatization to yield a robust triazole. Future efforts will explore these second-generation molecules in applications involving more complex systems.

## Supporting Information Available

^1^H, ^13^C, and ^19^F spectra, as well as key 2-D NMR spectra, other characterizations, and synthetic procedures (PDF)
NMR spectra information (ZIP)
