# Supplementary material for: Development of Transiently Strainable Benzocycloheptenes for Catalyst-Free, Visible-Light-Mediated [3 + 2]-Cycloadditions
Source: Bioconjug Chem. 2025 Feb 4;36(2):302–8. doi: 10.1021/acs.bioconjchem.4c00595 (PMC11843616; doi:10.1021/acs.bioconjchem.4c00595)

The Development of Transiently Strainable Benzocycloheptenes for Catalyst-Free, Visible Light Mediated [3+2]-Cycloadditions

Shivangi Kharbanda[a], Osaid Alkhamayseh[a], Georgia Eastham[b] and Jimmie D. Weaver III*[a]

[a]Department of Chemistry, Oklahoma State University, OK 74078

[b]Department of Chemistry, Massachusetts Institute of Technology, Cambridge, Massachusetts, 02139

Supporting information

General experimental **S2**

General procedures **S3**

Synthesis of alkene 1 (BC7) **S5**

The photocatalytic cycloaddition with BC7 and oxidation. **S5**

Synthesis of alkene 3 (oxo-BC7). **S8**

Optimization studies using oxo-BC7 **S9**

The photocatalytic cycloaddition with oxo-BC7 and oxidation. **S10**

Determination of the correct regioisomer. **S16**

Determination of cis vs trans-ring fusion. **S19**

Synthesis of alkene 5 (Br-oxo-BC7). **S22**

Absorption spectrum of alkene 5 (Br-oxo-BC7). **S22**

Optimization of the catalyst free, light driven cycloaddition with Br-oxo-BC7. **S22**

The photo-cycloaddition with 5 (Br-oxo-BC7). **S25**

Syntheses of alkenes 6 and 8 (Br-lactam-BC7). **S29**

Absorption spectra of alkenes 6 and 8. **S30**

The photo-cycloaddition with 8 (Br-lactam-BC7). **S31**

References **S33**

NMR spectra **S34**

General Experimental

All reagents were obtained from commercial suppliers (Aldrich, TCI chemicals, Oakwood chemicals, Alfa Aesar) and used without further purification unless otherwise noted. Some of the azides were purchased from Sigma Aldrich, while other azides were synthesized according to literature procedures. 1-Benzosuberone was purchased from TCI chemicals and Sigma Aldrich. Reactions were monitored by thin layer chromatography (TLC) which was obtained from sorbent technology (Silica XHL TLC Plates w/UV254, glass backed 250 μm, 20 x 20 cm), and were visualized with ultraviolet light. Photocatalyst *fac*-tris(2-phenyl pyridinato-C^2, *N*) iridium(III), (Ir(ppy)$_3$) and fac-tris-[2-(4-fluorophenyl) pyridinato-C^2, N)] Iridium(III) (Ir(Fppy)$_3$ was synthesized according to literature procedure.[1] Reactions were monitored by ^{1}H NMR, ^{19}F NMR and GC-MS (Chemical Ionization, QP 2020NX Shimadzu, equipped with an autosampler). NMR spectra were obtained on a 400 MHz Bruker Avance III spectrometer or Bruker Neo 800 MHz spectrometer equipped with a TCI cryo-probe. ^{1}H, ^{19}F NMR and ^{13}C chemical shifts are reported in ppm relative to the residual solvent peak. Absorption spectra were recorded on a Shimadzu UV-2600 UV-vis spectrometer and emission spectra were recorded on a Fluorolog3 – fluorescence spectrometer. Purifications were carried out using Teledyne Isco Combiflash Rf 200i flash chromatograph with Sorbtech Rf normal phase silica (4 g, 12 g or 24 g columns) with product detection at 254 and 288 nm. HRMS was obtained with a Thermo Scientific Orbitrap Fusion Tribrid Mass Spectrometer, utilizing the quadrupole mass analyzer.

General procedure A. The photocatalytic cycloaddition with BC7 and oxidation.

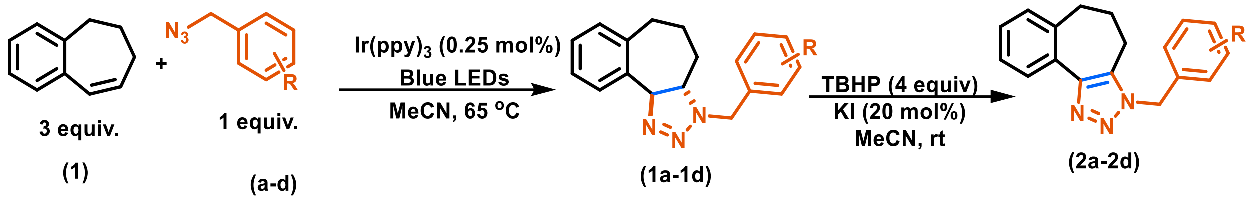

I. An NMR tube was charged with a solution of azide (1.0 equiv), BC7 (3 equiv), $Ir(ppy)_3$ (0. 25 mol%) in acetonitrile (0.2 M), and a sealed capillary containing deuterated benzene. The tube was capped with rubber septum and the reaction mixture was degassed with argon bubbling for 10-15 minutes. The tubes were then sealed with parafilm and placed in the light bath maintained at 65 °C *via* the use of a thermostat heating mantle. The progress of the reaction was monitored by ^{1}H NMR. To minimize unintentional reaction, the tube was quickly covered with aluminum foil after being removed from light bath and kept dark until loading into the NMR instrument. After completion of the photocycloaddition reaction, silica was added to the reaction mixture to decompose the minor regioisomer. After the complete decomposition- as indicated by 1H NMR, the reaction mixture was filtered from the silica and purified by normal phase chromatography using basic alumina as stationary phase and EtOAc/hexanes as mobile phase. The obtained triazoline was confirmed by ^{1}H and ^{13}C NMR and was further characterized after oxidation to the corresponding triazoles.

II. After isolation of the triazoline (major regioisomer), it was then added to the NMR tube (0.1 mmol) along with TBHP (4 equiv), KI (20 mol%), CH_3CN and a sealed capillary containing deuterated benzene, and the oxidation was monitored by ^{1}H NMR. After the complete consumption of triazoline, CH_3CN was removed under reduced pressure and the residue was treated with deionized water (5 mL) and extracted with EtOAc (2 x 5 mL). The combined organic portions were washed with aqueous sodium thiosulfate and brine solution, dried with anhydrous $MgSO_4$, filtered, concentrated to obtain the crude product that was purified by normal phase chromatography using silica as stationary phase and EtOAc/hexanes as mobile phase.

General procedure B. The photocatalytic cycloaddition with oxo-BC7 and oxidation (one-pot).

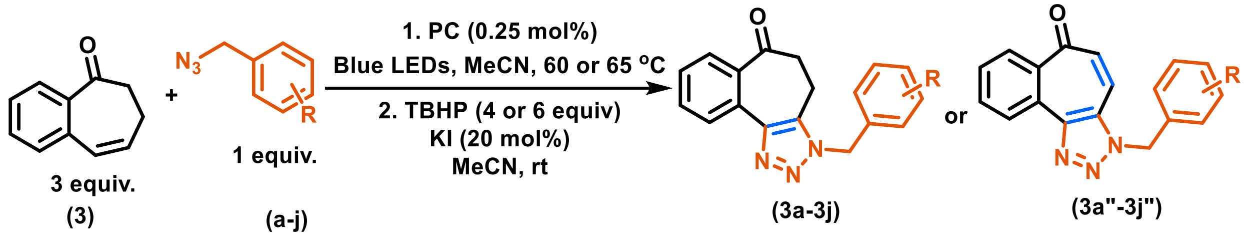

An NMR tube was charged with a solution of azide (1.0 equiv), oxo-BC7 (3 equiv), $Ir(ppy)_3$ or $Ir(Fppy)_3$ (0.25 mol%) in acetonitrile (0.2 M), and a sealed capillary containing deuterated

benzene. The tube was capped with rubber septum and the reaction mixture was degassed with argon bubbling for 10-15 minutes. The tubes were then sealed with parafilm and placed in the light bath maintained at 60 °C or 65 °C *via* the use of a thermostat heating mantle. The progress of the reaction was monitored by ^{1}H NMR. To minimize unintentional reaction, the tube was quickly covered with aluminum foil after being removed from light bath and kept under dark until loading into the NMR instrument. After completion of the photocycloaddition reaction, TBHP (4 equiv or 6 equiv) and KI (20 mol%) were added to the same NMR tube and the oxidation was monitored by ^{1}H NMR. After the complete consumption of triazoline, CH_3CN was removed under reduced pressure and the residue was treated with deionized water (5 mL) and extracted with EtOAc (2 x 5 mL). The combined organic portions were washed with aqueous sodium thiosulfate and brine solution, dried with anhydrous $MgSO_4$, filtered, concentrated to obtain the crude product (for the reactions done with the $Ir(ppy)_3$ as photocatalyst) that was purified by normal phase chromatography using basic alumina or silica as stationary phase and EtOAc/hexanes as mobile phase. In cases in which $Ir(Fppy)_3$ was used as photocatalyst, the yield was calculated (from ^{1}H NMR after workup) by adding 4-methyl benzyl chloride as an internal standard.

General procedure C. The photo-cycloaddition with Br-oxo-BC7.

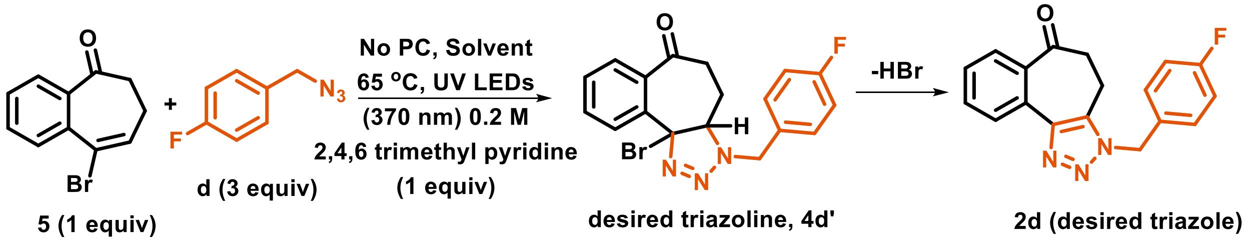

An NMR tube was charged with a solution of azide (1.0 or 3.0 equiv), Br-oxo-BC7 (1 equiv), 2,4,6 trimethyl pyridine (1 equiv.) in a solution of DMSO and water (2:3, v:v) (0.2 M). The tube was capped with rubber septum and the reaction mixture was degassed with argon bubbling for 10-15 minutes. The tubes were then sealed with parafilm and placed in the UV light (370 nm) bath maintained at 65 °C *via* the use of a thermostatic heating mantle. The reaction was irradiated for 24 hours. The reaction mixture was then extracted with EtOAc (2 x 5 mL) and deionized water (5 mL). The combined organic portions were washed brine solution, dried with anhydrous $MgSO_4$, filtered, concentrated to obtain the crude product that was purified by normal phase chromatography using silica as stationary phase and EtOAc/hexanes as mobile phase. In cases where 1.0 equiv of azide was used and the product had already been isolated and characterized, the yield was calculated by ^{1}H NMR (after workup) by adding 4-methyl benzyl chloride as an internal standard. In cases where the reaction was irradiated under 395 nm, yield was calculated by ^{1}H NMR (after workup) by adding 4-methyl benzyl chloride as an internal standard.

General procedure D. The photo-cycloaddition with Br-lactam-BC7.

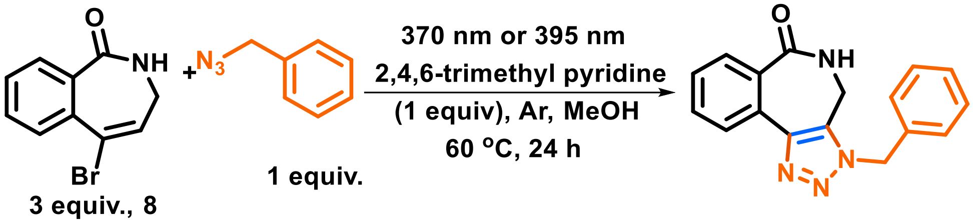

An NMR tube was charged with a solution of azide (1.0 equiv), Br-lactam-BC7 (3 equiv), 2,4,6 trimethyl pyridine (1 equiv) in MeOH (0.2 M). The tube was capped with rubber septum and the reaction mixture was degassed with argon bubbling for 10-15 minutes. The tubes were then sealed with parafilm and placed in the UV light (370 nm) bath maintained at 60 °C *via* the use of a thermostat heating mantle. The reaction was irradiated for 24 hours. The reaction mixture was then extracted with EtOAc (2 x 5 mL) and deionized water (5 mL). The combined organic portions were washed brine solution, dried with anhydrous $MgSO_4$, filtered, concentrated to obtain the crude product that was purified by normal phase chromatography using silica as stationary phase and EtOAc/hexanes as mobile phase. In cases where the reaction was irradiated under 395 nm, and the product had already been isolated and characterized, the yield was calculated by ^{1}H NMR (after workup) by adding 4-methyl benzyl chloride as an internal standard.

Synthesis of alkene 1 (BC7).

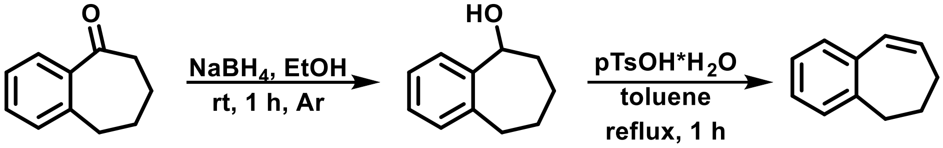

1

The procedure reported previously by our group[2] was followed and scaled to 500 mg (3.13 mmol) of **1-benzosuberone**, affording an 87% overall yield (392 mg, 2.72 mmol) of desired alkene **1**.

The photocatalytic cycloaddition with BC7 and oxidation.

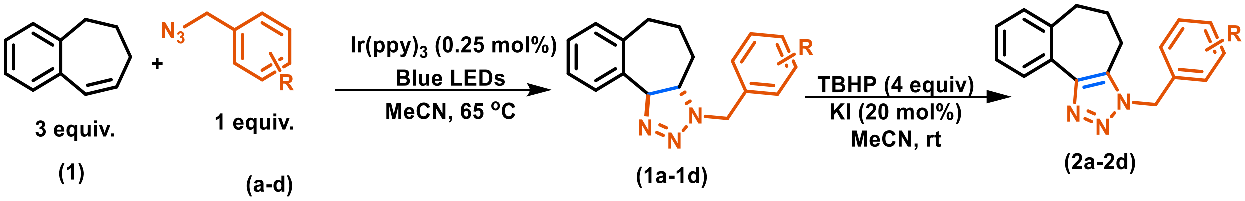

1a. 3-Benzyl-3,3a,4,5,6,10b-hexahydrobenzo[3,4]cyclohepta[1,2-d][1,2,3]triazole was

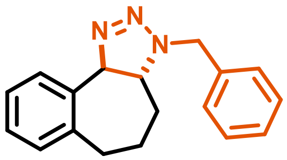

prepared by **general procedure A (I).** A mixture of benzyl azide (27 mg, 0.20 mmol), alkene **1** (87 mg, 0.60 mmol), **Ir(ppy)**$_3$ (0.25 mol%) and CH_3CN (0.2 M) was placed in the blue light bath at 65 °C to afford **1a** and **1a'** (the minor regioisomer) as a yellow oil. Silica was added to decompose the minor regioisomer, **1a'**. After decomposition, this mixture was purified by column chromatography using basic alumina (EtOAc/hexanes = 5:95) to afford **1a** (30 mg, 54%) ^{1}H NMR (400 MHz, CD_3CN) δ 7.6 (dd, J = 7.4, 1.6 Hz, 1H), 7.3 – 7.3 (m, 4H), 7.3 – 7.2 (m, 1H), 7.2 – 7.1 (m, 2H), 7.1 – 7.1 (m, 1H), 5.0 (d, J = 16.2 Hz, 1H), 4.7 (d, J = 15.0 Hz, 1H), 4.6 (d, J = 15.1 Hz, 1H), 2.7 (dd, J = 8.2, 3.3 Hz, 2H), 2.4 (ddd, J = 16.2, 11.1, 3.3 Hz, 1H), 2.3 (dq, J = 12.9, 3.5 Hz, 1H), 1.6 (tdd, J = 13.0, 11.0, 3.6 Hz, 1H), 1.2 – 1.1 (m, 1H). ^{13}C NMR (101 MHz, CD_3CN) δ 141.6, 139.6, 137.3, 130.7, 129.7, 129.5, 128.6, 128.3, 127.5, 125.4, 83.3, 66.3, 53.9, 35.4, 32.7, 25.8.

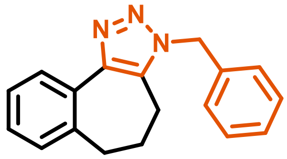

2a. 3-Benzyl-3,4,5,6-tetrahydrobenzo[3,4]cyclohepta[1,2-d][1,2,3]triazole was prepared by **general procedure A (II)**. **1a** (30 mg, 0.11 mmol) was then oxidized by TBHP (4 equiv, 42 µL), KI (20 mol%, 3.7 mg) at room temperature to obtain **2a** (28 mg) in 94% yield after purification by column chromatography using silica (EtOAc/hexanes = 10:90). ^{1}H NMR (400 MHz, CD_3CN) δ 8.3 (dd, J = 7.8, 1.4 Hz, 1H), 7.4 – 7.3 (m, 3H), 7.3 (td, J = 7.5, 1.7 Hz, 1H), 7.2 – 7.2 (m, 2H), 7.2 (dd, J = 7.2, 1.4 Hz, 1H), 7.2 – 7.1 (m, 1H), 5.5 (s, 2H), 2.9 – 2.8 (m, 4H), 2.0 – 1.9 (m, 2H). ^{13}C NMR (101 MHz, $CDCl_3$) δ 144.1, 139.6, 134.8, 133.4, 130.0, 129.5, 129.1, 128.4, 127.6, 127.3, 127.1, 126.8, 52.1, 35.2, 25.6, 23.5. Calculated HRMS(ESI) for $C_{18}H_{17}N_3$ (M+H) + is 276.1495 observed 276.1495.

1b. 3-((3s,5s,7s)-Adamantan-1-yl)-3,3a,4,5,6,10b-hexahydrobenzo[3,4]cyclohepta[1,2-d][1,2,3]triazole was prepared by **general procedure A**. A mixture of 1-azidoadamantane (36

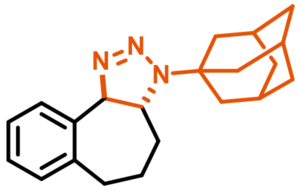

mg, 0.20 mmol), alkene **1** (87 mg, 0.60 mmol), **Ir(ppy)**$_3$ (0.25 mol%) and CH_3CN (0.2 M) was placed in the blue light bath at 65 °C to afford **1b** and **1b'** as a yellow oil. Silica was added to decompose the minor regioisomer (**1b'**). After decomposition, this mixture was purified by column chromatography using basic alumina (EtOAc/hexanes = 5:95) to afford **1b** (42 mg, 65%). ^{1}H NMR (400 MHz, CD_3CN) δ 7.57 – 7.52 (m, 1H), 7.27 – 7.13 (m, 3H), 5.01 (d, J = 15.5 Hz, 1H), 2.95 (ddd, J = 15.4, 10.6, 2.9 Hz, 1H), 2.87 – 2.77 (m, 2H), 2.61 (dq, J = 13.1, 3.4 Hz, 1H), 2.15 – 1.96 (m, 7H), 1.82 (ddt, J = 10.9, 3.7, 1.9 Hz, 3H), 1.75 (ddd, J = 13.3, 10.5, 3.3 Hz, 1H), 1.69 (dd, J = 3.9, 2.1 Hz, 6H), 1.44 – 1.24 (m, 1H). ^{13}C NMR (101 MHz, CD_3CN) δ 141.8, 140.5, 130.3, 128.1, 127.5, 125.3, 84.1, 63.3, 59.1, 41.9, 37.5, 36.9, 35.5, 30.6, 26.4.

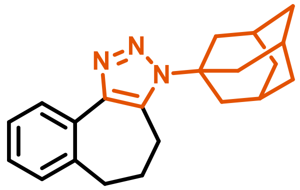

2b. 3-((3s,5s,7s)-Adamantan-1-yl)-3,4,5,6-tetrahydrobenzo[3,4]cyclohepta[1,2-d][1,2,3]triazole was prepared by **general procedure A (II)**. **1b** (42 mg, 0.14 mmol) was then oxidized by TBHP (4 equiv, 54 µL), KI (20 mol%, 4.6 mg) to obtain **2b** (37.5 mg) as yellow solid in 90% yield after purification by column chromatography using silica (EtOAc/hexanes = 10:90). ^{1}H NMR (400 MHz, $CDCl_3$) δ 8.35 (dd, *J* = 7.8, 1.4 Hz, 1H), 7.29 (td, *J* = 7.6, 1.5 Hz, 1H), 7.18 (td, *J* = 7.4, 1.4 Hz, 1H), 7.11 (dd, *J* = 7.6, 1.5 Hz, 1H), 3.18 (t, *J* = 6.6 Hz, 2H), 2.93 – 2.78 (m, 2H), 2.42 (d, *J* = 3.0 Hz, 6H), 2.32 – 2.22 (m, 3H), 2.09 (dt, *J* = 10.6, 6.4 Hz, 2H), 1.79 (t, *J* = 3.1 Hz, 6H). ^{13}C NMR (101 MHz, $CDCl_3$) δ 144.7, 139.8, 133.3, 131.0, 129.0, 127.7, 127.3, 126.6, 62.6, 41.7, 36.1, 35.0, 29.8, 28.2, 25.3. Calculated HRMS(ESI) for $C_{21}H_{25}N_3$ (M+H) + is 320.2121 observed 320.2124. mp – 174-176 °C.

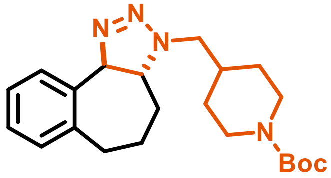

1c. tert-Butyl 4-((4,5,6,10b-tetrahydrobenzo[3,4]cyclohepta[1,2-d][1,2,3]triazol-3(3aH)-yl)methyl)piperidine-1-carboxylate was prepared by **general procedure A (I)**. A mixture of *tert*-butyl 4-(azidomethyl)piperidine-1-carboxylate (48 mg, 0.20 mmol), alkene **1** (87 mg, 0.60 mmol), **Ir(ppy)$_3$** (0.25 mol%) and CH_3CN (0.2 M) was placed in the blue light bath at 65 °C to afford **1c** and **1c'** as a yellow oil. Silica was added to decompose the minor regioisomer (**1c'**). After decomposition, this mixture was purified by column chromatography using basic alumina (EtOAc/hexanes = 5:95) to afford **1c** (50 mg, 65%). ^{1}H NMR (400 MHz, CD_3CN) δ 7.72 – 7.65 (m, 1H), 7.24 (td, *J* = 7.2, 2.1 Hz, 1H), 7.22 – 7.14 (m, 2H), 4.93 (d, *J* = 16.4 Hz, 1H), 4.11 – 3.99 (m, 2H), 3.38 (dd, *J* = 13.7, 5.9 Hz, 1H), 3.02 (dd, *J* = 13.7, 8.4 Hz, 1H), 2.87 – 2.77 (m, 2H), 2.77 – 2.56 (m, 2H), 2.41 (dq, *J* = 12.7, 3.4 Hz, 1H), 2.13 – 2.00 (m, 1H), 1.97 (s, 1H), 1.76 – 1.64 (m, 3H), 1.42 (s, 9H), 1.37 – 1.25 (m, 1H), 1.20 – 1.02 (m, 2H). ^{13}C NMR (101 MHz, CD_3CN) δ 155.4, 141.8, 139.7, 130.7, 128.3, 127.6, 125.7, 83.0, 79.7, 67.8, 55.6, 44.1, 36.0, 35.6, 33.2, 31.0, 28.8, 26.0.

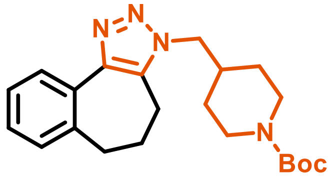

2c. tert-Butyl 4-((5,6-dihydrobenzo[3,4]cyclohepta[1,2-d][1,2,3]triazol-3(4H)-yl)methyl)piperidine-1-carboxylate was prepared by **general procedure A (II)**. **1c** (50 mg, 0.13 mmol) was then oxidized by TBHP (4 equiv, 50 µL), KI (20 mol%, 4.3 mg) to obtain **2c** (45.7 mg) as yellow solid in 92% yield after purification by column chromatography using silica (EtOAc/hexanes = 10:90). ^{1}H NMR (400 MHz, CD_3CN) δ 8.24 (dd, *J* = 7.7, 1.3 Hz, 1H), 7.28 (ddd, *J* = 7.8, 6.7, 2.1 Hz, 1H), 7.26 – 7.13 (m, 2H), 4.12 (d, *J* = 7.4 Hz, 2H), 4.05 (td, *J* = 11.7, 5.7 Hz, 3H), 2.93 (t, *J* = 6.6 Hz, 2H), 2.90 – 2.86 (m, 2H), 2.12 (dqd, *J* = 11.4, 7.5, 3.7 Hz, 1H), 2.02 (qd, *J* = 6.6, 2.9 Hz, 2H), 1.62 – 1.54 (m, 2H), 1.41 (s, 10H), 1.27 – 1.11 (m, 3H). ^{13}C NMR (101 MHz, $CDCl_3$) δ 154.8, 143.4, 139.5, 133.2, 130.0, 129.5, 127.6, 127.1, 126.8, 79.7, 53.0, 43.5, 37.1, 35.2, 29.8, 28.5, 25.8, 23.6. Calculated HRMS(ESI) for $C_{22}H_{30}N_4O_2$ (M+H)+ is 383.2442 observed 383.2443. mp – 143-145 °C.

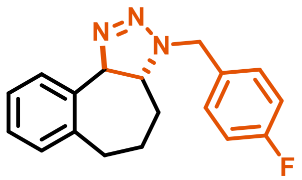

1d. 3-(4-Fluorobenzyl)-3,3a,4,5,6,10b-hexahydrobenzo[3,4]cyclohepta[1,2-d][1,2,3]triazole was prepared by **general procedure A (I)**. A mixture of 1-(azidomethyl)-4-fluorobenzene (30.2 mg, 0.20 mmol), alkene **1** (87 mg, 0.60 mmol), **Ir(ppy)₃** (0.25 mol%) and CH_3CN (0.2 M) was placed in the blue light bath at 65 °C to afford **1d** and **1d'** as a yellow oil. Silica was added to decompose the minor regioisomer (**1d'**). After decomposition, this mixture was purified by column chromatography using basic alumina (EtOAc/hexanes = 5:95) to afford **1d** (43 mg, 72%). ^{1}H NMR (400 MHz, d6-Acetone) δ 7.70 – 7.67 (m, 1H), 7.44 – 7.36 (m, 2H), 7.21 (td, *J* = 7.2, 2.0 Hz, 1H), 7.14 (qd, *J* = 7.5, 2.0 Hz, 2H), 7.13 – 7.03 (m, 2H), 5.02 (d, *J* = 16.1 Hz, 1H), 4.76 (d, *J* = 15.1 Hz, 1H), 4.68 (d, *J* = 15.1 Hz, 1H), 2.89 – 2.71 (m, 2H), 2.50 (ddd, *J* = 16.1, 11.1, 3.4 Hz, 1H), 2.35 (dq, *J* = 12.9, 3.5 Hz, 1H), 2.03 – 1.96 (m, 1H), 1.80 – 1.63 (m, 1H), 1.32 – 1.15 (m, 1H). ^{13}C NMR (101 MHz, d6-Acetone) δ 163.9 (d, *J* = 244.0 Hz), 142.2, 140.2, 134.3 (d, *J* = 3.3 Hz), 132.3 (d, *J* = 8.2 Hz), 131.3, 128.9, 128.2, 126.3, 116.8 (d, *J* = 21.4 Hz), 84.3, 67.0, 53.9, 36.2, 33.5, 26.7. ^{19}F NMR (376 MHz, d6-Acetone) δ -116.26 (tt, *J* = 8.9, 4.4 Hz).

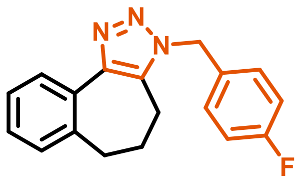

2d. 3-(4-Fluorobenzyl)-3,4,5,6-tetrahydrobenzo[3,4]cyclohepta[1,2-d][1,2,3]triazole was prepared by **general procedure A (II)**. **1d** (43 mg, 0.15 mmol) was then oxidized by TBHP (4 equiv, 58 µL), KI (20 mol%, 5.0 mg) to obtain **2d** (38.3 mg) as yellow solid in 87% yield after purification by column chromatography using silica (EtOAc/hexanes = 10:90). ^{1}H NMR (400 MHz, CD_3CN) δ 8.24 (dd, *J* = 7.8, 1.4 Hz, 1H), 7.32 – 7.23 (m, 3H), 7.18 (td, *J* = 7.3, 1.5 Hz, 1H), 7.14 (dd, *J* = 7.5, 1.6 Hz, 1H), 7.12 – 7.05 (m, 2H), 5.47 (s, 2H), 2.85 – 2.78 (m, 4H), 2.00 – 1.89 (m, 2H). ^{13}C NMR (101 MHz, $CDCl_3$) δ 162.7 (d, *J* = 247.5 Hz), 144.2, 139.6, 133.2, 130.5 (d, *J* = 3.3 Hz), 129.9, 129.5, 129.1 (d, *J* = 8.2 Hz), 127.6, 127.1, 126.8, 116.1 (d, *J* = 21.7 Hz), 51.3, 35.1, 25.5, 23.4. ^{19}F NMR (376 MHz, CD_3CN) δ -115.90 (tt, *J* = 9.5, 5.4 Hz). Calculated HRMS(ESI) for $C_{18}H_{16}FN_3$ (M+H)+ is 294.1401 observed 294.1404. mp – 125-127 °C.

Synthesis of alkene 3 (oxo-BC7).

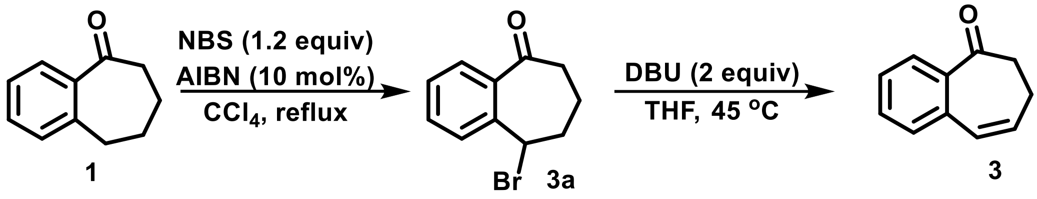

Alkene 3 was synthesized by the reported procedure.[3]

Optimization studies for oxo-BC7.

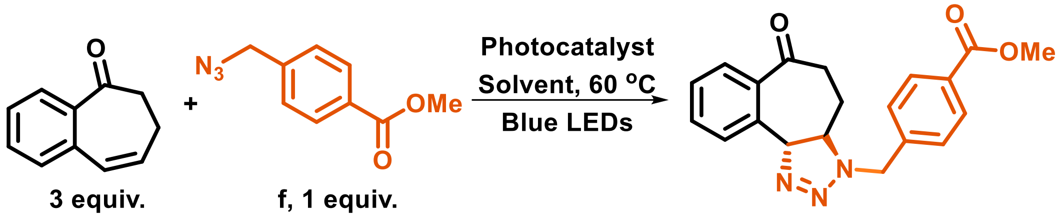

Methyl 4-(azidomethyl)benzoate (**f**, SI Table 1) was chosen as the model substrate along with **oxo-BC7** for this study. Firstly, we screened different photocatalysts which revealed the most anticipated results. Namely, that the homoleptic iridium class of photocatalysts which have been indicated as excellent energy transfer catalysts performed well, with Ir(Fppy)3 giving the optimal result. We next evaluated solvents, which revealed MeCN as the optimal solvent. A screen of photocatalyst loading showed 0.25 mol% to be optimal for our specific conditions, i.e., reactor and path length but continued to work at significantly lower loadings. Finally, the reaction concentrations were varied while maintaining the relative amounts as indicated in Table1 which showed that the reaction could be concentrated significantly without detriment. However, given the scale of these reactions were performed at, 0.1 M was generally used.

SI Table 1. Optimization of the reaction conditions[a]

	Entry	Solvent	0.1 mol% PC	Conc. w.r.t azide (M)	NMR conv.
Photocatalyst Screening	1	MeCN	$Ir(ppy)_3$	0.1	64%
	2	MeCN	2-CzPn	0.1	7%
	3	MeCN	$Ir(Fppy)_3$	0.1	88%
	4	MeCN	$Ir(dFppy)_3$	0.1	76%
	5	MeCN	$Ir(4'\text{-}Meppy)_3$	0.1	79%
	6	MeCN	$Ir(Ftbuppy)_3$	0.1	82%
	7	MeCN	$Ru(bpy)_3PF_6$	0.1	0%

	Entry	Solvent	$Ir(Fppy)_3$ mol%	Conc. w.r.t azide (M)	NMR conv.
Photocatalyst Loading	14	MeCN	0.05	0.1	68%
	15	MeCN	0.1	0.1	85%
	16	MeCN	0.25	0.1	96%
	17	MeCN	0.5	0.1	91%
	18	MeCN	1	0.1	87%
	19	MeCN	1.2	0.1	79%

	Entry	Solvent	$Ir(Fppy)_3$ mol%	Conc. w.r.t azide (M)	NMR conv.
Solvent Screening	8	THF	0.1	0.1	68%
	9	DMF	0.1	0.1	28%
	10	MeOH	0.1	0.1	0%
	11	MeCN	0.1	0.1	88%
	12	EtOAc	0.1	0.1	45%
	13	DMSO	0.1	0.1	0%

	Entry	Solvent	$Ir(Fppy)_3$ mol%	Conc. w.r.t azide (M)	NMR conv.
Reaction Concentration	20	MeCN	0.25	0.05	80%
	21	MeCN	0.25	0.1	96%
	22	MeCN	0.25	0.2	100%
	23	MeCN	0.25	0.5	100%

[a]NMR conv. for the product formation are based on ^1H NMR. [a]All the reactions were stopped at t = 11 h.

Optimized conditions:	Argon purging	No Argon purging
$Ir(Fppy)_3$ (0.25mol%)<br>MeCN (0.1 M)<br>65°C, Blue LEDs	82%[b]	77%[b]

[b]NMR conv. for the product formation are based on ^1H NMR at t = 6h.

The photocatalytic cycloaddition with oxo-BC7 and oxidation.

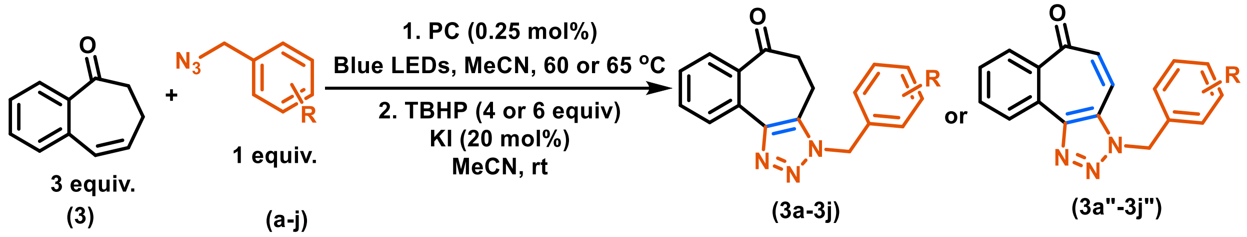

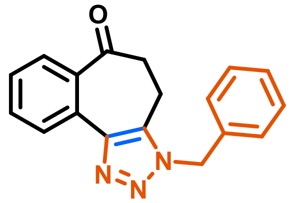

3a. 3-Benzyl-4,5-dihydrobenzo[3,4]cyclohepta[1,2-d][1,2,3]triazol-6(3H)-one was prepared by **general procedure B**. A mixture of benzyl azide (27 mg, 0.20 mmol), alkene **3** (87 mg, 0.60 mmol), **Ir(ppy)3** (0.25 mol%) and CH_3CN (0.2 M) was placed in the blue light bath at 65 °C to afford triazoline which was then oxidized by TBHP (4 equiv, 77 µL), KI (20 mol%, 6.6 mg) to obtain **3a** (50.3 mg) in 87% yield as yellow oil after purification by column chromatography using silica (EtOAc/hexanes = 20:80). ^{1}H NMR (400 MHz, CD_3CN) δ 8.57 (dd, *J* = 8.0, 1.2 Hz, 1H), 7.75 (dd, *J* = 7.9, 1.5 Hz, 1H), 7.62 (ddd, *J* = 7.9, 7.3, 1.5 Hz, 1H), 7.40 – 7.30 (m, 4H), 7.26 – 7.22 (m, 2H), 5.56 (s, 2H), 3.00 – 2.93 (m, 4H). ^{13}C NMR (101 MHz, CD_3CN) δ 201.8, 143.9, 137.2, 136.3, 135.7, 133.5, 130.9, 130.0, 129.9, 129.2, 128.3, 128.3, 126.8, 52.5, 39.7, 20.1. Calculated HRMS(ESI) for $C_{18}H_{15}N_3O$ (M+H)+ is 290.1288 observed 290.1287.

Ir(Fppy)3 as photocatalyst: A mixture of benzyl azide (13.3 mg, 0.10 mmol), alkene **3** (47.4 mg, 0.30 mmol), Ir(Fppy)3 (0.25 mol%) and CH_3CN (0.2 M) was placed in the blue light bath at 60 °C to afford triazoline which was then oxidized by TBHP (4 equiv, 39 µL), KI (20 mol%, 3.3 mg) to obtain **3a** yield (84%) calculated from 1H NMR, (after workup) by adding 4-methyl benzyl chloride as an internal standard.

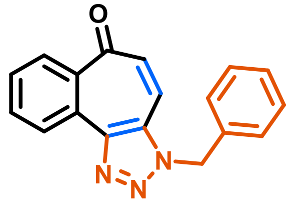

3a". 3-Benzylbenzo[3,4]cyclohepta[1,2-d][1,2,3]triazol-6(3H)-one was prepared by **general procedure B**. A mixture of benzyl azide (27 mg, 0.20 mmol), alkene **3** (87 mg, 0.60 mmol), **Ir(ppy)3** (0.25 mol%) and CH_3CN (0.2 M) was placed in the blue light bath at 65 °C to afford triazoline which was then oxidized by TBHP (6 equiv, 116 µL), KI (20 mol%, 6.6 mg) to obtain **3a"** (49.4 mg) in 86% yield as yellow solid after purification by column chromatography using silica (EtOAc/hexanes = 15:85). ^{1}H NMR (400 MHz, Acetone) δ 8.96 (dd, *J* = 8.1, 1.3 Hz, 1H), 8.33 (dd, *J* = 8.2, 1.4 Hz, 1H), 7.88 (ddd, *J* = 8.2, 7.1, 1.5 Hz, 1H), 7.77 (d, *J* = 12.2 Hz, 1H), 7.70 (ddd, *J* = 8.4, 7.1, 1.4 Hz, 1H), 7.44 – 7.33 (m, 5H), 6.83 (d, *J* = 12.2 Hz, 1H), 6.01 (s, 2H). ^{13}C NMR (201 MHz, Acetone) δ 187.5, 145.6, 135.9, 135.5, 133.3, 132.9, 130.80, 130.75, 129.6, 129.4, 129.0, 128.3, 127.3, 126.6, 121.2, 51.8. Calculated HRMS(ESI) for $C_{18}H_{13}N_3O$ (M+H) + is 288.1131 observed 288.1137. mp – 118-120 °C.

Ir(Fppy)3 as photocatalyst: A mixture of benzyl azide (13.3 mg, 0.10 mmol), alkene **3** (47.4 mg, 0.30 mmol), Ir(Fppy)3 (0.25 mol%) and CH_3CN (0.2 M) was placed in the blue light bath at 60 °C to afford triazoline which was then oxidized by TBHP (6 equiv, 58 µL), KI (20 mol%, 3.3 mg) to

obtain **3a”** yield (70%) calculated from 1H NMR, (after workup) by adding 4-methyl benzyl chloride as an internal standard.

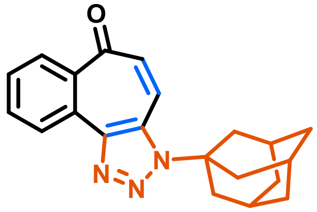

3b”. 3-((3s,5s,7s)-Adamantan-1-yl)benzo[3,4]cyclohepta[1,2-d][1,2,3]triazol-6(3H)-one was prepared by **general procedure B**. A mixture of 1-azidoadamantane (36 mg, 0.20 mmol), alkene **3** (87 mg, 0.60 mmol), **Ir(ppy)3** (0.25 mol%) and CH_3CN (0.2 M) to afford triazoline which was then oxidized by TBHP (6 equiv, 116 µL), KI (20 mol%, 6.6 mg) to obtain **3b”** (62.2 mg) in 94% yield as white solid after purification by column chromatography using silica (EtOAc/hexanes = 15:85). ^{1}H NMR (800 MHz, $CDCl_3$) δ 9.01 (dd, *J* = 8.1, 1.2 Hz, 1H), 8.36 (dd, *J* = 8.1, 1.3 Hz, 1H), 7.84 (d, *J* = 12.4 Hz, 1H), 7.79 (ddd, *J* = 8.2, 7.0, 1.4 Hz, 1H), 7.63 (ddd, *J* = 8.2, 7.0, 1.3 Hz, 1H), 6.85 (d, *J* = 12.4 Hz, 1H), 2.51 (d, *J* = 3.0 Hz, 6H), 2.36 (p, *J* = 3.1 Hz, 3H), 1.87 (t, *J* = 3.2 Hz, 6H). ^{13}C NMR (201 MHz, $CDCl_3$) δ 189.0, 147.4, 136.0, 133.1, 132.0, 130.7, 130.2, 129.9, 129.8, 127.5, 122.6, 63.5, 42.6, 36.1, 29.9. Calculated HRMS(ESI) for $C_{21}H_{21}N_3O$ (M+H)+ is 332.1757 observed 332.1761. mp – 190-192 °C.

Ir(Fppy)3 as photocatalyst: A mixture of 1-azidoadamantane (17.7 mg, 0.10 mmol), alkene **3** (47.4 mg, 0.30 mmol), Ir(Fppy)3 (0.25 mol%) and CH_3CN (0.2 M) was placed in the blue light bath at 60 °C to afford triazoline which was then oxidized by TBHP (6 equiv, 58 µL), KI (20 mol%, 3.3 mg) to obtain **3b”** yield (83%) calculated from 1H NMR, (after workup) by adding 4-methyl benzyl chloride as an internal standard.

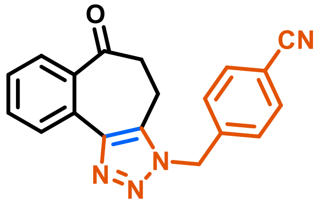

3c. 4-((6-Oxo-5,6-dihydrobenzo[3,4]cyclohepta[1,2-d][1,2,3]triazol-3(4H)-yl)methyl)benzonitrile was prepared by **general procedure B**. A mixture of 4-(azidomethyl)benzonitrile (31.6 mg, 0.20 mmol), alkene **3** (87 mg, 0.60 mmol), **Ir(ppy)3** (0.25 mol%) and CH_3CN (0.2 M) was placed in the blue light bath at 65 °C to afford triazoline which was then oxidized by TBHP (4 equiv, 77 µL), KI (20 mol%, 6.6 mg) to obtain **3c** (56 mg) in 89% yield as yellow solid after purification by column chromatography using silica (EtOAc/hexanes = 20:80). ^{1}H NMR (800 MHz, CD_3CN) δ 8.60 (dd, *J* = 8.0, 1.2 Hz, 1H), 7.78 (dd, *J* = 7.8, 1.4 Hz, 1H), 7.75 – 7.73 (m, 2H), 7.65 (ddd, *J* = 8.0, 7.3, 1.5 Hz, 1H), 7.41 (td, *J* = 7.5, 1.3 Hz, 1H), 7.39 – 7.37 (m, 2H), 5.66 (s, 2H), 3.02 – 2.99 (m, 2H), 2.99 – 2.94 (m, 2H). ^{13}C NMR (201 MHz, CD_3CN) δ 201.7, 144.0, 141.5, 137.2, 135.9, 133.7, 133.5, 130.7, 130.0, 129.0, 128.4, 126.8, 119.3, 112.7, 51.9, 39.6, 20.0. Calculated HRMS(ESI) for $C_{19}H_{14}N_4O$ (M+H)+ is 315.1240 observed 315.1243. mp – 130-132 °C.

Ir(Fppy)3 as photocatalyst: A mixture of 4-(azidomethyl)benzonitrile (15.8 mg, 0.10 mmol), alkene **3** (47.4 mg, 0.30 mmol), Ir(Fppy)3 (0.25 mol%) and CH_3CN (0.2 M) was placed in the blue light bath at 60 °C to afford triazoline which was then oxidized by TBHP (4 equiv, 39 µL), KI (20

mol%, 3.3 mg) to obtain **3c** yield (88%) calculated from ^{1}H NMR, (after workup) by adding 4-methyl benzyl chloride as an internal standard.

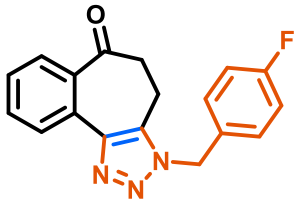

3d. 3-(4-Fluorobenzyl)-4,5-dihydrobenzo[3,4]cyclohepta[1,2-d][1,2,3]triazol-6(3H)-one was prepared by **general procedure B**. A mixture of 1-(azidomethyl)-4-fluorobenzene (30.2 mg, 0.20 mmol), alkene **3** (87 mg, 0.60 mmol), **Ir(ppy)$_3$** (0.25 mol%) and CH_3CN (0.2 M) was placed in the blue light bath at 65 °C to afford triazoline which was then oxidized by TBHP (4 equiv, 77 µL), KI (20 mol%, 6.6 mg) to obtain **3d** (56.5 mg) in 92% yield as yellow oil after purification by column chromatography using silica (EtOAc/hexanes = 20:80). ^{1}H NMR (400 MHz, CD_3CN) δ 8.55 (dd, *J* = 8.0, 1.2 Hz, 1H), 7.73 (dd, *J* = 7.8, 1.4 Hz, 1H), 7.60 (td, *J* = 7.7, 1.5 Hz, 1H), 7.36 (td, *J* = 7.6, 1.2 Hz, 1H), 7.27 (dd, *J* = 8.6, 5.4 Hz, 2H), 7.09 (t, *J* = 8.8 Hz, 2H), 5.53 (s, 2H), 3.00 – 2.92 (m, 4H). ^{13}C NMR (101 MHz, CD_3CN) δ 201.8, 163.4 (d, *J* = 244.7 Hz), 143.9, 137.1, 135.6, 133.4, 132.3 (d, *J* = 3.2 Hz), 130.8, 130.5 (d, *J* = 8.4 Hz), 129.9, 128.3, 126.7, 116.6 (d, *J* = 21.9 Hz), 51.8, 39.6, 20.1. ^{19}F NMR (376 MHz, CD_3CN) δ -115.63 (tt, *J* = 14.3, 5.4 Hz). Calculated HRMS(ESI) for $C_{18}H_{14}FN_3O$ (M+H)+ is 308.1194 observed 308.1198.

Ir(Fppy)$_3$ as photocatalyst: A mixture of 1-(azidomethyl)-4-fluorobenzene (15.1 mg, 0.10 mmol), alkene **3** (47.4 mg, 0.30 mmol), Ir(Fppy)$_3$ (0.25 mol%) and CH_3CN (0.2 M) was placed in the blue light bath at 60 °C to afford triazoline which was then oxidized by TBHP (4 equiv, 39 µL), KI (20 mol%, 3.3 mg) to obtain **3d** yield (95%) calculated from 1H NMR, (after workup) by adding 4-methyl benzyl chloride as an internal standard.

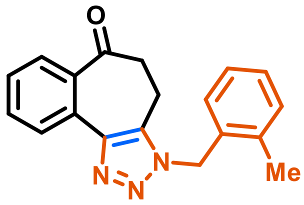

3e. 3-(2-Methylbenzyl)-4,5-dihydrobenzo[3,4]cyclohepta[1,2-d][1,2,3]triazol-6(3H)-one was prepared by **general procedure B**. A mixture of 1-(azidomethyl)-2-methylbenzene (29.4 mg, 0.20 mmol), alkene **3** (87 mg, 0.60 mmol), **Ir(ppy)$_3$** (0.25 mol%) and CH_3CN (0.2 M) was placed in the blue light bath at 65 °C to afford triazoline which was then oxidized by TBHP (4 equiv, 77 µL), KI (20 mol%, 6.6 mg) to obtain **3e** (58.2 mg) in 96% yield as yellow oil after purification by column chromatography using silica (EtOAc/hexanes = 20:80). ^{1}H NMR (800 MHz, $CDCl_3$) δ 8.68 (dd, *J* = 8.0, 1.2 Hz, 1H), 7.79 (dd, *J* = 7.7, 1.5 Hz, 1H), 7.61 (td, *J* = 7.6, 1.5 Hz, 1H), 7.35 (td, *J* = 7.5, 1.3 Hz, 1H), 7.23 (dtd, *J* = 14.7, 7.6, 1.4 Hz, 2H), 7.15 (td, *J* = 7.5, 1.6 Hz, 1H), 6.78 (d, *J* = 7.7 Hz, 1H), 5.57 (s, 2H), 3.05 – 2.99 (m, 2H), 2.87 – 2.81 (m, 2H), 2.37 (s, 3H). ^{13}C NMR (201 MHz, $CDCl_3$) δ 201.1, 143.8, 136.2, 135.9, 134.0, 133.0, 132.4, 131.1, 129.7, 129.3, 128.7, 127.8, 127.1, 126.8, 126.6, 50.7, 39.1, 19.8, 19.4. Calculated HRMS(ESI) for $C_{19}H_{17}N_3O$ (M+H)+ is 304.1444 observed 304.1450.

Ir(Fppy)$_3$ as photocatalyst: A mixture of 1-(azidomethyl)-4-fluorobenzene (14.7 mg, 0.10 mmol), alkene **3** (47.4 mg, 0.30 mmol), Ir(Fppy)$_3$ (0.25 mol%) and CH_3CN (0.2 M) was placed in the blue light bath at 60 °C to afford triazoline which was then oxidized by TBHP (4 equiv, 39

μL), KI (20 mol%, 3.3 mg) to obtain **3e** yield (92%) calculated from 1H NMR, (after workup) by adding 4-methyl benzyl chloride as an internal standard.

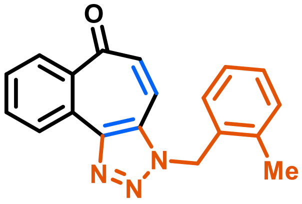

3e”. 3-(2-Methylbenzyl)benzo[3,4]cyclohepta[1,2-d][1,2,3]triazol-6(3H)-one was prepared by **general procedure B**. A mixture of 1-(azidomethyl)-2-methylbenzene (29.4 mg, 0.20 mmol), alkene **3** (87 mg, 0.60 mmol), **Ir(ppy)$_3$** (0.25 mol%) and CH_3CN (0.2 M) to afford triazoline which was then oxidized by TBHP (6 equiv, 116 μL), KI (20 mol%, 6.6 mg) to obtain **3e”** (54.2 mg) in 90% yield as yellow solid after purification by column chromatography using silica (EtOAc/hexanes = 15:85). ^{1}H NMR (400 MHz, Acetone) δ 8.97 (dd, J = 8.1, 1.4 Hz, 1H), 8.34 (dd, J = 8.2, 1.5 Hz, 1H), 7.88 (ddd, J = 8.3, 7.1, 1.5 Hz, 1H), 7.75 – 7.66 (m, 1H), 7.69 (d, J = 12.2 Hz, 1H), 7.30 – 7.20 (m, 2H), 7.15 (td, J = 7.4, 1.8 Hz, 1H), 6.86 (d, J = 7.7 Hz, 1H), 6.81 (d, J = 12.2 Hz, 1H), 6.01 (s, 2H), 2.47 (s, 3H). ^{13}C NMR (201 MHz, Acetone) δ 188.6, 146.4, 137.1, 136.9, 134.6, 134.2, 133.9, 132.1, 131.8, 131.7, 130.6, 130.4, 129.3, 128.0, 127.7, 127.4, 122.3, 51.0, 19.4. Calculated HRMS(ESI) for $C_{19}H_{15}N_3O$ (M+H)+ is 302.1288 observed 302.1292. mp – 186-188 °C.

Ir(Fppy)$_3$ as photocatalyst: A mixture of 1-(azidomethyl)-2-methylbenzene (17.7 mg, 0.10 mmol), alkene **3** (47.4 mg, 0.30 mmol), Ir(Fppy)$_3$ (0.25 mol%) and CH_3CN (0.2 M) was placed in the blue light bath at 60 °C to afford triazoline which was then oxidized by TBHP (6 equiv, 58 μL), KI (20 mol%, 3.3 mg) to obtain **3e”** yield (92%) calculated from 1H NMR, (after workup) by adding 4-methyl benzyl chloride as an internal standard.

This fully aromatic tricyclic motif **(3e”)** appeared to fluoresce under UV light so emission spectrum spectra were recorded on a Fluorolog3 – fluorescence spectrometer (at reaction concentration approximately 0.5 mM in MeCN at 25 °C). Excitation wavelength = 364 nm.

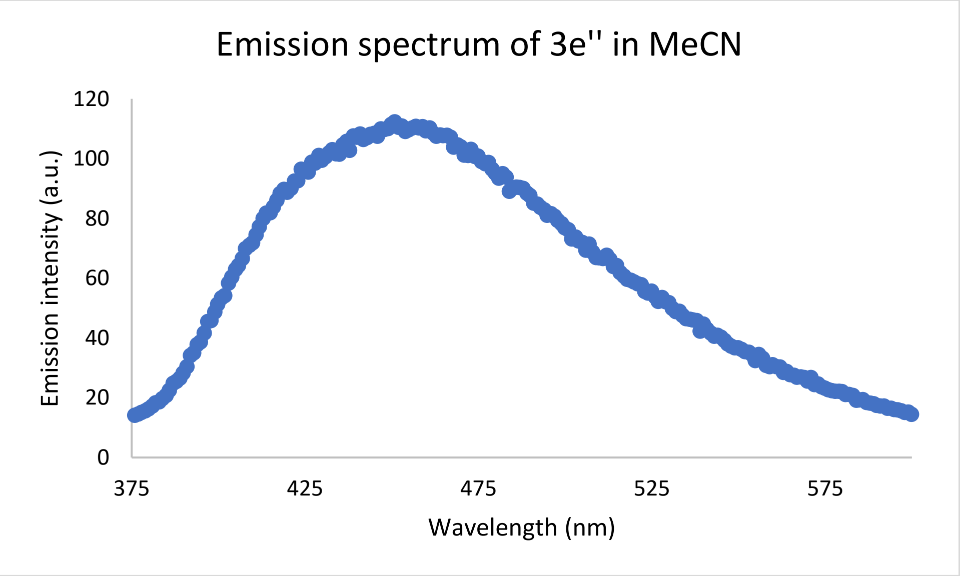

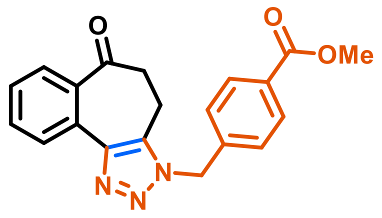

3f. Methyl 4-((6-oxo-5,6-dihydrobenzo[3,4]cyclohepta[1,2-d][1,2,3]triazol-3(4H)-yl)methyl)benzoate was prepared by **general procedure B**. A mixture of 1-(azidomethyl)-2-methylbenzene (59 mg, 0.31 mmol), alkene **3** (146 mg, 0.92 mmol), **Ir(Fppy)₃** (0.25 mol%) and CH_3CN (0.2 M) was placed in the blue light bath at 60 °C to afford triazoline which was then oxidized by TBHP (4 equiv, 124 µL), KI (20 mol%, 10.3 mg) to obtain **3f** (94.3 mg, 0.27 mmol) in 88% yield as dark brown oil after purification by column chromatography using silica (EtOAc/hexanes = 20:80). ^{1}H NMR (800 MHz, $CDCl_3$) δ 8.67 (dd, *J* = 8.1, 1.3 Hz, 1H), 8.04 – 8.02 (m, 2H), 7.79 (dd, *J* = 7.8, 1.4 Hz, 1H), 7.63 – 7.60 (m, 1H), 7.36 (td, *J* = 7.5, 1.2 Hz, 1H), 7.26 – 7.23 (m, 2H), 5.62 (s, 2H), 3.91 (s, 3H), 3.03 – 2.99 (m, 2H), 2.87 – 2.84 (m, 2H). ^{13}C NMR (201 MHz, $CDCl_3$) δ 200.8, 166.4, 144.1, 139.2, 136.3, 133.7, 133.0, 130.8, 130.7, 129.5, 129.3, 128.0, 127.1, 126.6, 52.5, 52.1, 39.0, 19.6. Calculated HRMS(ESI) for $C_{20}H_{17}N_3O_3$ (M+H) + is 348.1343 observed 348.1345.

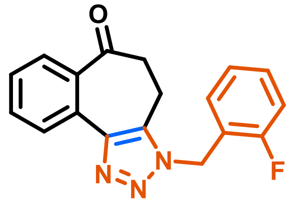

3g. 3-(2-Fluorobenzyl)-4,5-dihydrobenzo[3,4]cyclohepta[1,2-d][1,2,3]triazol-6(3H)-one was prepared by **general procedure B**. A mixture of 1-(azidomethyl)-2-fluorobenzene (30.2 mg, 0.20 mmol), alkene **2** (87 mg, 0.60 mmol), **Ir(ppy)₃** (0.25 mol%) and CH_3CN (0.2 M) was placed in the blue light bath at 65 °C to afford triazoline which was then oxidized by TBHP (4 equiv, 77 µL), KI (20 mol%, 6.6 mg) to obtain **3g** (55.3 mg) in 90% yield as yellow solid after purification by column chromatography using silica (EtOAc/hexanes = 20:80). ^{1}H NMR (400 MHz, CD_3CN) δ 8.56 (dd, *J* = 8.0, 1.3 Hz, 1H), 7.76 (dd, *J* = 7.8, 1.5 Hz, 1H), 7.62 (ddd, *J* = 8.0, 7.3, 1.5 Hz, 1H), 7.42 – 7.35 (m, 2H), 7.21 – 7.14 (m, 3H), 5.60 (d, *J* = 0.9 Hz, 2H), 3.01 (s, 4H). ^{13}C NMR (101 MHz, CD_3CN) δ 201.8, 161.3 (d, *J* = 246.0 Hz), 143.7, 137.1, 135.8, 133.4, 131.5 (d, *J* = 8.4 Hz), 130.8 (d, *J* = 3.3 Hz), 129.9, 128.3, 126.8, 125.8 (d, *J* = 3.6 Hz), 123.2 (d, *J* = 14.5 Hz), 116.5 (d, *J* = 21.2 Hz), 46.5 (d, *J* = 4.6 Hz), 39.7, 19.9 (d, *J* = 1.4 Hz). ^{19}F NMR (376 MHz, $CDCl_3$) δ -118.46 (dt, *J* = 8.7, 5.4 Hz). Calculated HRMS(ESI) for $C_{18}H_{14}FN_3O$ (M+H)+ is 308.1194 observed 308.1196. mp – 117-119 °C.

Ir(Fppy)₃ as photocatalyst: A mixture of 1-(azidomethyl)-4-fluorobenzene (14.7 mg, 0.10 mmol), alkene **2** (47.4 mg, 0.30 mmol), Ir(Fppy)₃ (0.25 mol%) and CH_3CN (0.2 M) was placed in the blue light bath at 60 °C to afford triazoline which was then oxidized by TBHP (4 equiv, 39 µL), KI (20 mol%, 3.3 mg) to obtain **3g** yield (86%) calculated from 1H NMR, (after workup) by adding 4-methyl benzyl chloride as an internal standard.

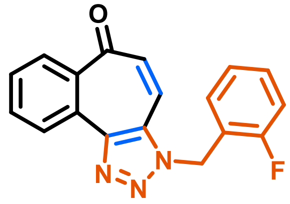

3g". 3-(2-Fluorobenzyl)benzo[3,4]cyclohepta[1,2-d][1,2,3]triazol-6(3H)-one was prepared by **general procedure B**. 1-(azidomethyl)-2-fluorobenzene (30.2 mg, 0.20 mmol), alkene **3** (87 mg, 0.60 mmol), **Ir(ppy)₃** (0.25 mol%) and CH_3CN (0.2 M) was placed in the blue light bath at 65 °C to afford triazoline which was then oxidized by TBHP (6

equiv, 116 μL), KI (20 mol%, 6.6 mg) to obtain **3g”** (56.1 mg) in 92% yield as yellow solid after purification by column chromatography using silica (EtOAc/hexanes = 15:85). ^{1}H NMR (400 MHz, Acetone) δ 8.98 – 8.90 (m, 1H), 8.40 – 8.29 (m, 1H), 7.87 (ddd, *J* = 8.2, 7.1, 1.5 Hz, 1H), 7.80 (d, *J* = 12.2 Hz, 1H), 7.69 (ddd, *J* = 8.4, 7.1, 1.4 Hz, 1H), 7.48 – 7.38 (m, 1H), 7.30 (td, *J* = 7.6, 1.8 Hz, 1H), 7.26 – 7.18 (m, 2H), 6.88 (d, *J* = 12.2 Hz, 1H), 6.04 (s, 2H). ^{13}C NMR (201 MHz, Acetone) δ 188.5, 161.4 (d, *J* = 246.2 Hz), 146.3, 136.9, 134.3, 133.9, 131.9, 131.8, 131.7 (d, *J* = 8.4 Hz), 130.9 (d, *J* = 3.6 Hz), 130.6, 130.3, 127.6, 125.9 (d, *J* = 3.7 Hz), 123.4 (d, *J* = 14.2 Hz), 122.0, 116.6 (d, *J* = 21.3 Hz), 46.8 (d, *J* = 4.4 Hz). ^{19}F NMR (376 MHz, Acetone) δ -118.72 (dt, *J* = 11.6, 6.1 Hz). Calculated HRMS(ESI) for $C_{18}H_{12}FN_3O$ (M+H)+ is 306.1037 observed 306.1042. mp – 134-136 °C.

Ir(Fppy)₃ as photocatalyst: A mixture of 1-(azidomethyl)-2-fluorobenzene (15.1 mg, 0.10 mmol), alkene **3** (47.4 mg, 0.30 mmol), Ir(Fppy)$_3$ (0.25 mol%) and CH_3CN (0.2 M) was placed in the blue light bath at 60 °C to afford triazoline which was then oxidized by TBHP (6 equiv, 58 μL), KI (20 mol%, 3.3 mg) to obtain **3g”** yield (90%) calculated from 1H NMR, (after workup) by adding 4-methyl benzyl chloride as an internal standard.

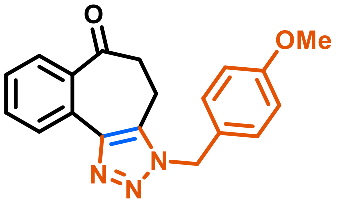

3h. 3-(4-Methoxybenzyl)-4,5-dihydrobenzo[3,4]cyclohepta[1,2-d][1,2,3]triazol-6(3H)-one was prepared by **general procedure B**. A mixture of 1-(azidomethyl)-4-methoxybenzene (32.6 mg, 0.20 mmol), alkene **3** (87 mg, 0.60 mmol), **Ir(ppy)₃** (0.25 mol%) and CH_3CN (0.2 M) was placed in the blue light bath at 65 °C to afford triazoline which was then oxidized by TBHP (4 equiv, 77 μL), KI (20 mol%, 6.6 mg) to obtain **3h** (53.6 mg) in 84% yield as yellow oil after purification by column chromatography using silica (EtOAc/hexanes = 20:80). ^{1}H NMR (800 MHz, $CDCl_3$) δ 8.64 (dd, *J* = 8.0, 1.2 Hz, 1H), 7.76 (dd, *J* = 7.8, 1.5 Hz, 1H), 7.62 – 7.57 (m, 1H), 7.33 (td, *J* = 7.5, 1.3 Hz, 1H), 7.14 (d, *J* = 8.7 Hz, 2H), 6.86 (d, *J* = 8.8 Hz, 2H), 5.49 (s, 2H), 3.78 (s, 3H), 3.04 – 2.96 (m, 2H), 2.92 – 2.84 (m, 2H). ^{13}C NMR (201 MHz, $CDCl_3$) δ 201.1, 159.9, 143.9, 136.2, 133.5, 132.9, 129.7, 129.2, 128.8, 127.7, 126.5, 126.3, 114.7, 55.5, 52.1, 39.1, 19.7. Calculated HRMS(ESI) for $C_{19}H_{17}N_3O_2$ (M+H)+ is 320.1394 observed 320.1400.

Ir(Fppy)₃ as photocatalyst: A mixture of 1-(azidomethyl)-4-methoxybenzene (16.3 mg, 0.10 mmol), alkene **3** (47.4 mg, 0.30 mmol), Ir(Fppy)$_3$ (0.25 mol%) and CH_3CN (0.2 M) was placed in the blue light bath at 60 °C to afford triazoline which was then oxidized by TBHP (4 equiv, 39 μL), KI (20 mol%, 3.3 mg) to obtain **3h** yield (90%) calculated from 1H NMR, (after workup) by adding 4-methyl benzyl chloride as an internal standard.

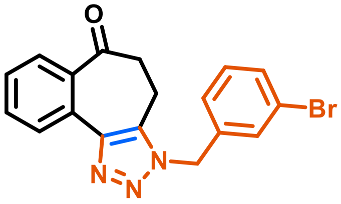

3i. 3-(3-Bromobenzyl)-4,5-dihydrobenzo[3,4]cyclohepta[1,2-d][1,2,3]triazol-6(3H)-one was prepared by **general procedure B**. A mixture of 1-(azidomethyl)-3-bromobenzene (50 mg, 0.24 mmol), alkene **3** (112 mg, 0.72 mmol), **Ir(Fppy)₃** (0.25 mol%) and CH_3CN (0.2 M) was placed in the blue light bath at 60 °C to afford triazoline

which was then oxidized by TBHP (4 equiv, 96 μL), KI (20 mol%, 8 mg) to obtain **3i** (74.8 mg) in 86% yield as yellow oil after purification by column chromatography using silica (EtOAc/hexanes = 20:80). ^{1}H NMR (400 MHz, $CDCl_3$) δ 8.66 (dd, *J* = 8.1, 1.2 Hz, 1H), 7.79 (dd, *J* = 7.8, 1.5 Hz, 1H), 7.61 (ddd, *J* = 8.0, 7.3, 1.5 Hz, 1H), 7.47 (ddd, *J* = 8.1, 2.0, 1.0 Hz, 1H), 7.41 – 7.32 (m, 2H), 7.23 (t, *J* = 7.8 Hz, 1H), 7.10 (ddd, *J* = 7.7, 1.8, 1.0 Hz, 1H), 5.53 (s, 2H), 3.07 – 2.99 (m, 2H), 2.92 – 2.84 (m, 2H). ^{13}C NMR (201 MHz, $CDCl_3$) δ 200.8, 144.0, 136.6, 136.2, 133.6, 132.9, 132.0, 130.9, 130.3, 129.5, 129.3, 127.9, 126.6, 125.8, 123.4, 51.6, 39.0, 19.6. Calculated HRMS(ESI) for $C_{18}H_{14}BrN_3O$ (M+H)+ is 368.0393 observed 368.0405.

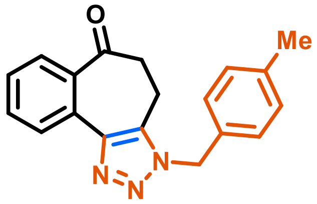

3j. 3-(4-Methylbenzyl)-4,5-dihydrobenzo[3,4]cyclohepta[1,2-d][1,2,3]triazol-6(3H)-one was prepared by **general procedure B**. A mixture of 1-(azidomethyl)-2-methylbenzene (29.4 mg, 0.20 mmol), alkene **3** (87 mg, 0.60 mmol), **Ir(ppy)$_3$** (0.25 mol%) and CH_3CN (0.2 M) to afford triazoline which was then oxidized by TBHP (4 equiv, 77 μL), KI (20 mol%, 6.6 mg) to obtain **3j** (53.3 mg) in 88% yield as yellow solid after purification by column chromatography using silica (EtOAc/hexanes = 20:80). ^{1}H NMR (400 MHz, $CDCl_3$) δ 8.65 (dd, *J* = 8.0, 1.2 Hz, 1H), 7.77 (dd, *J* = 7.8, 1.4 Hz, 1H), 7.60 (ddd, *J* = 8.0, 7.3, 1.5 Hz, 1H), 7.34 (td, *J* = 7.6, 1.3 Hz, 1H), 7.15 (d, *J* = 7.9 Hz, 2H), 7.08 (d, *J* = 8.0 Hz, 2H), 5.52 (s, 2H), 3.08 – 2.95 (m, 2H), 2.93 – 2.81 (m, 2H), 2.33 (s, 3H). ^{13}C NMR (101 MHz, $CDCl_3$) δ 201.1, 143.8, 138.7, 136.2, 133.6, 132.9, 131.3, 130.0, 129.7, 129.2, 127.7, 127.2, 126.5, 52.4, 39.1, 21.2, 19.7. Calculated HRMS(ESI) for $C_{19}H_{17}N_3O$ (M+H)+ is 304.1444 observed 304.1446. mp – 100-102 ^{o}C.

Ir(Fppy)$_3$ as photocatalyst: A mixture of 1-(azidomethyl)-2-methylbenzene (17.7 mg, 0.10 mmol), alkene 3 (47.4 mg, 0.30 mmol), Ir(Fppy)$_3$ (0.25 mol%) and CH_3CN (0.2 M) was placed in the blue light bath at 60 ^{o}C to afford triazoline which was then oxidized by TBHP (4 equiv, 39 μL), KI (20 mol%, 3.3 mg) to obtain **3j** yield (72%) calculated from 1H NMR, (after workup) by adding 4-methyl benzyl chloride as an internal standard.

Determination of the correct regioisomer.

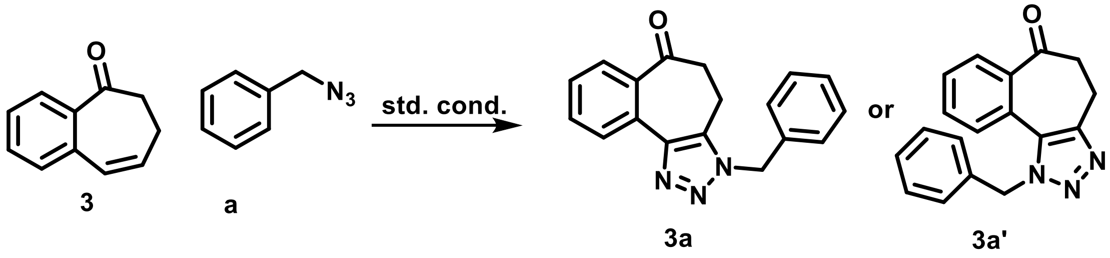

The photocatalytic cycloaddition with oxo-BC7 (3) yielded a single regioisomer. To identify the correct regioisomer, we conducted spectroscopic experiments (on triazole), including COSY and NOESY. We initially assigned all the aromatic protons using COSY, followed by NOESY to assess the spatial proximity of the 1H-1H protons.

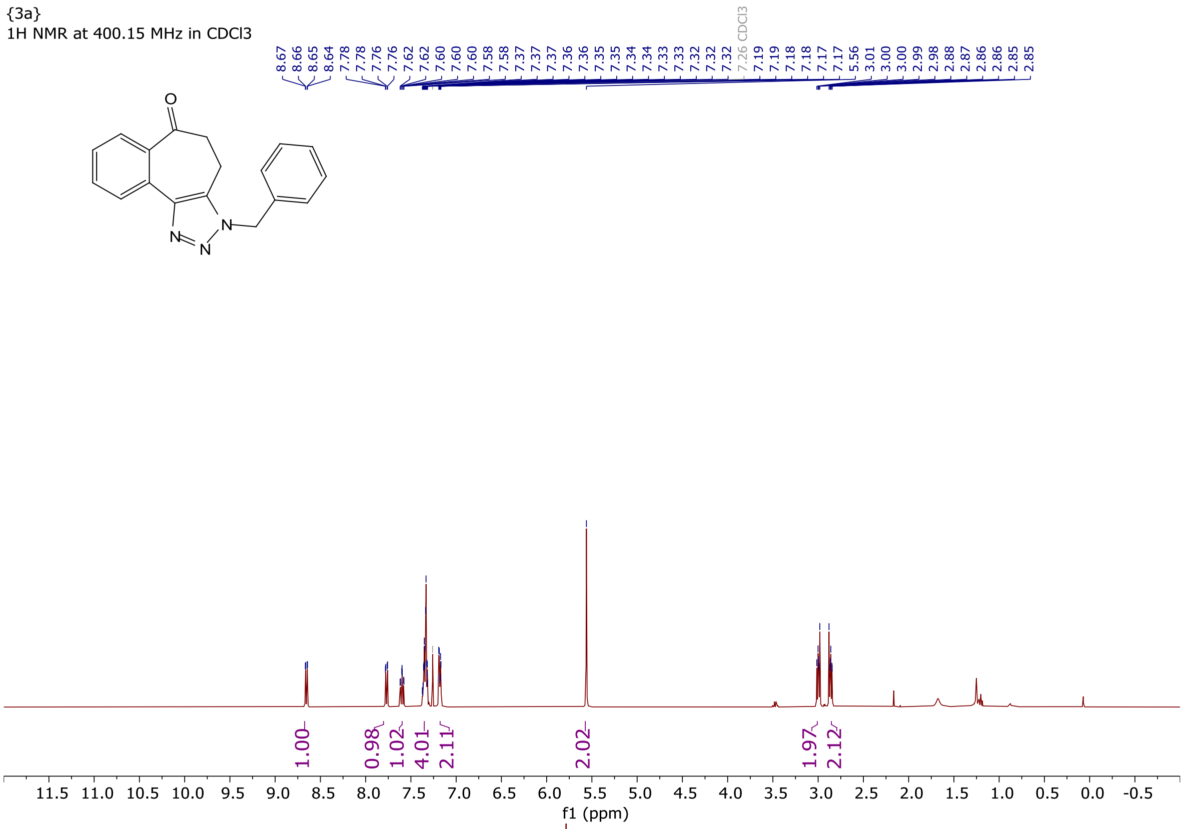
{3a}
1H NMR at 400.15 MHz in CDCl3
8.67 8.66 8.65 8.64 7.78 7.78 7.76 7.76 7.62 7.62 7.60 7.60 7.58 7.58 7.37 7.37 7.36 7.36 7.35 7.35 7.34 7.34 7.33 7.33 7.32 7.32 7.26 CDCl3 7.19 7.19 7.18 7.18 7.17 7.17 5.56 3.01 3.00 2.99 2.98 2.88 2.87 2.86 2.85 2.85
1.00
0.98
1.02
4.01
2.11
2.02
1.97
2.12
f1 (ppm)

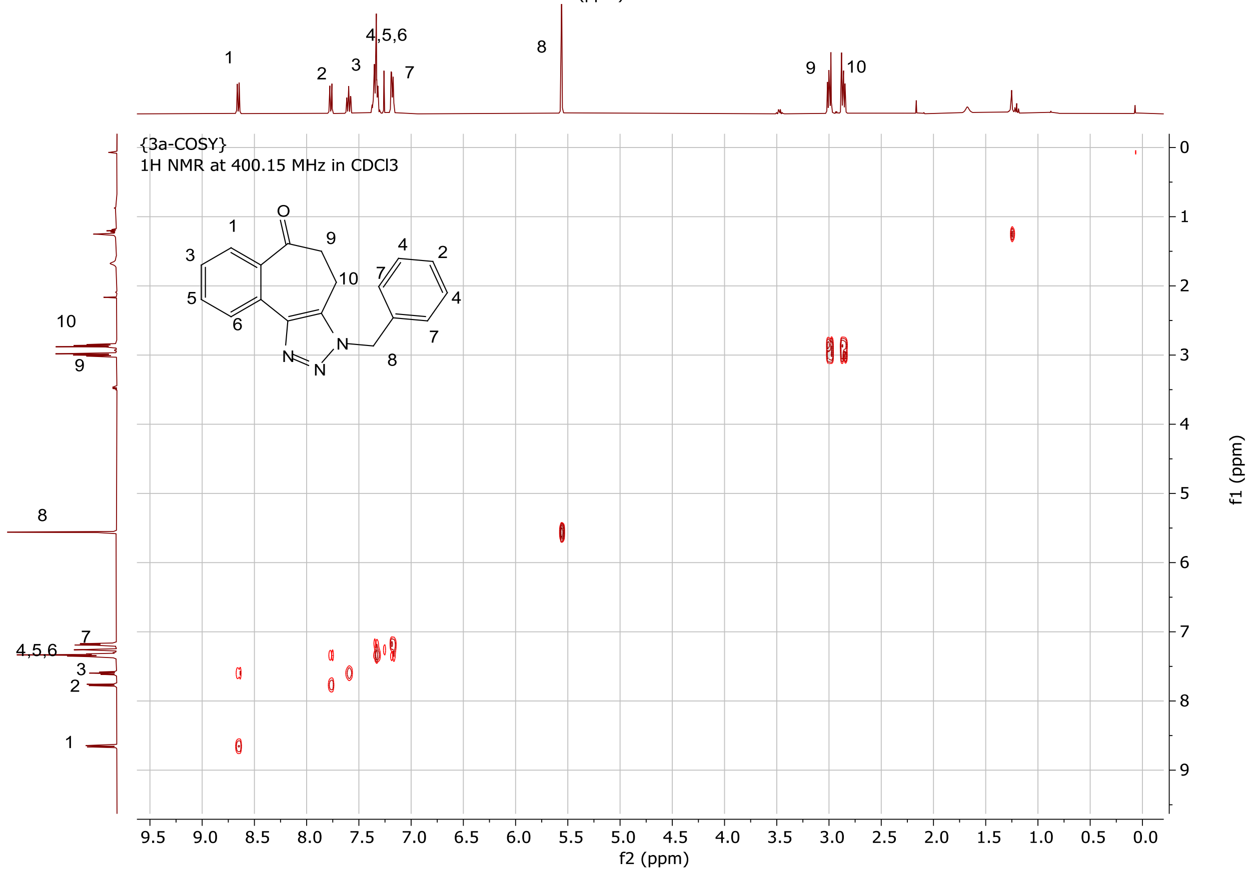
{3a-COSY}
1H NMR at 400.15 MHz in CDCl3
1
2
3
4,5,6
7
8
9
10
f2 (ppm)
f1 (ppm)

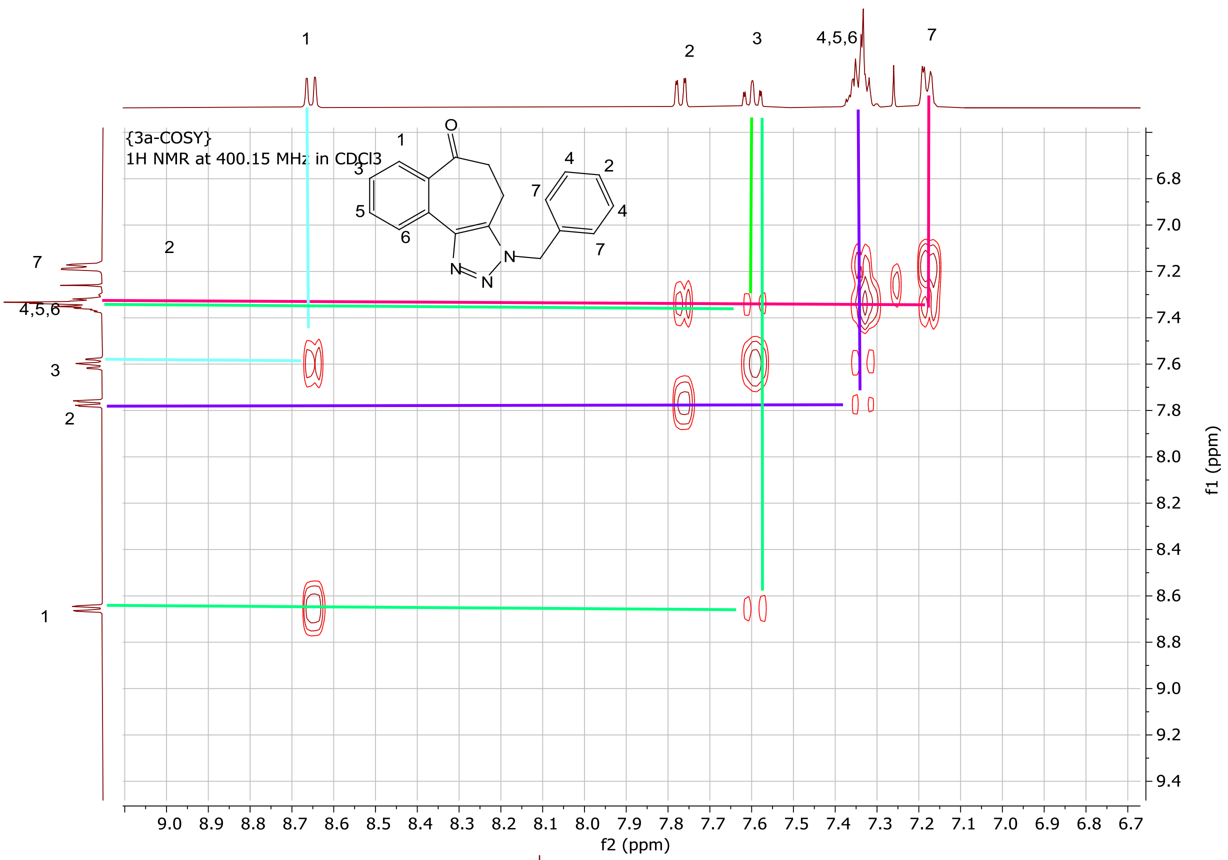

{3a-COSY}
1H NMR at 400.15 MHz in CDCl3
1
2
3
4,5,6
7
f2 (ppm)
f1 (ppm)

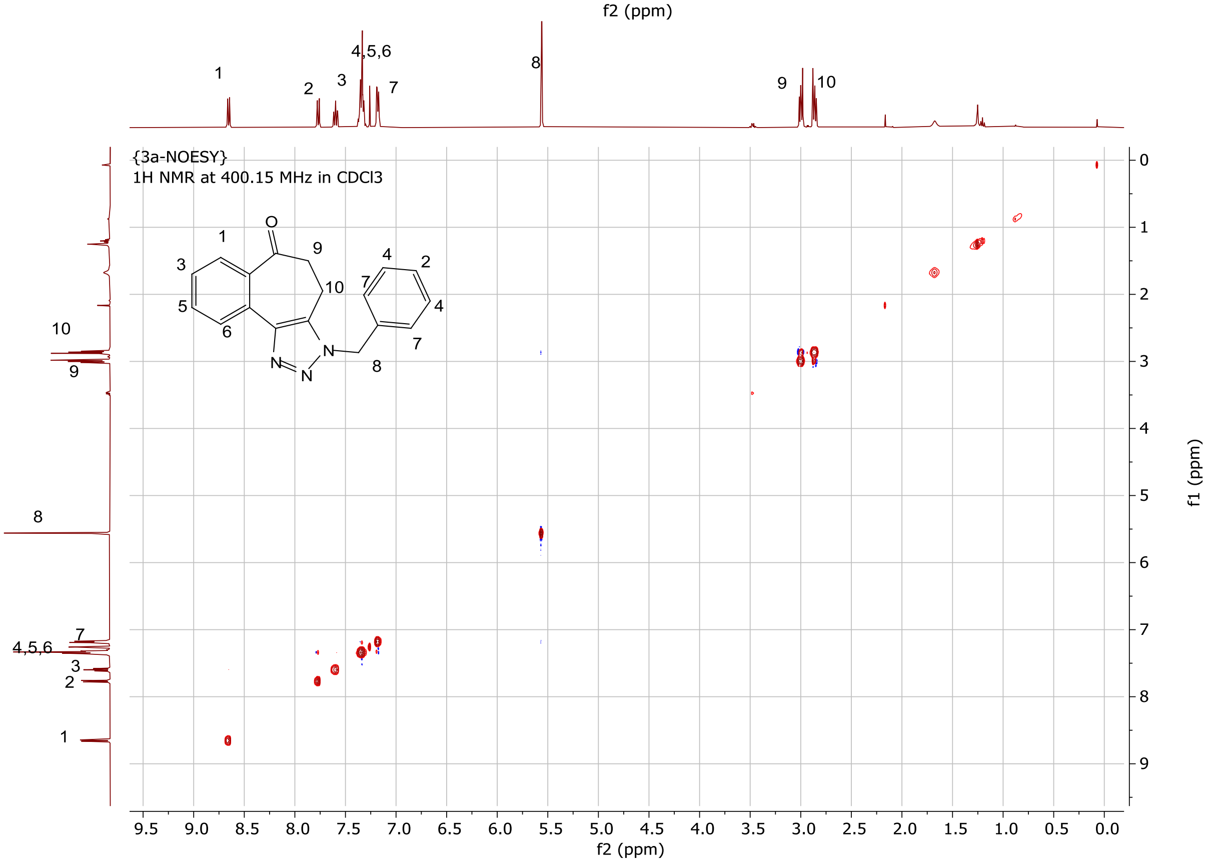

{3a-NOESY}
1H NMR at 400.15 MHz in CDCl3
1
2
3
4,5,6
7
8
9
10
f2 (ppm)
f1 (ppm)

NOESY revealed a correlation between the protons at positions 8 and 10, while no correlation was found between the protons at positions 8 and 6. This confirms that **3a** is the regioisomer obtained under our standard conditions.

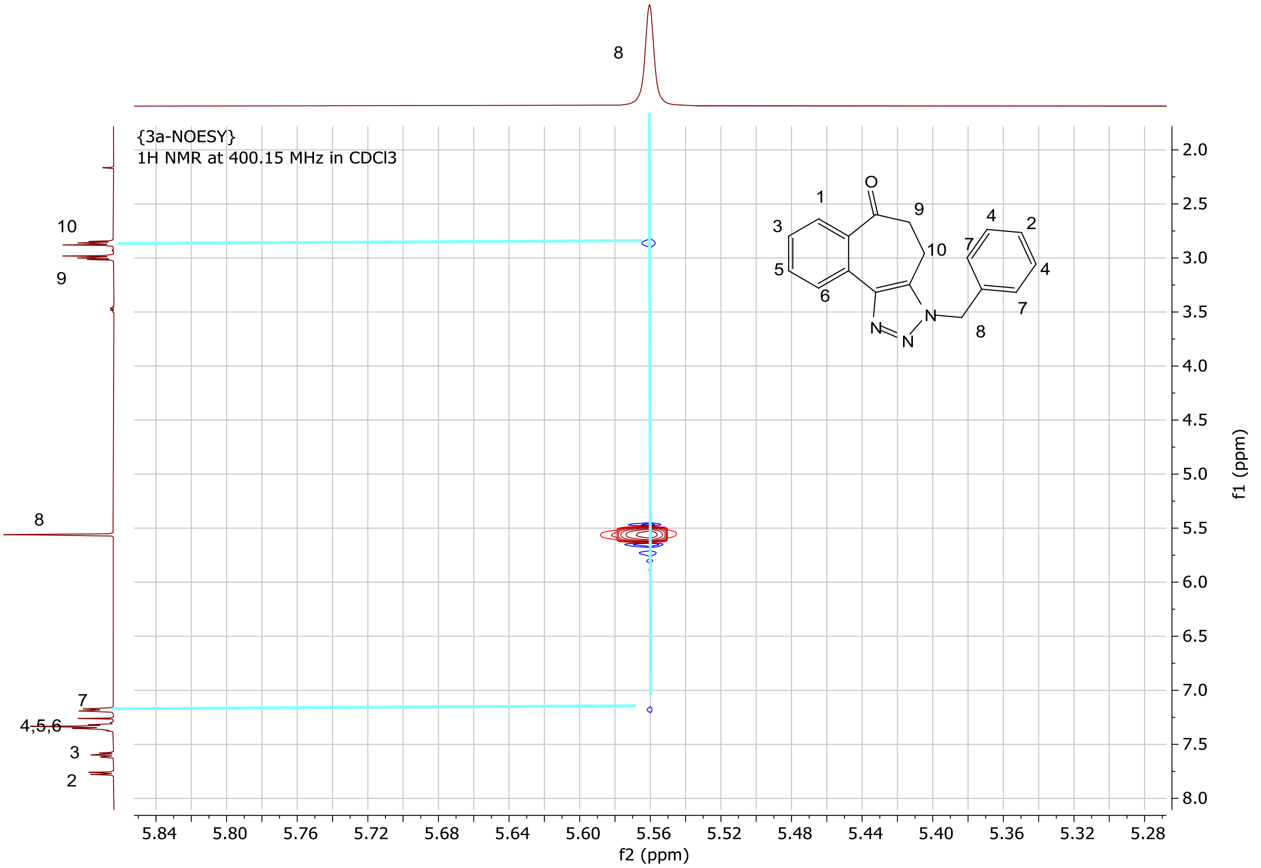

Determination of cis vs trans-ring fusion

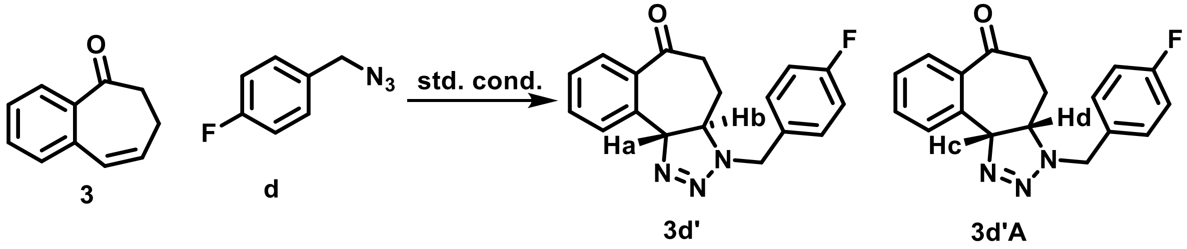

The photocatalytic cycloaddition with **oxo-BC7** (**3**) produced a single regioisomer unlike **BC7** reported earlier by our group to produce both regioisomers.[2] In order to probe the diastereoselectivity of the ring fusion, we performed the similar assessment as reported earlier by our group.[2] The trans-ring fusion was established based on the coupling constants of the methine protons on the ring junction in the crude ^{1}HNMR (shown below). The methine protons were assigned by COSY NMR. The coupling constant between the ring methine protons was found to be 15.9 Hz- suggestive of *trans* geometry between **Ha** and **Hb** protons. We performed an energy

minimization using simple MM2 calculations of **3d’** (*trans*) which indicated a dihedral angle between **Ha** and **Hb** to be 165°. Whereas, when the structure of the *cis*-diastereomer **3d’A** was minimized, the dihedral angle between **Hc** and **Hd** was found to be 37°. According to Karplus curve for ethane derivatives,[4] the magnitude of the coupling constant observed (15.9 Hz) is consistent with a large dihedral angle (i.e., 165°) of **3d’**. Furthermore, the small dihedral angle calculated for **3d’A** would be expected to give a very small coupling constant. Thus, the coupling constants observed by 1H NMR, and the dihedral angle corresponds to **3d’**, that is the *trans*-ring fusion for the cycloaddition of alkene **3** (**oxo-BC7**), which is expected if the geometry of the reactive double bond is *trans* and the cycloaddition is not a step-wise process.

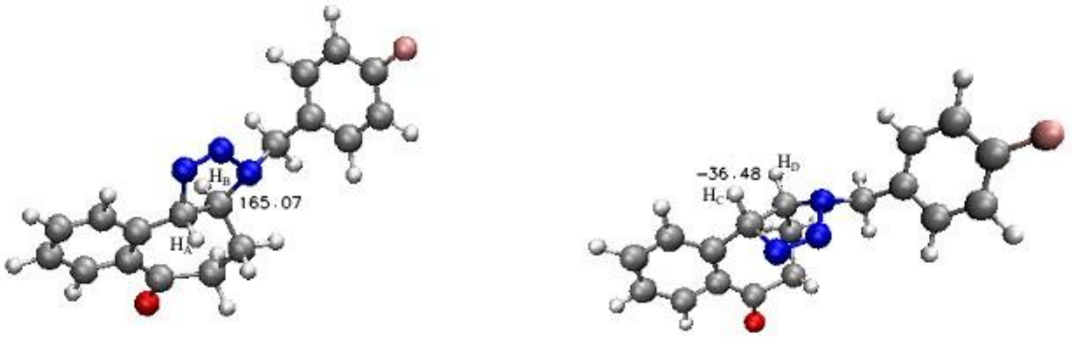

3d **3d’A**

Figure S1. Minimized energy structure for the trans (**3d’**) and cis (**3d’A**) ring fusion.

3d' crude NMR at 400.15 MHz in C6D6

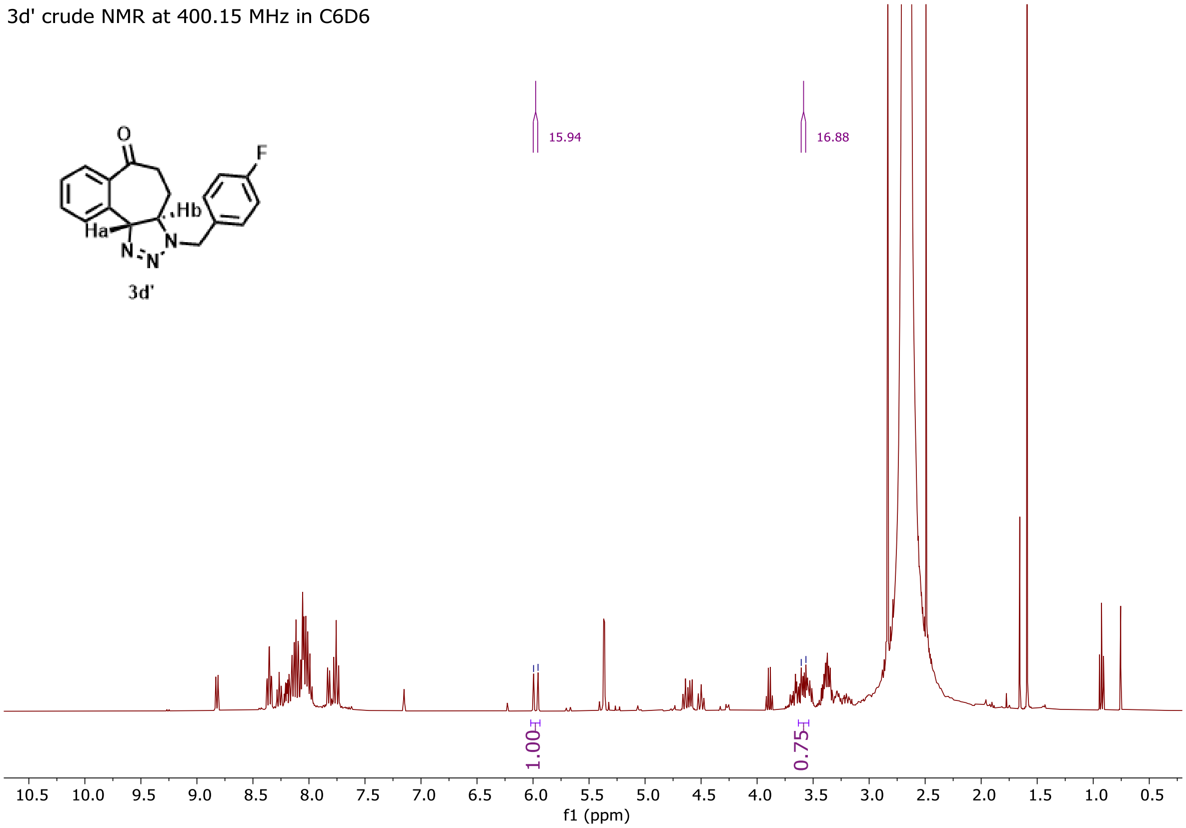

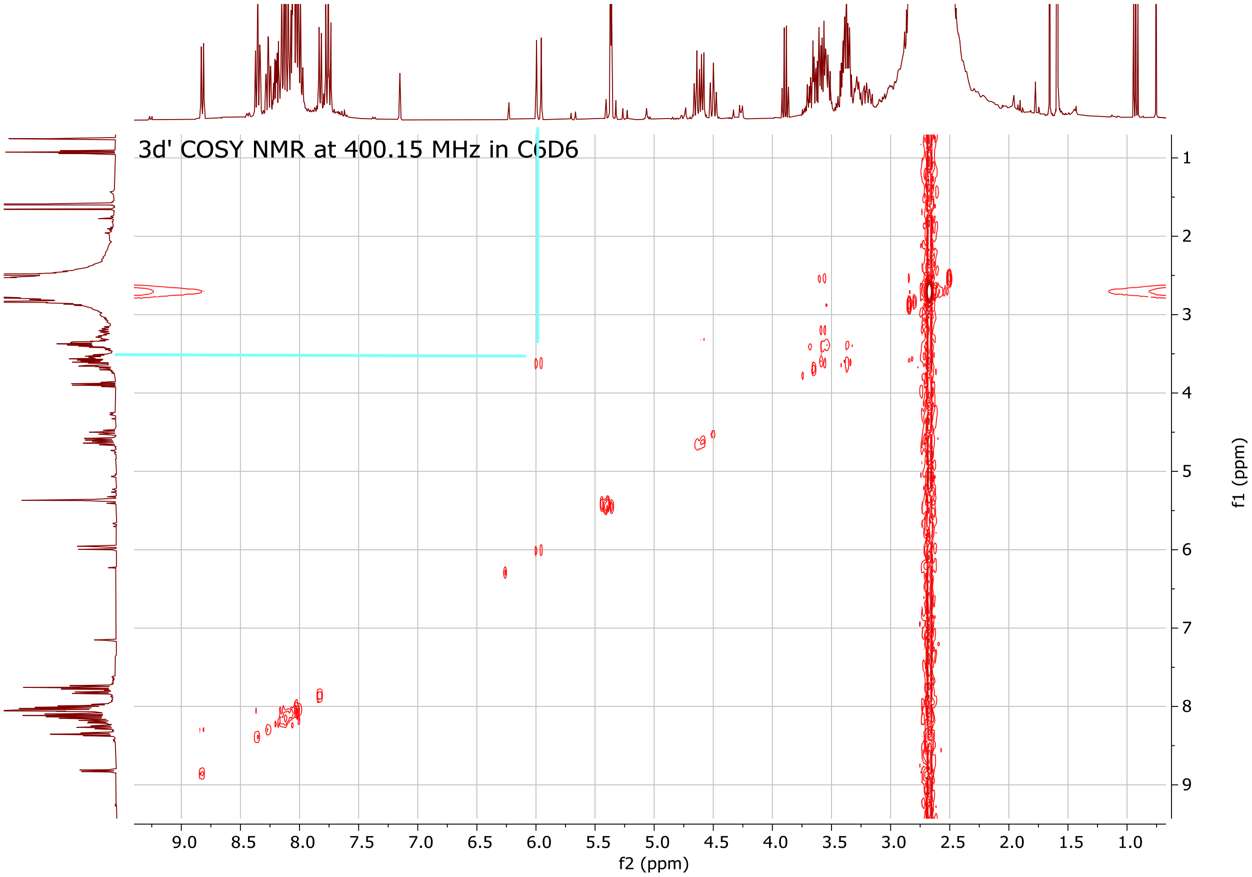

Synthesis of alkene 5 (Br-oxo-BC7).

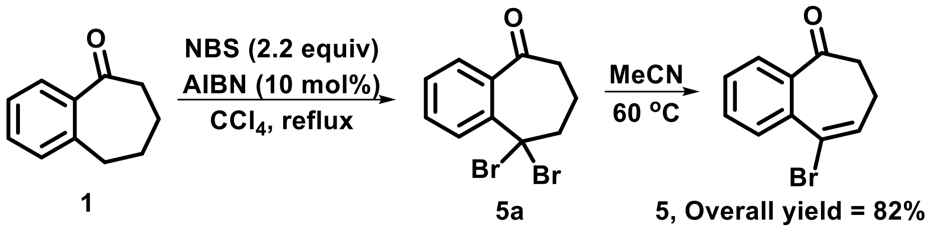

Alkene **5** was synthesized by the reported procedure.[3]

Adsorption spectrum of alkene 5 (Br-oxo-BC7).

Absorption spectrum was recorded on a Shimadzu UV-2600 UV-vis spectrometer at reaction concentration (approximately 0.5 mM) in MeCN at 25 °C.

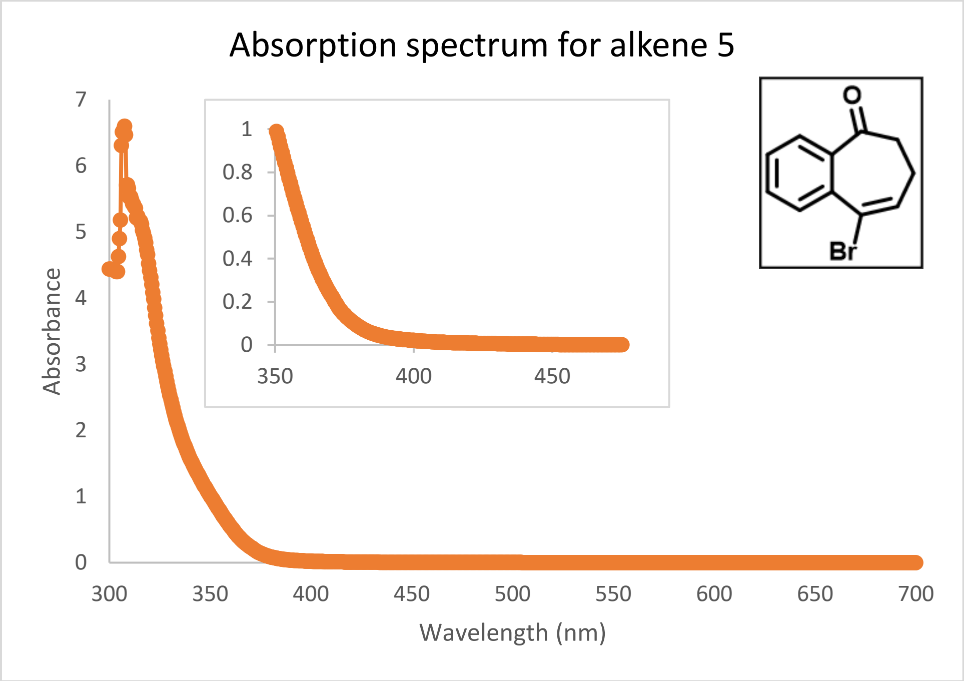

Optimization of the catalyst free, light driven cycloaddition with Br-oxo-BC7.

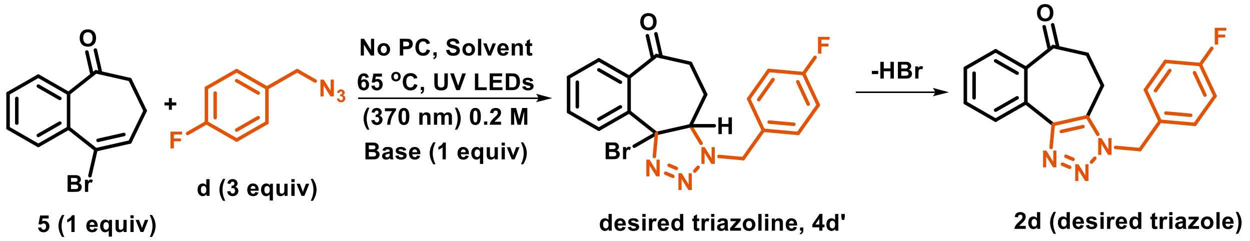

Unlike previous reactions, this reaction produced HBr in the reaction mixture, which was found to be detrimental. So, a campaign to evaluate bases and solvents was performed.

Base Screening

Entry	Base[a]	NMR conv.
1	Piperidine	0%[c]
2	Pyrrolidine	0%[c]
3	2,6 di-tertbutylpyridine	44%[d]
4	DBU	0%[c]
5	Triethyl amine	56%[d]
6	2,4,6-trimethylpyridine	79%[d]
7	DMAP	8%[c]
8	1,1,3,3 tetramethylguanidine	0%[c]
9	3,5-dimethylpyridine	45%[d]

Solvent Screening

Entry	Solvent[b]	NMR conv.
10	MeCN	79%[d]
11	EtOAc	25%[c]
12	DMF	80%[d]
13	DMSO	88%[c]
14	Acetone	76%[d]
15	1,4 dioxane	32%[c]
16	Toluene	17%[c]
17	MeOH	20%[c]

SI table 2. [a]Solvent employed for base screening is MeCN. [b]2,4,6-trimethylpyridine is used as a base for solvent screening. [c]Conversion based on ^{19}F NMR. [d]Conversion based on ^{19}F NMR post workup.

An initial screening of bases (entries 1-9, SI table 2) in MeCN solvent, revealed that 2,4,6-trimethyl pyridine gave the best yield of the bases evaluated. We then turned to an evaluation of the solvent (entries 10-17), in which we determined that DMSO provided the best results (88% NMR yield). At this point, the reaction mixture was heterogenous, so we wanted to study the effect of water on the reaction.

Water Equivalents Screening

A series of reactions were set up to evaluate the effect of water on the yield for the light driven cycloaddition of alkene **5** with azide **d**.

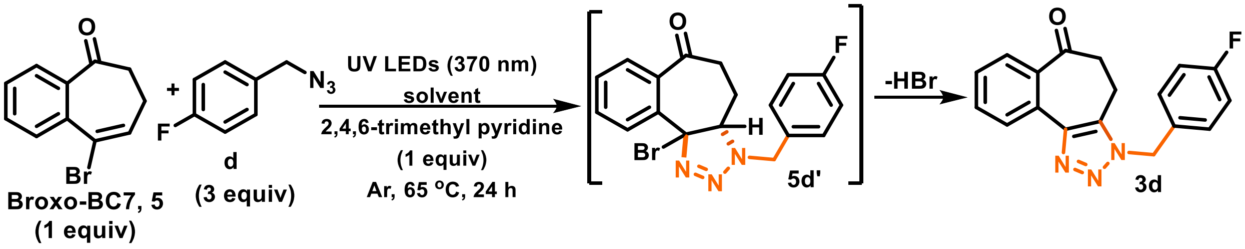

4 NMR tubes were each charged with **5** (23.6 mg, 0.1 mmol), 1-(azidomethyl)-4-fluorobenzene (45.3 mg, 0.3 mmol), 2,4,6-trimethyl pyridine (1 equiv) and water- ranging from 0.1-0.4 mL and DMSO such that the total volume = 0.5 mL. The mixtures were capped with a septum and degassed by argon sparging as described above. Then each reaction was placed in the UV light bath (370 nm) at 65 °C to afford the triazole **3d**. The reactions were irradiated for 24 hours followed by an EtOAc-water workup as mentioned above (**General procedure C**). Product **3d** yields are

calculated from 1H NMR, (homogeneous after workup) by adding 4-methyl benzyl chloride (13.2 μL, 0.1 mmol), as an internal standard.

S. No.	DMSO (mL)	DI water (mL)	NMR yield (^{1}H NMR)
**1**	0.4	0.1	71%
**2**	0.3	0.2	55%
**3**	0.2	0.3	97%
**4**	0.1	0.4	60%

Before irradiation:

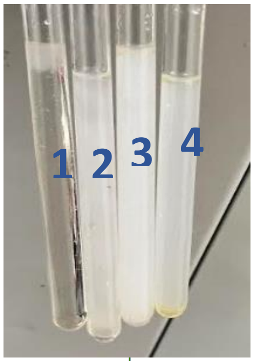

After irradiation:

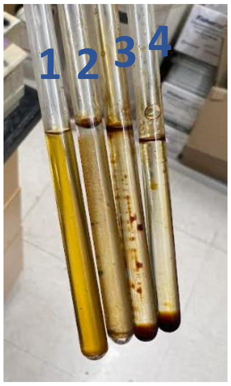

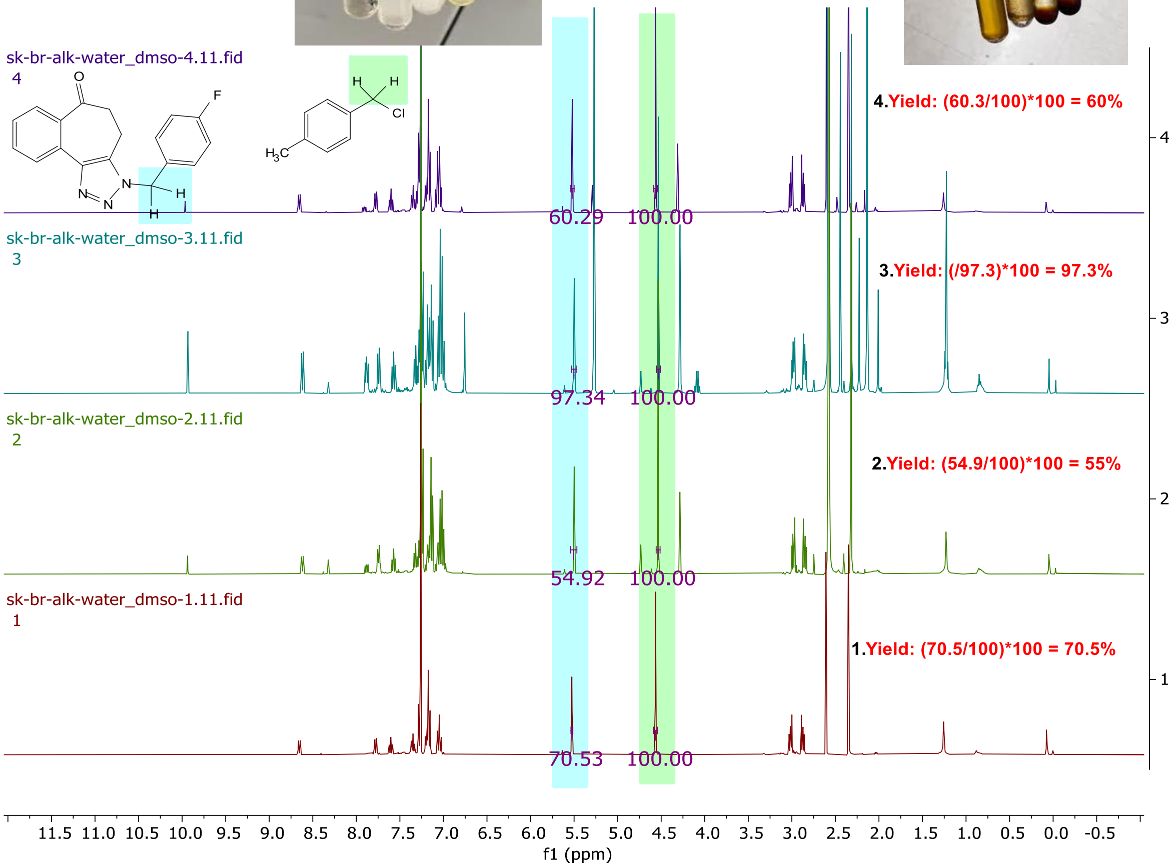

At 65 ^{o}C, DMSO:water at 2:3 seems to be the optimized solvent ratio for this reaction.

The photocatalytic cycloaddition with Br-oxo-BC7.

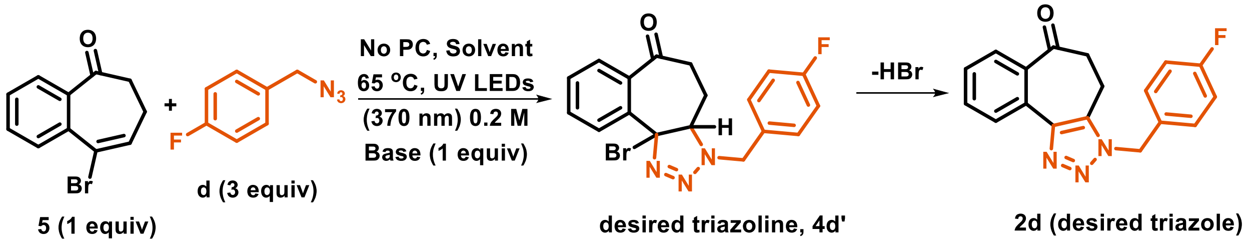

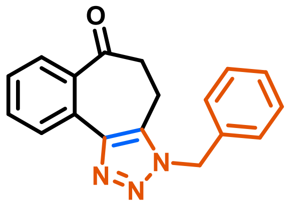

3a. 3-Benzyl-4,5-dihydrobenzo[3,4]cyclohepta[1,2-d][1,2,3]triazol-6(3H)-one was prepared by **general procedure C**. A mixture of benzyl azide (79.8 mg, 0.60 mmol), alkene **5** (47.2 mg, 0.20 mmol), 2,4,6-trimethyl pyridine (27 μL, 0.20 mmol) and DMSO:water (2:3) (0.2 M) was placed in the UV light bath (370 nm) at 65 °C to afford triazole **3a** (44 mg) in 76% yield after purification by column chromatography using silica (EtOAc/hexanes = 20:80).

For 1 equiv. of azide: A mixture of benzyl azide (13.3 mg, 0.10 mmol), alkene **5** (23.6 mg, 0.10 mmol), 2,4,6-trimethyl pyridine (13.5 μL, 0.10 mmol) and DMSO:water (2:3) (0.2 M) was placed in the UV light bath (370 nm) at 65 °C to afford triazole **3a** in 70% yield calculated from 1H NMR, (after workup) by adding 4-methyl benzyl chloride as an internal standard.

At 395 nm: A mixture of benzyl azide (79.8 mg, 0.60 mmol), alkene **5** (47.2 mg, 0.20 mmol), 2,4,6-trimethyl pyridine (27 μL, 0.20 mmol) and DMSO:water (2:3) (0.2 M) was placed in the 395 nm light bath at 60 °C for 72 h to afford triazole **3a** in 96% yield calculated from 1H NMR, (after workup) by adding 4-methyl benzyl chloride as an internal standard.

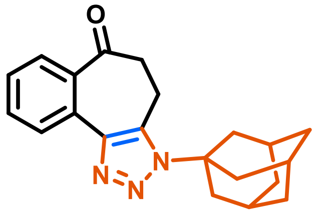

3b. 3-((3s,5s,7s)-Adamantan-1-yl)-4,5-dihydrobenzo[3,4]cyclohepta[1,2-d][1,2,3]triazol-6(3H)-one was prepared by **general procedure C**. A mixture of 1-azidoadamantane (106.2 mg, 0.60 mmol), alkene **5** (47.2 mg, 0.20 mmol), 2,4,6 trimethyl pyridine (27 μL, 0.20 mmol) and DMSO:water (2:3) (0.2 M) was placed in the UV light bath (370 nm) at 65 °C to afford triazole **3b** (46 mg) in 69% yield as yellow oil after purification by column chromatography using silica (EtOAc/hexanes = 20:80). ^{1}H NMR (800 MHz, $CDCl_3$) δ 8.63 (dd, J = 8.1, 1.2 Hz, 1H), 7.88 (dd, J = 7.8, 1.4 Hz, 1H), 7.59 (ddd, J = 8.2, 7.2, 1.5 Hz, 1H), 7.34 (td, J = 7.5, 1.3 Hz, 1H), 3.35 – 3.30 (m, 2H), 3.10 – 3.05 (m, 2H), 2.39 (d, J = 3.1 Hz, 7H), 2.32 – 2.26 (m, 3H), 1.85 – 1.78 (m, 6H). ^{13}C NMR (201 MHz, $CDCl_3$) δ 200.8, 144.6, 135.0, 133.9, 133.1, 130.9, 129.3, 127.5, 127.2, 62.4, 41.9, 40.5, 36.0, 29.8, 21.6.

For 1 equiv. of azide: A mixture of 1-azidoadamantane (17.7 mg, 0.10 mmol), alkene **3** (23.6 mg, 0.10 mmol), 2,4,6-trimethyl pyridine (13.5 μL, 0.10 mmol) and DMSO:water (2:3) (0.2 M) was

placed in the UV light bath (370 nm) at 65 °C to afford triazole **3b** in 89% yield calculated from 1H NMR, (after workup) by adding 4-methyl benzyl chloride as an internal standard.
At 395 nm: A mixture of 1-azidoadamantane (106.2 mg, 0.60 mmol), alkene **5** (47.2 mg, 0.20 mmol), 2,4,6 trimethyl pyridine (27 µL, 0.20 mmol) and DMSO:water (2:3) (0.2 M) was placed in the 395 nm light bath at 60 °C for 72 h to afford triazole **3b** in 94% yield calculated from 1H NMR, (after workup) by adding 4-methyl benzyl chloride as an internal standard.

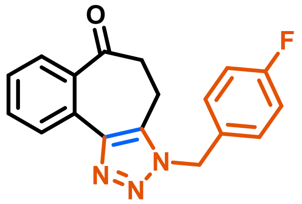

3d. 3-(4-Fluorobenzyl)-4,5-dihydrobenzo[3,4]cyclohepta[1,2-d][1,2,3]triazol-6(3H)-one was prepared by **general procedure C**. A mixture of 1-(azidomethyl)-4-fluorobenzene (90.6 mg, 0.60 mmol), alkene **5** (47.2 mg, 0.20 mmol), 2,4,6 trimethyl pyridine (27 µL, 0.20 mmol) and DMSO:water (2:3) (0.2 M) was placed in the UV light bath (370 nm) at 65 °C to afford triazole **3d** (55.3 mg) in 90% yield after purification by column chromatography using silica (EtOAc/hexanes = 20:80).
For 1 equiv. of azide: A mixture of 1-(azidomethyl)-4-fluorobenzene (15.1 mg, 0.10 mmol), alkene **5** (23.6 mg, 0.10 mmol), 2,4,6 trimethyl pyridine (13.5 µL, 0.10 mmol) and DMSO:water (2:3) (0.2 M) was placed in the UV light bath (370 nm) at 65 °C to afford triazole **3d** in 70% yield calculated from 1H NMR, (after workup) by adding 4-methyl benzyl chloride as an internal standard.

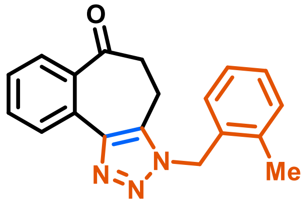

3e. 3-(2-Methylbenzyl)-4,5-dihydrobenzo[3,4]cyclohepta[1,2-d][1,2,3]triazol-6(3H)-one was prepared by **general procedure C**. A mixture of 1-(azidomethyl)-2-methylbenzene (88.2 mg, 0.60 mmol), alkene **5** (47.2 mg, 0.20 mmol), 2,4,6 trimethyl pyridine (27 µL, 0.20 mmol) and DMSO:water (2:3) (0.2 M) was placed in the UV light bath (370 nm) at 65 °C to afford triazole **3e** (49 mg) in 81% yield after purification by column chromatography using silica (EtOAc/hexanes = 20:80).
For 1 equiv. of azide: A mixture of 1-(azidomethyl)-2-methylbenzene (14.7 mg, 0.10 mmol), alkene **5** (23.6 mg, 0.10 mmol), 2,4,6 trimethyl pyridine (13.5 µL, 0.10 mmol) and DMSO:water (2:3) (0.2 M) was placed in the UV light bath (370 nm) at 65 °C to afford triazole **3e** in 87% yield calculated from 1H NMR, (after workup) by adding 4-methyl benzyl chloride as an internal standard.

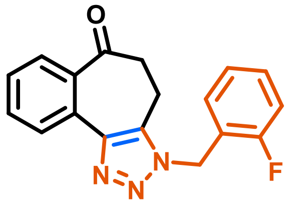

3g. 3-(2-Fluorobenzyl)-4,5-dihydrobenzo[3,4]cyclohepta[1,2-d][1,2,3]triazol-6(3H)-one was prepared by **general procedure C**. A mixture of 1-(azidomethyl)-2-fluorobenzene (90.6 mg, 0.60 mmol), alkene **5** (47.2 mg, 0.20 mmol), 2,4,6-trimethyl pyridine (27 µL, 0.20 mmol) and DMSO:water (2:3) (0.2 M) was placed in the UV light bath

(370 nm) at 65 ^{o}C to afford triazole **3g** (48 mg) in 78% yield after purification by column chromatography using silica (EtOAc/hexanes = 20:80).

For 1 equiv. of azide: A mixture of 1-(azidomethyl)-2-fluorobenzene (15.1 mg, 0.10 mmol), alkene **5** (23.6 mg, 0.10 mmol), 2,4,6-trimethyl pyridine (13.5 µL, 0.10 mmol) and DMSO:water (2:3) (0.2 M) was placed in the UV light bath (370 nm) at 65 ^{o}C to afford triazole **3g** in 77% yield calculated from 1H NMR, (after workup) by adding 4-methyl benzyl chloride as an internal standard.

At 395 nm: A mixture of 1-(azidomethyl)-2-fluorobenzene (90.6 mg, 0.60 mmol), alkene **5** (47.2 mg, 0.20 mmol), 2,4,6-trimethyl pyridine (27 µL, 0.20 mmol) and DMSO:water (2:3) (0.2 M) was placed in the 395 nm light bath at 60 ^{o}C for 72 h to afford triazole **3g** in 95% yield calculated from 1H NMR, (after workup) by adding 4-methyl benzyl chloride as an internal standard.

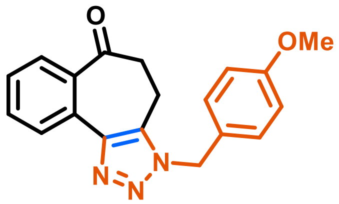

3h. 3-(4-Methoxybenzyl)-4,5-dihydrobenzo[3,4]cyclohepta[1,2-d][1,2,3]triazol-6(3H)-one was prepared by **general procedure C**. A mixture of 1-(azidomethyl)-4-methoxybenzene (97.8 mg, 0.60 mmol), alkene **5** (47.2 mg, 0.20 mmol), 2,4,6 trimethyl pyridine (27 µL, 0.20 mmol) and DMSO:water (2:3) (0.2 M) was placed in the UV light bath (370 nm) at 65 ^{o}C to afford triazole **3h** (36.4 mg) in 57% yield after purification by column chromatography using silica (EtOAc/hexanes = 20:80).

For 1 equiv. of azide: A mixture of 1-(azidomethyl)-4-methoxybenzene (16.3 mg, 0.10 mmol), alkene **5** (23.6 mg, 0.10 mmol), 2,4,6-trimethyl pyridine (13.5 µL, 0.10 mmol) and DMSO:water (2:3) (0.2 M) was placed in the UV light bath (370 nm) at 65 ^{o}C to afford triazole **3h** in 68% yield calculated from 1H NMR, (after workup) by adding 4-methyl benzyl chloride as an internal standard.

At 395 nm: A mixture of 1-(azidomethyl)-4-methoxybenzene (97.8 mg, 0.60 mmol), alkene **5** (47.2 mg, 0.20 mmol), 2,4,6 trimethyl pyridine (27 µL, 0.20 mmol) and DMSO:water (2:3) (0.2 M) was placed in the 395 nm light bath at 60 ^{o}C for 72 h to afford triazole **3h** in 87% yield calculated from 1H NMR, (after workup) by adding 4-methyl benzyl chloride as an internal standard.

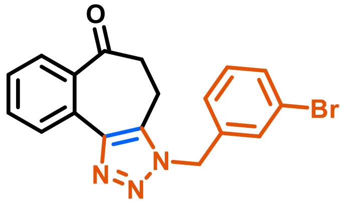

3i. 3-(3-Bromobenzyl)-4,5-dihydrobenzo[3,4]cyclohepta[1,2-d][1,2,3]triazol-6(3H)-one was prepared by **general procedure C**. A mixture of 1-(azidomethyl)-3-bromobenzene (126.6 mg, 0.60 mmol), alkene **5** (47.2 mg, 0.20 mmol), 2,4,6-trimethyl pyridine (27 µL, 0.20 mmol) and DMSO:water (2:3) (0.2 M) was placed in the UV light bath (370 nm) at 65 ^{o}C to afford triazole **3i** (44 mg) in 76% yield after purification by column chromatography using silica (EtOAc/hexanes = 20:80).

For 1 equiv. of azide: A mixture of 1-(azidomethyl)-3-bromobenzene (21.1 mg, 0.10 mmol), alkene **5** (23.6 mg, 0.10 mmol), 2,4,6-trimethyl pyridine (13.5 µL, 0.10 mmol) and DMSO:water (2:3) (0.2 M) was placed in the UV light bath (370 nm) at 65 °C to afford triazole **3i** in 70% yield calculated from 1H NMR, (after workup) by adding 4-methyl benzyl chloride as an internal standard.

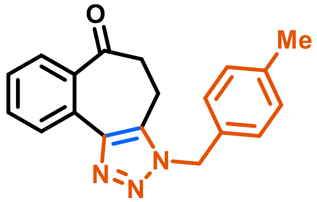

3j. 3-(4-Methylbenzyl)-4,5-dihydrobenzo[3,4]cyclohepta[1,2-d][1,2,3]triazol-6(3H)-one was prepared by **general procedure C**. A mixture of 1-(azidomethyl)-2-methylbenzene (88.2 mg, 0.60 mmol), alkene **5** (47.2 mg, 0.20 mmol), 2,4,6 trimethyl pyridine (27 µL, 0.20 mmol) and DMSO:water (2:3) (0.2 M) was placed in the UV light bath (370 nm) at 65 °C to afford triazole **3j** (53.3 mg) in 88% yield after purification by column chromatography using silica (EtOAc/hexanes = 20:80).

For 1 equiv. of azide: A mixture of 1-(azidomethyl)-2-methylbenzene (14.7 mg, 0.10 mmol), alkene **5** (23.6 mg, 0.10 mmol), 2,4,6-trimethyl pyridine (13.5 µL, 0.10 mmol) and DMSO:water (2:3) (0.2 M) was placed in the UV light bath (370 nm) at 65 °C to afford triazole **3j** in 77% yield calculated from 1H NMR, (after workup) by adding 4-methyl benzyl chloride as an internal standard.

At 395 nm: A mixture of 1-(azidomethyl)-2-methylbenzene (88.2 mg, 0.60 mmol), alkene **5** (47.2 mg, 0.20 mmol), 2,4,6 trimethyl pyridine (27 µL, 0.20 mmol) and DMSO:water (2:3) (0.2 M) was placed in the 395 nm light bath at 60 °C for 72 h to afford triazole **3j** in 81% yield calculated from 1H NMR, (after workup) by adding 4-methyl benzyl chloride as an internal standard.

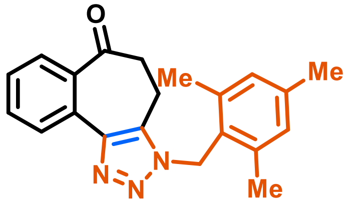

3k. 3-(2,4,6-Trimethylbenzyl)-4,5-dihydrobenzo[3,4]cyclohepta[1,2-d][1,2,3]triazol-6(3H)-one was prepared by **general procedure C**. A mixture of 2-(azidomethyl)-1,3,5-trimethylbenzene (105 mg, 0.60 mmol), alkene **5** (47.2 mg, 0.20 mmol), 2,4,6-trimethyl pyridine (27 µL, 0.20 mmol) and DMSO:water (2:3) (0.2 M) was placed in the UV light bath (370 nm) at 65 °C to afford triazole **3k** (31.1 mg) in 47% yield as yellow solid after purification by column chromatography using silica (EtOAc/hexanes = 20:80). ^{1}H NMR (400 MHz, $CDCl_3$) δ 8.63 (dd, *J* = 8.1, 1.3 Hz, 1H), 7.76 (dd, *J* = 7.8, 1.5 Hz, 1H), 7.66 – 7.53 (m, 1H), 7.32 (td, *J* = 7.5, 1.3 Hz, 1H), 6.89 (s, 2H), 5.53 (s, 2H), 3.06 – 2.95 (m, 2H), 2.83 – 2.73 (m, 2H), 2.28 (s, 3H), 2.24 (s, 6H). ^{13}C NMR (201 MHz, $CDCl_3$) δ 201.2, 143.4, 138.9, 137.6, 136.1, 133.8, 132.9, 130.0, 129.9, 129.1, 127.6, 127.0, 126.7, 47.9, 39.3, 21.1, 20.2, 19.5. Calculated HRMS(ESI) for $C_{21}H_{21}N_3O$ (M+H)+ is 332.1757 observed 332.1765. mp – 157-159 °C.

For 1 equiv. of azide: A mixture of 1-(azidomethyl)-2-methylbenzene (17.5 mg, 0.10 mmol), alkene **5** (23.6 mg, 0.10 mmol), 2,4,6-trimethyl pyridine (13.5 µL, 0.10 mmol) and DMSO:water (2:3) (0.2 M) was placed in the UV light bath (370 nm) at 65 °C to afford triazole **3k** in 56% yield

calculated from 1H NMR, (after workup) by adding 4-methyl benzyl chloride as an internal standard.

Photonic Efficiency Experiment:

A convenient actinometer described by Pitre, Scaiano, and Yoon[5] was used to assess the incident light (I_0) from our UV LEDs. 3 mL of the actinometer solution was placed in a test tube and immersed in the lights for 30 seconds. A comparison of the absorbance at 372 nm before and after the irradiation for 30 seconds indicated a change of 0.580. According to the following equation, that amounts to an I_0 of 2.8 x 10^{-7} $mol\ hv\ s^{-1}$.

$$I_0 = \frac{V\frac{(\Delta A_{372nm})}{(\varepsilon)(l)}}{(\Phi)(t)} = \frac{0.003\ L\frac{(0.580)}{(11100\ M^{-1}cm^{-1})(1\ cm)}}{(0.019)(30\ s)} = 2.75\ \times 10^{-7}$$

V is the sample volume, ε is the molar absorptivity of 9,10-diphenylanthracene, l is the path length of the cuvette, Φ is the quantum yield of the actinometer, and t is the irradiation time in seconds. From this valuation of I_0, the photonic efficiency of the reaction between Br-oxo BC7, azide and collidine was determined with a 0.1 mmol scale reaction. Br-oxo BC7 (23.6 mg, 0.1 mmol), 4-fluoro azide (15.1 mg, 0.1 mmol), 2,4,6- trimethyl pyridine (0.1 mmol, mg), and DMSO:water (2:3) (0.1 M) was placed in an NMR tube (sealed using a rubber septum with parafilm, purged with argon) in the UV light bath (370 nm) at 65 °C. After 3 hours, the reaction mixture was extracted with DCM (2 x 5 mL) and deionized water (5 mL) to determine the conversion (40% to triazole, or approximately 3.8 x 10^{-9} mol per second).

Photonic efficiency $= (\frac{rate\ of\ triazole\ formation}{I_0})$ was determined to be 0.0136 over the first 40% conversion.

Synthesis of alkene 6.

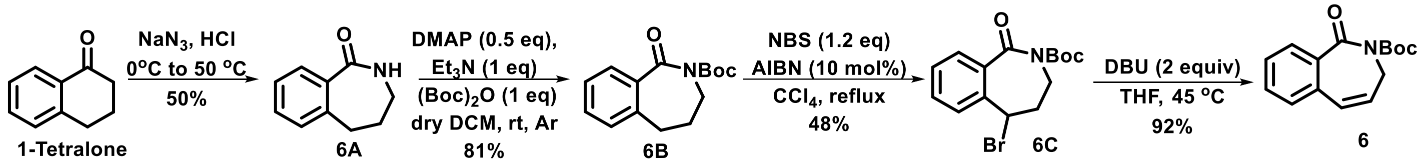

Alkene **6** was synthesized by the reported procedure.[3]

Synthesis of alkene 8 (Bromo-lactam BC7).

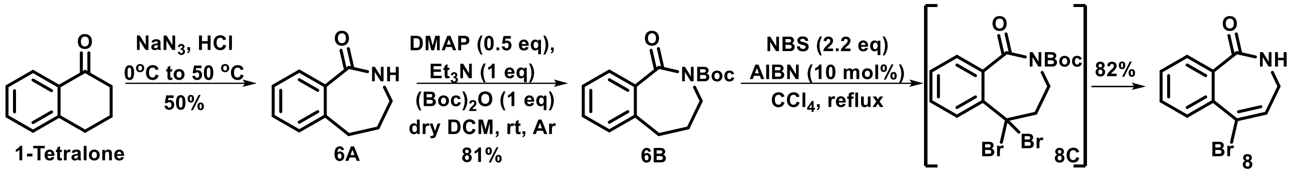

Alkene **8** was synthesized by the reported procedure.[3]

Absorption spectra of alkenes 6 and 8.

Absorption spectra were recorded on a Shimadzu UV-2600 UV-vis spectrometer at reaction concentration (approximately 0.05 mM) in in MeCN for **6** and MeOH for **8** at 25 °C.

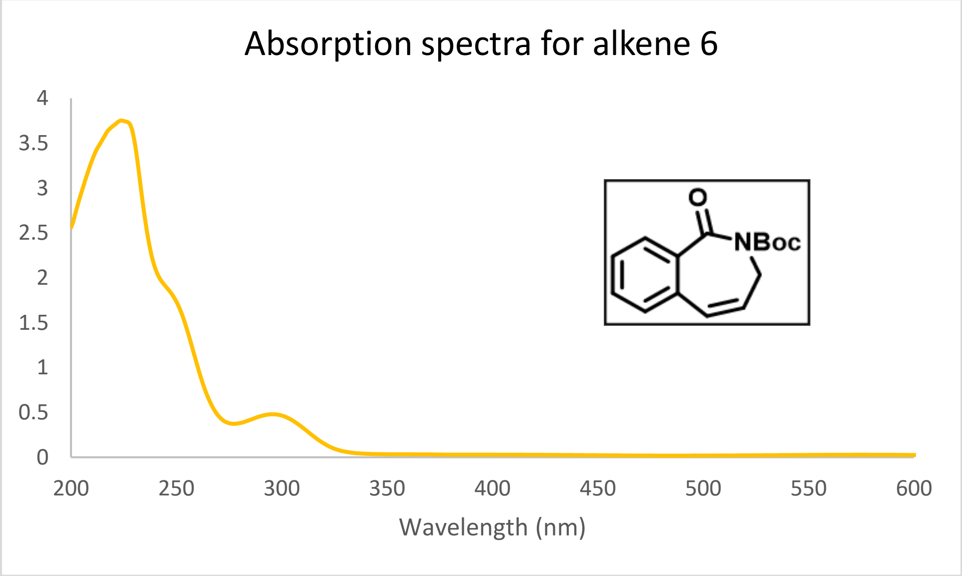

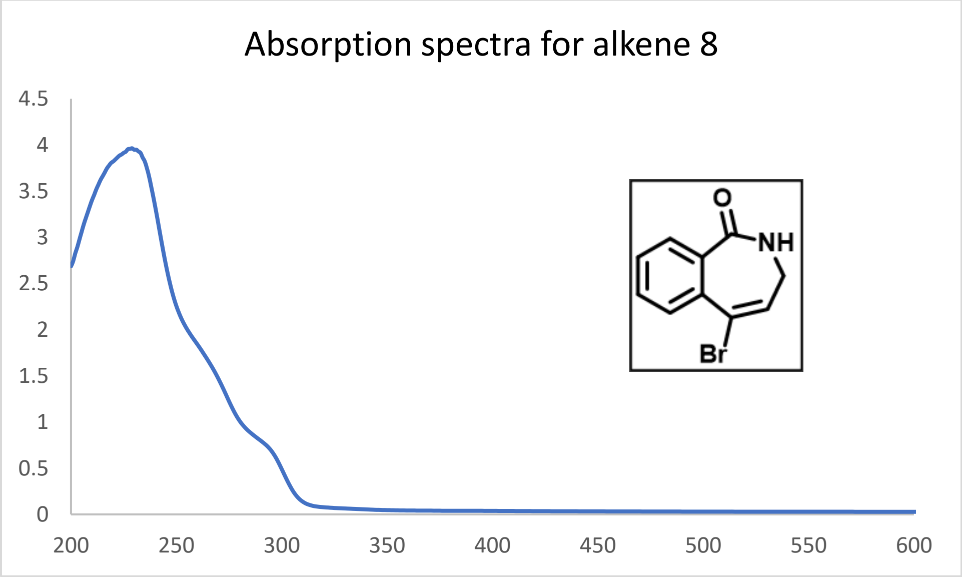

Catalyst free light driven cycloaddition with alkene 8.

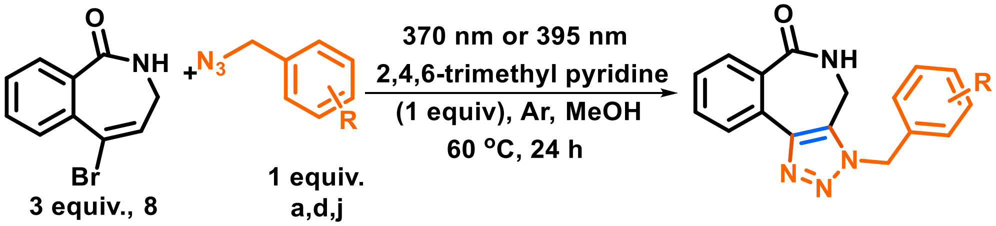

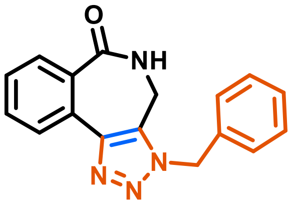

8a. 3-Benzyl-4,5-dihydrobenzo[c][1,2,3]triazolo[4,5-e]azepin-6(3H)-one was prepared by **general procedure D**. A mixture of benzyl azide (26.6 mg, 0.20 mmol), alkene **8** (142.2 mg, 0.30 mmol), 2,4,6-trimethyl pyridine (27 µL, 0.20 mmol) and MeOH (0.2 M) was placed in the UV light bath (370 nm) at 60 °C for 24 h to afford triazole **8a** (34.8 mg) as yellow oil in 60% yield after purification by column chromatography using silica (EtOAc/hexanes = 80:20). ^{1}H NMR (800 MHz, $CDCl_3$) δ 8.1 (dd, *J* = 7.9, 1.3 Hz, 1H), 8.1 (dd, *J* = 7.9, 1.4 Hz, 1H), 7.6 (td, *J* = 7.6, 1.4 Hz, 1H), 7.5 (td, *J* = 7.6, 1.3 Hz, 1H), 7.3 – 7.3 (m, 2H), 7.3 – 7.3 (m, 1H), 7.2 (dd, *J* = 7.0, 1.8 Hz, 2H), 7.2 (d, *J* = 5.6 Hz, 1H), 5.6 (s, 2H), 4.1 (d, *J* = 5.7 Hz, 2H). ^{13}C NMR (201 MHz, $CDCl_3$) δ 171.4, 146.2, 134.4, 133.1, 132.4, 132.2, 131.3, 129.5, 129.0, 128.7, 128.5, 127.1, 126.7, 52.7, 33.9. Calculated HRMS(ESI) for $C_{17}H_{14}N_4O$ (M+H)+ is 291.1240 observed 291.1244.

At 395 nm: A mixture of benzyl azide (13.3 mg, 0.10 mmol), alkene **8** (71.1 mg, 0.30 mmol), 2,4,6-trimethyl pyridine (13.5 µL, 0.10 mmol) and MeOH (0.2 M) was placed in the 395 nm light bath at 60 °C for 72 h to afford triazole **8a** in 91% yield calculated from 1H NMR, (after workup) by adding 4-methyl benzyl chloride as an internal standard.

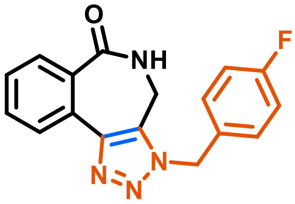

8d. 3-(4-Fluorobenzyl)-4,5-dihydrobenzo[c][1,2,3]triazolo[4,5-e]azepin-6(3H)-one was prepared by **general procedure D**. A mixture of 1-(azidomethyl)-4-fluorobenzene (30.2 mg, 0.20 mmol), alkene **8** (142.2 mg, 0.30 mmol), 2,4,6-trimethyl pyridine (27 µL, 0.20 mmol) and MeOH (0.2 M) was placed in the UV light bath (370 nm) at 60 °C to afford triazole **8d** (32.0 mg) in 52% yield as yellow oil after purification by column chromatography using silica (EtOAc/hexanes = 80:20). ^{1}H NMR (800 MHz, $CDCl_3$) δ 8.1 (dd, *J* = 7.7, 1.3 Hz, 1H), 8.1 (dd, *J* = 7.9, 1.4 Hz, 1H), 7.8 (t, *J* = 5.6 Hz, 1H), 7.6 (td, *J* = 7.6, 1.4 Hz, 1H), 7.5 (td, *J* = 7.6, 1.3 Hz, 1H), 7.2 (dd, *J* = 8.6, 5.2 Hz, 2H), 7.0 – 7.0 (m, 2H), 5.6 (s, 2H), 4.1 (d, *J* = 5.6 Hz, 2H). ^{13}C NMR (201 MHz, $CDCl_3$) δ 171.7, 162.9 (d, *J* = 248.2 Hz), 146.1, 133.1, 132.4, 132.1, 131.3, 130.3 (d, *J* = 3.2 Hz), 129.1 (d, *J* = 8.7 Hz), 128.6, 128.5, 126.7, 116.5 (d, *J* =

21.7 Hz), 51.8, 33.8. ^{19}F NMR (376 MHz, $CDCl_3$) δ -112.4 (tt, *J* = 9.0, 5.2 Hz). Calculated HRMS(ESI) for $C_{17}H_{13}FN_4O$ (M+H)+ is 309.1146 observed 309.1150.

At 395 nm: A mixture of 1-(azidomethyl)-4-fluorobenzene (15.1 mg, 0.10 mmol), alkene **8** (71.1 mg, 0.30 mmol), 2,4,6-trimethyl pyridine (13.5 µL, 0.10 mmol) and MeOH (0.2 M) was placed in the 395 nm light bath at 60 °C for 72 h to afford triazole **8d** in 81% yield calculated from 1H NMR, (after workup) by adding 4-methyl benzyl chloride as an internal standard.

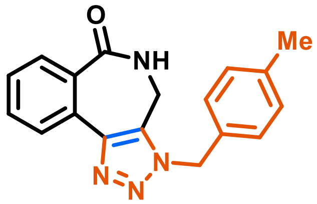

8j. 3-(4-Methylbenzyl)-4,5-dihydrobenzo[c][1,2,3]triazolo[4,5-e]azepin-6(3H)-one was prepared by **general procedure D**. A mixture of 1-(azidomethyl)-4-methylbenzene (29.4 mg, 0.20 mmol), alkene **8** (142.2 mg, 0.30 mmol), 2,4,6-trimethyl pyridine (27 µL, 0.20 mmol) and MeOH (0.2 M) was placed in the UV light (370 nm) bath at 60 °C to afford triazole **8j** (34.6 mg) in 57% yield as yellow oil after purification by column chromatography using silica (EtOAc/hexanes = 80:20). ^{1}H NMR (800 MHz, $CDCl_3$) δ 8.1 (dd, *J* = 7.9, 1.4 Hz, 1H), 8.1 (dd, *J* = 7.8, 1.4 Hz, 1H), 7.6 (td, *J* = 7.6, 1.4 Hz, 1H), 7.5 (td, *J* = 7.6, 1.3 Hz, 1H), 7.1 (d, *J* = 7.9 Hz, 2H), 7.1 (d, *J* = 7.9 Hz, 2H), 7.0 (t, *J* = 5.8 Hz, 1H), 5.6 (s, 2H), 4.1 (d, *J* = 5.7 Hz, 2H), 2.3 (s, 0H), 2.3 (s, 3H). ^{13}C NMR (201 MHz, $CDCl_3$) δ 171.3, 146.2, 139.0, 133.0, 132.4, 132.2, 131.4, 131.2, 130.1, 128.7, 128.4, 127.1, 126.7, 52.6, 33.9, 21.3. Calculated HRMS(ESI) for $C_{18}H_{16}N_4O$ (M+H)+ is 305.1397 observed 305.1401.

At 395 nm: A mixture of 1-(azidomethyl)-4-methylbenzene (14.7 mg, 0.10 mmol), alkene **8** (71.1 mg, 0.30 mmol), 2,4,6-trimethyl pyridine (13.5 µL, 0.10 mmol) and MeOH (0.2 M) was placed in the 395 nm light bath at 60 °C for 72 h to afford triazole **8j** in 92% yield calculated from 1H NMR, (after workup) by adding 4-methyl benzyl chloride as an internal standard.

REFERENCES

1. Singh, A.; Teegardin, K.; Kelly, M.; Prasad, K. S.; Krishnan, S.; Weaver, J. D., Facile synthesis and complete characterization of homoleptic and heteroleptic cyclometalated Iridium (III) complexes for photocatalysis. *J. Organomet. Chem.* **2015,** *776*, 51-59.
2. Singh, K.; Fennell, C. J.; Coutsias, E. A.; Latifi, R.; Hartson, S.; Weaver, J. D., Light harvesting for rapid and selective reactions: click chemistry with strain-loadable alkenes. *Chem* **2018,** *4* (1), 124-137.
3. Kharbanda, S.; Alkhamayseh, O.; Eastham, G.; Weaver, J. D., Synthesis of Functionalized Benzocycloheptenes, DOI: 10.1080/28378083.2024.2394899. *Essential Chem* **2024**.
4. Karplus, M., Vicinal proton coupling in nuclear magnetic resonance. *Journal of the American Chemical Society* **1963,** *85* (18), 2870-2871.
5. Pitre, S. P.; Scaiano, J. C.; Yoon, T. P., Photocatalytic Indole Diels–Alder cycloadditions mediated by heterogeneous platinum-modified titanium dioxide. *ACS catalysis* **2017,** *7* (10), 6440-6444.

[1a] 1H NMR at 400.15 MHz in CD3CN

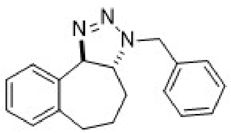

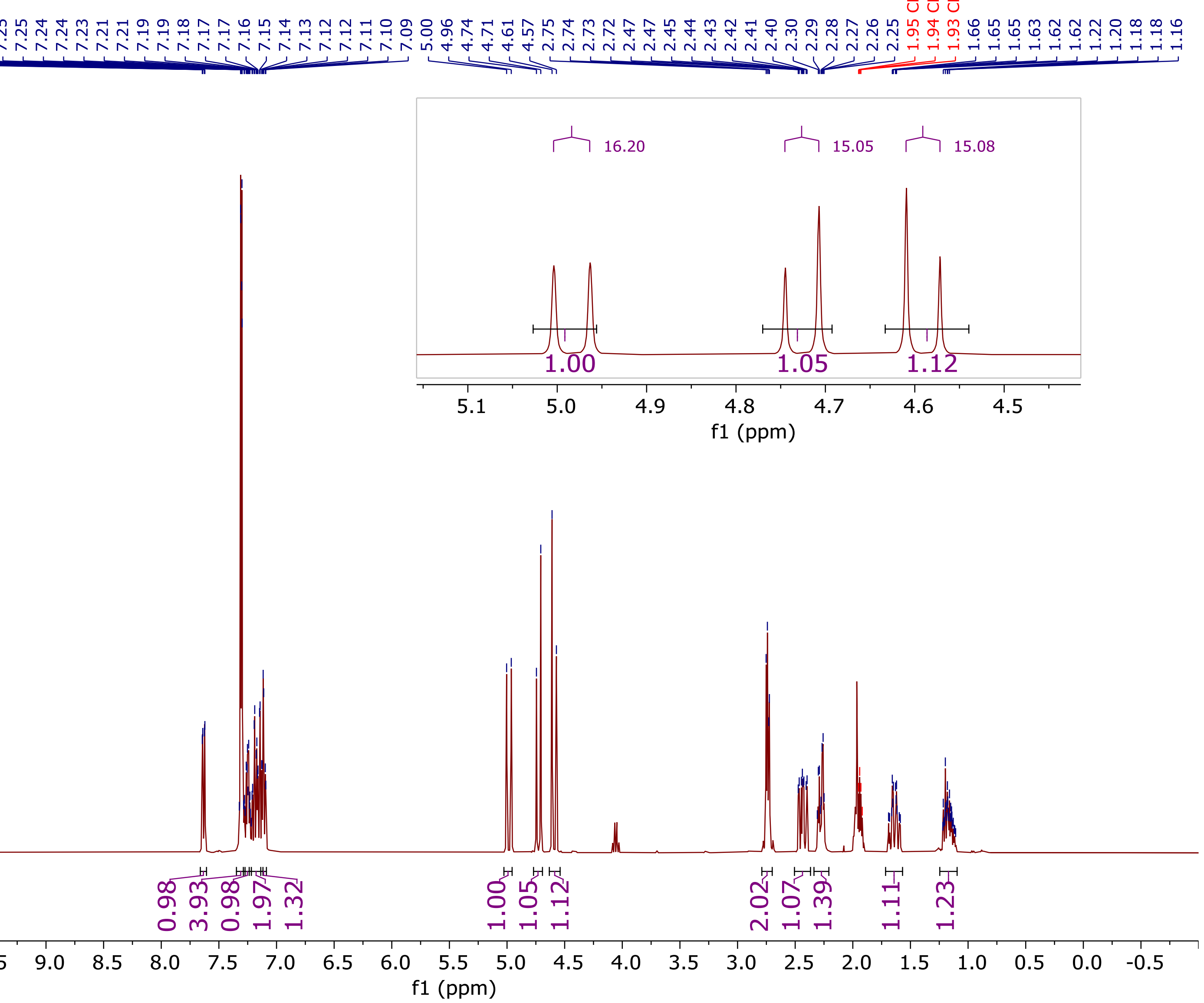

[1a] 13C NMR at 100.63 MHz in CD3CN

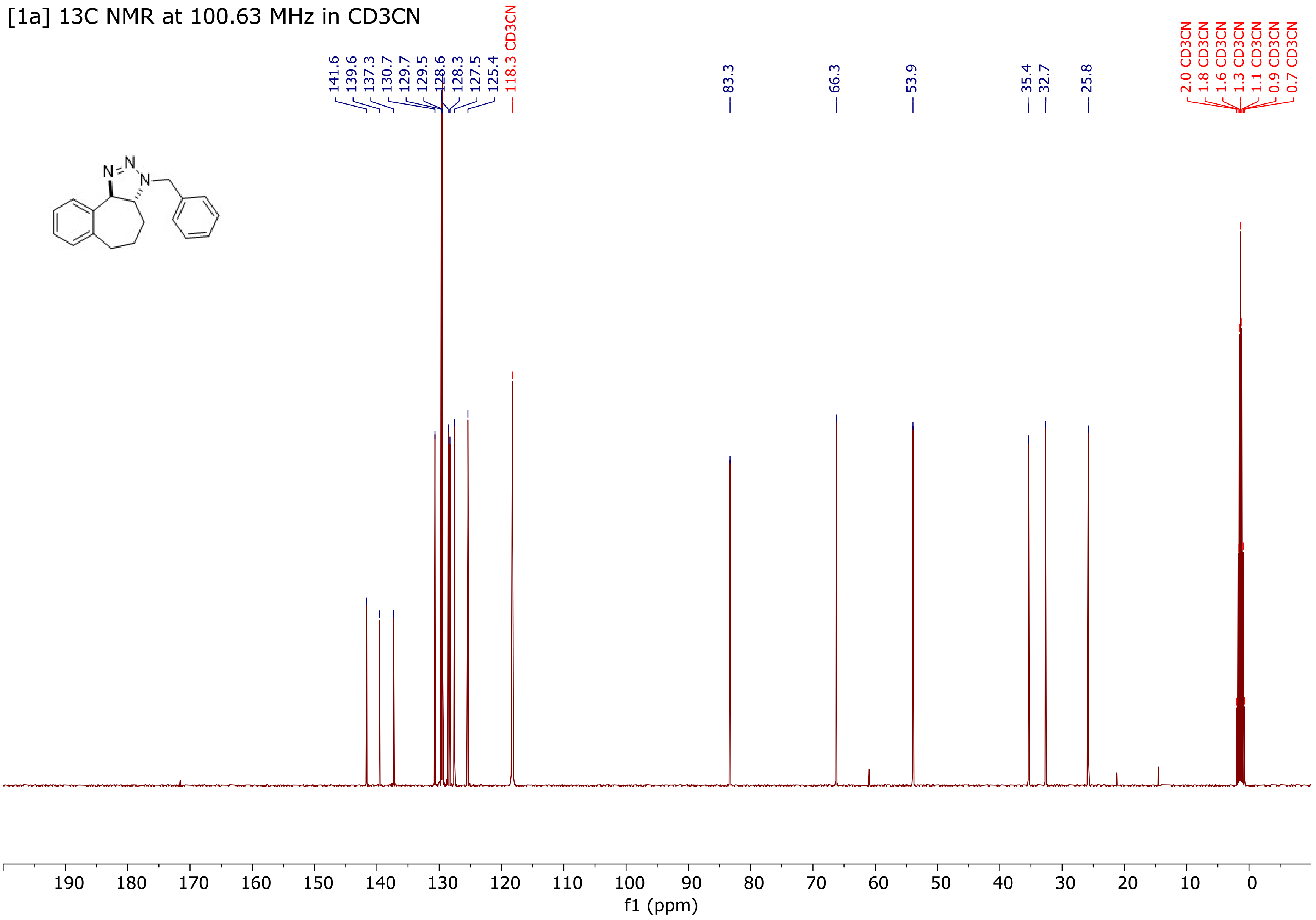

[2a] 1H NMR at 400.15 MHz in CD3CN

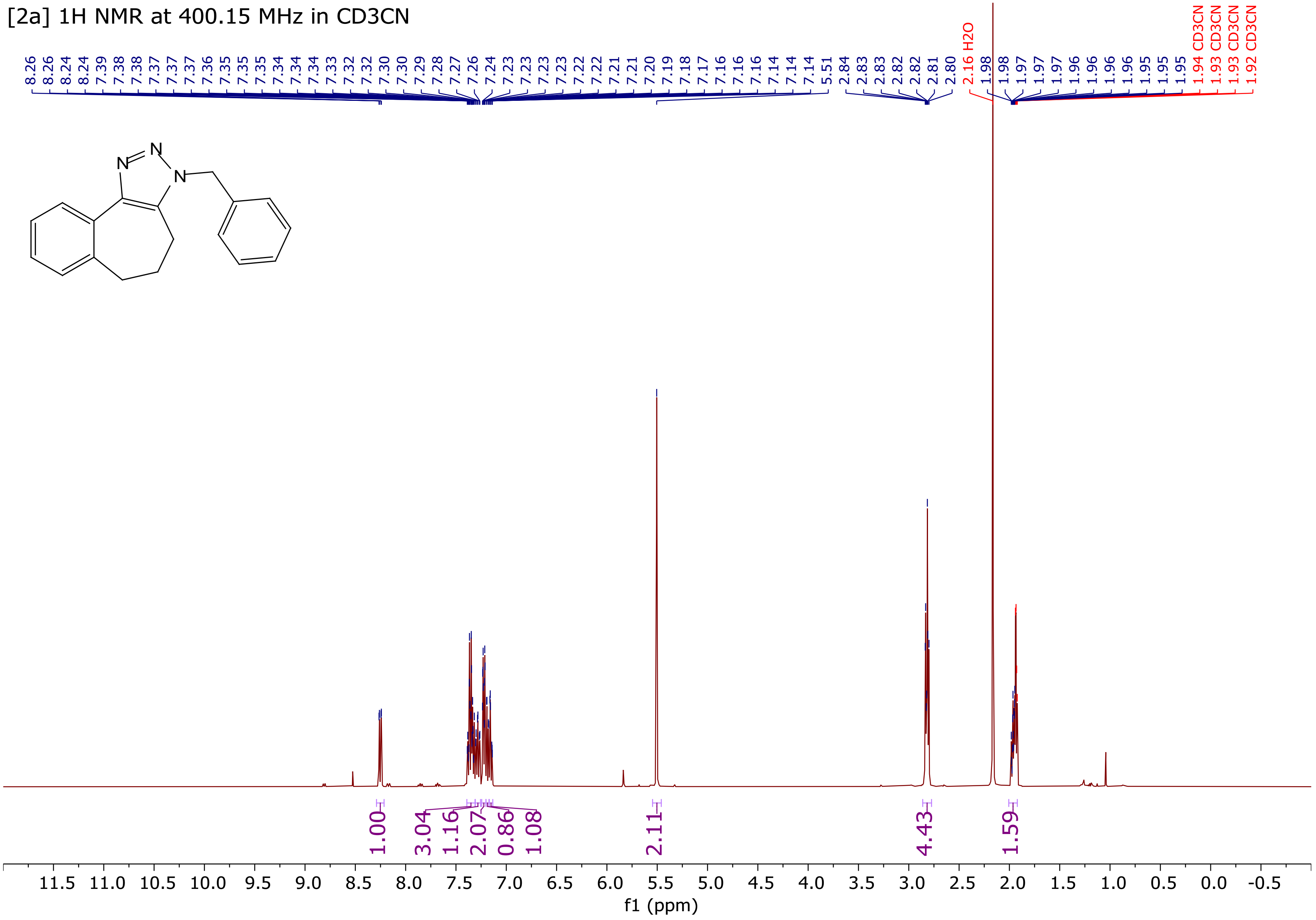

[2a] 13C NMR at 100.63 MHz in CDCl3

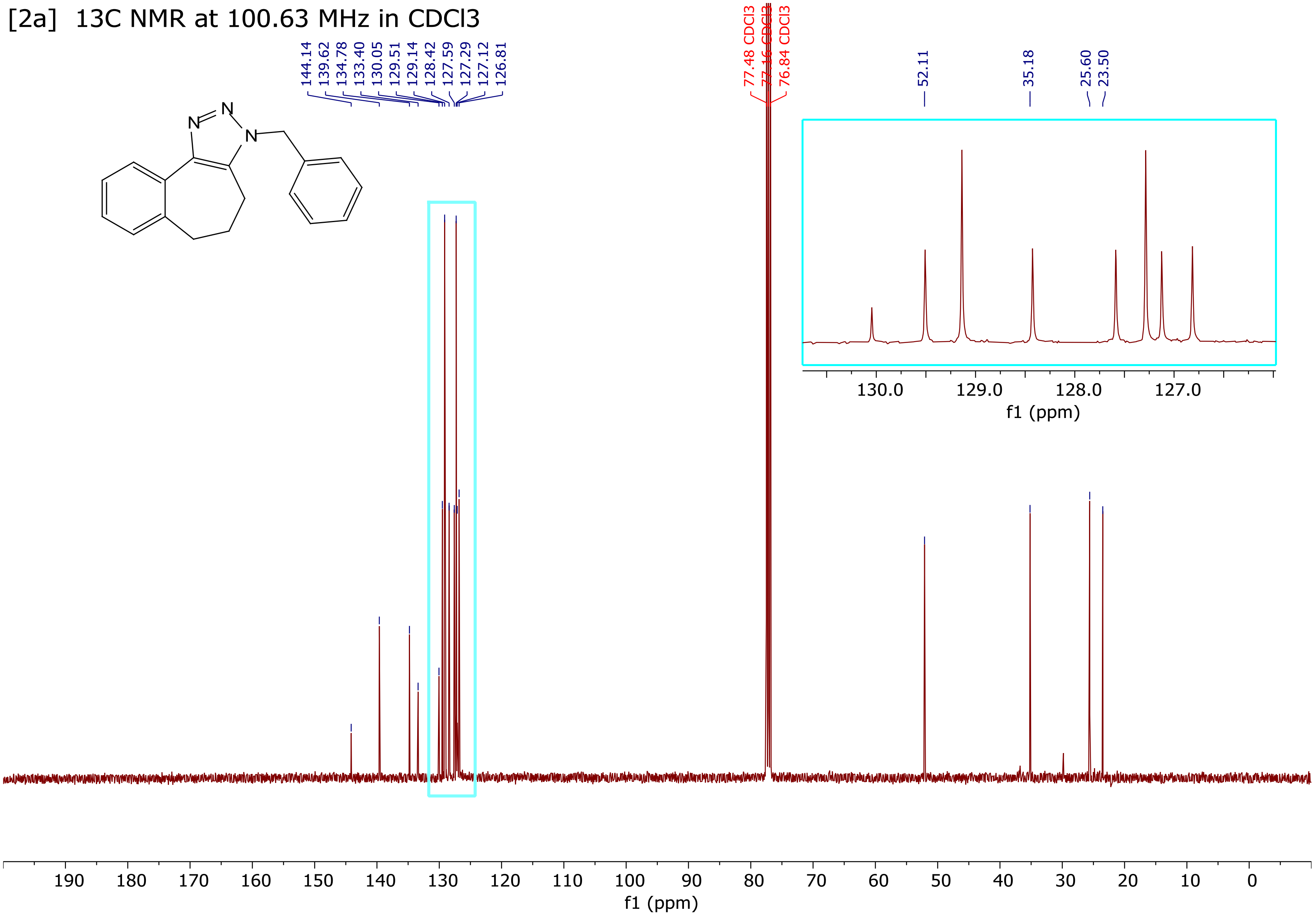

[1b] 1H NMR at 400.15 MHz in CD3CN

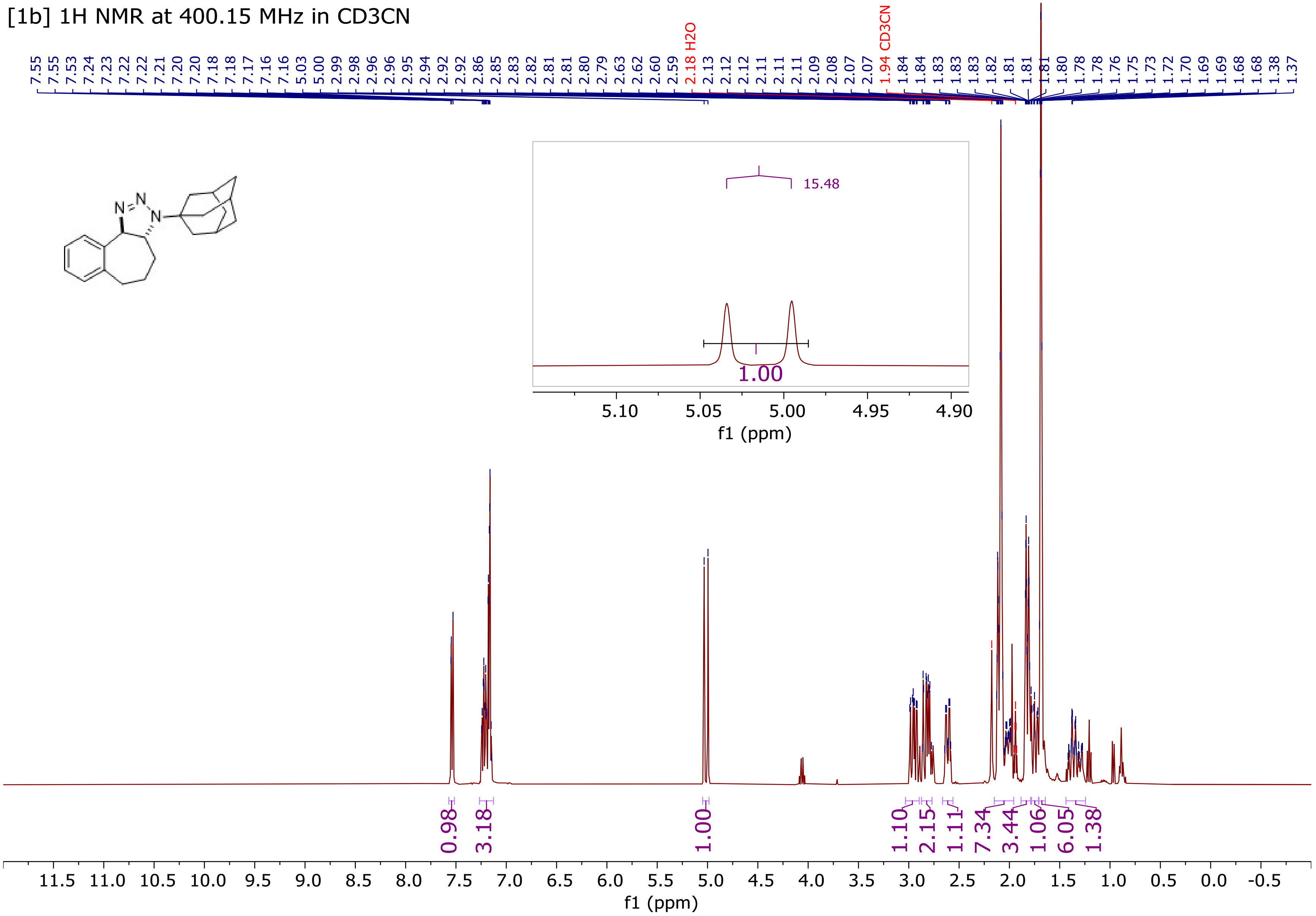

[1b] 13C NMR at 100.63 MHz in CD3CN

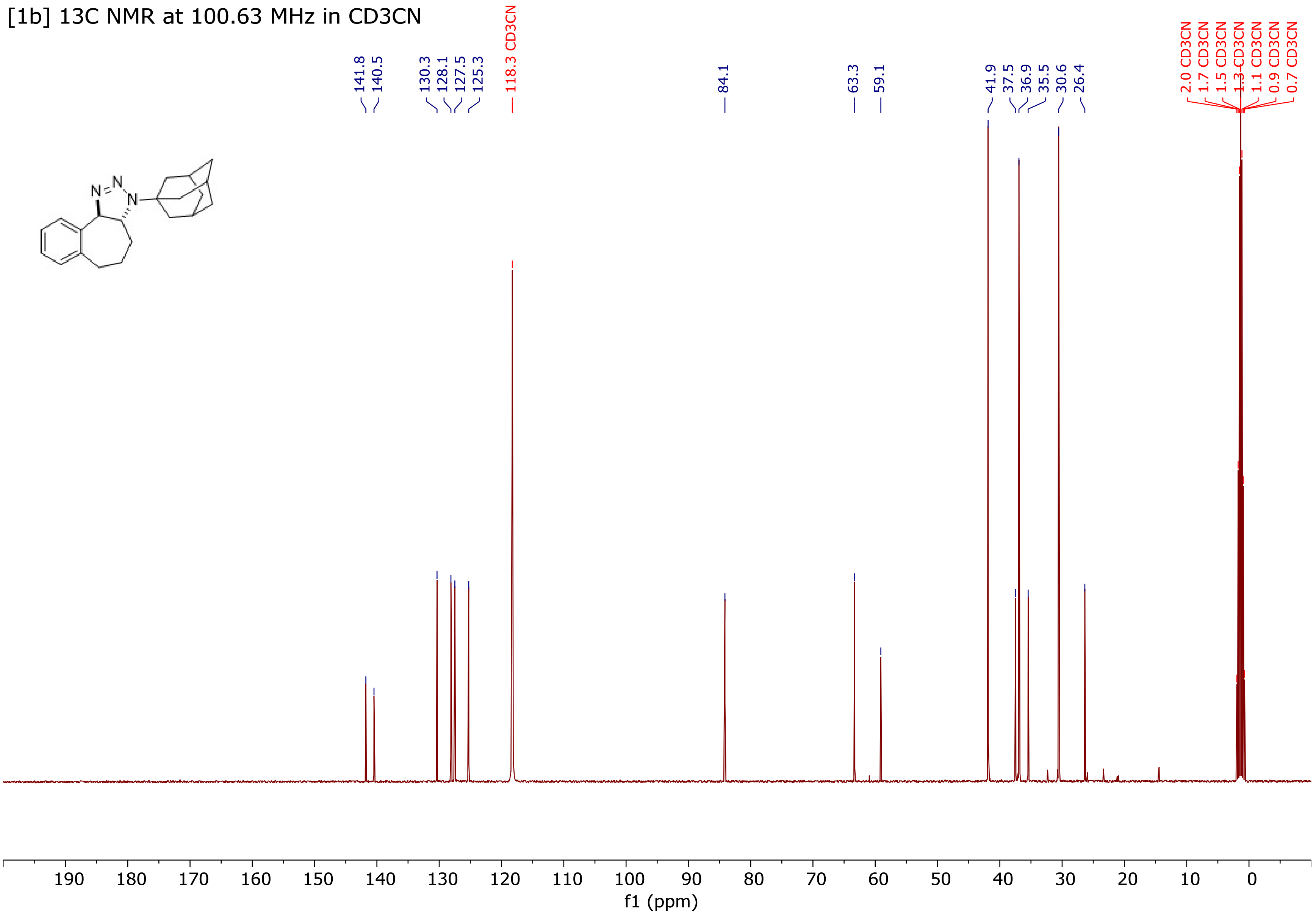

[2b] 1H NMR at 400.15 MHz in CDCl3

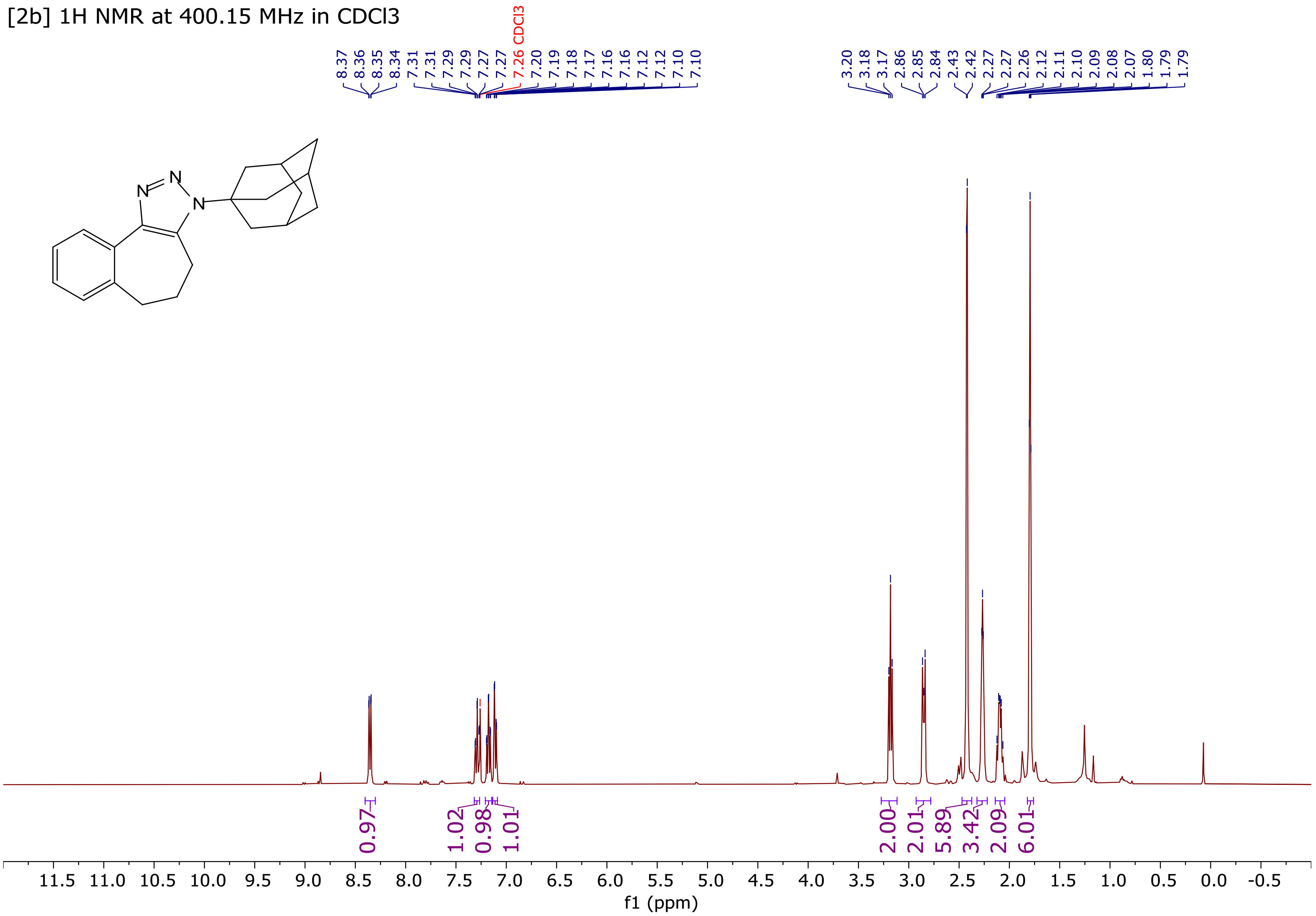

[2b] 13C NMR at 100.63 MHz in CDCl3

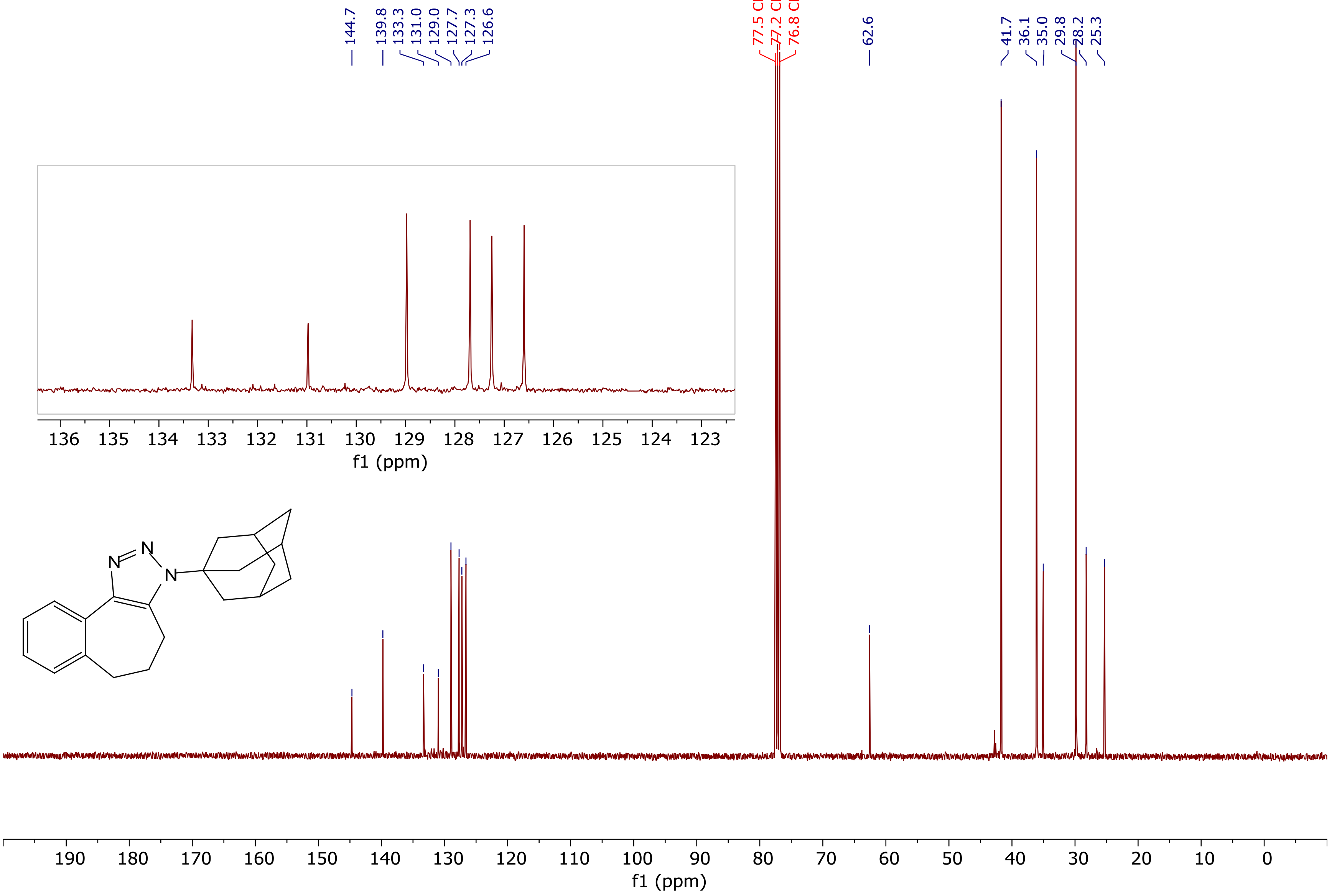

[1c] 1H NMR at 400.15 MHz in CD3CN

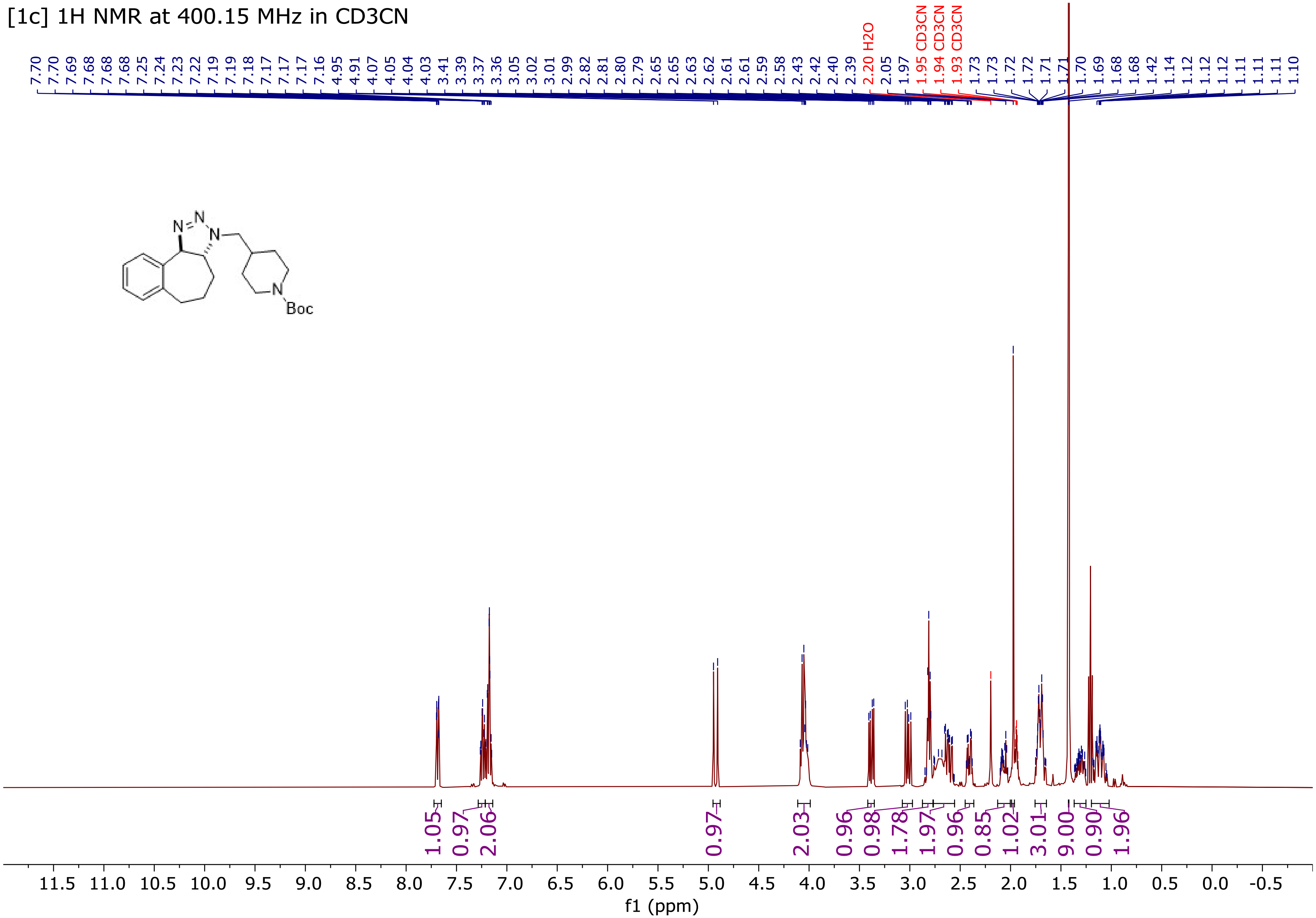

[1c] 13C NMR at 100.63 MHz in CD3CN

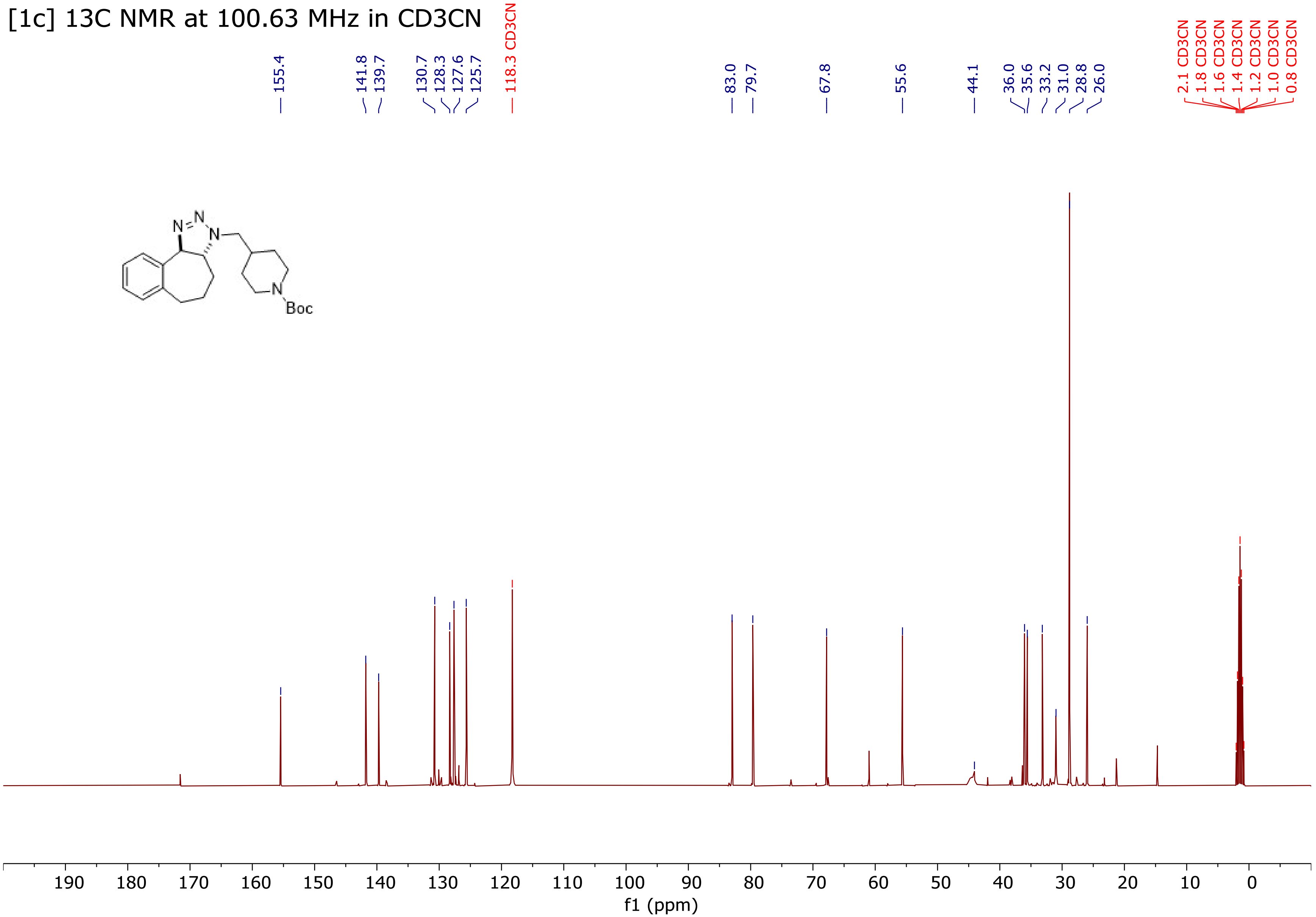

[2c] 1H NMR at 400.15 MHz in CD3CN

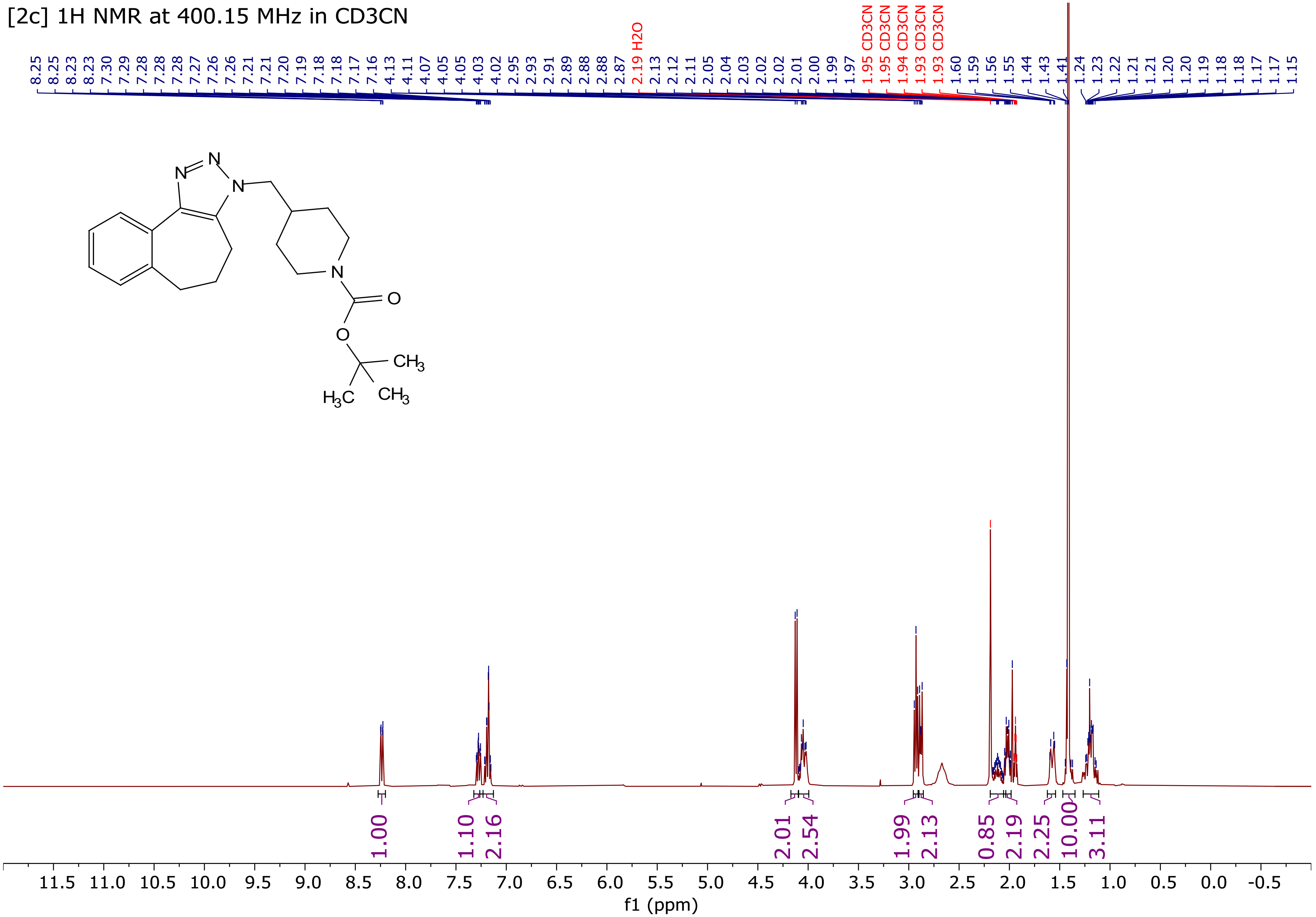

[2c] 13C NMR at 100.63 MHz in CDCl3

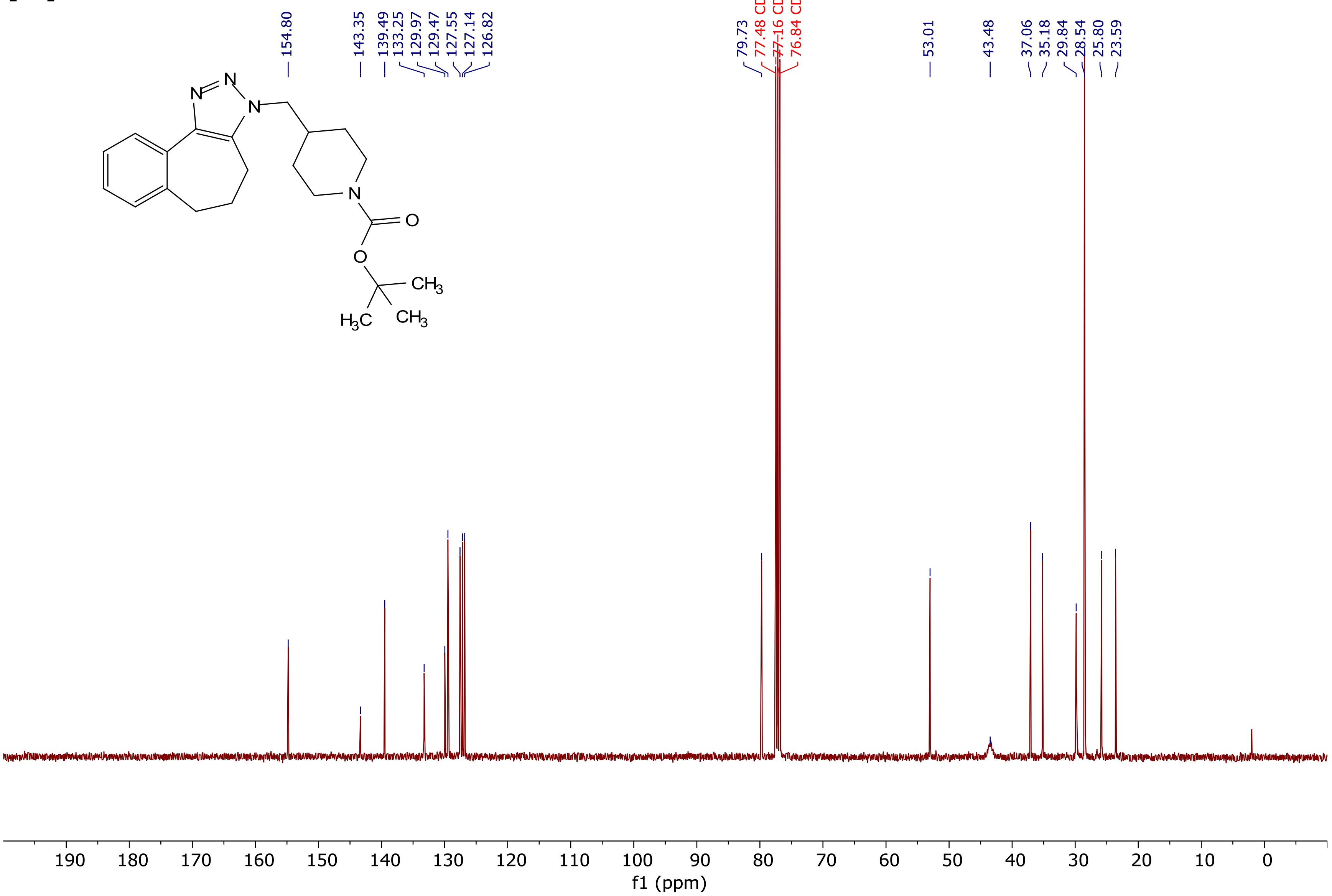

[1d] 1H NMR at 400.15 MHz in Acetone

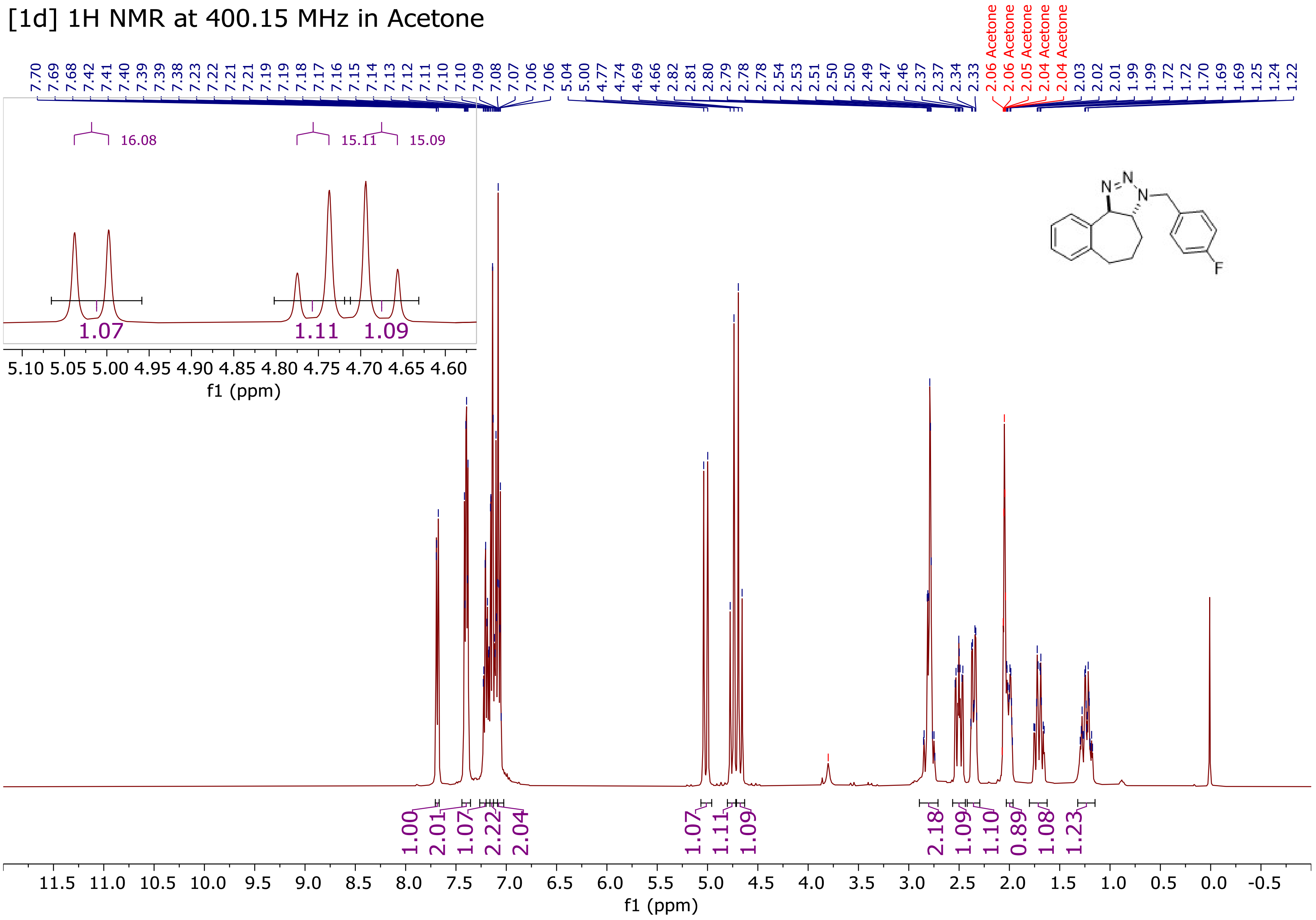

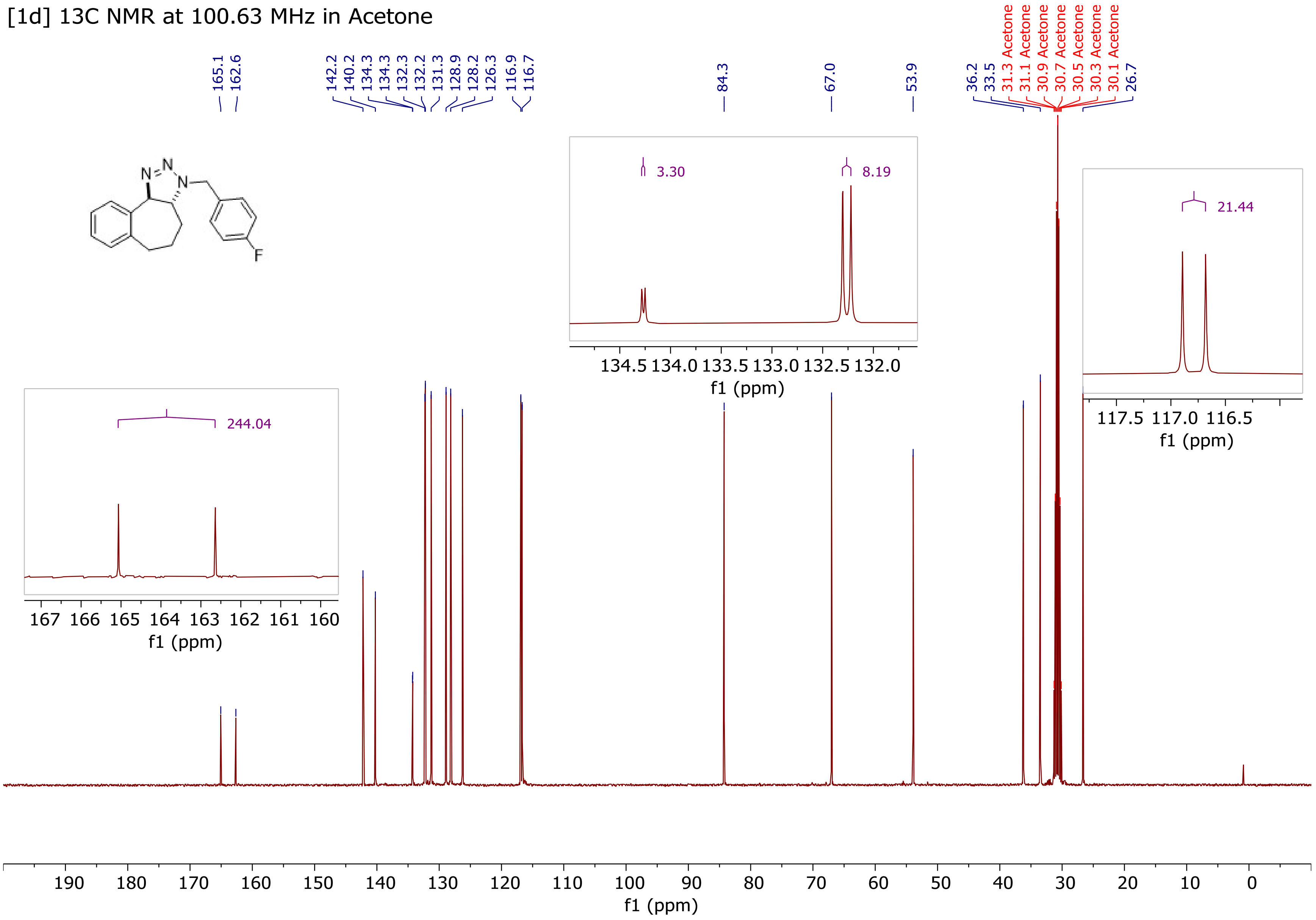
[1d] 13C NMR at 100.63 MHz in Acetone
165.1
162.6
142.2
140.2
134.3
134.3
132.3
132.2
131.3
128.9
128.2
126.3
116.9
116.7
84.3
67.0
53.9
36.2
33.5
31.3 Acetone
31.1 Acetone
30.9 Acetone
30.7 Acetone
30.5 Acetone
30.3 Acetone
30.1 Acetone
26.7
3.30
8.19
21.44
244.04
N
N
N
F
f1 (ppm)

[1d] 19F NMR at 376.48 MHz in Acetone

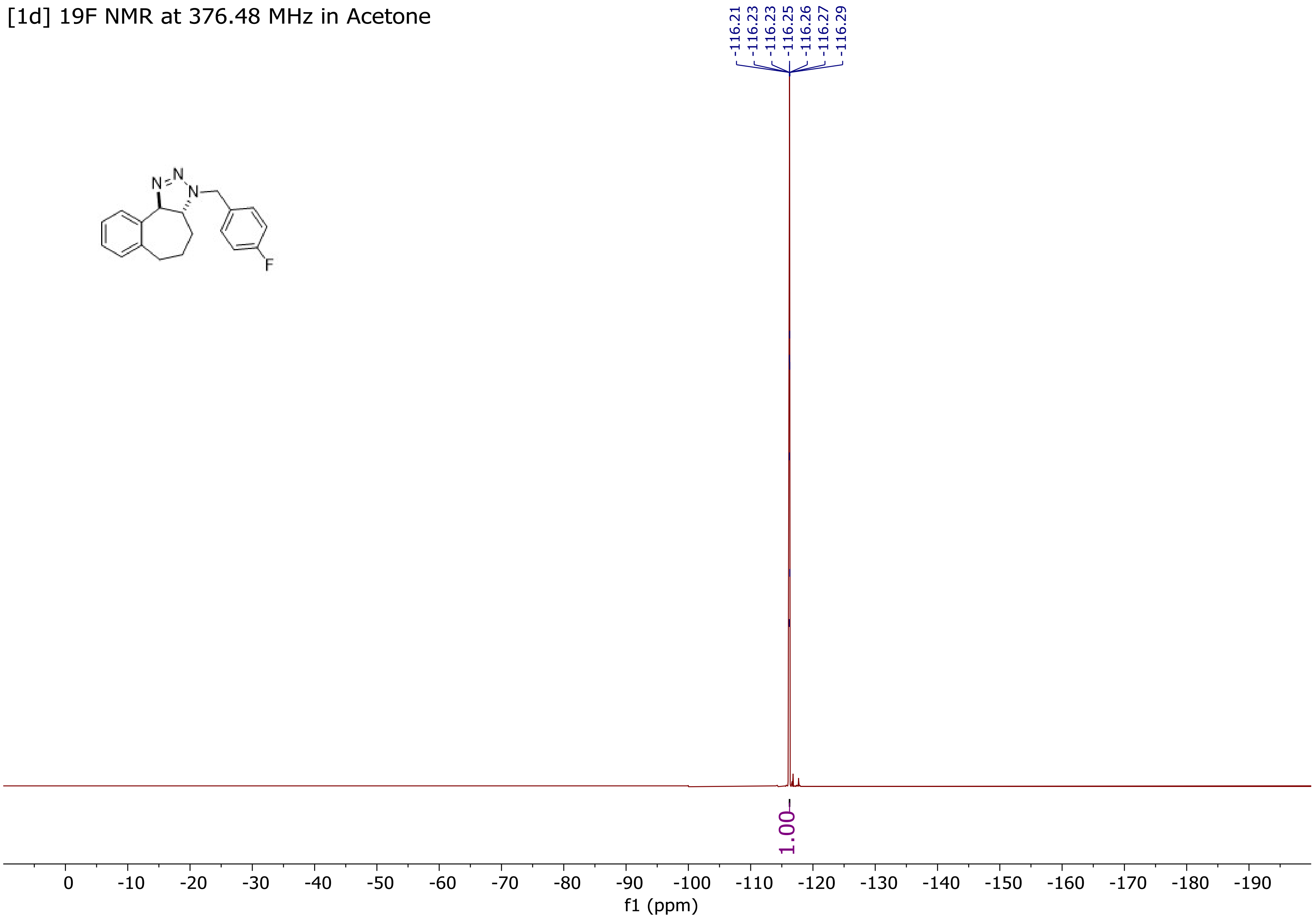

[2d] 1H NMR at 400.15 MHz in CD3CN

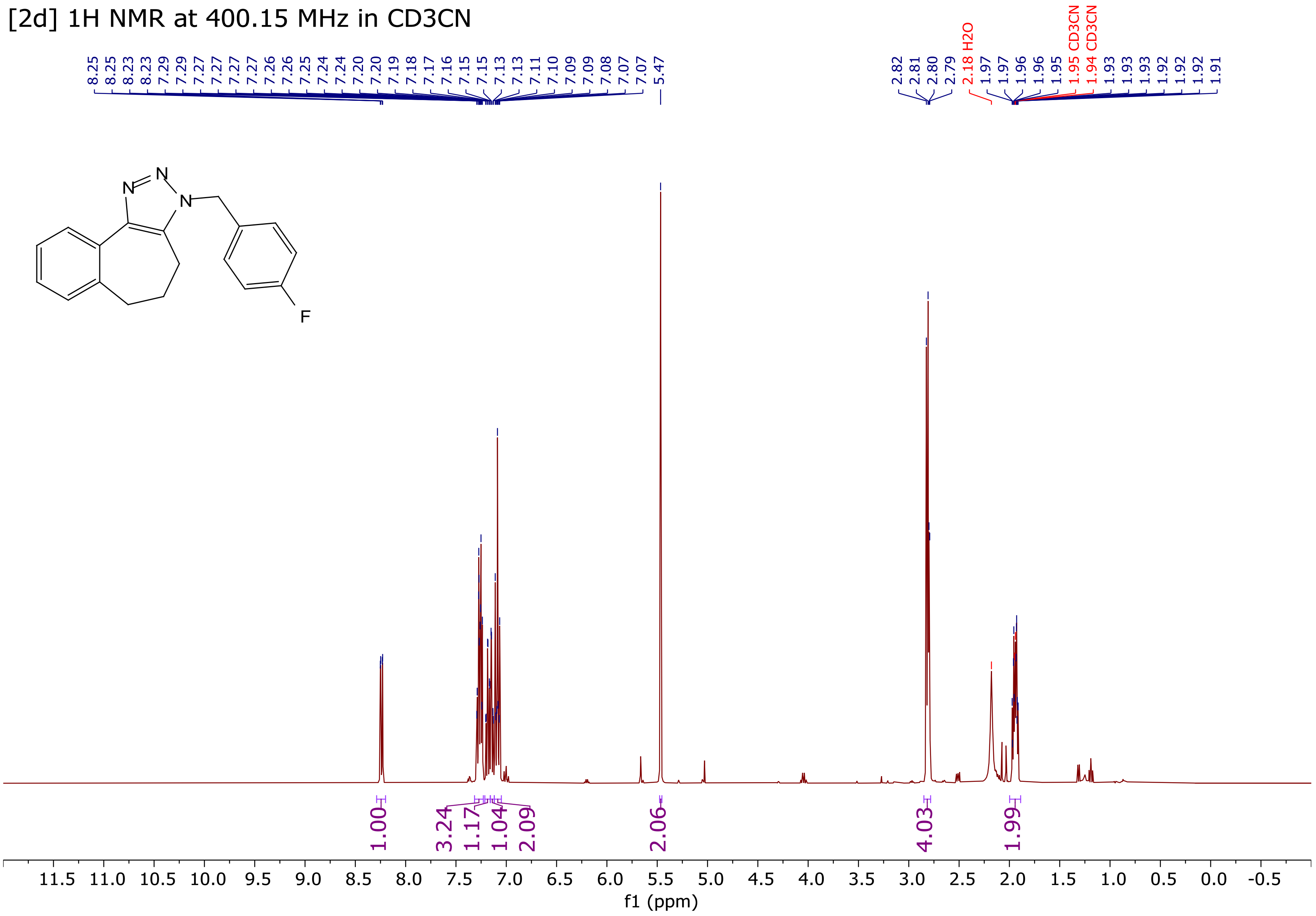

[2d] 13C NMR at 100.63 MHz in CDCl3

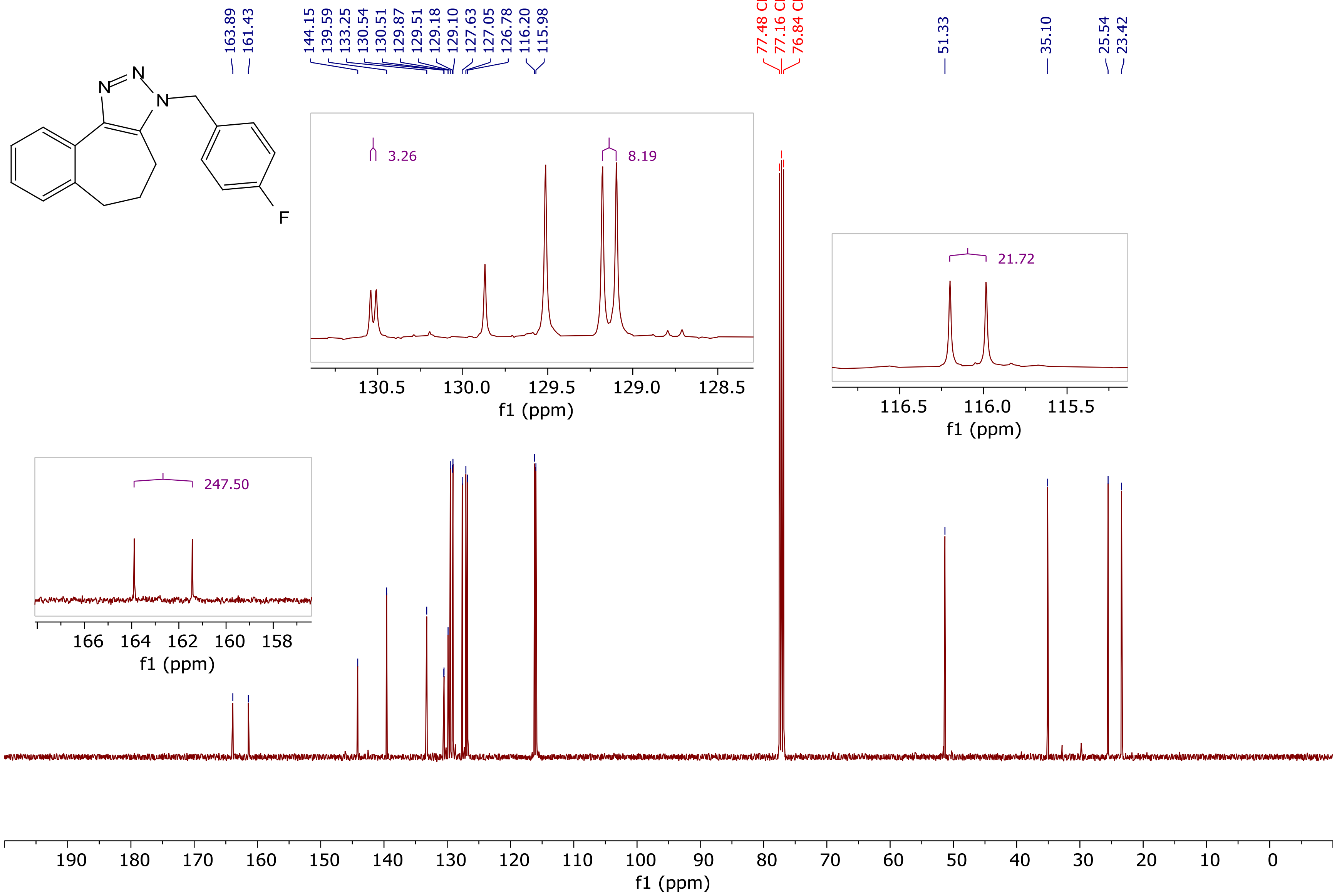

[2d] 19F NMR at 376.48 MHz in CD3CN

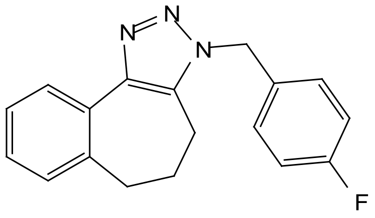

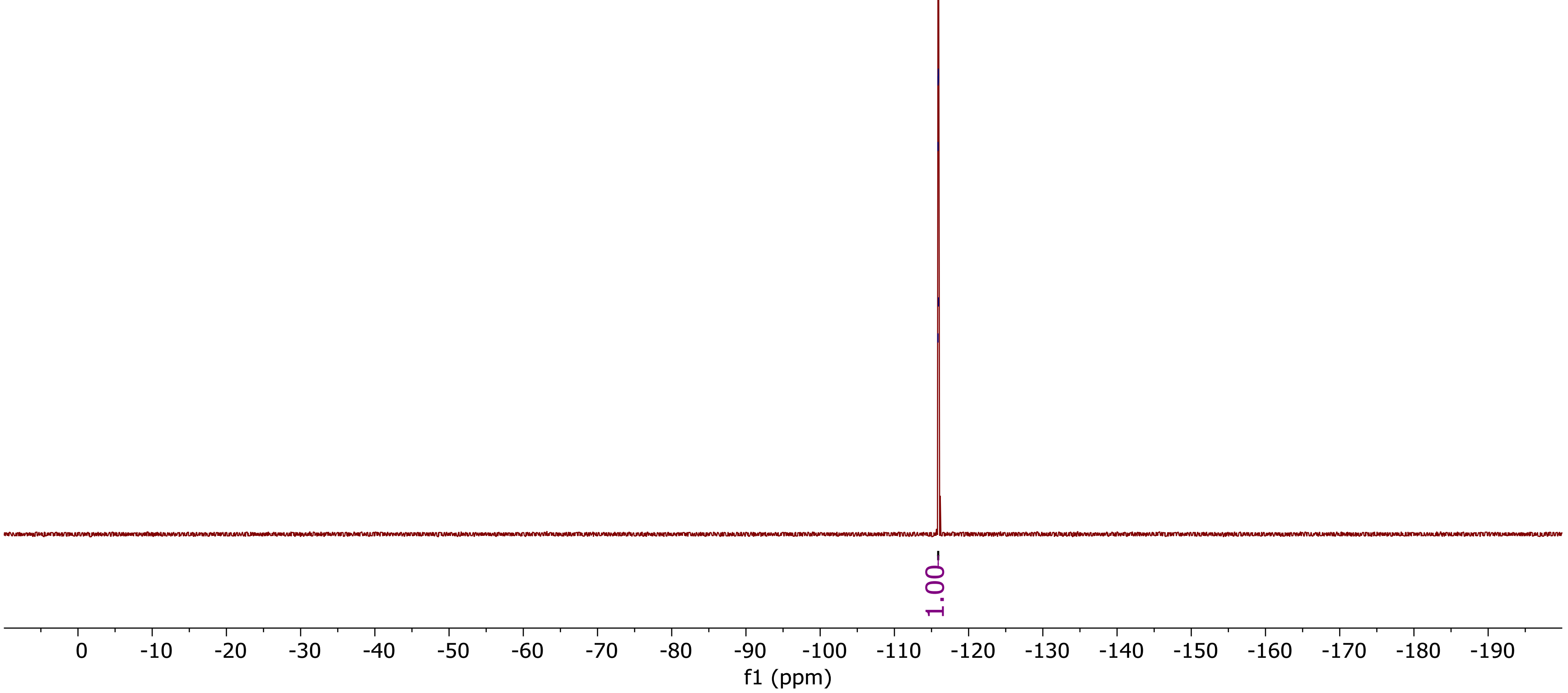

[3a] 1H NMR at 400.15 MHz in CD3CN

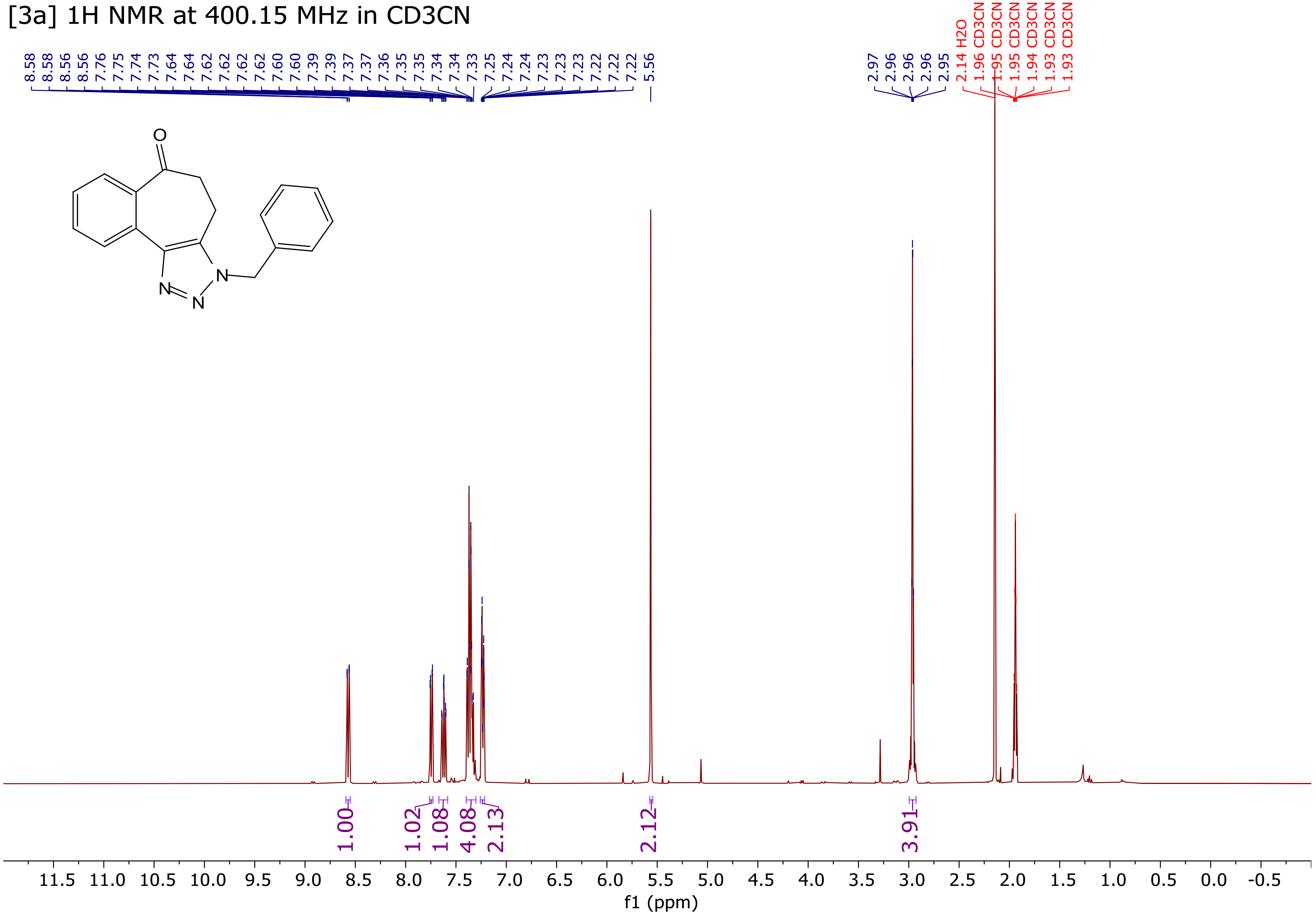

[3a] 13C NMR at 100.63 MHz in CD3CN

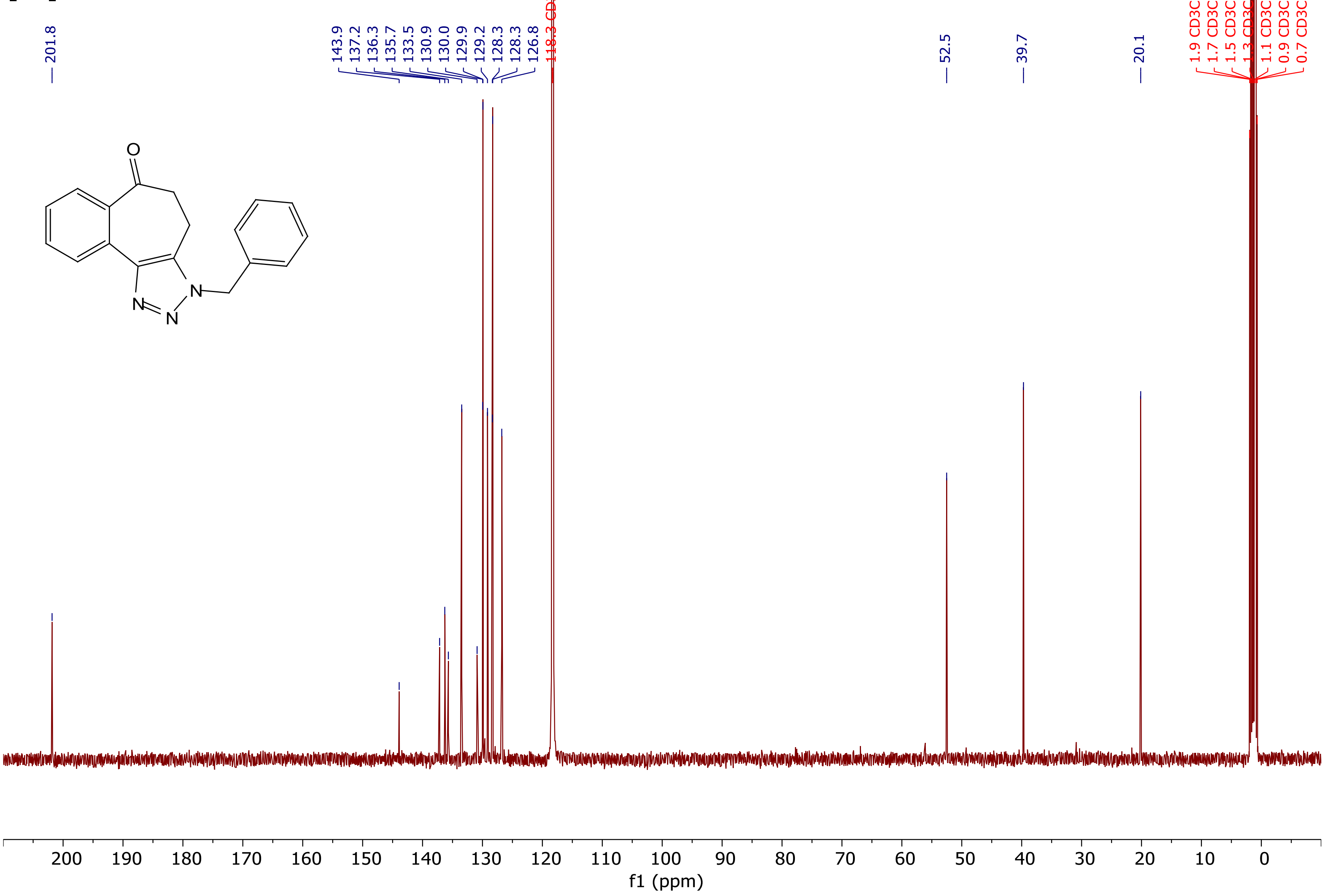

[3a"] 1H NMR at 400.15 MHz in Acetone

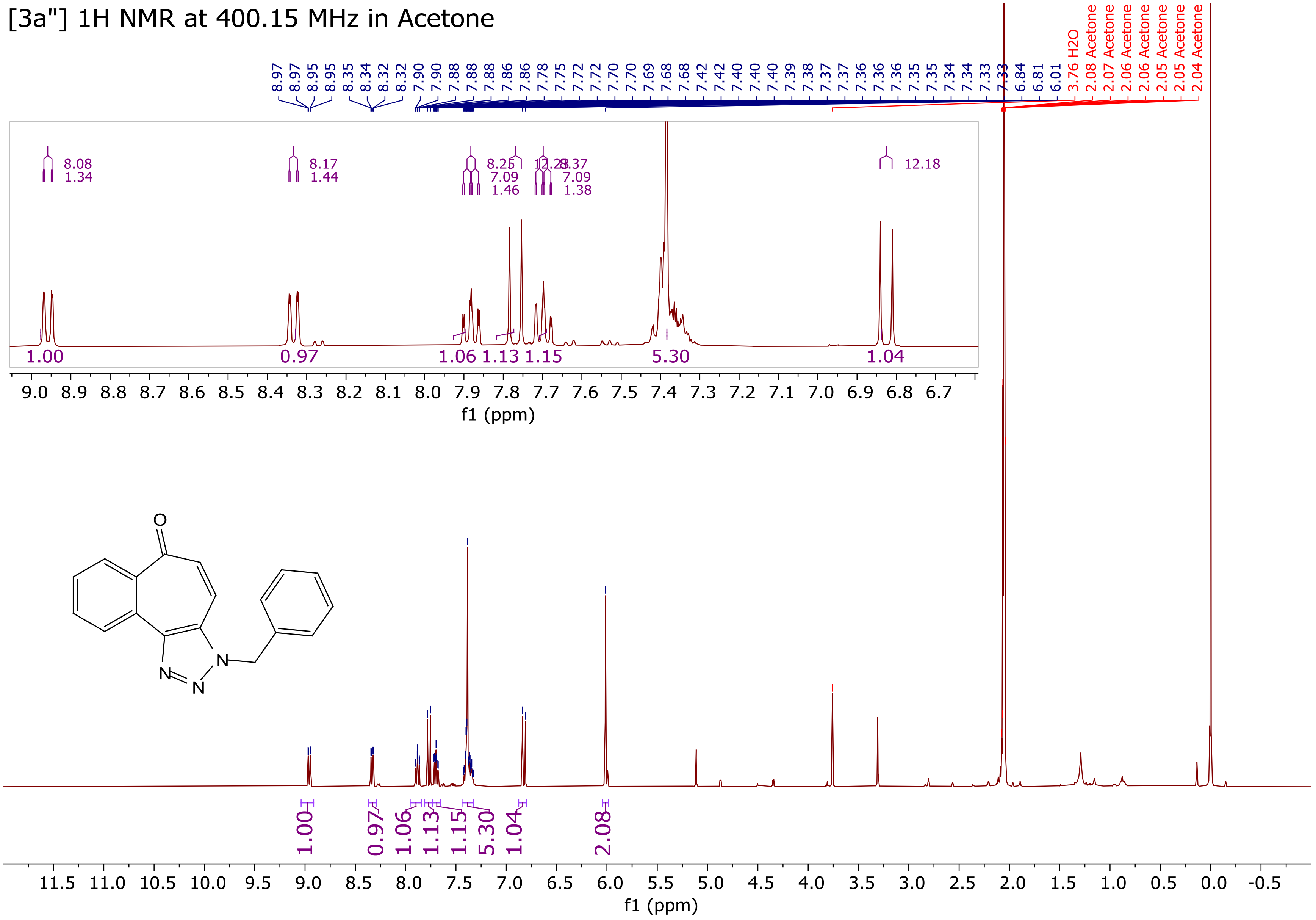

[3a"] 13C NMR at 201.27 MHz in Acetone

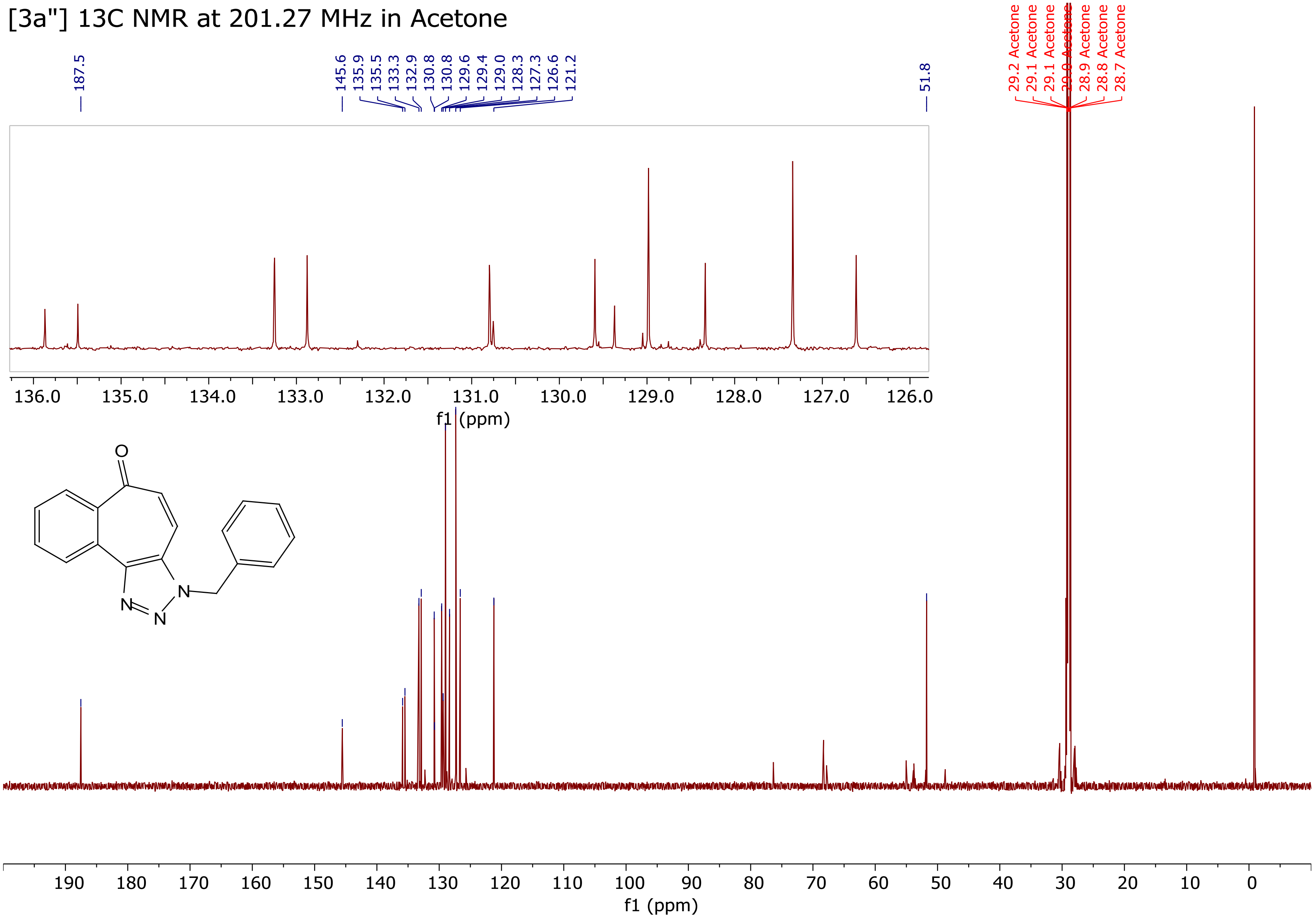

[3b] 1H NMR at 800.34 MHz in CDCl3

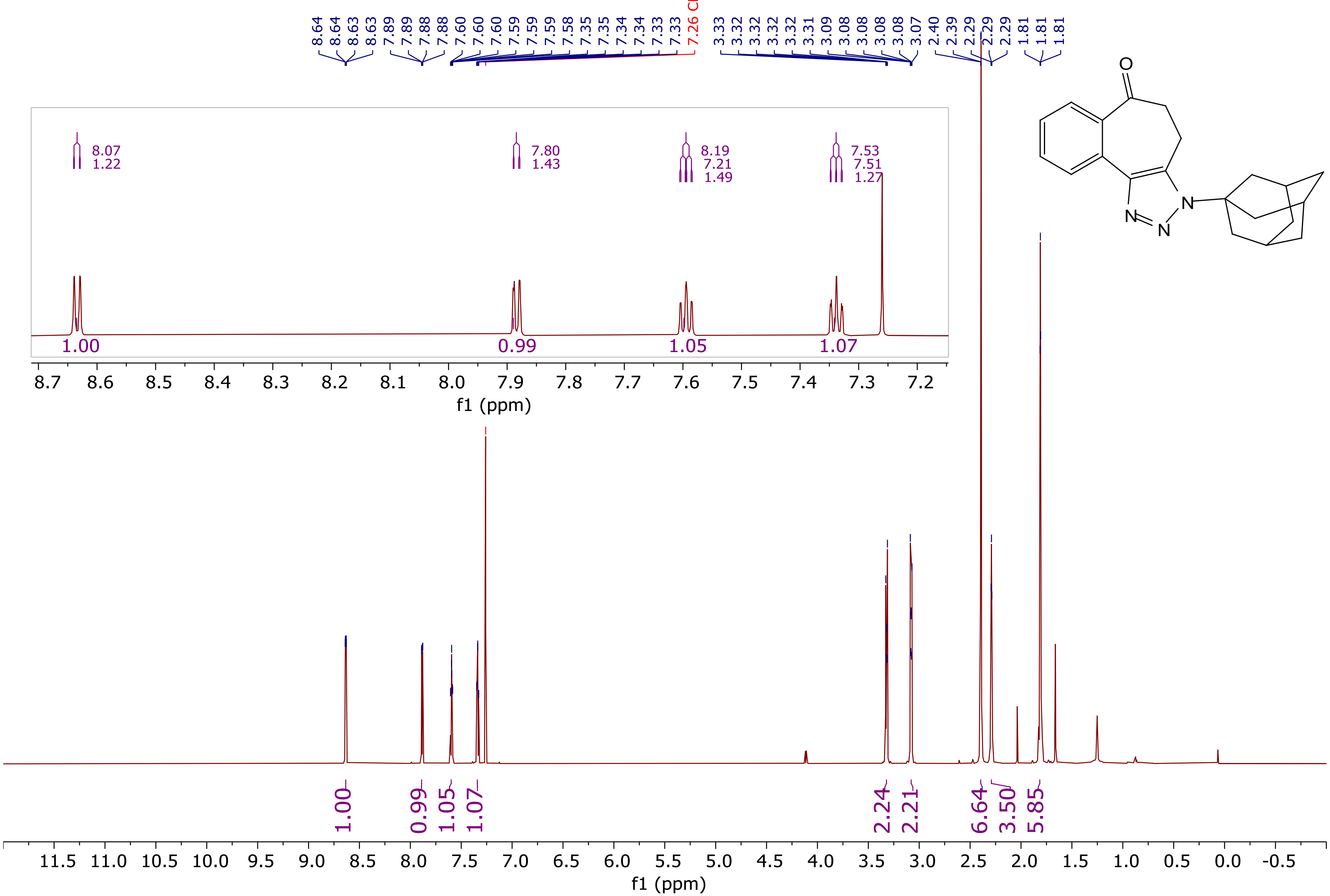

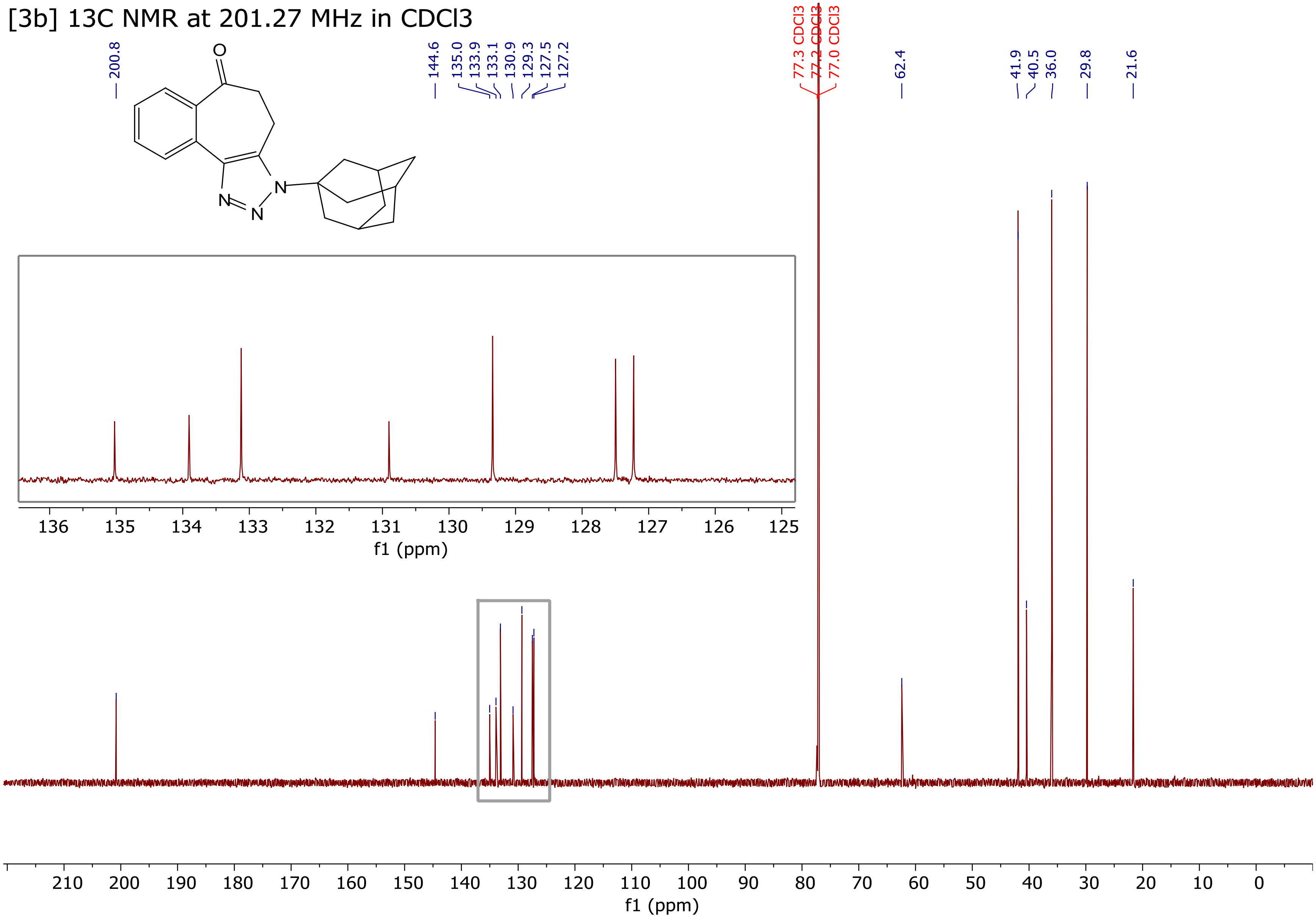

[3b] 13C NMR at 201.27 MHz in CDCl3
200.8
144.6
135.0
133.9
133.1
130.9
129.3
127.5
127.2
77.3 CDCl3
77.2 CDCl3
77.0 CDCl3
62.4
41.9
40.5
36.0
29.8
21.6
O
N
N
N
f1 (ppm)
f1 (ppm)

[3b"] 1H NMR at 800.34 MHz in CDCl3

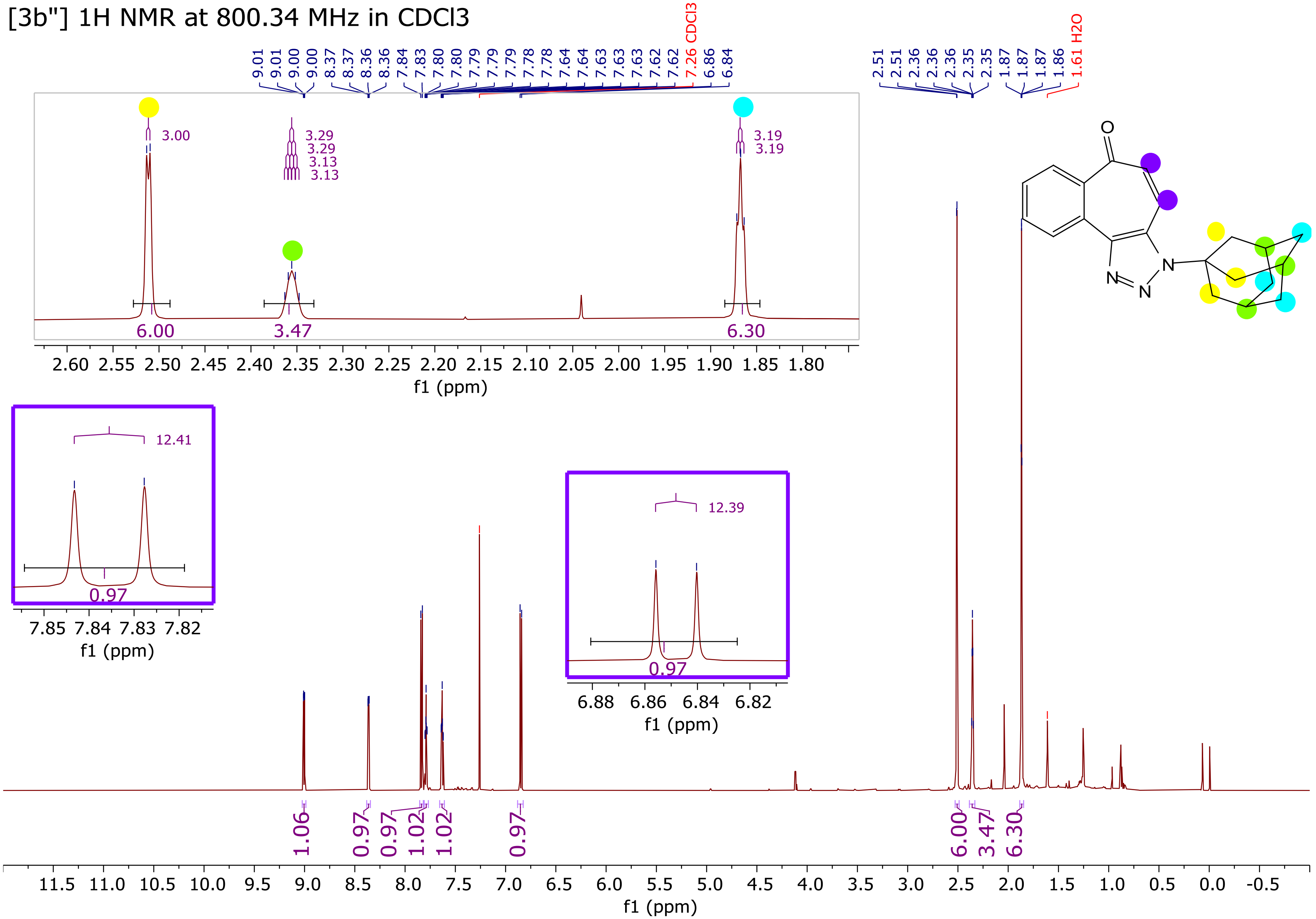

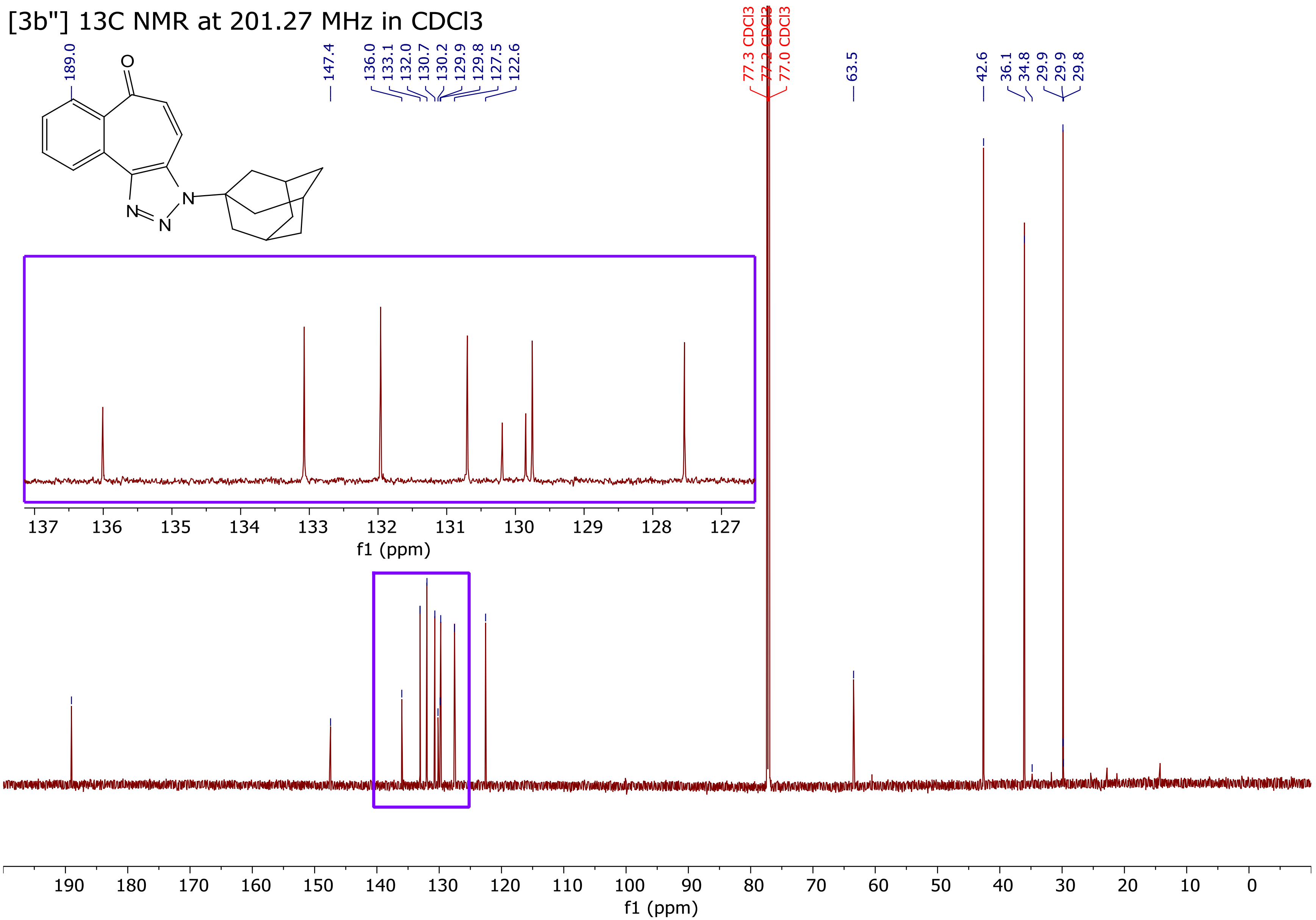
[3b"] 13C NMR at 201.27 MHz in CDCl3
189.0
147.4
136.0
133.1
132.0
130.7
130.2
129.9
129.8
127.5
122.6
77.3 CDCl3
77.0 CDCl3
63.5
42.6
36.1
34.8
29.9
29.9
29.8
O
N
N
N
f1 (ppm)
f1 (ppm)

[3c] 1H NMR at 800.34 MHz in CD3CN

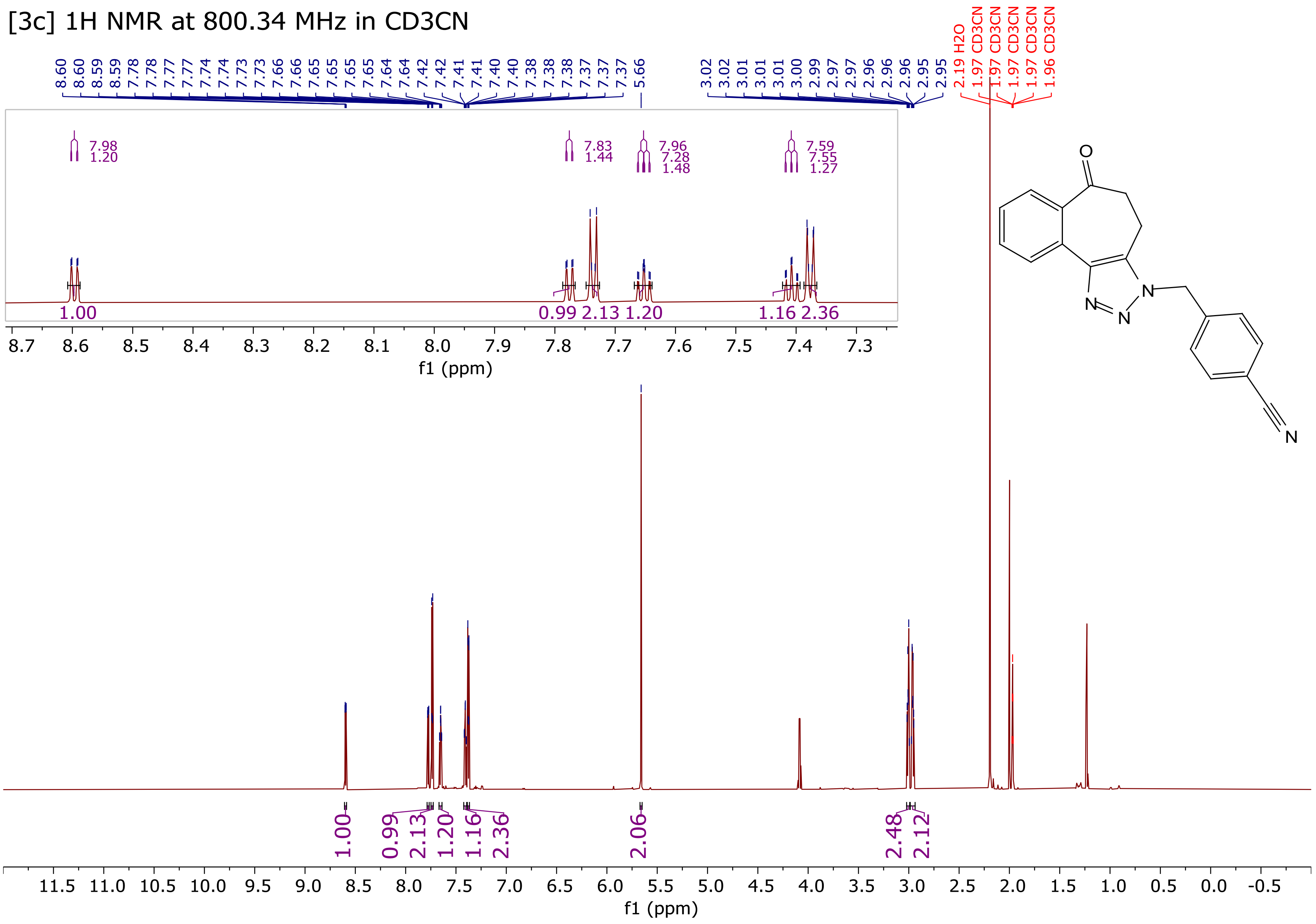

[3c] 13C NMR at 201.27 MHz in CD3CN

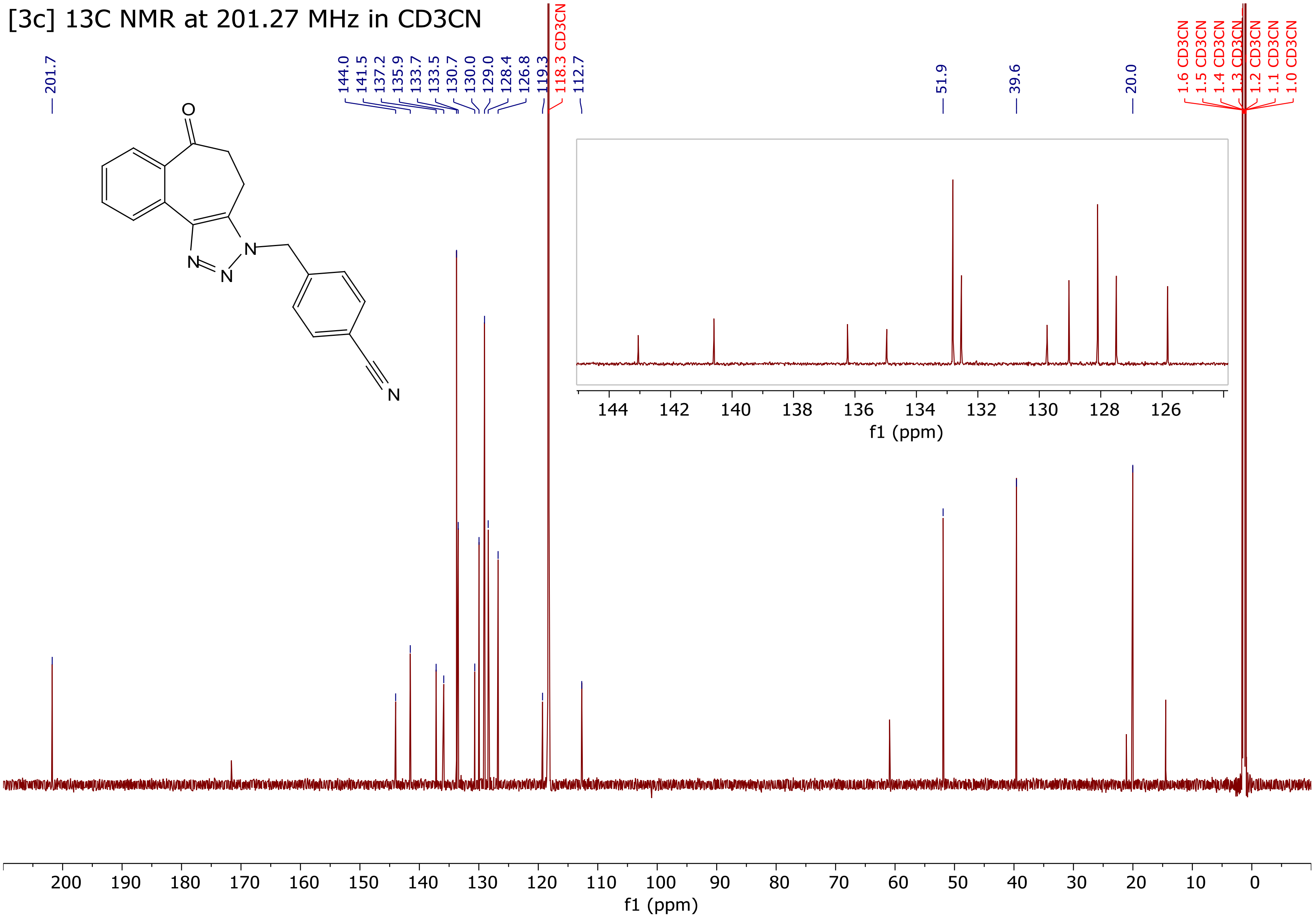

[3d] 1H NMR at 400.15 MHz in CD3CN

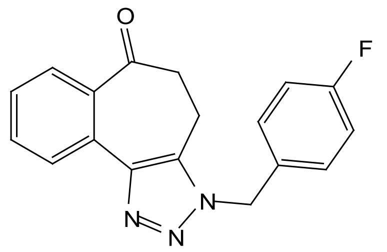

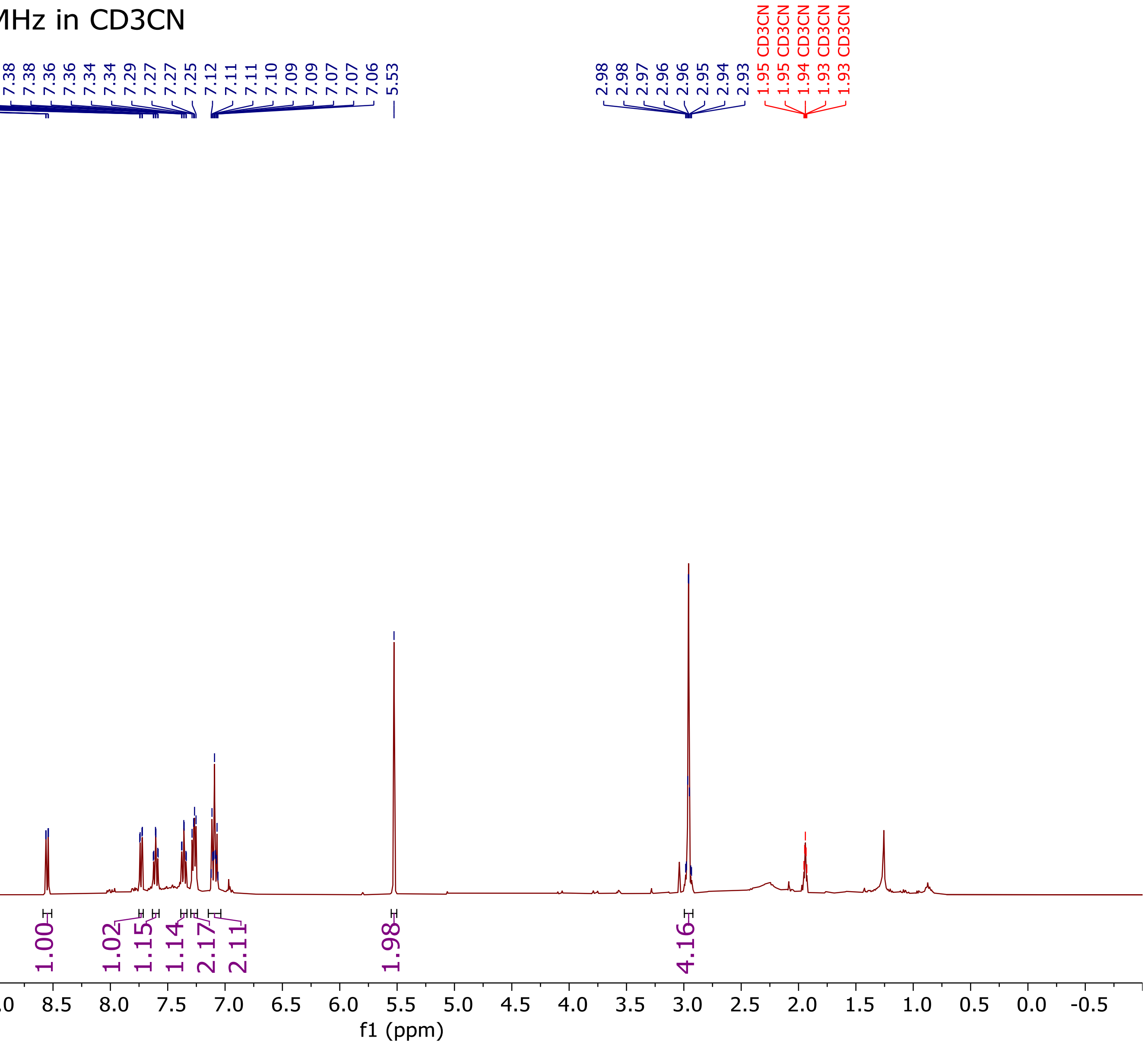

[3d] 13C NMR at 100.63 MHz in CD3CN

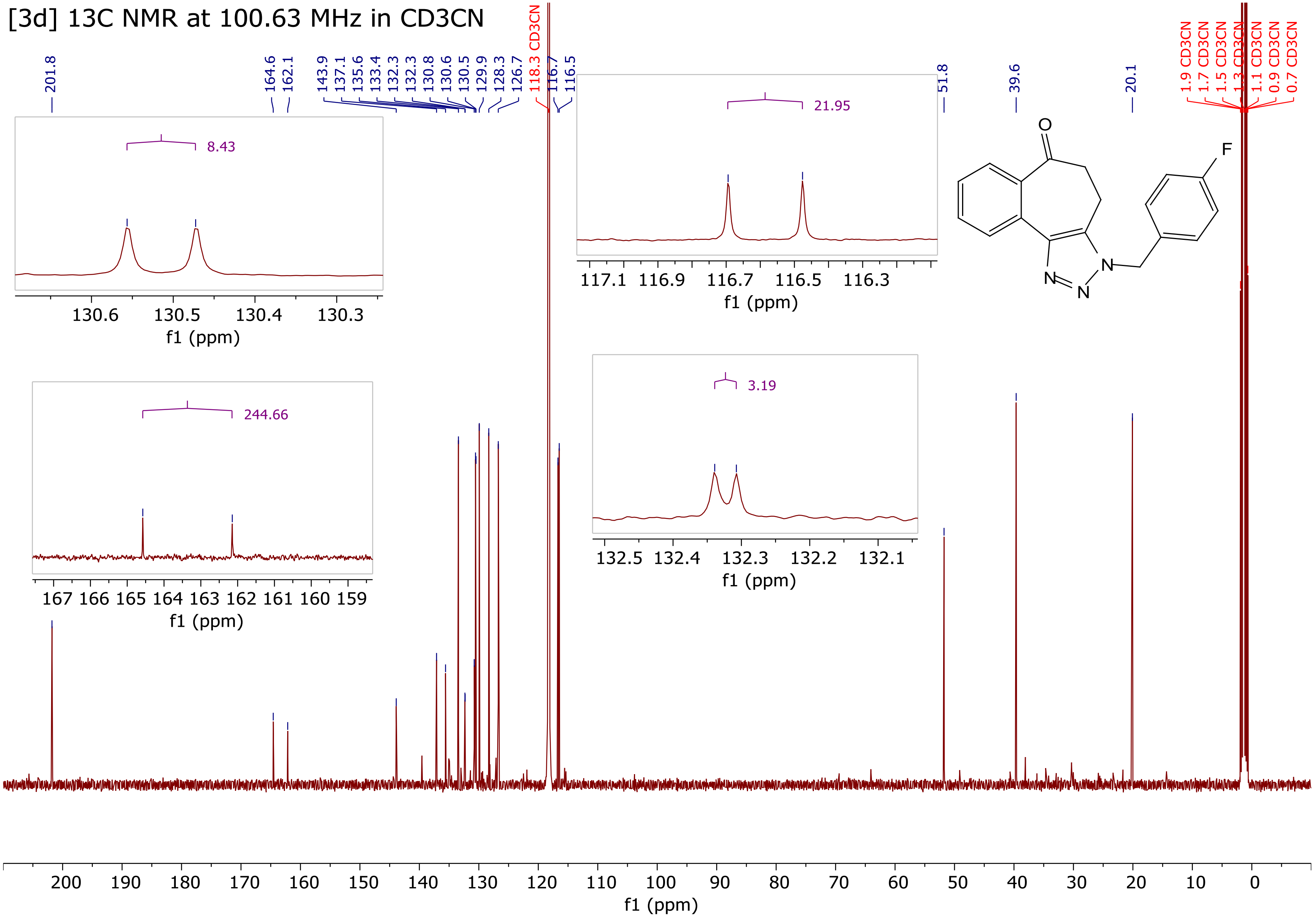

[3d] 19F NMR at 376.48 MHz in CD3CN

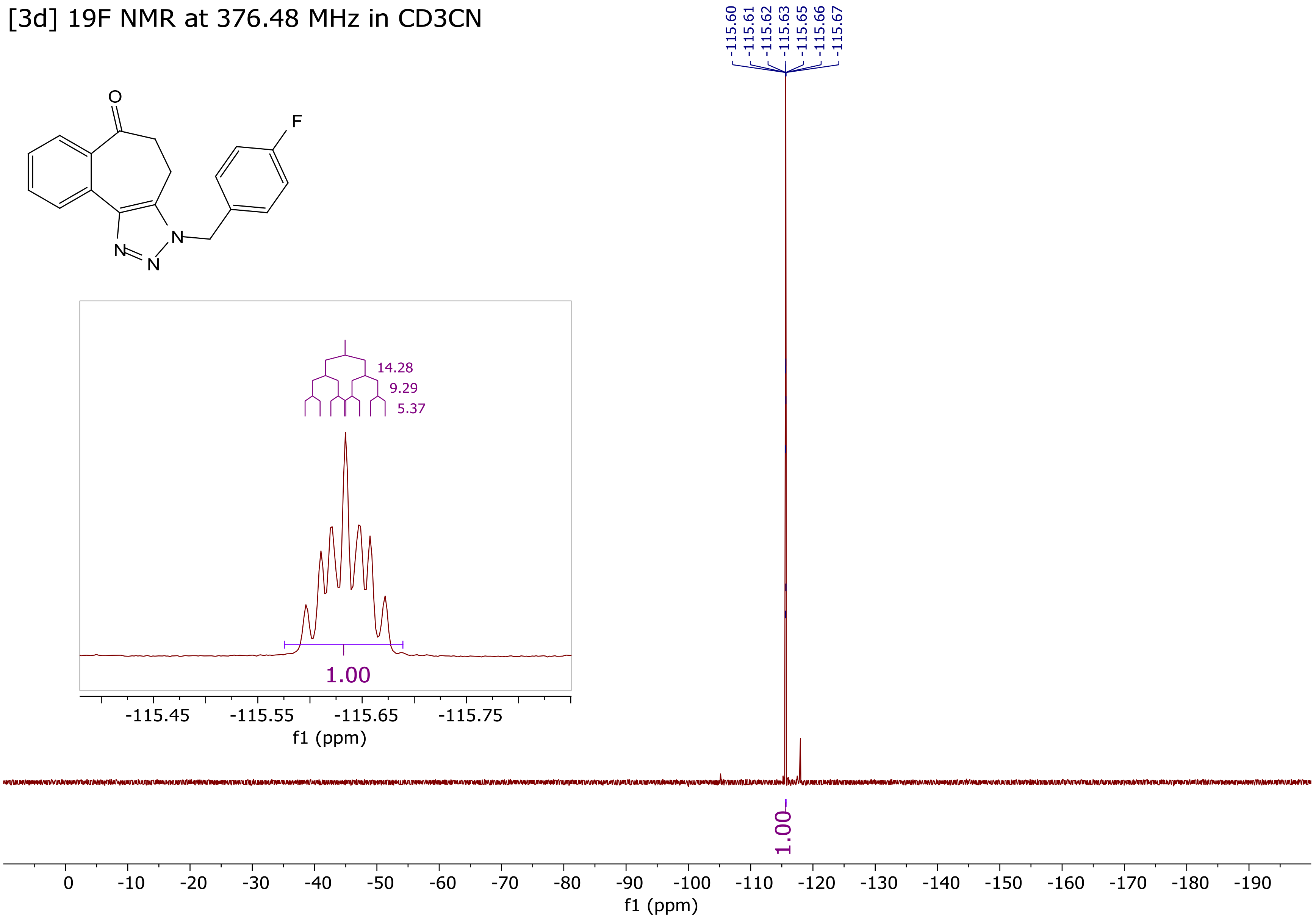

[3e] 1H NMR at 800.34 MHz in CDCl3

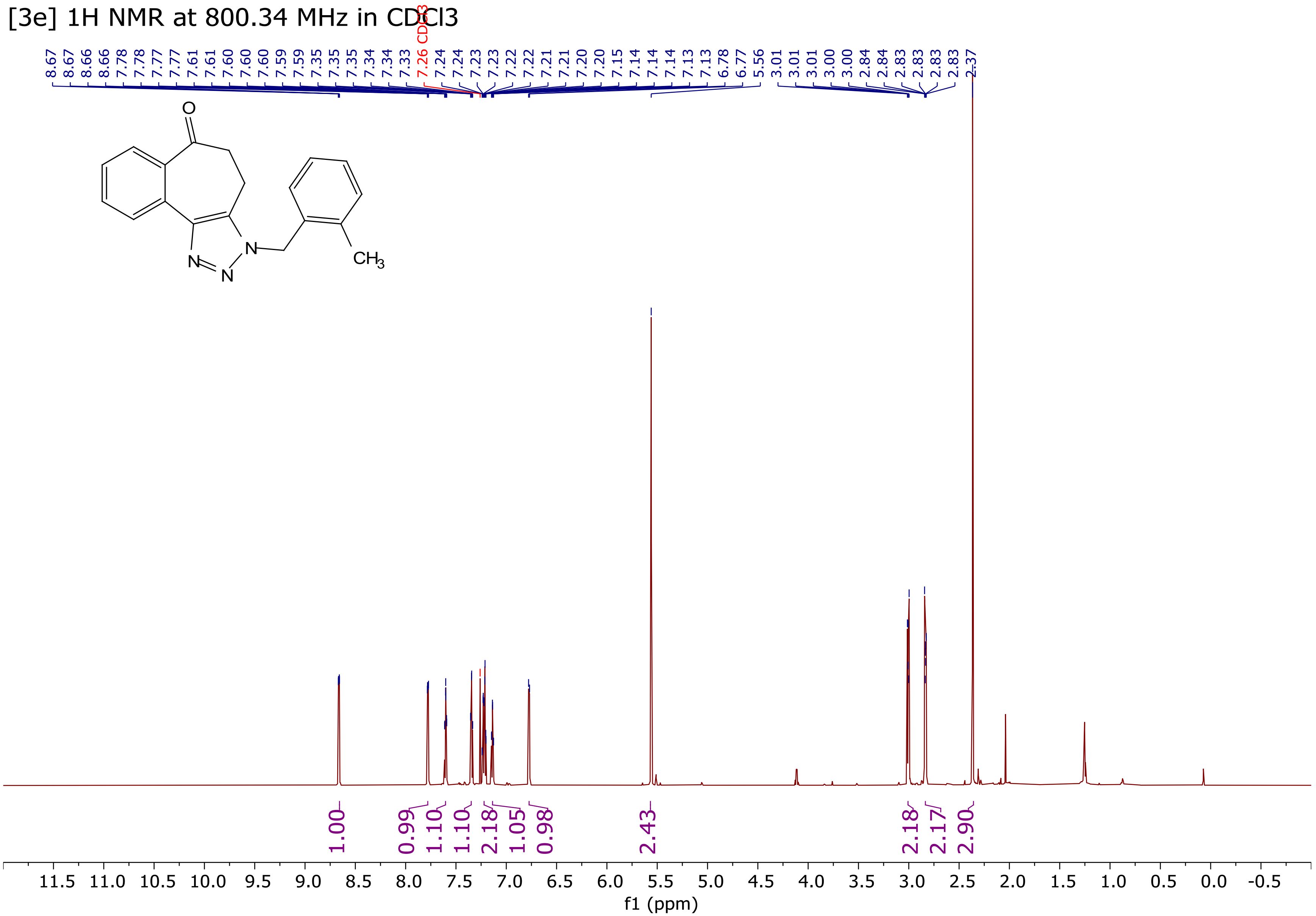

[3e] 13C NMR at 201.27 MHz in CDCl3

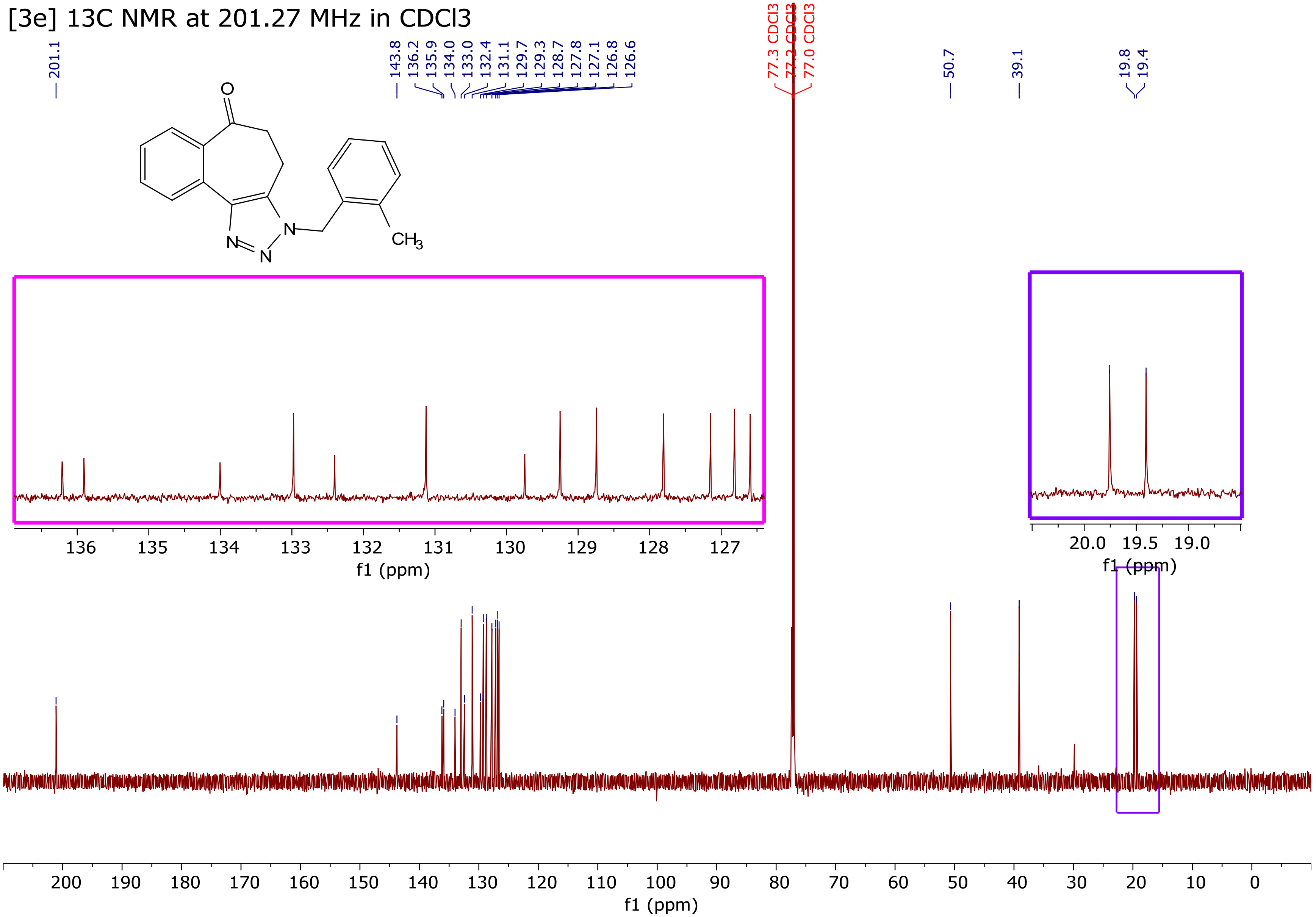

[3e"] 1H NMR at 400.15 MHz in Acetone

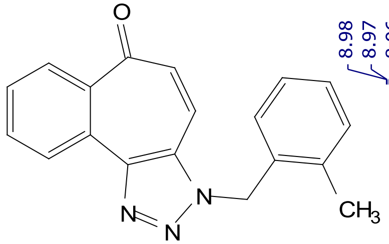

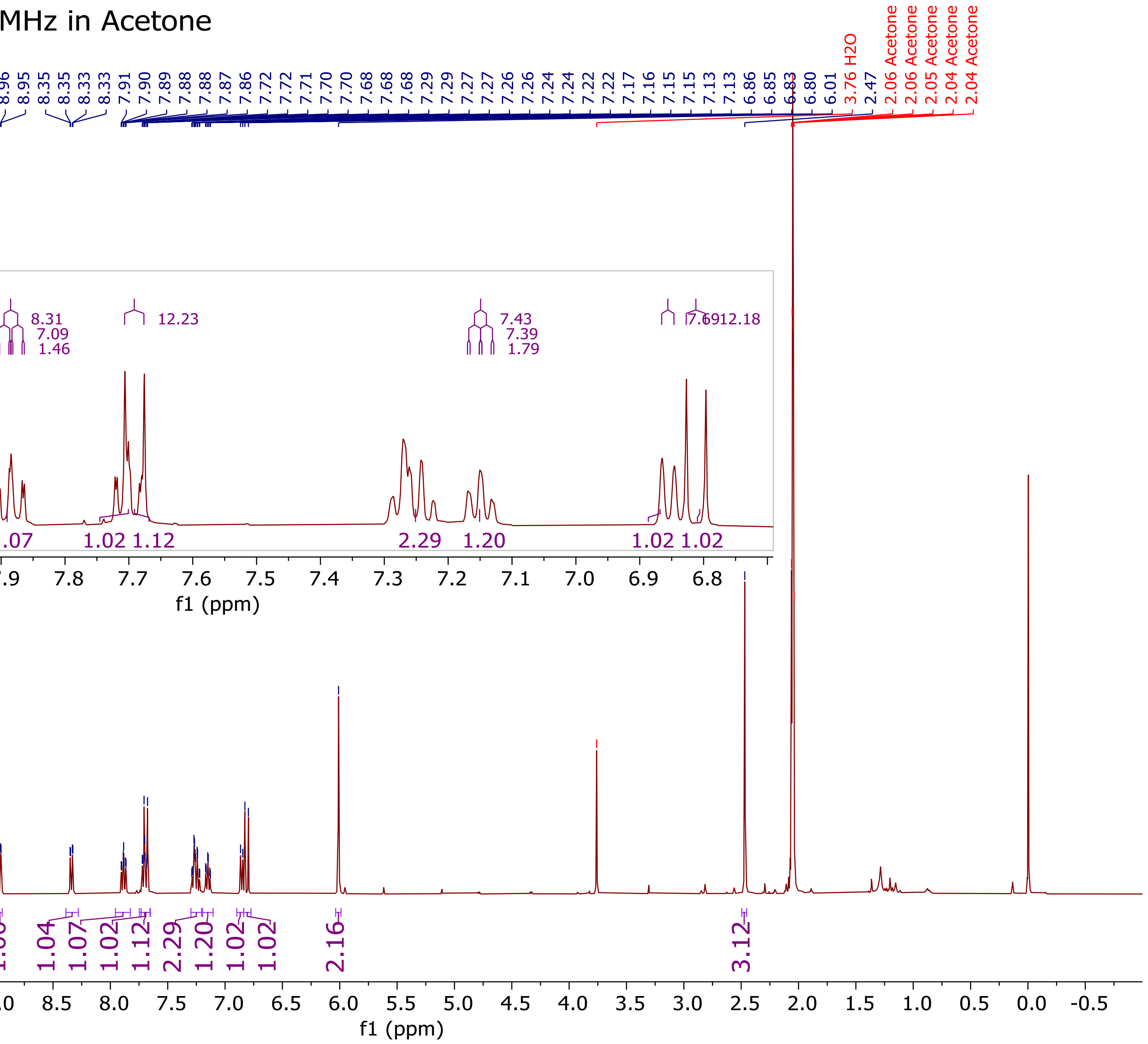

[3e"] 13C NMR at 201.27 MHz in Acetone

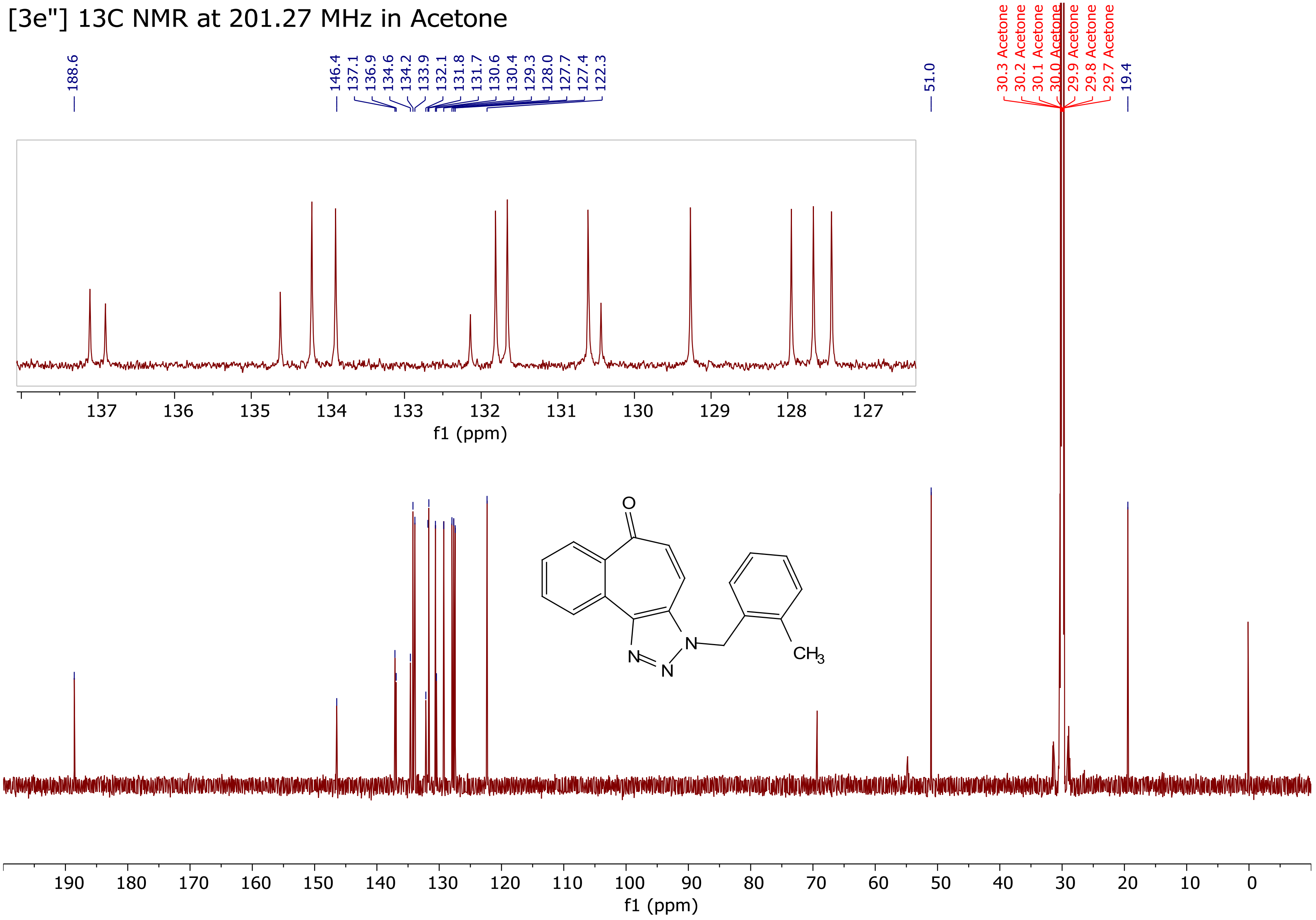

[3f] 1H NMR at 800.34 MHz in CDCl3

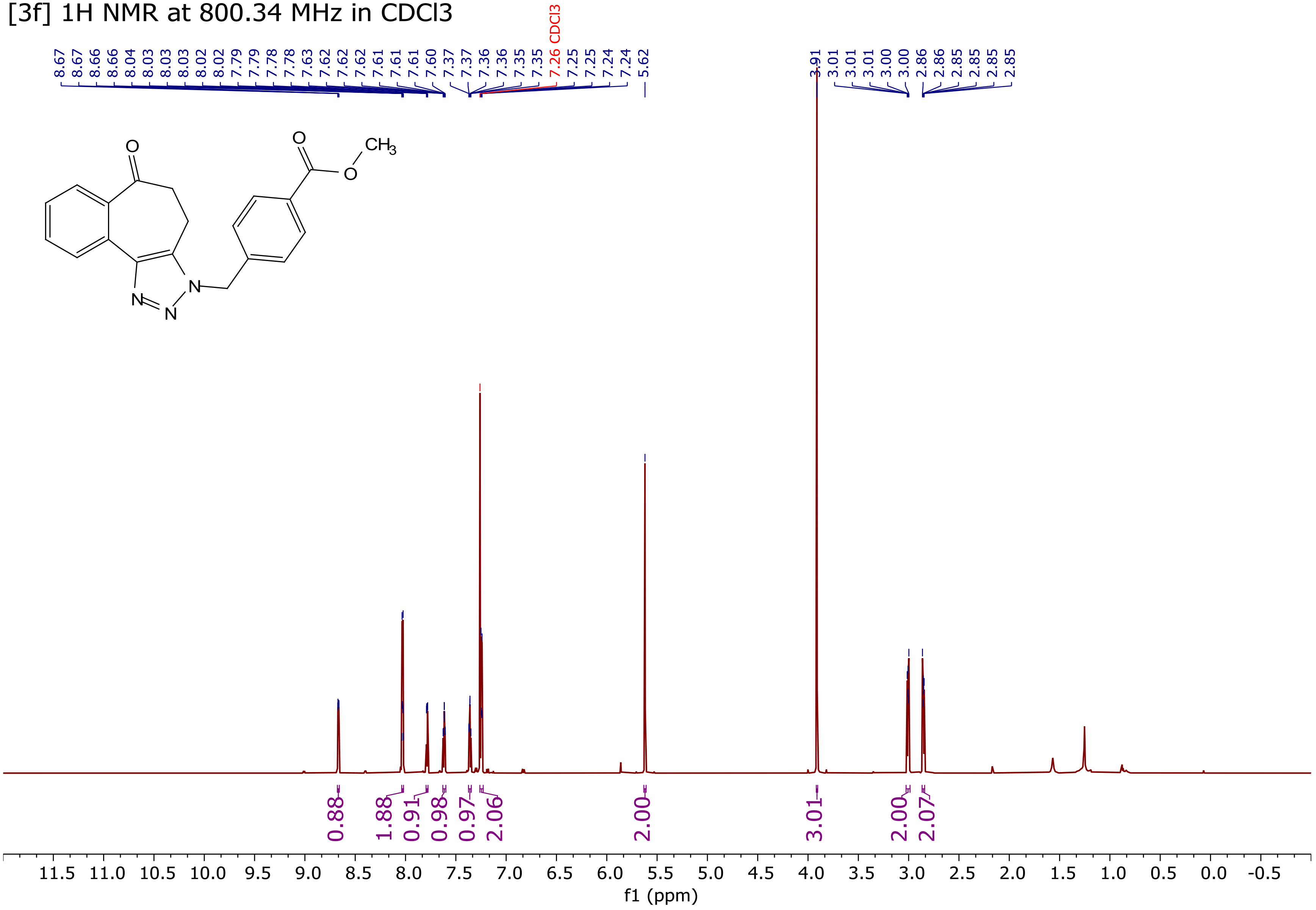

[3f] 13C NMR at 201.27 MHz in CDCl3

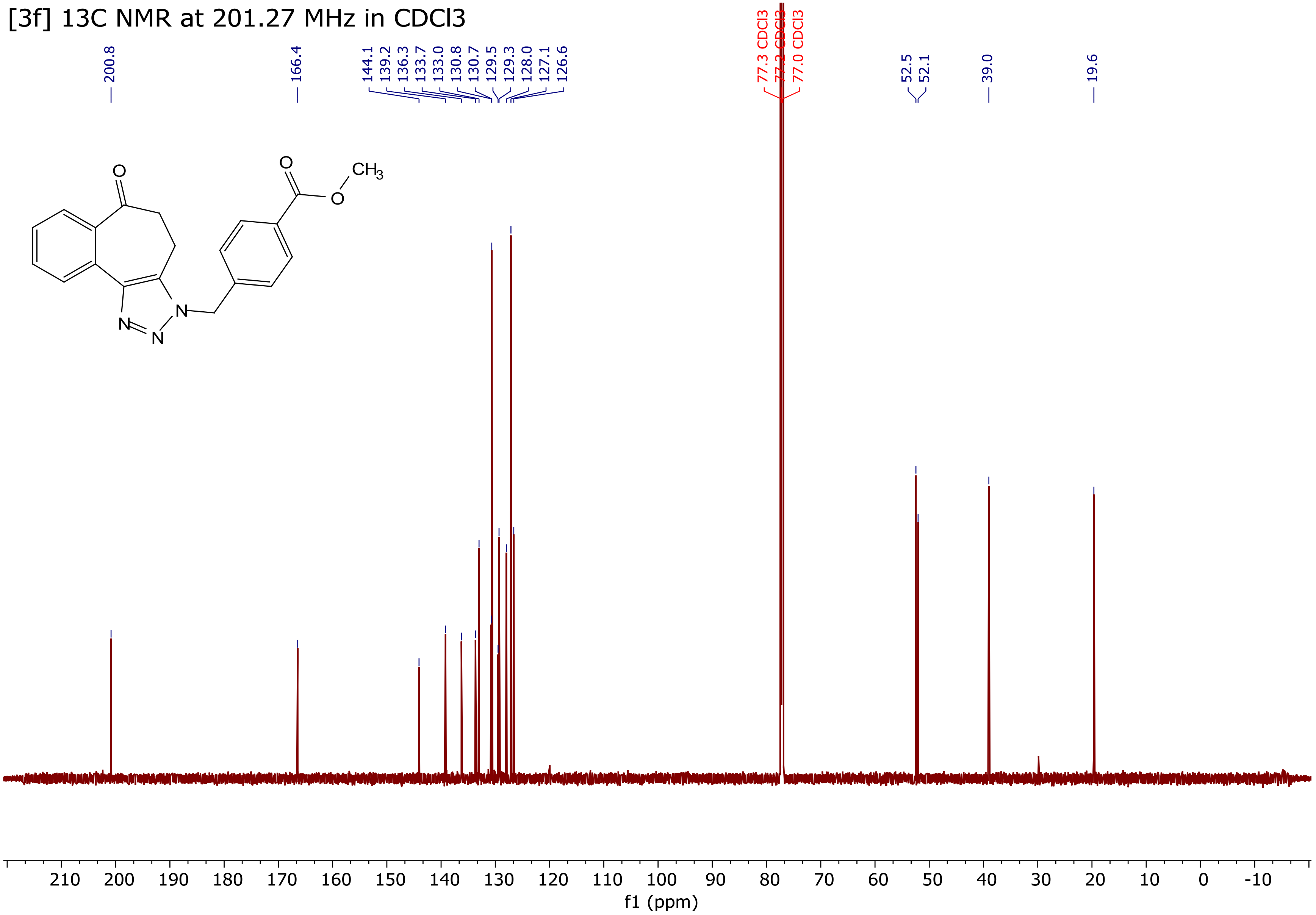

[3g] 1H NMR at 400.15 MHz in CD3CN

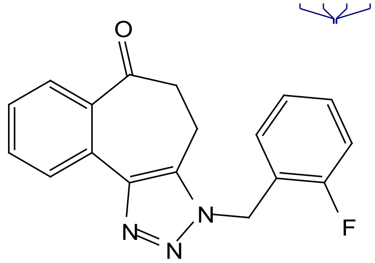

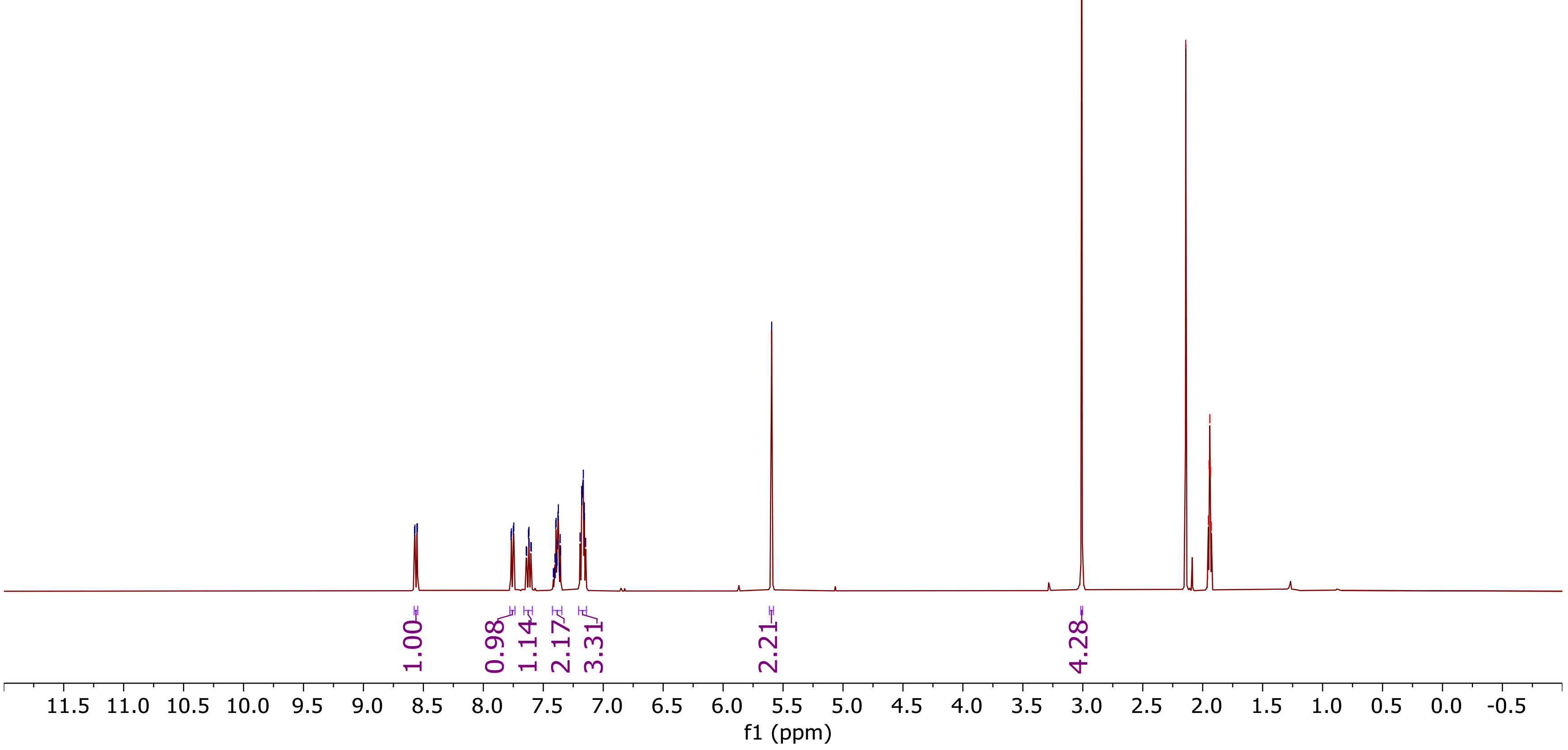

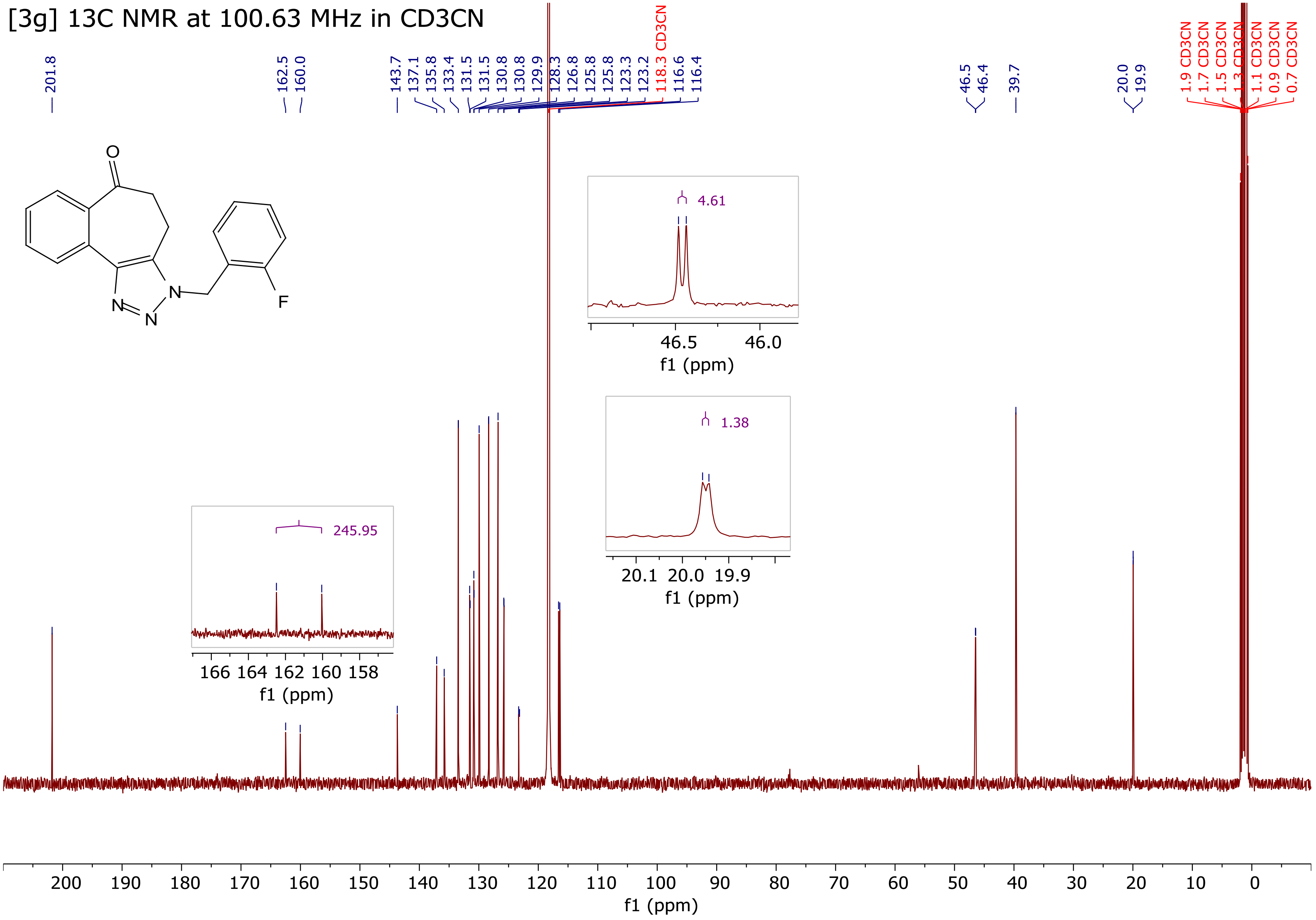
[3g] 13C NMR at 100.63 MHz in CD3CN
201.8
162.5
160.0
143.7
137.1
135.8
133.4
131.5
131.5
130.8
130.8
129.9
128.3
126.8
125.8
125.8
123.3
123.2
118.3 CD3CN
116.6
116.4
46.5
46.4
39.7
20.0
19.9
1.9 CD3CN
1.7 CD3CN
1.5 CD3CN
1.3 CD3CN
1.1 CD3CN
0.9 CD3CN
0.7 CD3CN
O
N
N
N
F
4.61
46.5
46.0
f1 (ppm)
1.38
20.1
20.0
19.9
f1 (ppm)
245.95
166
164
162
160
158
f1 (ppm)
200
190
180
170
160
150
140
130
120
110
100
90
80
70
60
50
40
30
20
10
0
f1 (ppm)

[3g] 19F NMR at 376.48 MHz in CDCl3

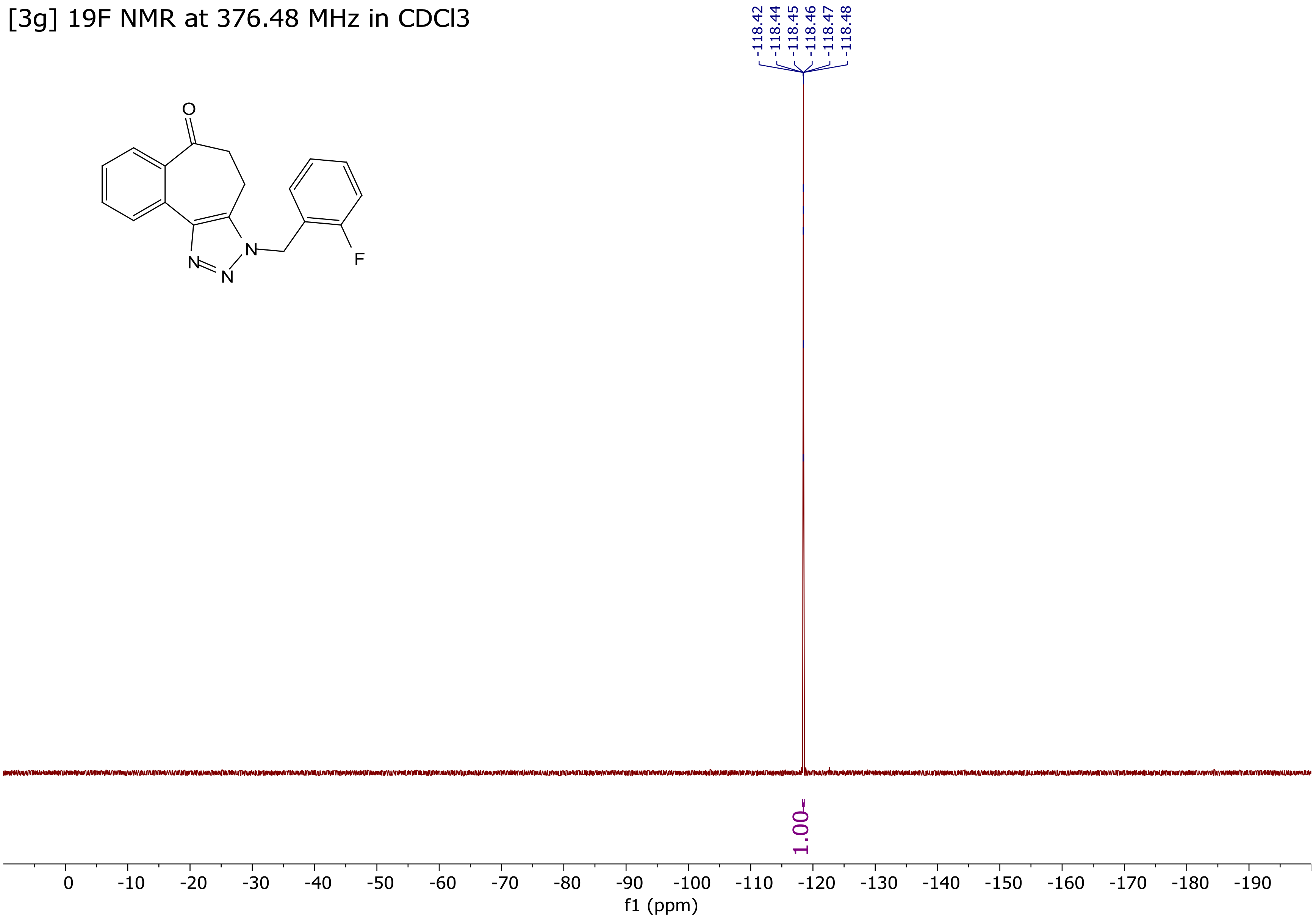

[3g"] 1H NMR at 400.15 MHz in Acetone

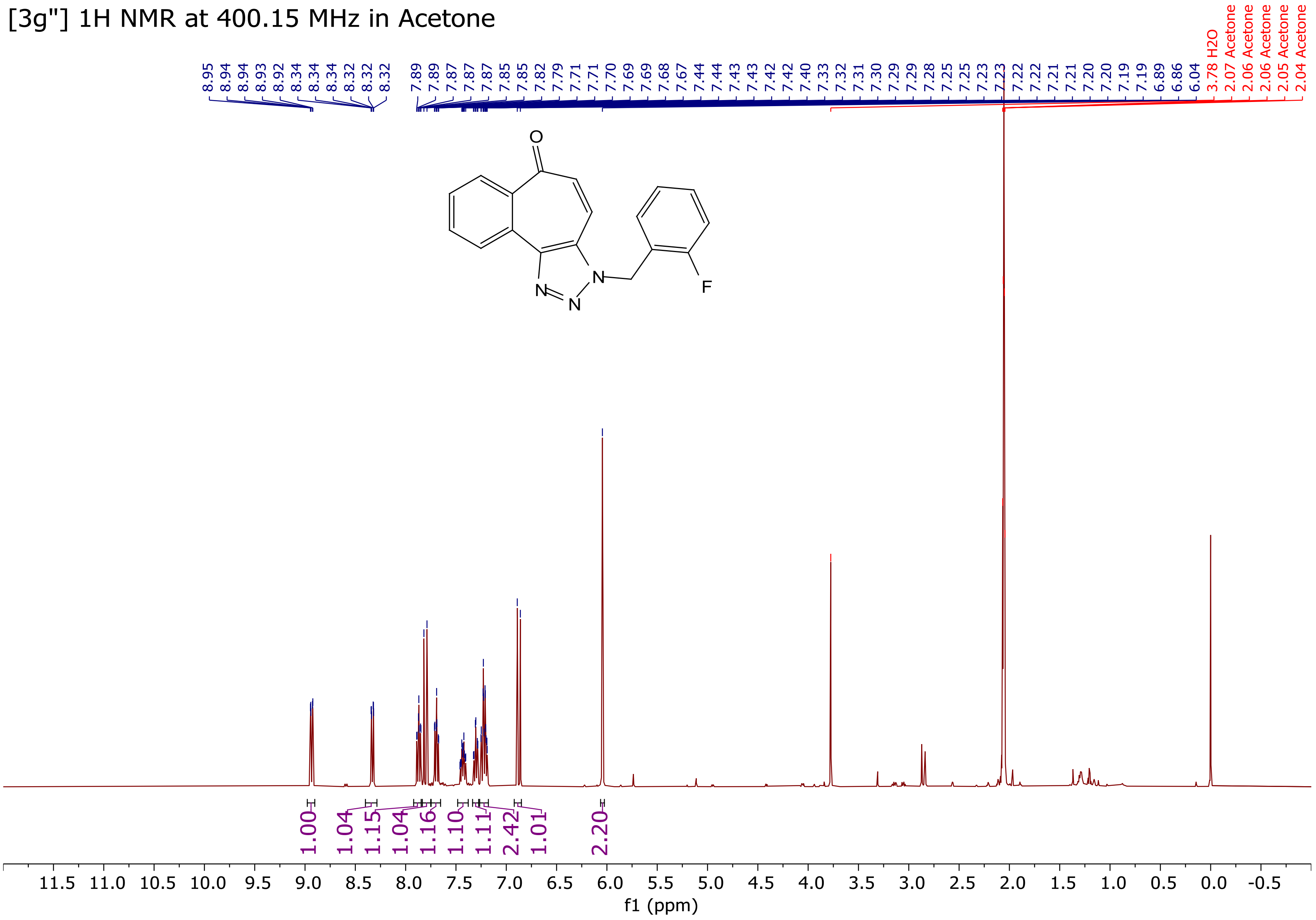

[3g"] 13C NMR at 201.27 MHz in Acetone

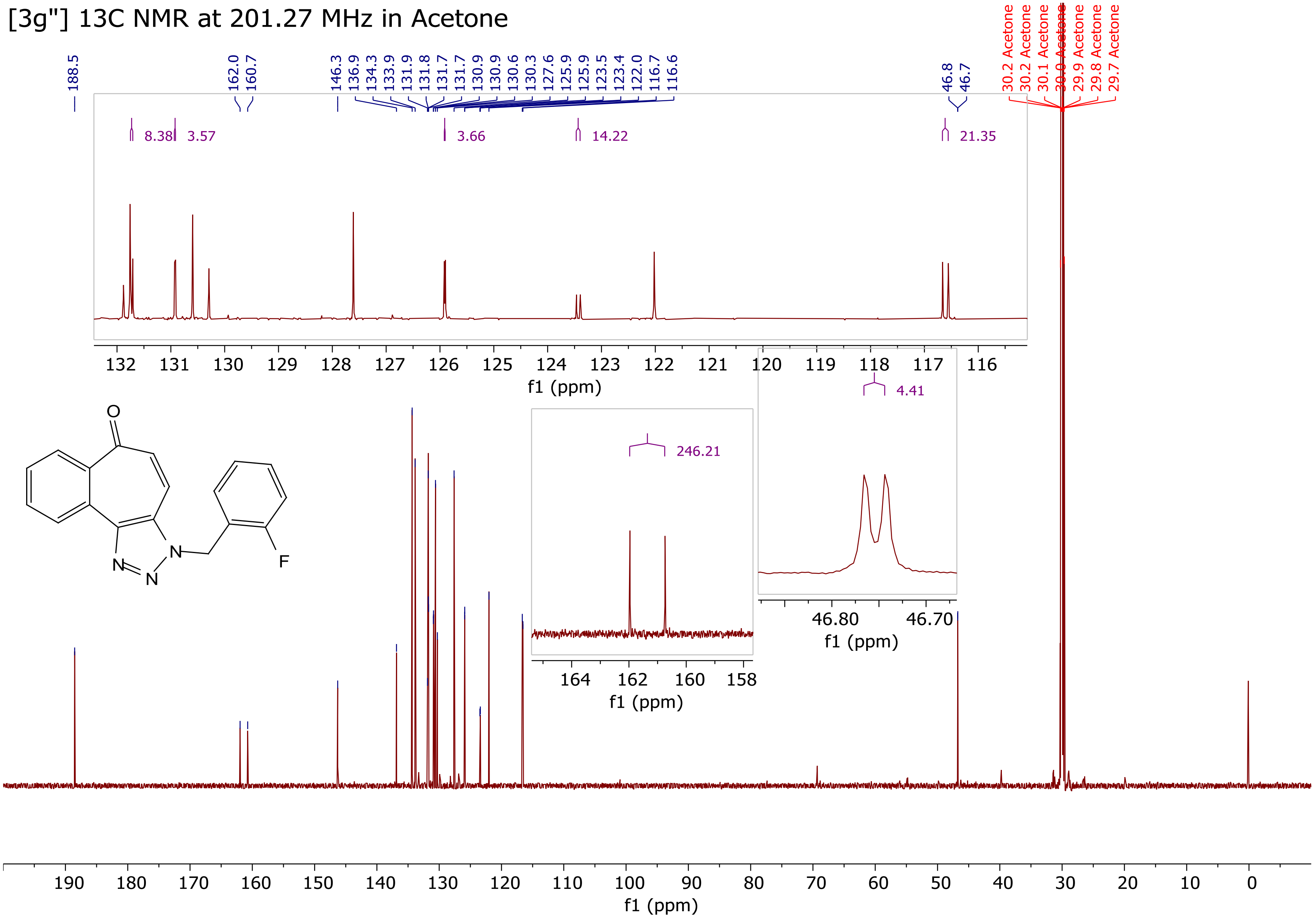

[3g"] 19F NMR at 376.48 MHz in Acetone

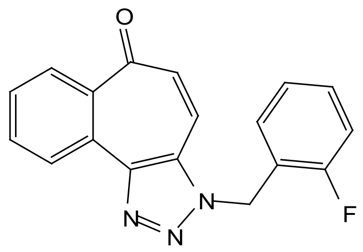

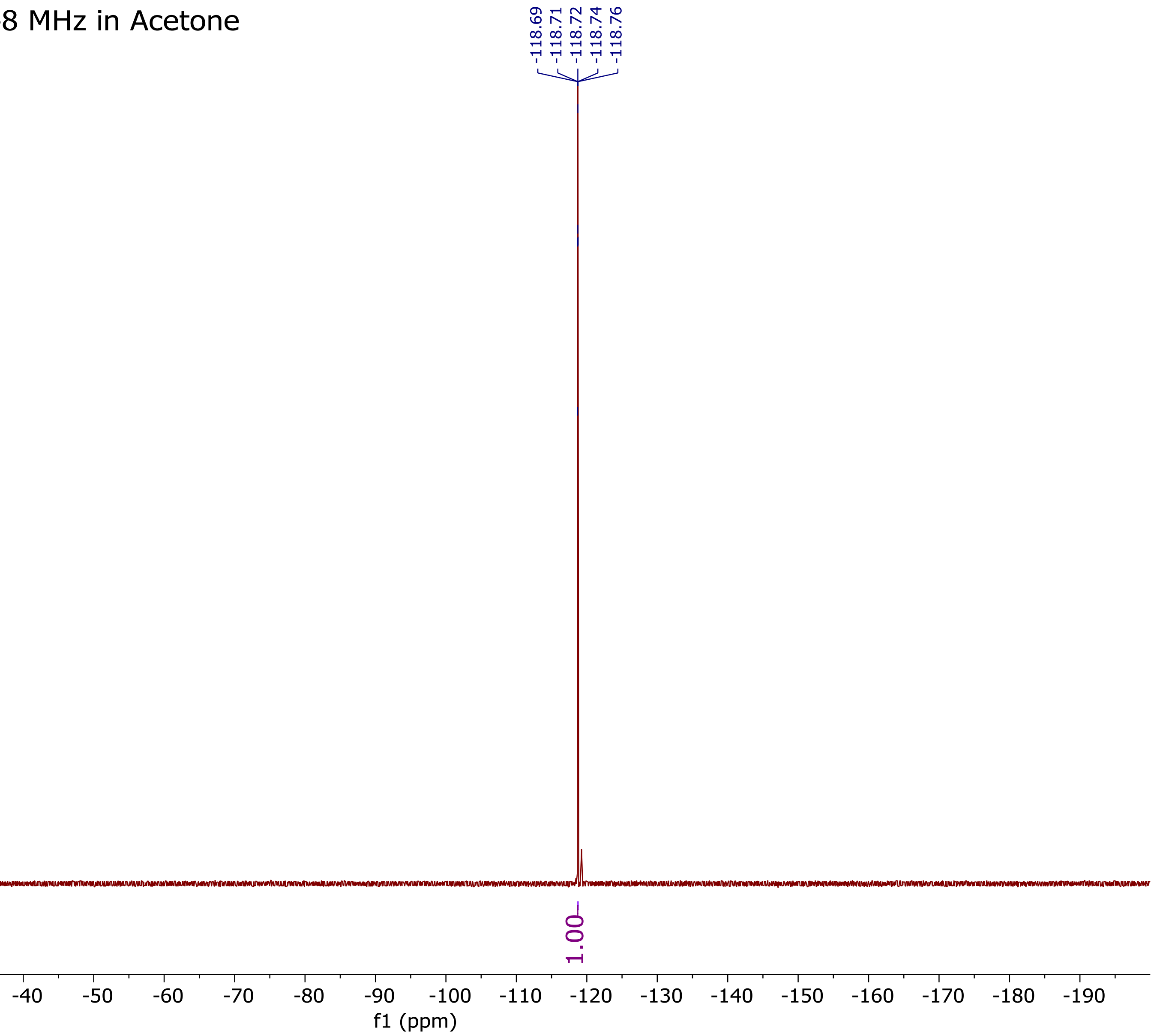

[3h] 1H NMR at 800.34 MHz in CDCl3

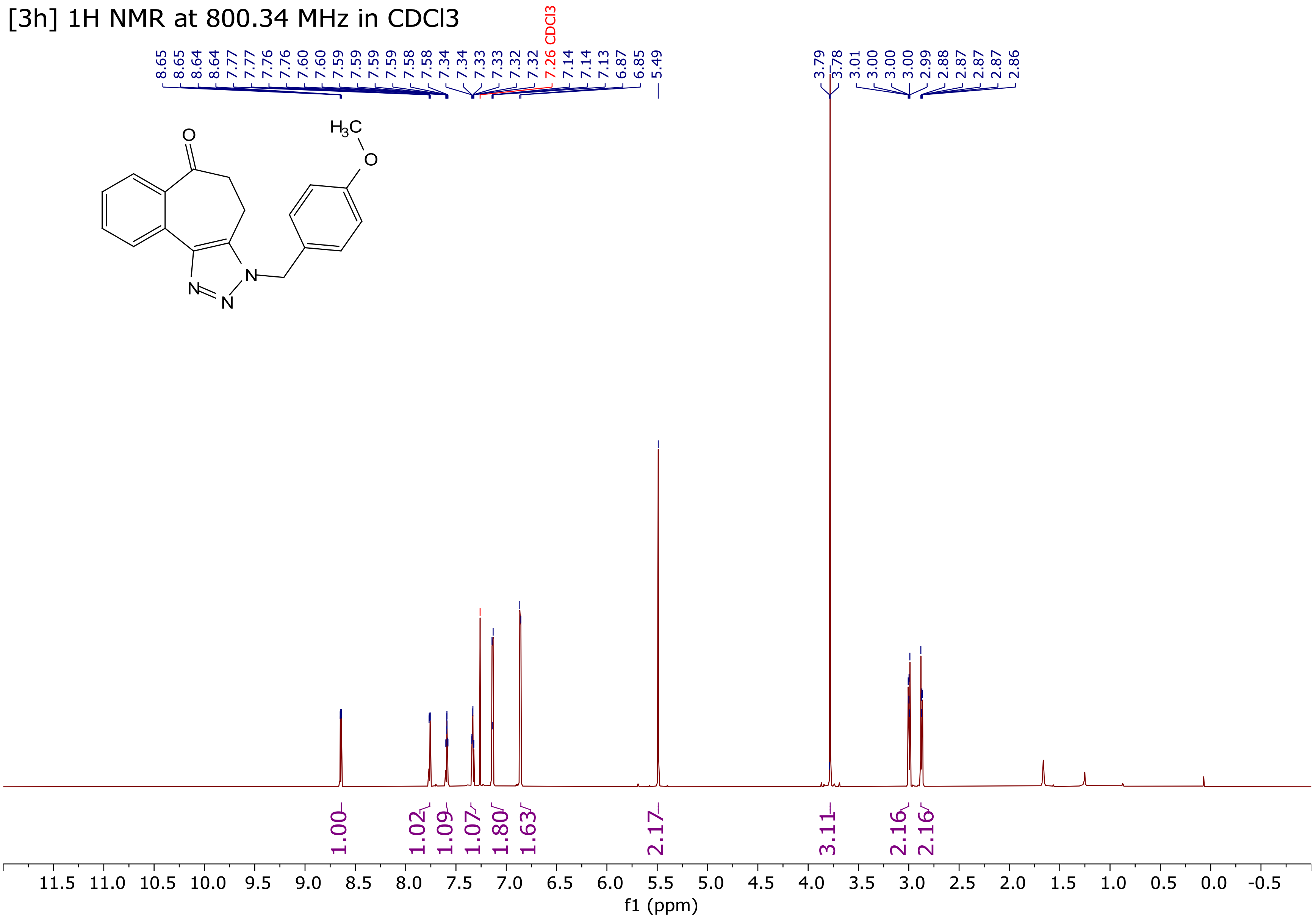

[3h] 13C NMR at 201.27 MHz in CDCl3

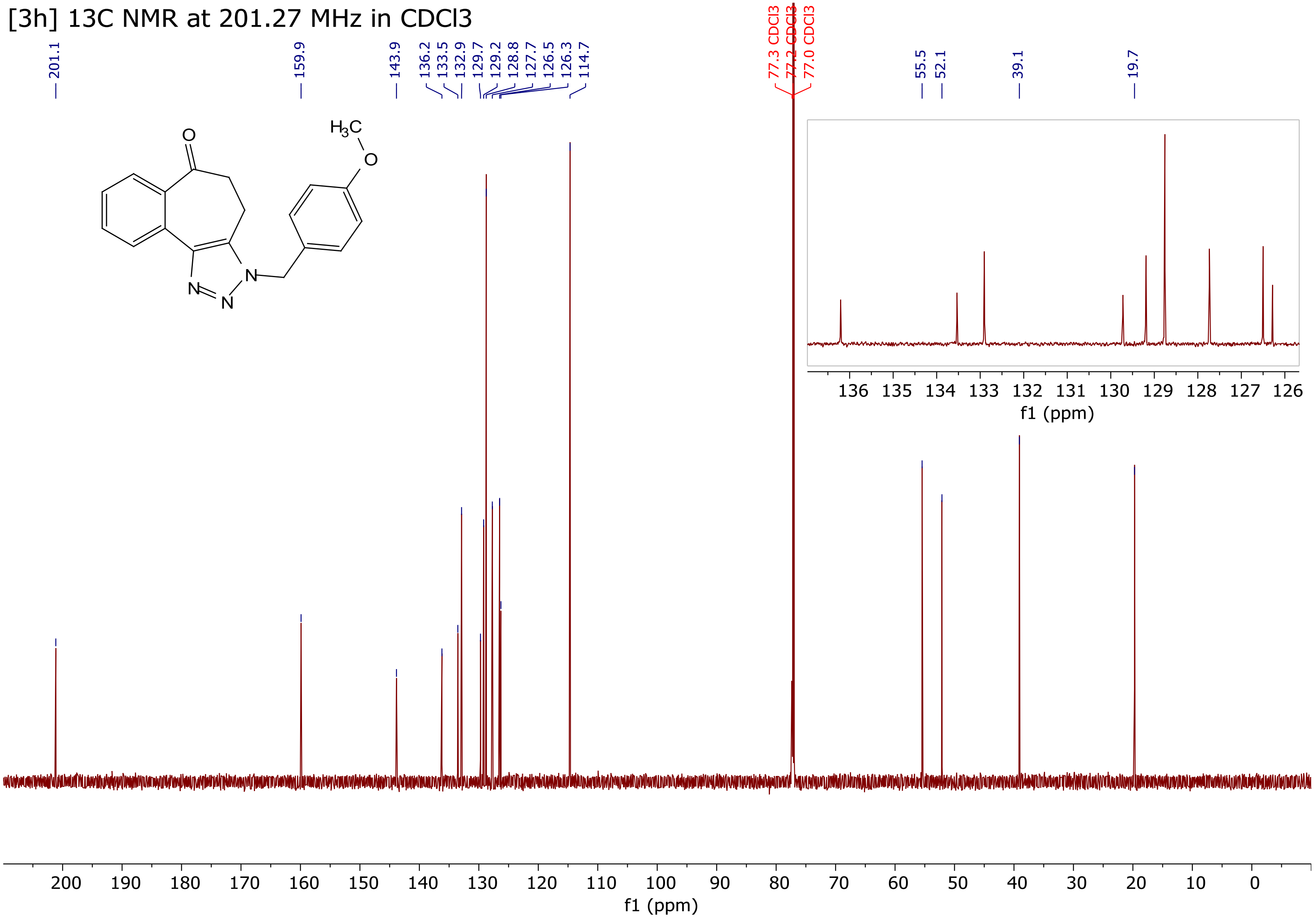

[3i] 1H NMR at 400.15 MHz in CDCl3

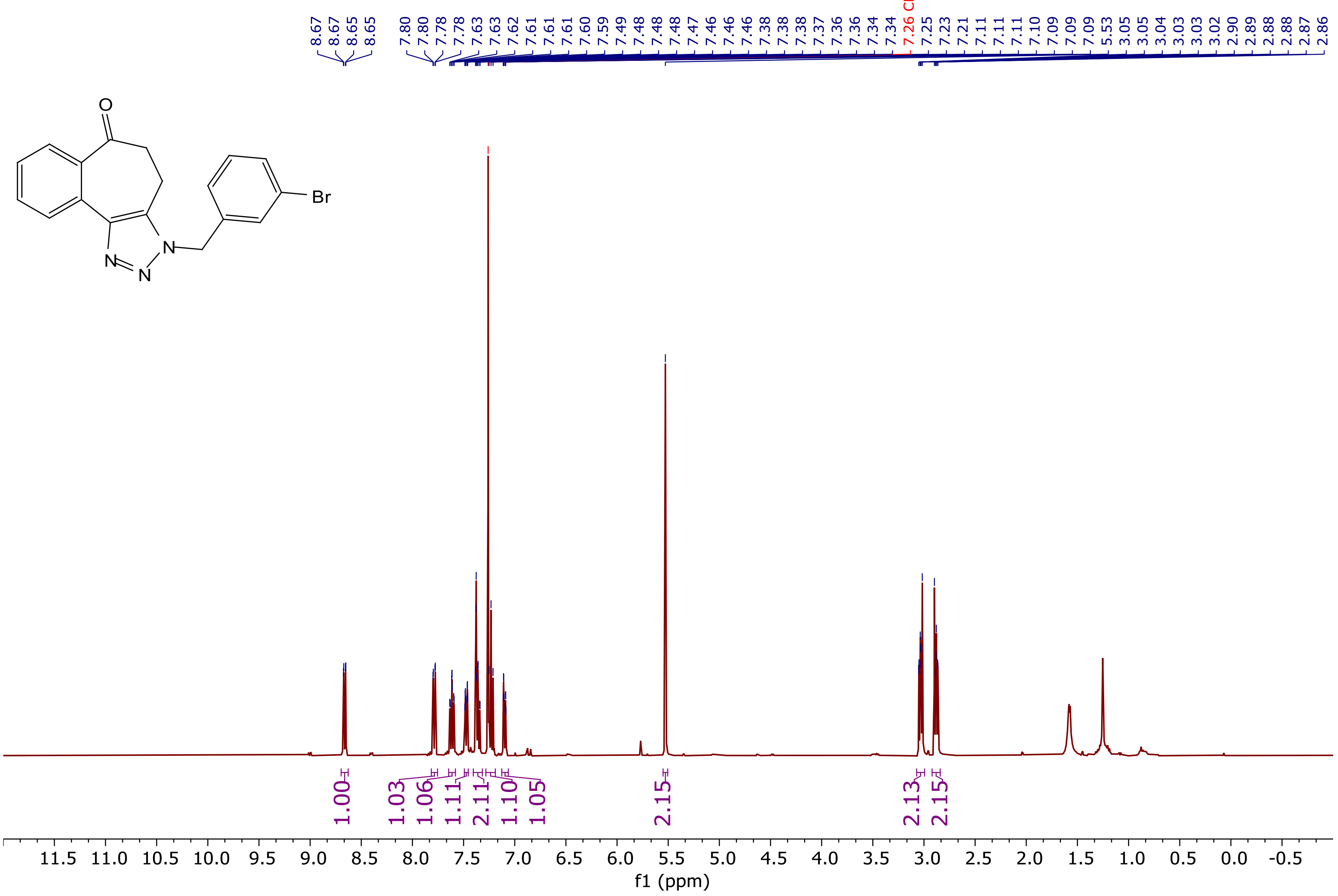

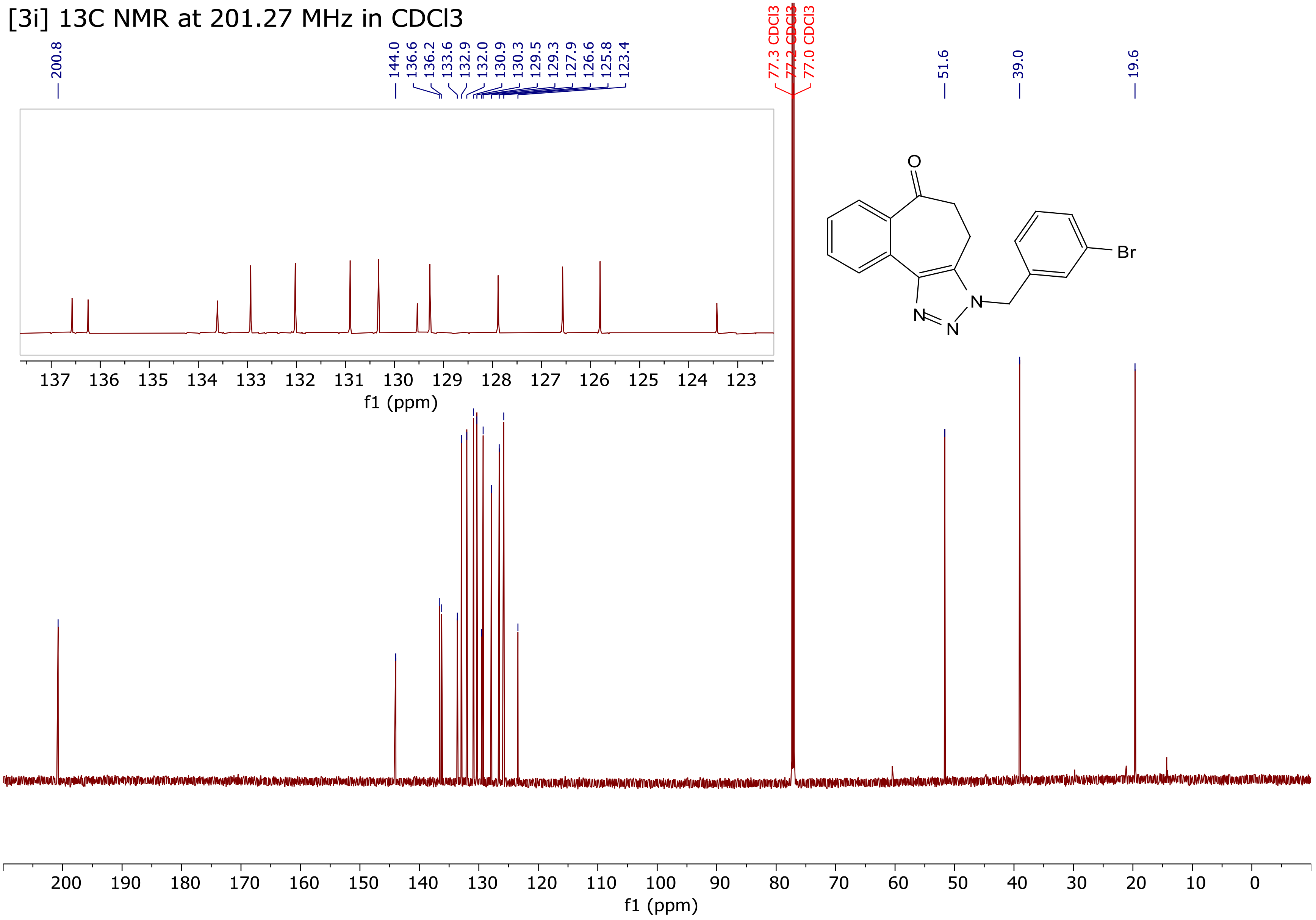
[3i] 13C NMR at 201.27 MHz in CDCl3
200.8
144.0
136.6
136.2
133.6
132.9
132.0
130.9
130.3
129.5
129.3
127.9
126.6
125.8
123.4
77.3 CDCl3
77.1 CDCl3
77.0 CDCl3
51.6
39.0
19.6
O
N
N
N
Br
f1 (ppm)
f1 (ppm)

[3j] 1H NMR at 400.15 MHz in CDCl3

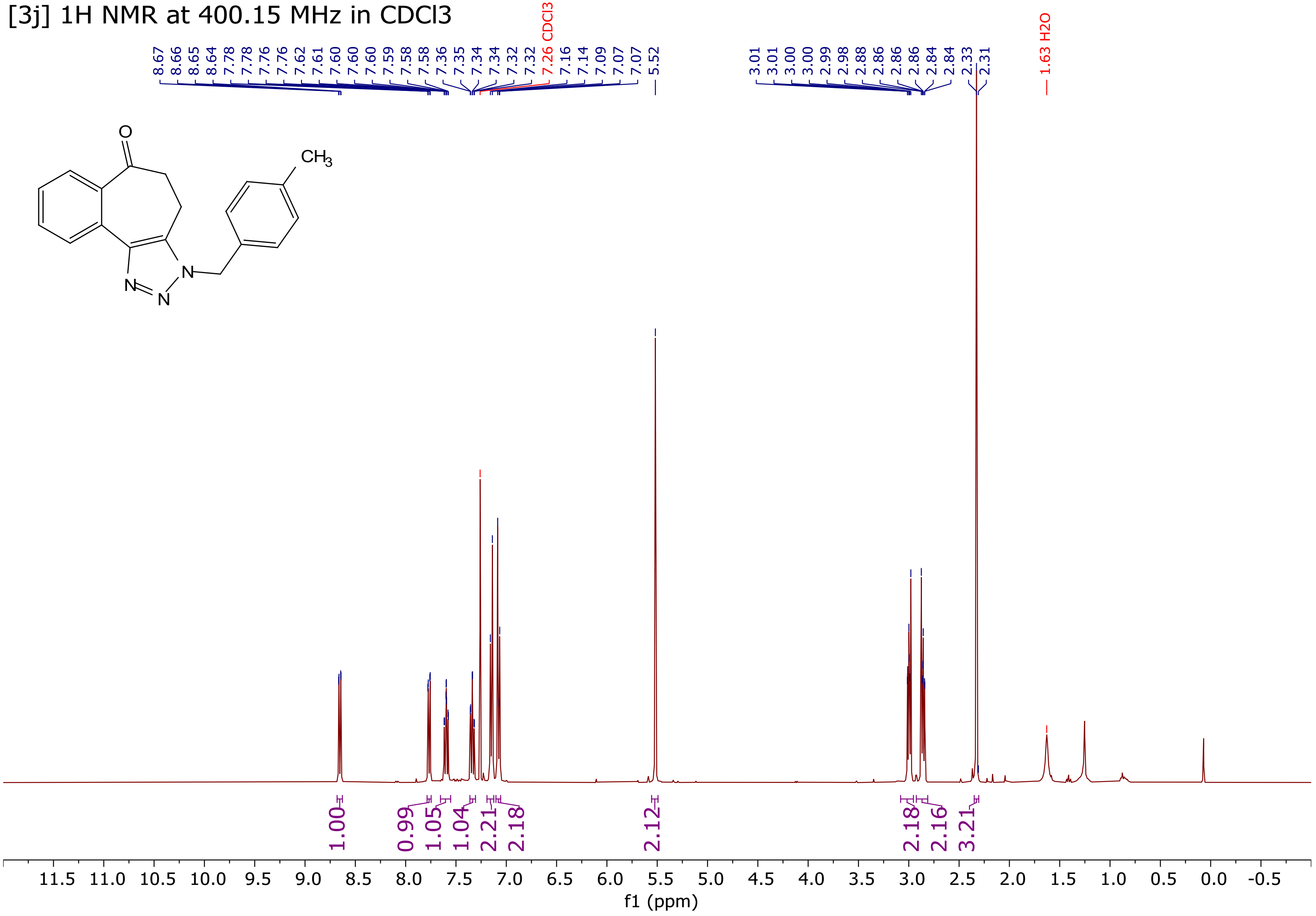

[3j] 13C NMR at 100.63 MHz in CDCl3

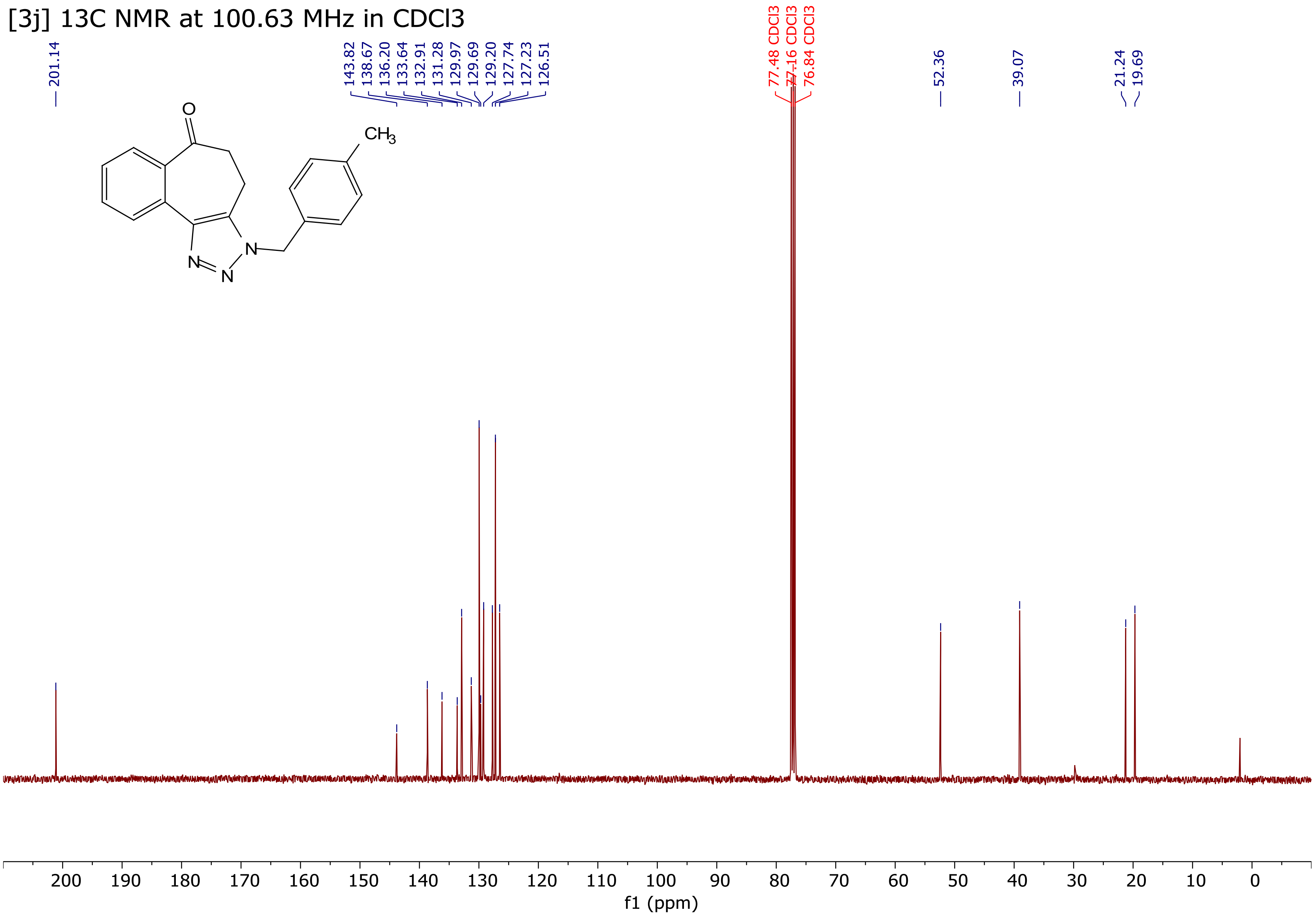

[3k] 1H NMR at 400.15 MHz in CDCl3

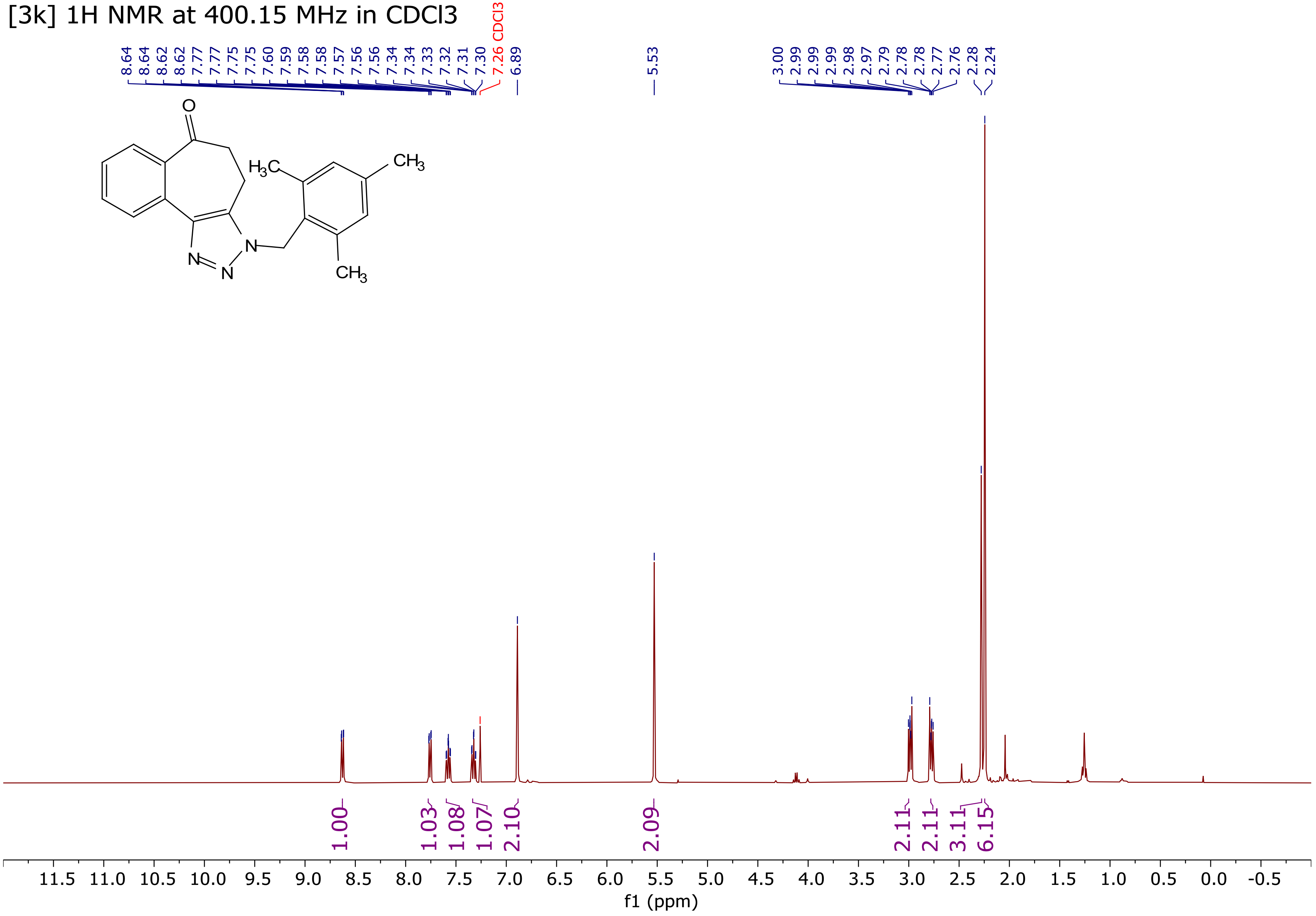

[3k] 13C NMR at 201.27 MHz in CDCl3

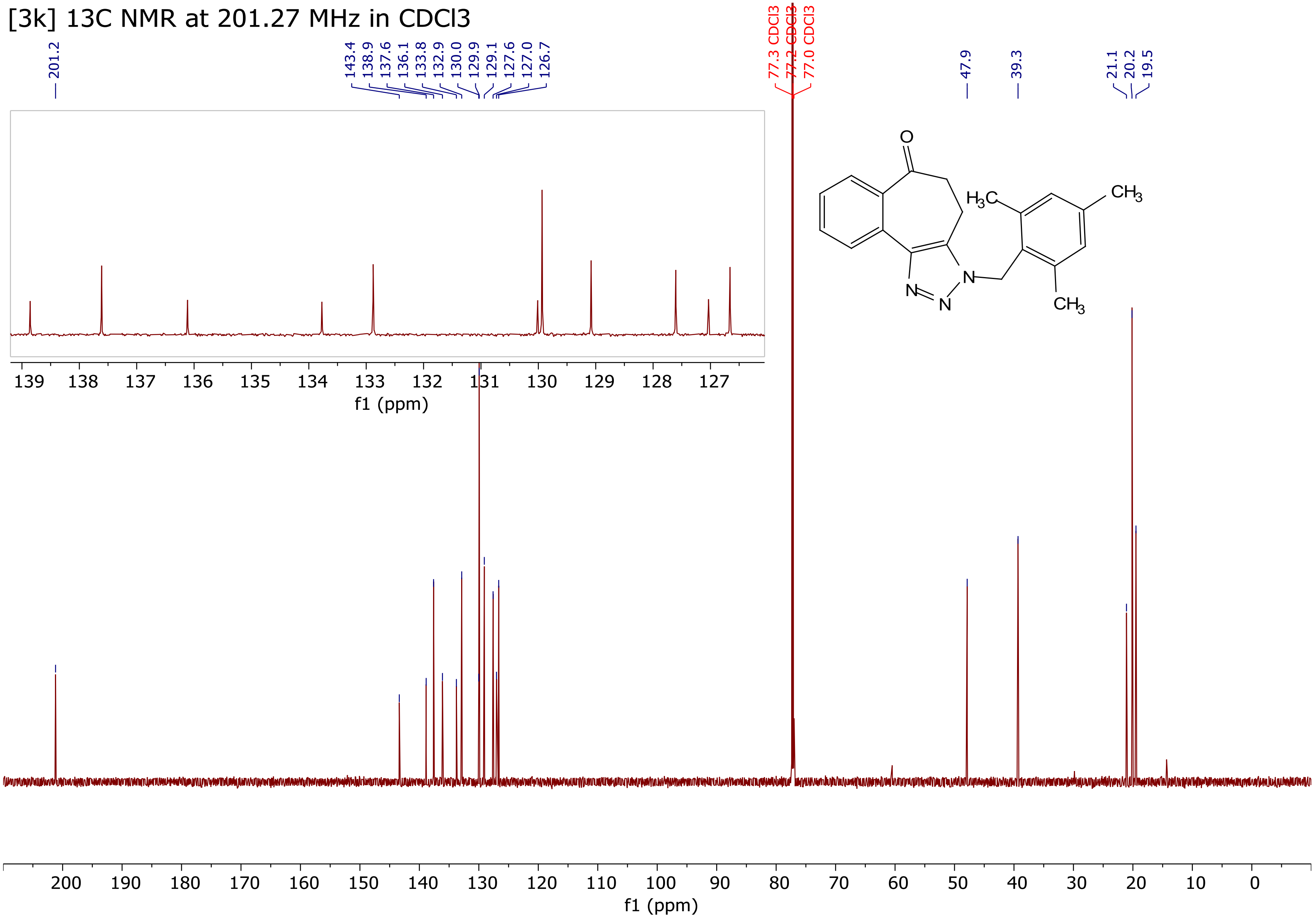

[7a] 1H NMR at 800.34 MHz in CDCl3

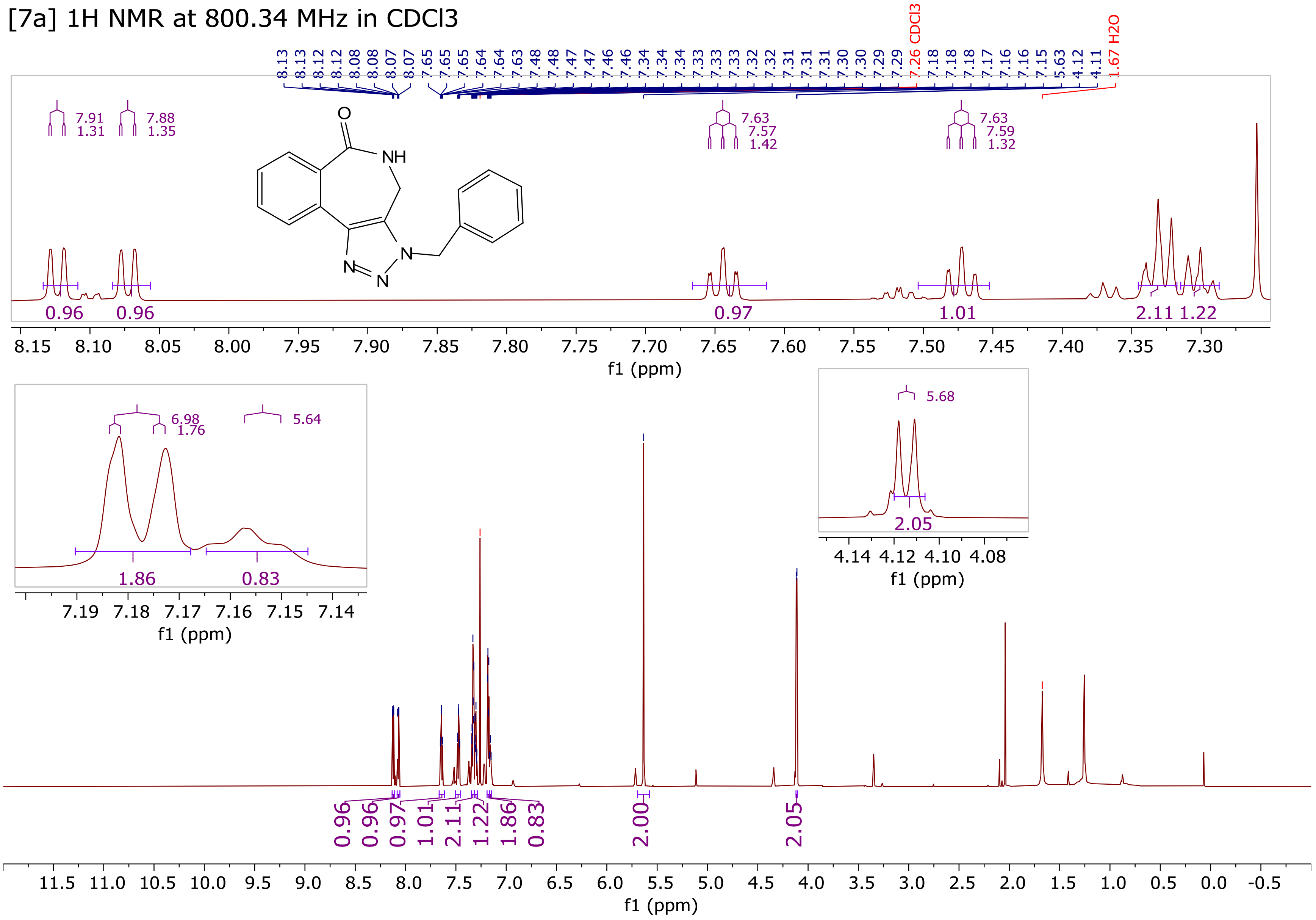

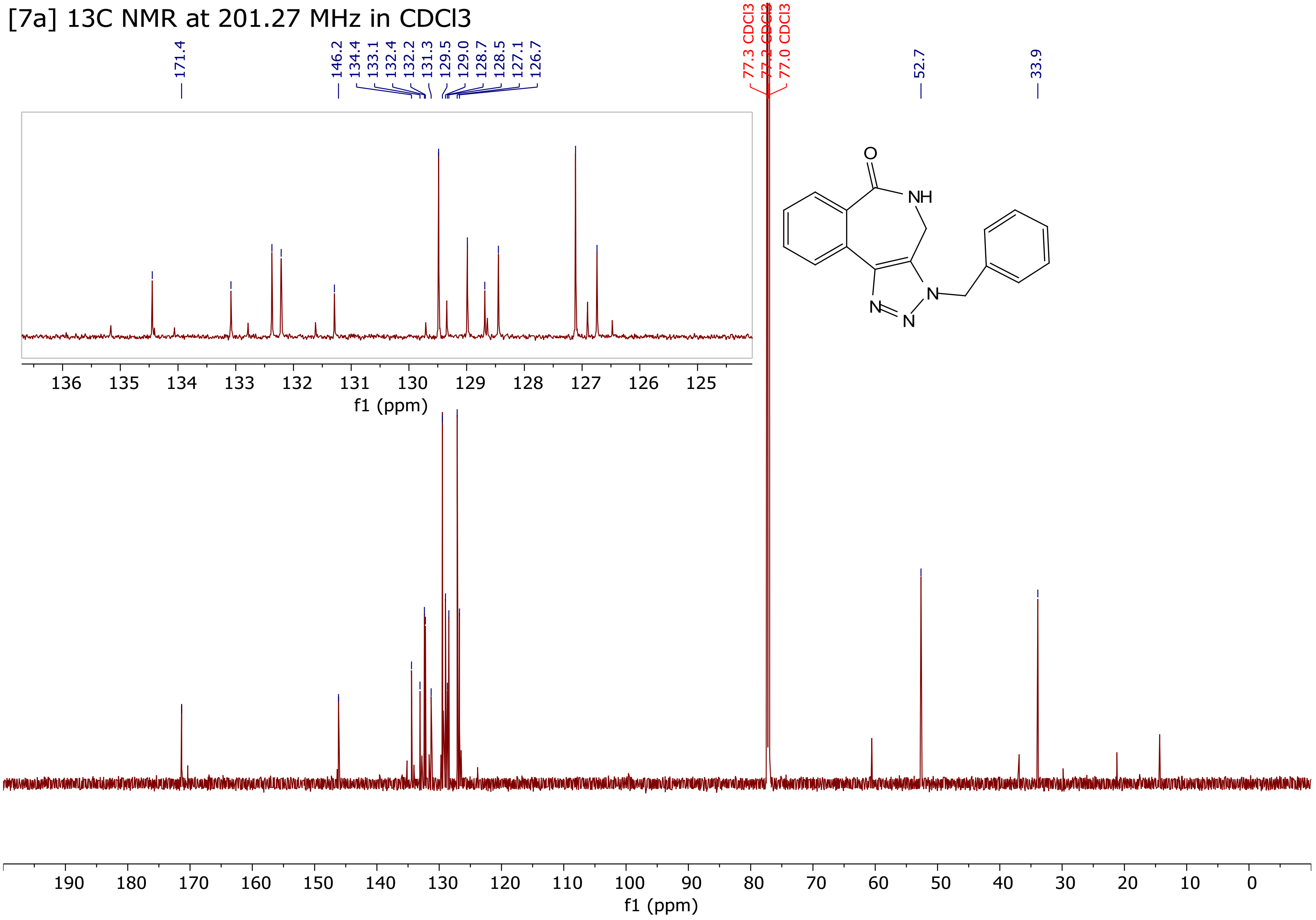
[7a] 13C NMR at 201.27 MHz in CDCl3
171.4
146.2
134.4
133.1
132.4
132.2
131.3
129.5
129.0
128.7
128.5
127.1
126.7
77.3 CDCl3
77.0 CDCl3
52.7
33.9
O
NH
N
N
N
f1 (ppm)
f1 (ppm)

[7d] 1H NMR at 800.34 MHz in CDCl3

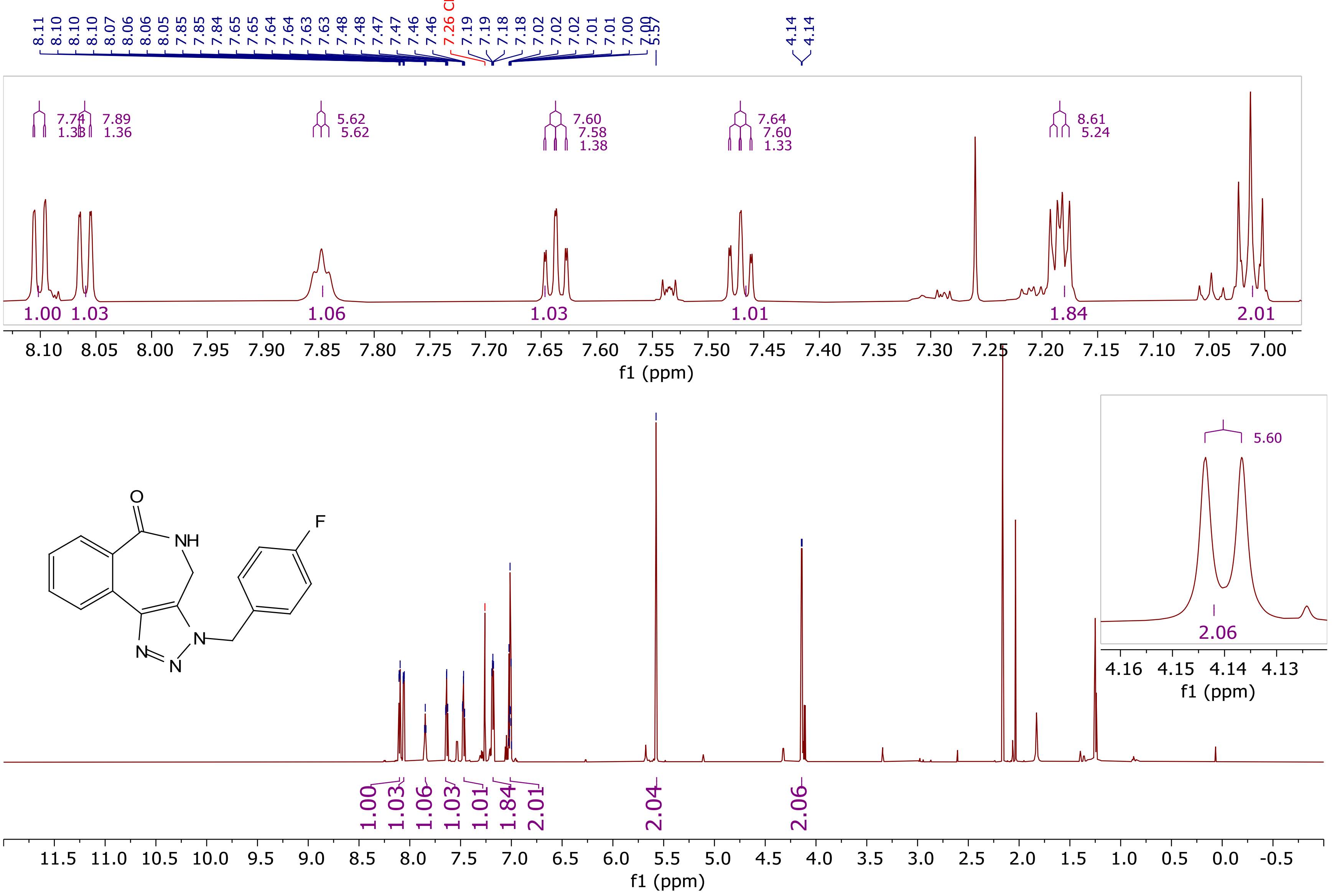

[7d] 13C NMR at 201.27 MHz in CDCl3

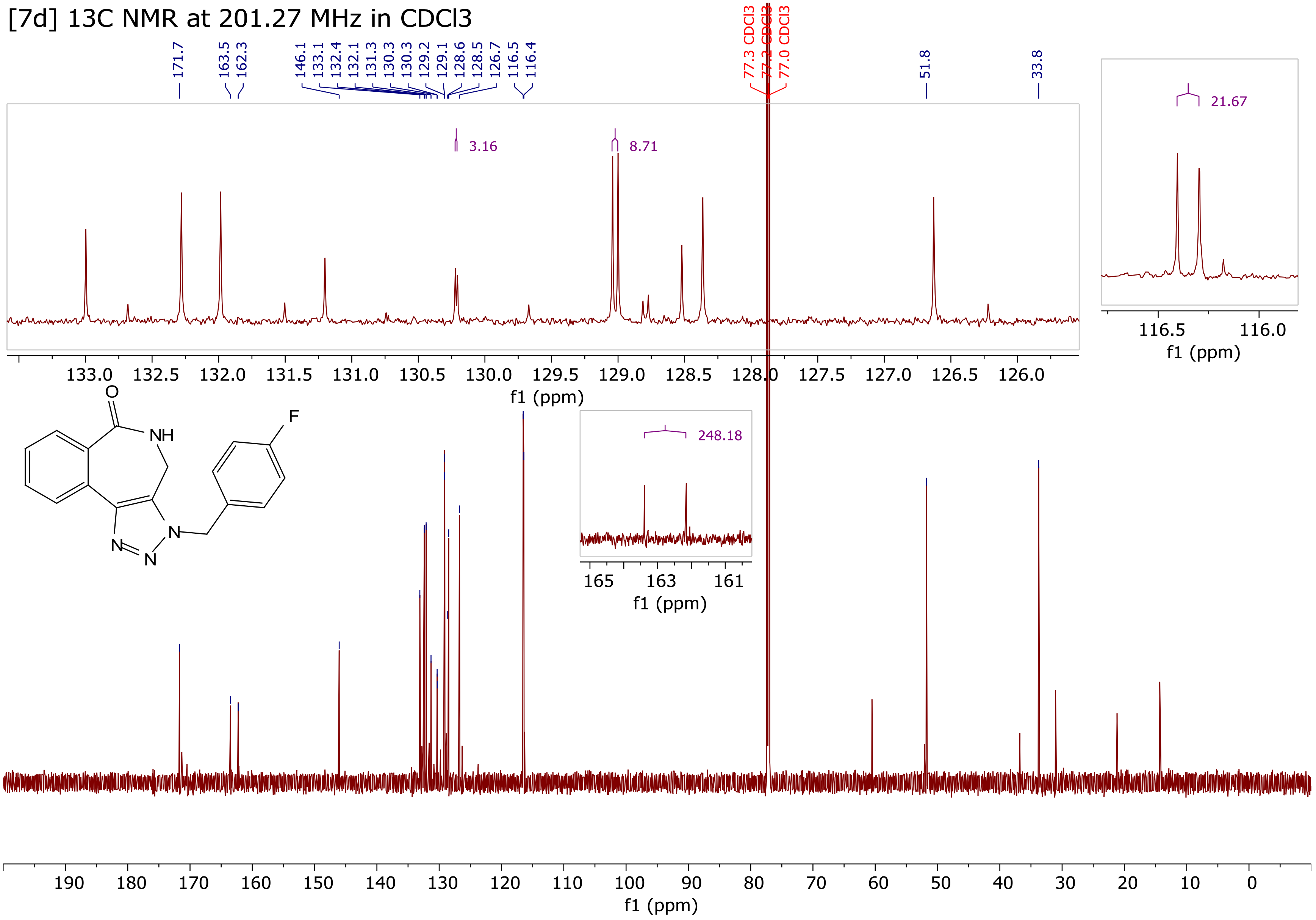

[7d] 19F NMR at 376.48 MHz in CDCl3

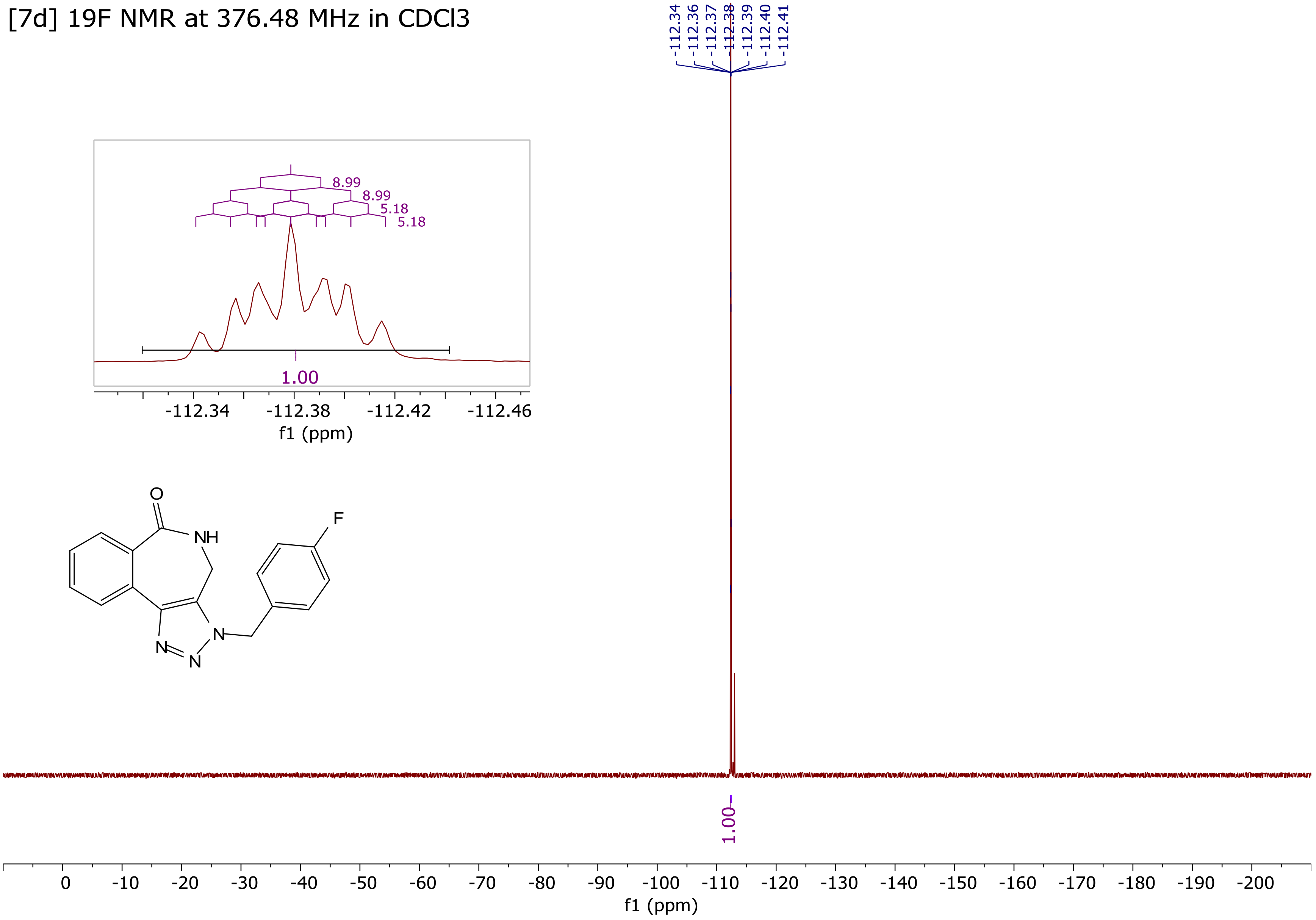

[7j] 1H NMR at 800.34 MHz in CDCl3

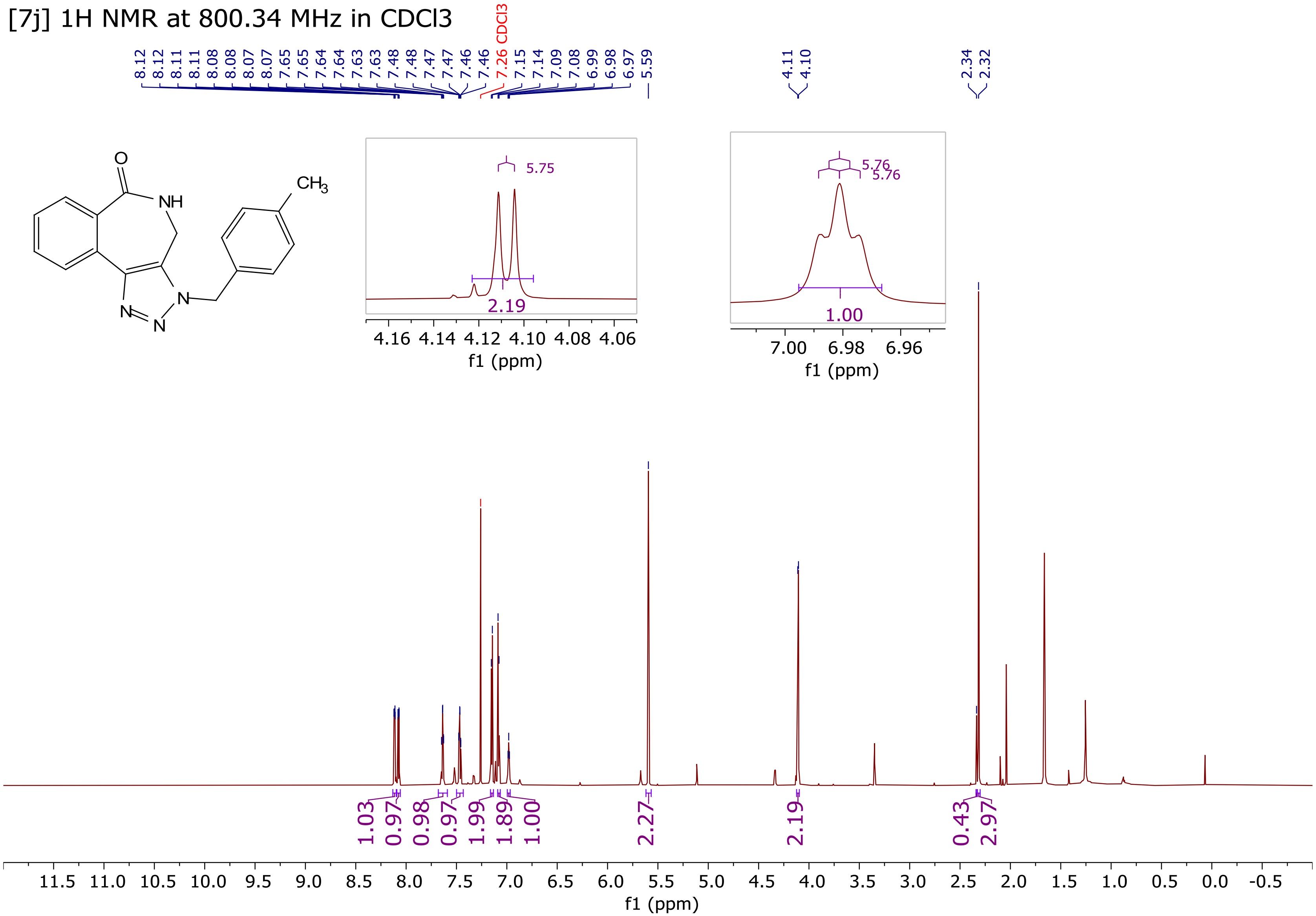

[7j] 13C NMR at 201.27 MHz in CDCl3

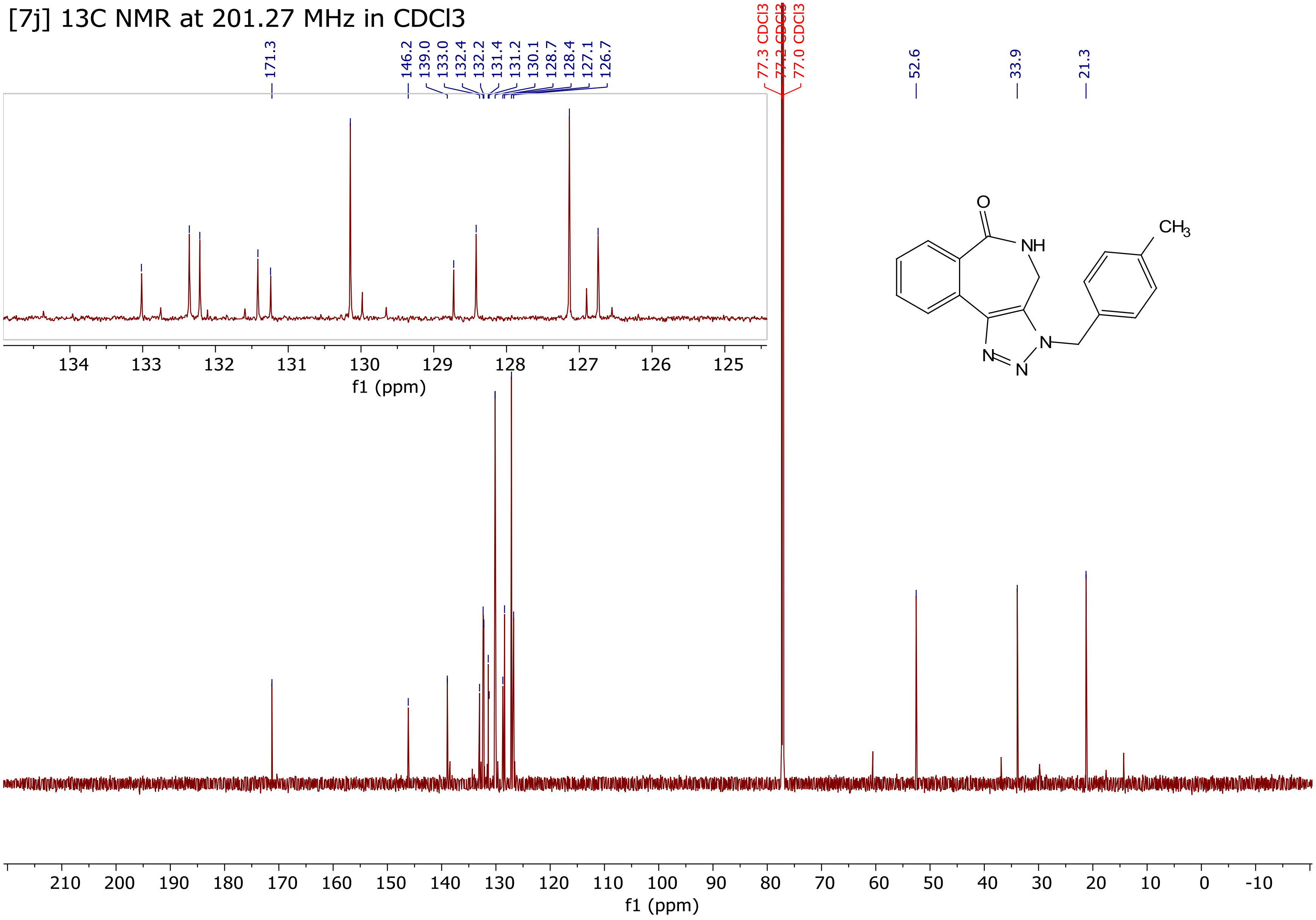

Supplement: Supplementary file 1 — bc4c00595_si_001.pdf [file bc4c00595_si_001.pdf]
