# Supplementary material for: Development of Transiently Strainable Benzocycloheptenes for Catalyst-Free, Visible-Light-Mediated [3 + 2]-Cycloadditions
Source: Bioconjug Chem. 2025 Feb 4;36(2):302–8. doi: 10.1021/acs.bioconjchem.4c00595 (PMC11843616; doi:10.1021/acs.bioconjchem.4c00595)
Supplement: Supplementary file 2 — bc4c00595_si_002.zip [file bc4c00595_si_002.zip › NMR/1b/Primary_NMR_Data_files/1H/pdata/1/email_sk-adm-26-33_10_1.pdf]

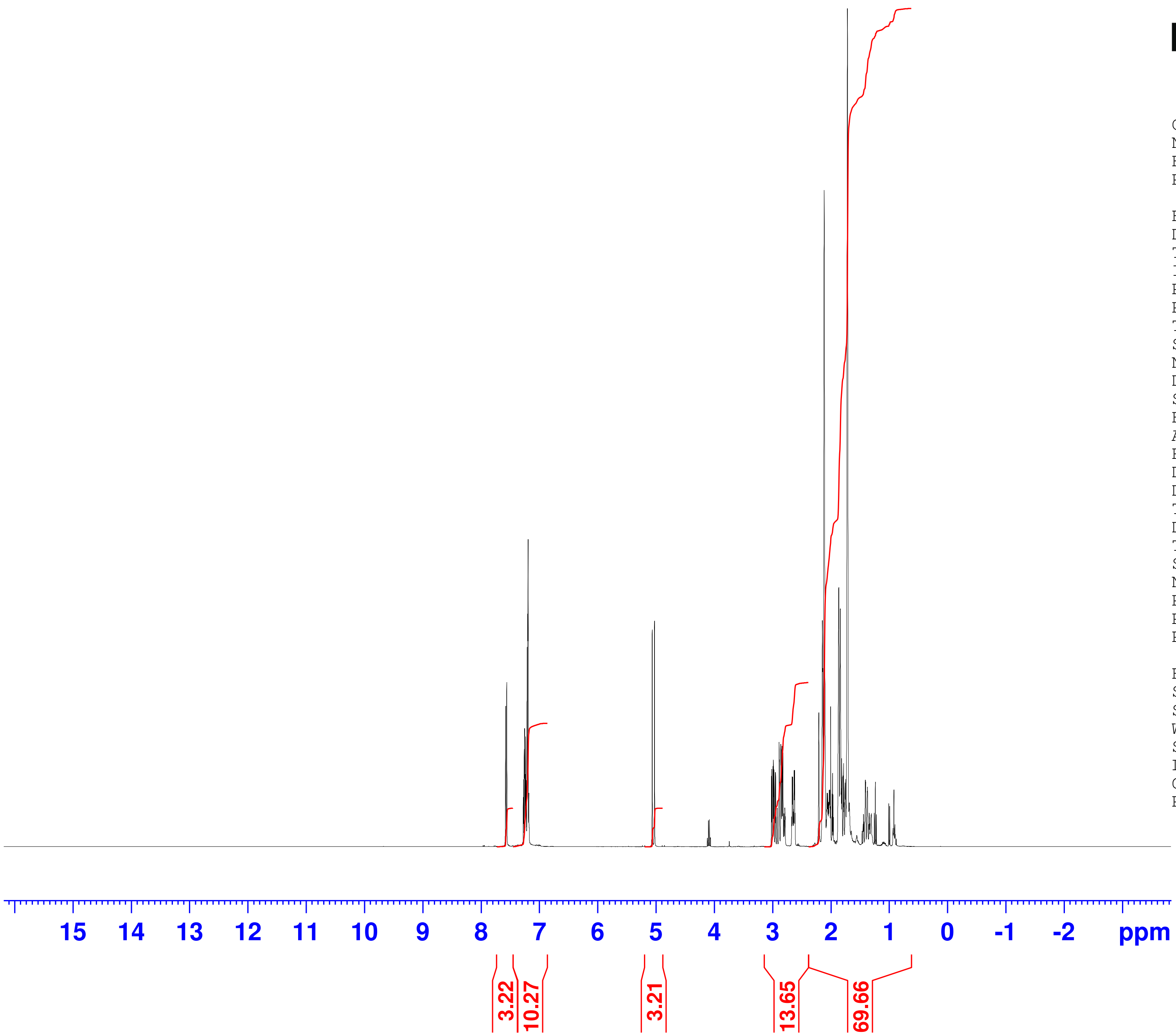


```
Current Data Parameters
NAME          sk-adm-26-33
EXPNO                   10
PROCNO                   1

F2 - Acquisition Parameters
Date_             20211101
Time                  1.28 h
INSTRUM              spect
PROBHD    Z116098_0222 (
PULPROG               zg30
TD                   65536
SOLVENT              CD3CN
NS                      16
DS                       2
SWH               8012.820 Hz
FIDRES            0.244532 Hz
AQ               4.0894465 sec
RG                   31.63
DW                  62.400 usec
DE                    6.50 usec
TE                   298.0 K
D1              1.00000000 sec
TD0                      1
SFO1           400.1524709 MHz
NUC1                    1H
P0                    3.33 usec
P1                   10.00 usec
PLW1           13.89000034 W

F2 - Processing parameters
SI                   65536
SF             400.1500000 MHz
WDW                     EM
SSB      0
LB                    0.30 Hz
GB       0
PC                    1.00
```
