# Supplementary material for: Development of Transiently Strainable Benzocycloheptenes for Catalyst-Free, Visible-Light-Mediated [3 + 2]-Cycloadditions
Source: Bioconjug Chem. 2025 Feb 4;36(2):302–8. doi: 10.1021/acs.bioconjchem.4c00595 (PMC11843616; doi:10.1021/acs.bioconjchem.4c00595)
Supplement: Supplementary file 2 — bc4c00595_si_002.zip [file bc4c00595_si_002.zip › NMR/1d/Primary_NMR_Data_files/13C/pdata/1/email_sk-p2-sm2-silica-spot2_11_1.pdf]

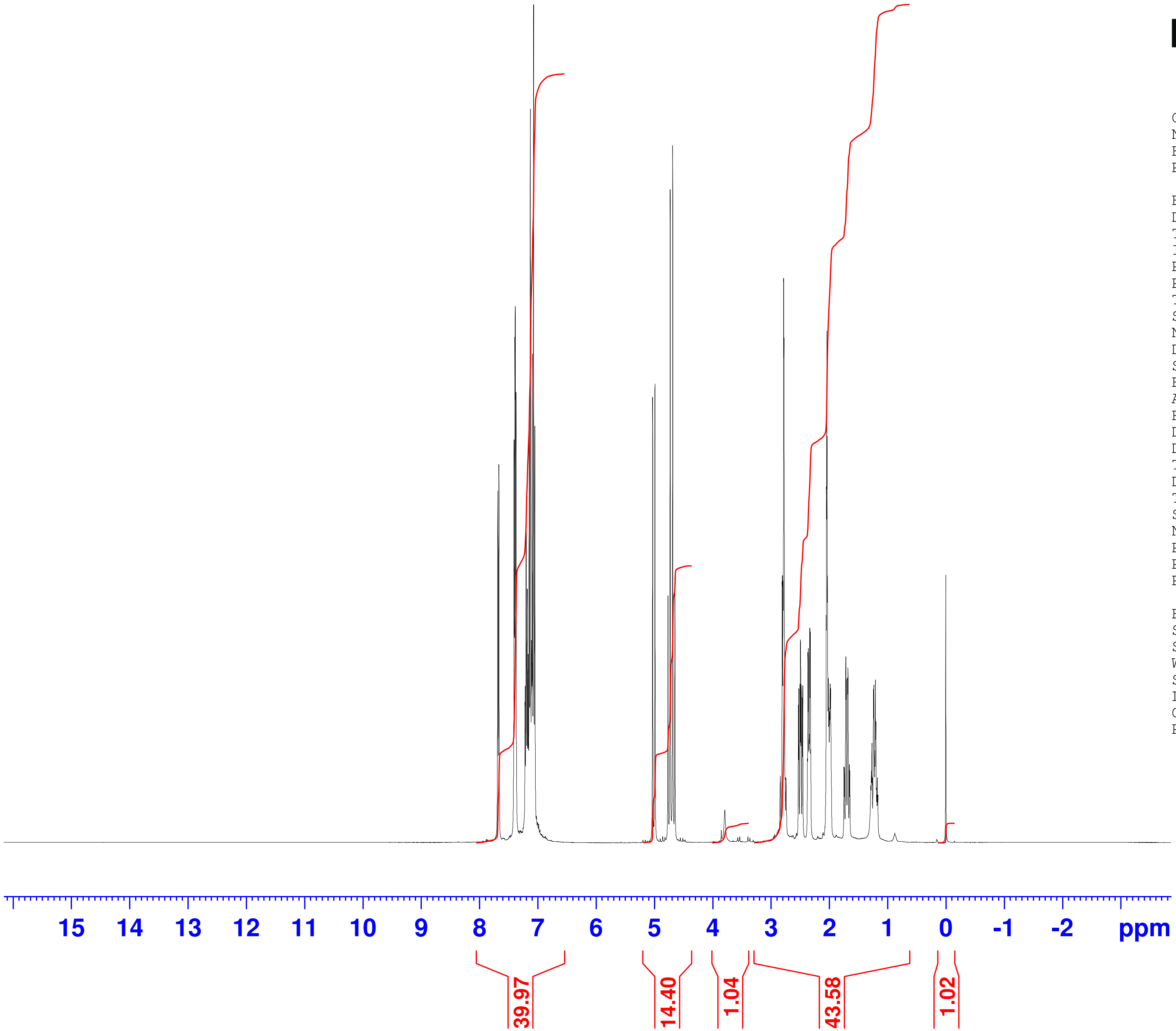


```
Current Data Parameters
NAME      sk-p2-sm2-silica-spot2
EXPNO                         11
PROCNO                         1

F2 - Acquisition Parameters
Date_               20211021
Time                    0.15 h
INSTRUM                spect
PROBHD     Z116098_0222 (
PULPROG                 zg30
TD                     65536
SOLVENT              Acetone
NS                        16
DS                         2
SWH                 8012.820 Hz
FIDRES              0.244532 Hz
AQ                 4.0894465 sec
RG                     11.35
DW                    62.400 usec
DE                      6.50 usec
TE                     298.0 K
D1                1.00000000 sec
TD0                        1
SFO1             400.1524709 MHz
NUC1                      1H
P0                      3.33 usec
P1                     10.00 usec
PLW1             13.89000034 W

F2 - Processing parameters
SI                     65536
SF               400.1500107 MHz
WDW                       EM
SSB      0
LB                      0.30 Hz
GB       0
PC                      1.00
```
