# Supplementary material for: Development of Transiently Strainable Benzocycloheptenes for Catalyst-Free, Visible-Light-Mediated [3 + 2]-Cycloadditions
Source: Bioconjug Chem. 2025 Feb 4;36(2):302–8. doi: 10.1021/acs.bioconjchem.4c00595 (PMC11843616; doi:10.1021/acs.bioconjchem.4c00595)
Supplement: Supplementary file 2 — bc4c00595_si_002.zip [file bc4c00595_si_002.zip › NMR/1d/Primary_NMR_Data_files/1H/pdata/1/email_sk-p2-sm2-silica-spot2_10_1.pdf]

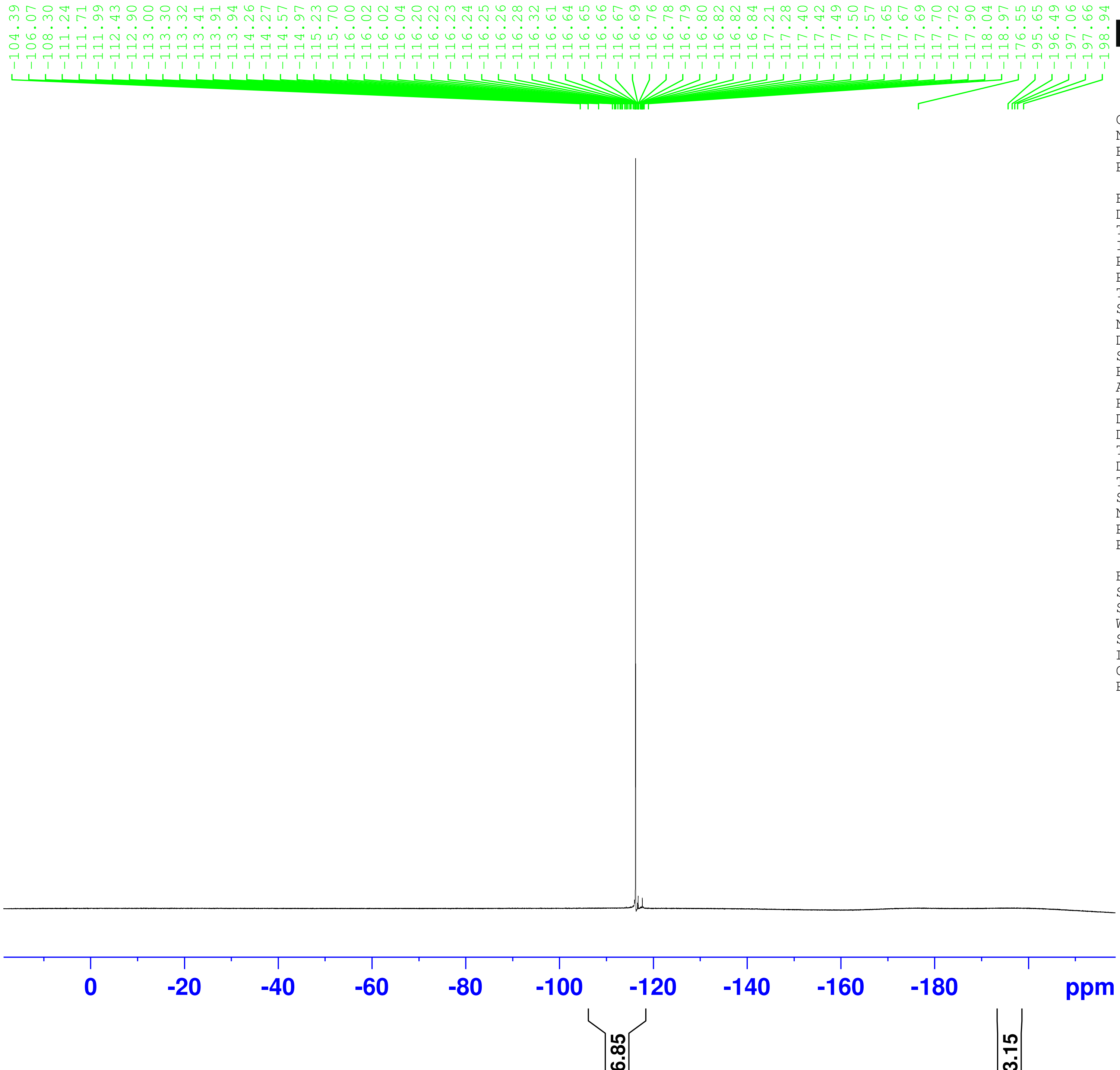

BRUKER

Current Data Parameters

NAME	sk-p2-sm2-silica-spot2
EXPNO	10
PROCNO	1

F2 - Acquisition Parameters

Date_	20211021
Time	0.12 h
INSTRUM	spect
PROBHD	Z116098_0222 (
PULPROG	zgflqn
TD	131072
SOLVENT	Acetone
NS	16
DS	4
SWH	89285.711 Hz
FIDRES	1.362392 Hz
AQ	0.7340032 sec
RG	206.31
DW	5.600 usec
DE	6.50 usec
TE	298.0 K
D1	1.00000000 sec
TD0	1
SFO1	376.4795333 MHz
NUC1	19F
P1	14.00 usec
PLW1	22.00000000 W

F2 - Processing parameters

SI	65536
SF	376.5171850 MHz
WDW	EM
SSB	0
LB	0.30 Hz
GB	0
PC	1.00
