# Supplementary material for: Development of Transiently Strainable Benzocycloheptenes for Catalyst-Free, Visible-Light-Mediated [3 + 2]-Cycloadditions
Source: Bioconjug Chem. 2025 Feb 4;36(2):302–8. doi: 10.1021/acs.bioconjchem.4c00595 (PMC11843616; doi:10.1021/acs.bioconjchem.4c00595)
Supplement: Supplementary file 2 — bc4c00595_si_002.zip [file bc4c00595_si_002.zip › NMR/1d/Primary_NMR_Data_files/NOE/pdata/1/email_sk-p2-sm2-silica-spot2_14_1.pdf]

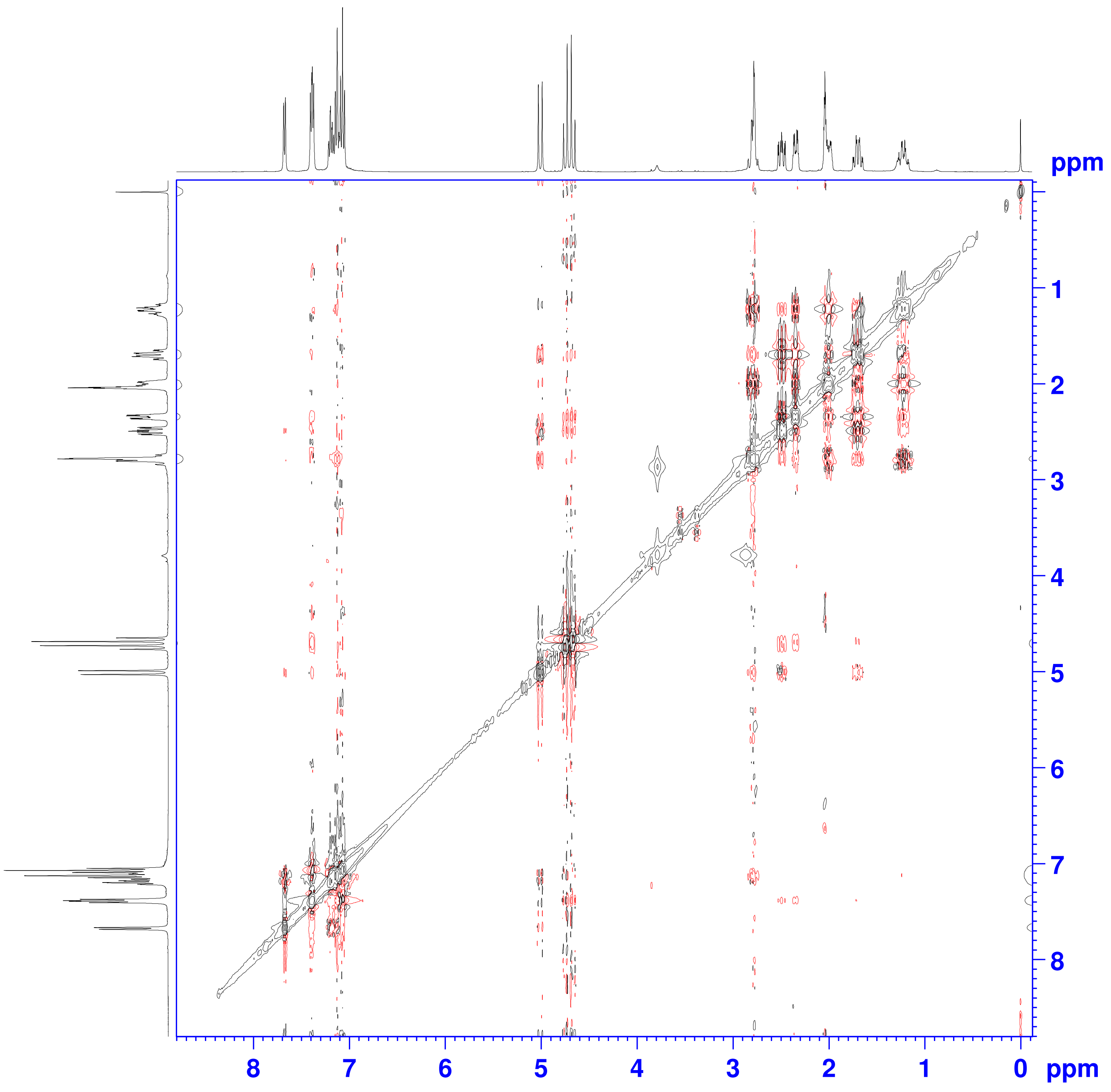

Current Data Parameters

NAME	sk-p2-sm2-silica-spot2	
EXPNO	14	
PROCNO	1	

F2 - Acquisition Parameters

Date_	20211021	
Time	2.43	h
INSTRUM	spect	
PROBHD	Z116098_0222 (	
PULPROG	noesygpphpp	
TD	2048	
SOLVENT	Acetone	
NS	4	
DS	32	
SWH	3571.428	Hz
FIDRES	3.487723	Hz
AQ	0.2867200	sec
RG	11.35	
DW	140.000	usec
DE	6.50	usec
TE	298.0	K
D0	0.00012727	sec
D1	1.96928000	sec
D8	0.30000001	sec
D11	0.03000000	sec
D12	0.00002000	sec
D16	0.00020000	sec
IN0	0.00028000	sec
TDav	1	
SFO1	400.1517476	MHz
NUC1	1H	
P1	10.00	usec
P2	20.00	usec
P17	2500.00	usec
PLW1	13.89000034	W
PLW10	1.54330003	W
GPNAM[1]	SMSQ10.100	
GPZ1	40.00	%
P16	1000.00	usec

F1 - Acquisition parameters

TD	256	
SFO1	400.1517	MHz
FIDRES	27.901785	Hz
SW	8.925	ppm
FnMODE	States-TPPI	

F2 - Processing parameters

SI	1024	
SF	400.1500107	MHz
WDW	QSINE	
SSB	2	
LB	0	Hz
GB	0	
PC	1.00	

F1 - Processing parameters

SI	1024	
MC2	States-TPPI	
SF	400.1500107	MHz
WDW	QSINE	
SSB	2	
LB	0	Hz
GB	0	
