# Supplementary material for: Development of Transiently Strainable Benzocycloheptenes for Catalyst-Free, Visible-Light-Mediated [3 + 2]-Cycloadditions
Source: Bioconjug Chem. 2025 Feb 4;36(2):302–8. doi: 10.1021/acs.bioconjchem.4c00595 (PMC11843616; doi:10.1021/acs.bioconjchem.4c00595)
Supplement: Supplementary file 2 — bc4c00595_si_002.zip [file bc4c00595_si_002.zip › NMR/2c/Primary_NMR_Data_files/1H/pdata/1/email_sk-n-boc-triazoline_10_1.pdf]

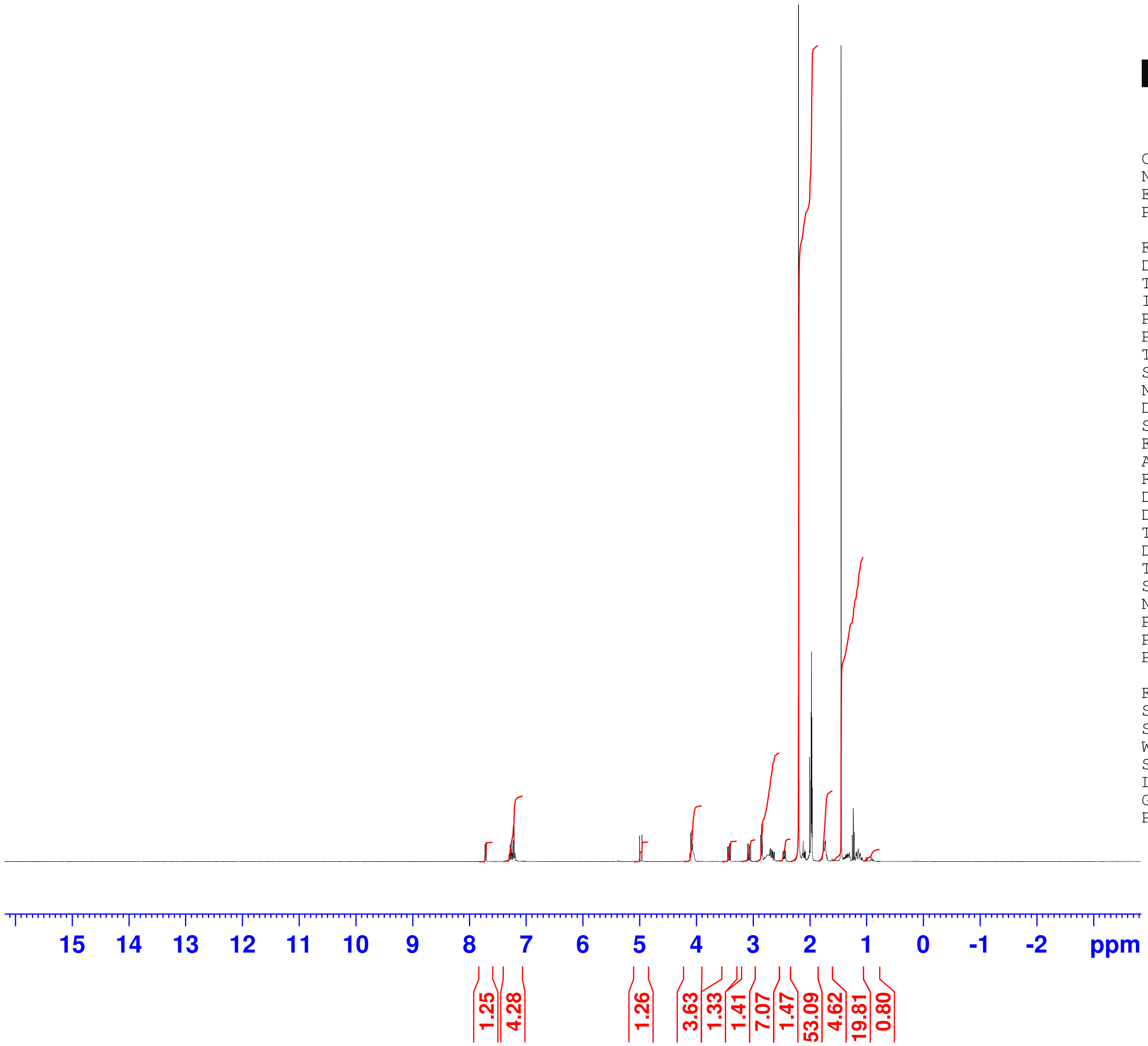


```
Current Data Parameters
NAME       sk-n-boc-triazoline
EXPNO                    10
PROCNO                    1

F2 - Acquisition Parameters
Date_              20211007
Time                   4.13 h
INSTRUM               spect
PROBHD     Z116098_0222 (
PULPROG                zg30
TD                    65536
SOLVENT                CD3CN
NS                       16
DS                        2
SWH                8012.820 Hz
FIDRES             0.244532 Hz
AQ                4.0894465 sec
RG                   111.22
DW                   62.400 usec
DE                     6.50 usec
TE                    298.0 K
D1               1.00000000 sec
TD0                       1
SFO1            400.1524709 MHz
NUC1                     1H
P0                     3.33 usec
P1                    10.00 usec
PLW1            13.89000034 W

F2 - Processing parameters
SI                    65536
SF              400.1500000 MHz
WDW                      EM
SSB        0
LB                     0.30 Hz
GB         0
PC                     1.00
```
