# Supplementary material for: Development of Transiently Strainable Benzocycloheptenes for Catalyst-Free, Visible-Light-Mediated [3 + 2]-Cycloadditions
Source: Bioconjug Chem. 2025 Feb 4;36(2):302–8. doi: 10.1021/acs.bioconjchem.4c00595 (PMC11843616; doi:10.1021/acs.bioconjchem.4c00595)
Supplement: Supplementary file 2 — bc4c00595_si_002.zip [file bc4c00595_si_002.zip › NMR/2d/Primary_NMR_Data_files/13C/pdata/1/email_sk-p2-sm2-polar spot_12_1.pdf]

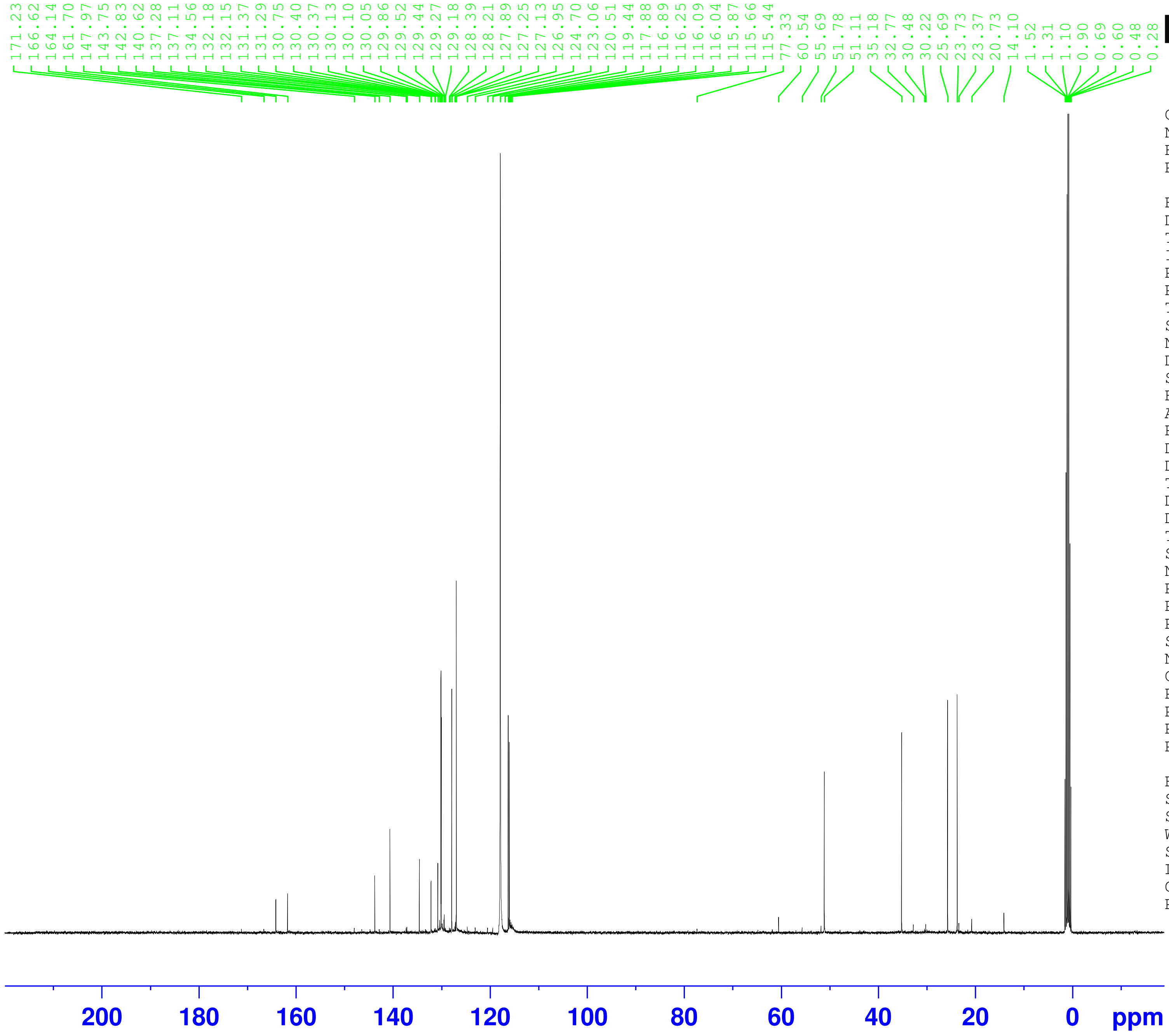


```
Current Data Parameters
NAME      sk-p2-sm2-polar spot
EXPNO                   12
PROCNO                   1

F2 - Acquisition Parameters
Date_             20210914
Time                 23.42 h
INSTRUM              spect
PROBHD    Z116098_0222 (
PULPROG            zgpg30
TD                   65536
SOLVENT              CD3CN
NS                    1024
DS                       4
SWH              24038.461 Hz
FIDRES            0.733596 Hz
AQ               1.3631488 sec
RG                  206.31
DW                  20.800 usec
DE                    6.50 usec
TE                   298.0 K
D1              2.00000000 sec
D11             0.03000000 sec
TD0                      1
SFO1           100.6278593 MHz
NUC1                   13C
P0                    3.33 usec
P1                   10.00 usec
PLW1           70.00000000 W
SFO2           400.1516006 MHz
NUC2                    1H
CPDPRG[2           waltz65
PCPD2                90.00 usec
PLW2           13.89000034 W
PLW12           0.17148000 W
PLW13           0.08625400 W

F2 - Processing parameters
SI                   32768
SF             100.6177435 MHz
WDW                     EM
SSB      0
LB                    1.00 Hz
GB       0
PC                    1.40
```
