# Supplementary material for: Development of Transiently Strainable Benzocycloheptenes for Catalyst-Free, Visible-Light-Mediated [3 + 2]-Cycloadditions
Source: Bioconjug Chem. 2025 Feb 4;36(2):302–8. doi: 10.1021/acs.bioconjchem.4c00595 (PMC11843616; doi:10.1021/acs.bioconjchem.4c00595)
Supplement: Supplementary file 2 — bc4c00595_si_002.zip [file bc4c00595_si_002.zip › NMR/3a/Primary_NMR_Data_files/HSQC/pdata/1/email_sk-1-triazole_12_1.pdf]

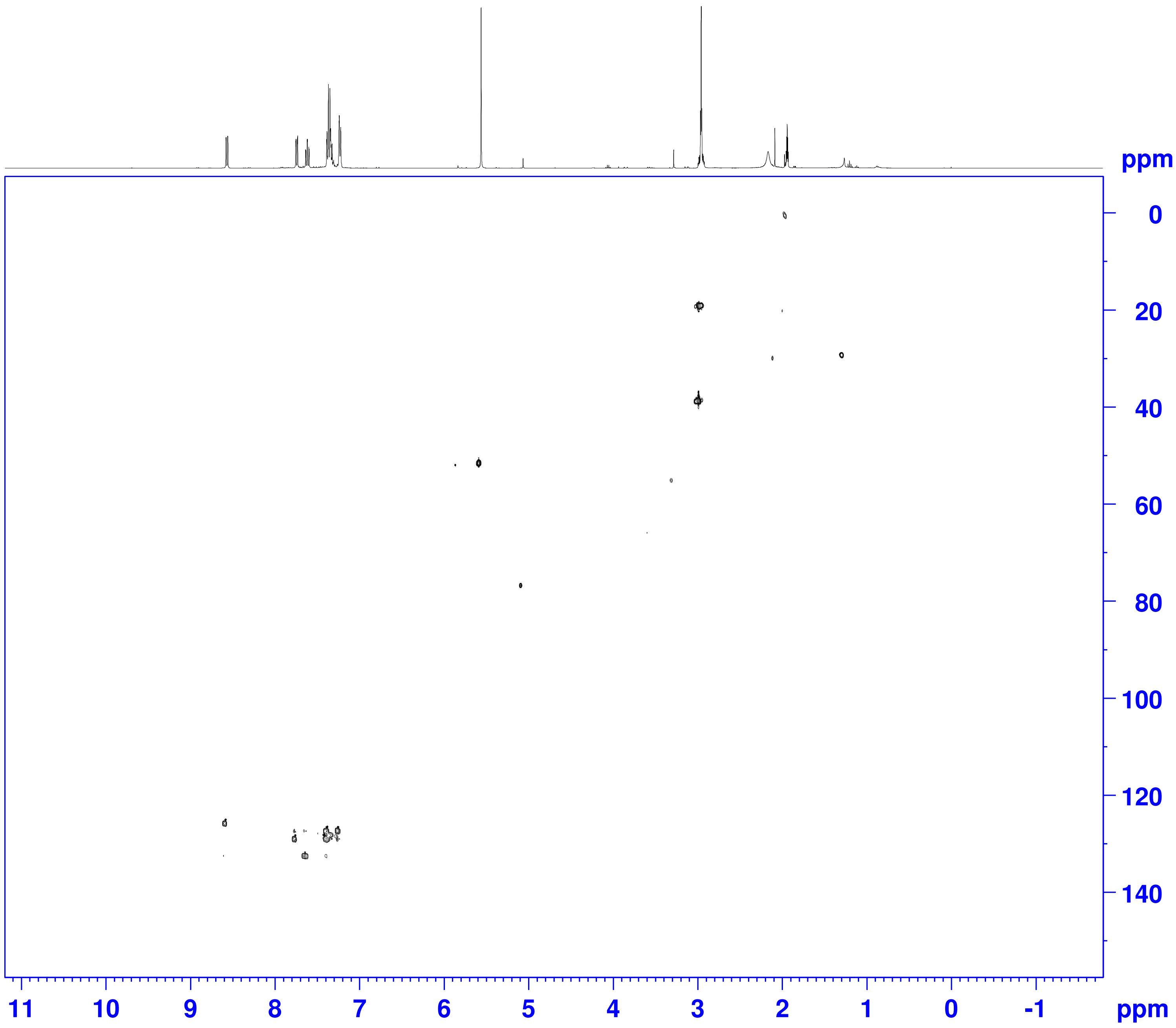

BRUKER

```
Current Data Parameters
NAME      sk-1-triazole
EXPNO                12
PROCNO                1

F2 - Acquisition Parameters
Date_          20220202
Time               8.05 h
INSTRUM           spect
PROBHD   Z116098_0222 (
PULPROG  hsqcedetgpsisp2.3
TD                 2048
SOLVENT           CD3CN
NS                    4
DS                   32
SWH            5197.505 Hz
FIDRES         5.075689 Hz
AQ            0.1970176 sec
RG               206.31
DW               96.200 usec
DE                 6.50 usec
TE                298.0 K
CNST2       145.0000000
CNST17       -0.5000000
D0           0.00000300 sec
D1           1.50000000 sec
D4           0.00172414 sec
D11          0.03000000 sec
D16          0.00020000 sec
D21          0.00344800 sec
D24          0.00086200 sec
IN0          0.00003010 sec
TDav                  1
SFO1        400.1518807 MHz
NUC1                 1H
P1                10.00 usec
P2                20.00 usec
P28             1000.00 usec
PLW1        13.89000034 W
SFO2        100.6253439 MHz
NUC2                13C
CPDPRG[2          garp4
P3                10.00 usec
P14              500.00 usec
P24             2000.00 usec
P31             2119.00 usec
PCPD2             80.00 usec
PLW0     0 W
PLW2        70.00000000 W
PLW12        1.09379995 W
SPNAM[3] Crp60,0.5,20.1
SPOAL3            0.500
SPOFFS3  0 Hz
SPW3        10.69499969 W
SPNAM[7]    Crp60comp.4
SPOAL7            0.500
SPOFFS7  0 Hz
SPW7        10.69499969 W
SPNAM[18  Crp60_xfilt.2
SPOAL18           0.500
SPOFFS18 0 Hz
SPW18        2.06040001 W
GPNAM[1]     SMSQ10.100
GPZ1              80.00 %
GPNAM[2]     SMSQ10.100
GPZ2              20.10 %
GPNAM[3]     SMSQ10.100
GPZ3              11.00 %
GPNAM[4]     SMSQ10.100
GPZ4              -5.00 %
P16             1000.00 usec
P19              600.00 usec

F1 - Acquisition parameters
TD                  256
SFO1           100.6253 MHz
FIDRES       129.775742 Hz
SW              165.081 ppm
FnMODE    Echo-Antiecho

F2 - Processing parameters
SI                 1024
SF          400.1500000 MHz
WDW               QSINE
SSB                   2
LB       0 Hz
GB       0
PC                 1.40

F1 - Processing parameters
SI                 1024
MC2       echo-antiecho
SF          100.6177975 MHz
WDW               QSINE
SSB                   2
LB       0 Hz
GB       0
```
