# Supplementary material for: Development of Transiently Strainable Benzocycloheptenes for Catalyst-Free, Visible-Light-Mediated [3 + 2]-Cycloadditions
Source: Bioconjug Chem. 2025 Feb 4;36(2):302–8. doi: 10.1021/acs.bioconjchem.4c00595 (PMC11843616; doi:10.1021/acs.bioconjchem.4c00595)
Supplement: Supplementary file 2 — bc4c00595_si_002.zip [file bc4c00595_si_002.zip › NMR/3d/Primary_NMR_Data_files/19F/pdata/1/email_sk-02-017_10_1.pdf]

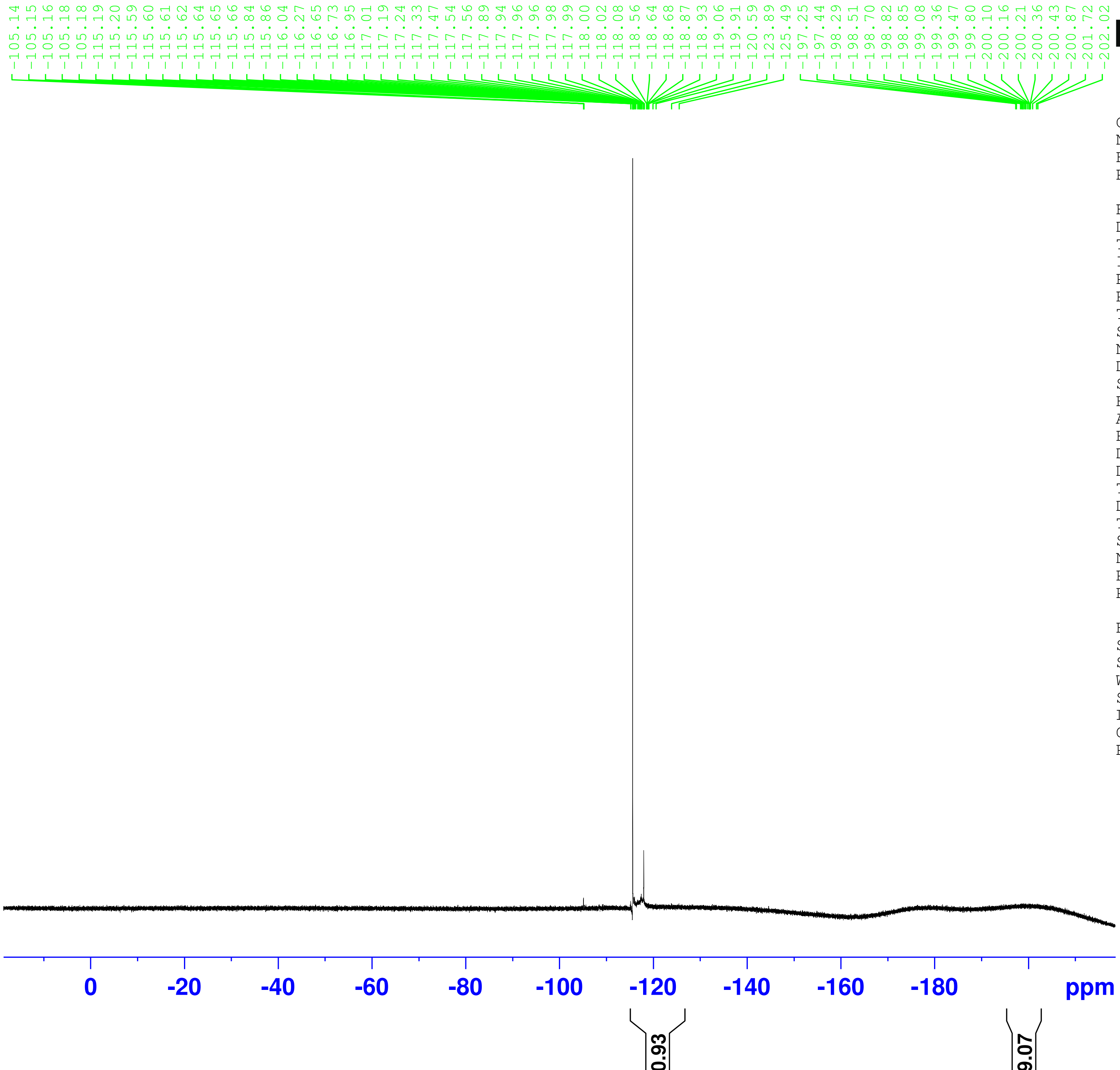

Current Data Parameters
NAME sk-02-017
EXPNO 10
PROCNO 1

F2 - Acquisition Parameters
Date_ 20220103
Time 2.02 h
INSTRUM spect
PROBHD Z116098_0222 (
PULPROG zgflqn
TD 131072
SOLVENT CD3CN
NS 16
DS 4
SWH 89285.711 Hz
FIDRES 1.362392 Hz
AQ 0.7340032 sec
RG 206.31
DW 5.600 usec
DE 6.50 usec
TE 298.0 K
D1 1.00000000 sec
TD0 1
SFO1 376.4795333 MHz
NUC1 19F
P1 14.00 usec
PLW1 22.00000000 W

F2 - Processing parameters
SI 65536
SF 376.5171850 MHz
WDW EM
SSB 0
LB 0.30 Hz
GB 0
PC 1.00
