# Supplementary material for: Development of Transiently Strainable Benzocycloheptenes for Catalyst-Free, Visible-Light-Mediated [3 + 2]-Cycloadditions
Source: Bioconjug Chem. 2025 Feb 4;36(2):302–8. doi: 10.1021/acs.bioconjchem.4c00595 (PMC11843616; doi:10.1021/acs.bioconjchem.4c00595)
Supplement: Supplementary file 2 — bc4c00595_si_002.zip [file bc4c00595_si_002.zip › NMR/3g/Primary_NMR_Data_files/1H/pdata/1/email_sk-k-5-p_10_1.pdf]

BRUKER

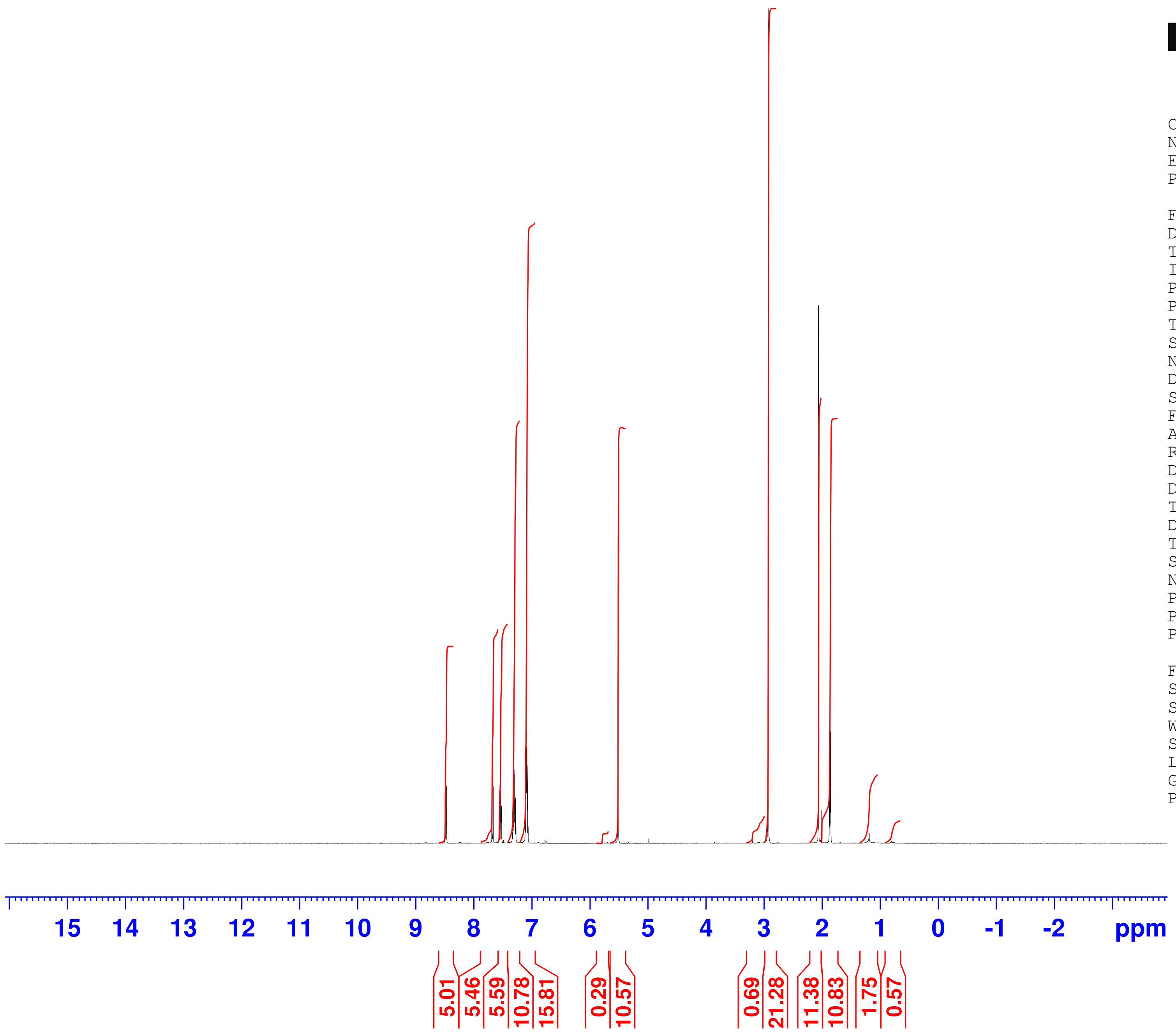


Current Data Parameters
NAME sk-k-5-p
EXPNO 10
PROCNO 1

F2 - Acquisition Parameters
Date_ 20220128
Time 5.03 h
INSTRUM spect
PROBHD Z116098_0222 (
PULPROG zg30
TD 65536
SOLVENT CD3CN
NS 16
DS 2
SWH 8012.820 Hz
FIDRES 0.244532 Hz
AQ 4.0894465 sec
RG 61.86
DW 62.400 usec
DE 6.50 usec
TE 298.0 K
D1 1.00000000 sec
TD0 1
SFO1 400.1524709 MHz
NUC1 1H
P0 3.33 usec
P1 10.00 usec
PLW1 13.89000034 W

F2 - Processing parameters
SI 65536
SF 400.1500440 MHz
WDW EM
SSB 0
LB 0.30 Hz
GB 0
PC 1.00
