# Supplementary material for: Development of Transiently Strainable Benzocycloheptenes for Catalyst-Free, Visible-Light-Mediated [3 + 2]-Cycloadditions
Source: Bioconjug Chem. 2025 Feb 4;36(2):302–8. doi: 10.1021/acs.bioconjchem.4c00595 (PMC11843616; doi:10.1021/acs.bioconjchem.4c00595)
Supplement: Supplementary file 2 — bc4c00595_si_002.zip [file bc4c00595_si_002.zip › NMR/3i/Primary_NMR_Data_files/13C/pdata/1/email_OA2020_51_1.pdf]

after combining fraction

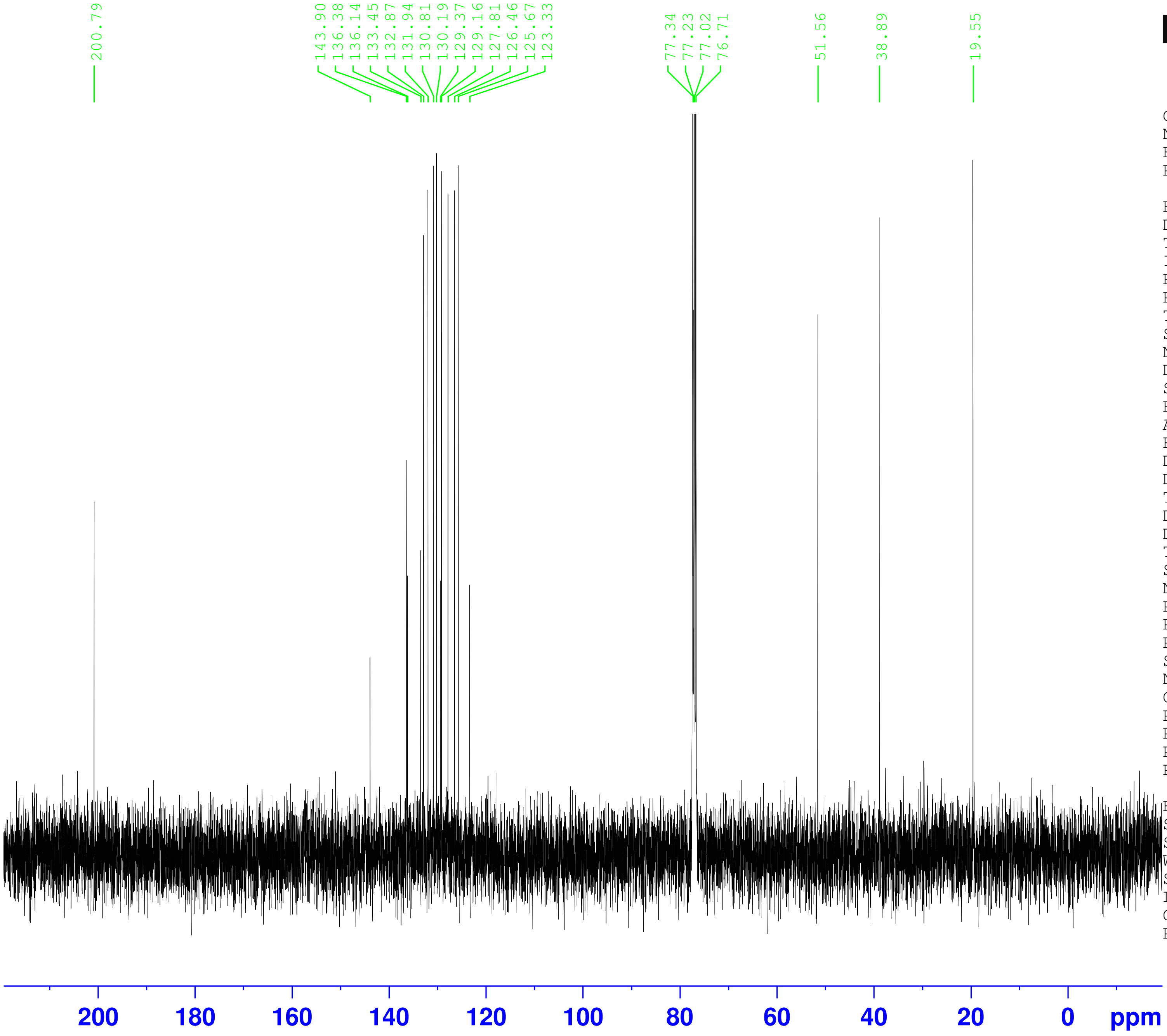

Current Data Parameters

NAME	OA2020	
EXPNO	51	
PROCNO	1	

F2 - Acquisition Parameters

Date_	20230710	
Time	15.53	h
INSTRUM	spect	
PROBHD	Z116098_0222 (	
PULPROG	zgpg30	
TD	65536	
SOLVENT	CDCl3	
NS	1024	
DS	4	
SWH	24038.461	Hz
FIDRES	0.733596	Hz
AQ	1.3631488	sec
RG	206.31	
DW	20.800	usec
DE	6.50	usec
TE	298.0	K
D1	2.00000000	sec
D11	0.03000000	sec
TD0	1	
SFO1	100.6278593	MHz
NUC1	13C	
P0	3.33	usec
P1	10.00	usec
PLW1	70.00000000	W
SFO2	400.1516006	MHz
NUC2	1H	
CPDPRG[2	waltz65	
PCPD2	90.00	usec
PLW2	13.89000034	W
PLW12	0.17148000	W
PLW13	0.08625400	W

F2 - Processing parameters

SI	32768	
SF	100.6177975	MHz
WDW	EM	
SSB	0	
LB	1.00	Hz
GB	0	
PC	1.40	
