# Supplementary material for: Development of Transiently Strainable Benzocycloheptenes for Catalyst-Free, Visible-Light-Mediated [3 + 2]-Cycloadditions
Source: Bioconjug Chem. 2025 Feb 4;36(2):302–8. doi: 10.1021/acs.bioconjchem.4c00595 (PMC11843616; doi:10.1021/acs.bioconjchem.4c00595)
Supplement: Supplementary file 2 — bc4c00595_si_002.zip [file bc4c00595_si_002.zip › NMR/3j/Primary_NMR_Data_files/13C/pdata/1/email_sk-3j_13_1.pdf]

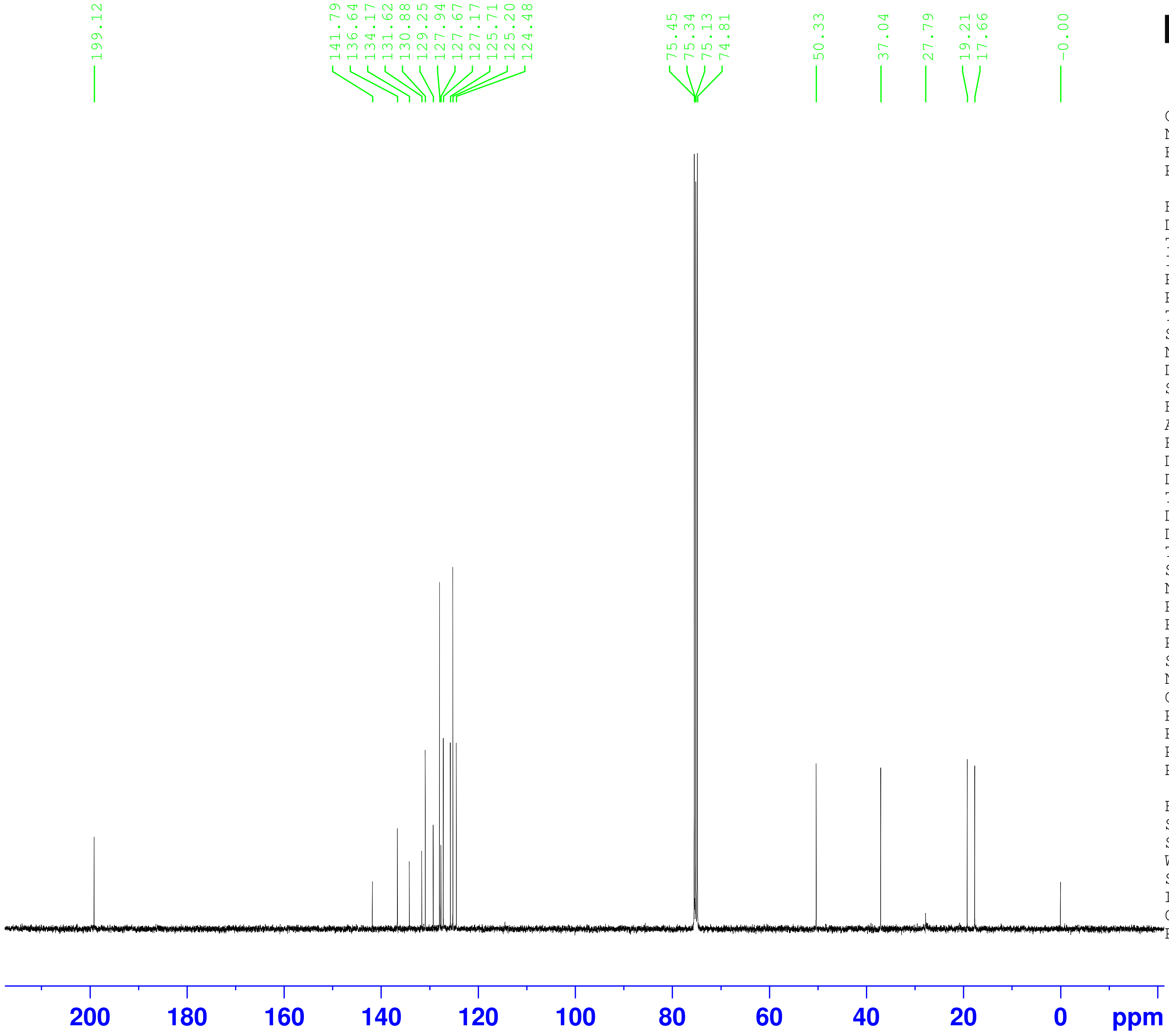

Current Data Parameters

NAME	sk-3j
EXPNO	13
PROCNO	1

F2 - Acquisition Parameters

Date_	20240505
Time	3.09 h
INSTRUM	spect
PROBHD	Z116098_0222 (
PULPROG	zgpg30
TD	65536
SOLVENT	CDCl3
NS	1024
DS	4
SWH	24038.461 Hz
FIDRES	0.733596 Hz
AQ	1.3631488 sec
RG	206.31
DW	20.800 usec
DE	6.50 usec
TE	298.0 K
D1	2.00000000 sec
D11	0.03000000 sec
TD0	1
SFO1	100.6278593 MHz
NUC1	13C
P0	3.33 usec
P1	10.00 usec
PLW1	70.00000000 W
SFO2	400.1516006 MHz
NUC2	1H
CPDPRG[2	waltz65
PCPD2	90.00 usec
PLW2	13.89000034 W
PLW12	0.17148000 W
PLW13	0.08625400 W

F2 - Processing parameters

SI	32768
SF	100.6179902 MHz
WDW	EM
SSB	0
LB	1.00 Hz
GB	0
PC	1.40
