# Supplementary material for: Development of Transiently Strainable Benzocycloheptenes for Catalyst-Free, Visible-Light-Mediated [3 + 2]-Cycloadditions
Source: Bioconjug Chem. 2025 Feb 4;36(2):302–8. doi: 10.1021/acs.bioconjchem.4c00595 (PMC11843616; doi:10.1021/acs.bioconjchem.4c00595)
Supplement: Supplementary file 2 — bc4c00595_si_002.zip [file bc4c00595_si_002.zip › NMR/3j/Primary_NMR_Data_files/1H/pdata/1/email_sk-me-tr-uv-ac_10_1.pdf]

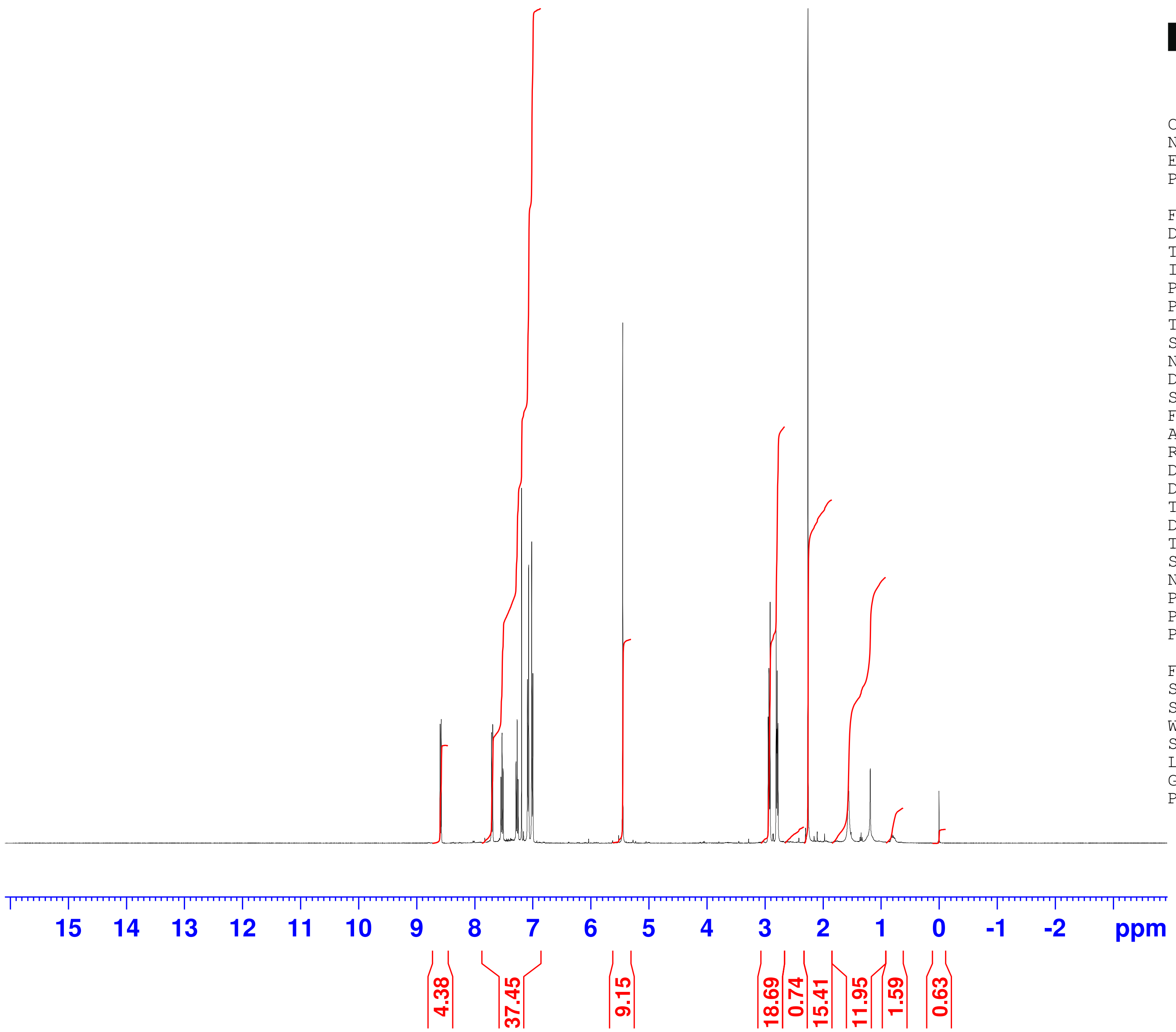

BRUKER

Current Data Parameters
NAME sk-me-tr-uv-ac
EXPNO 10
PROCNO 1

F2 - Acquisition Parameters

Date_	20230517	
Time	22.23	h
INSTRUM	spect	
PROBHD	Z116098_0222 (	
PULPROG	zg30	
TD	65536	
SOLVENT	CDCl3	
NS	16	
DS	2	
SWH	8012.820	Hz
FIDRES	0.244532	Hz
AQ	4.0894465	sec
RG	111.22	
DW	62.400	usec
DE	6.50	usec
TE	298.0	K
D1	1.00000000	sec
TD0	1	
SFO1	400.1524709	MHz
NUC1	1H	
P0	3.33	usec
P1	10.00	usec
PLW1	13.89000034	W

F2 - Processing parameters

SI		65536	
SF		400.1500377	MHz
WDW		EM	
SSB	0		
LB		0.30	Hz
GB	0		
PC		1.00	
