# Supplementary material for: Development of Transiently Strainable Benzocycloheptenes for Catalyst-Free, Visible-Light-Mediated [3 + 2]-Cycloadditions
Source: Bioconjug Chem. 2025 Feb 4;36(2):302–8. doi: 10.1021/acs.bioconjchem.4c00595 (PMC11843616; doi:10.1021/acs.bioconjchem.4c00595)
Supplement: Supplementary file 2 — bc4c00595_si_002.zip [file bc4c00595_si_002.zip › NMR/7d/Primary_NMR_Data_files/19F/pdata/1/email_sk-2-281-ac_11_1.pdf]

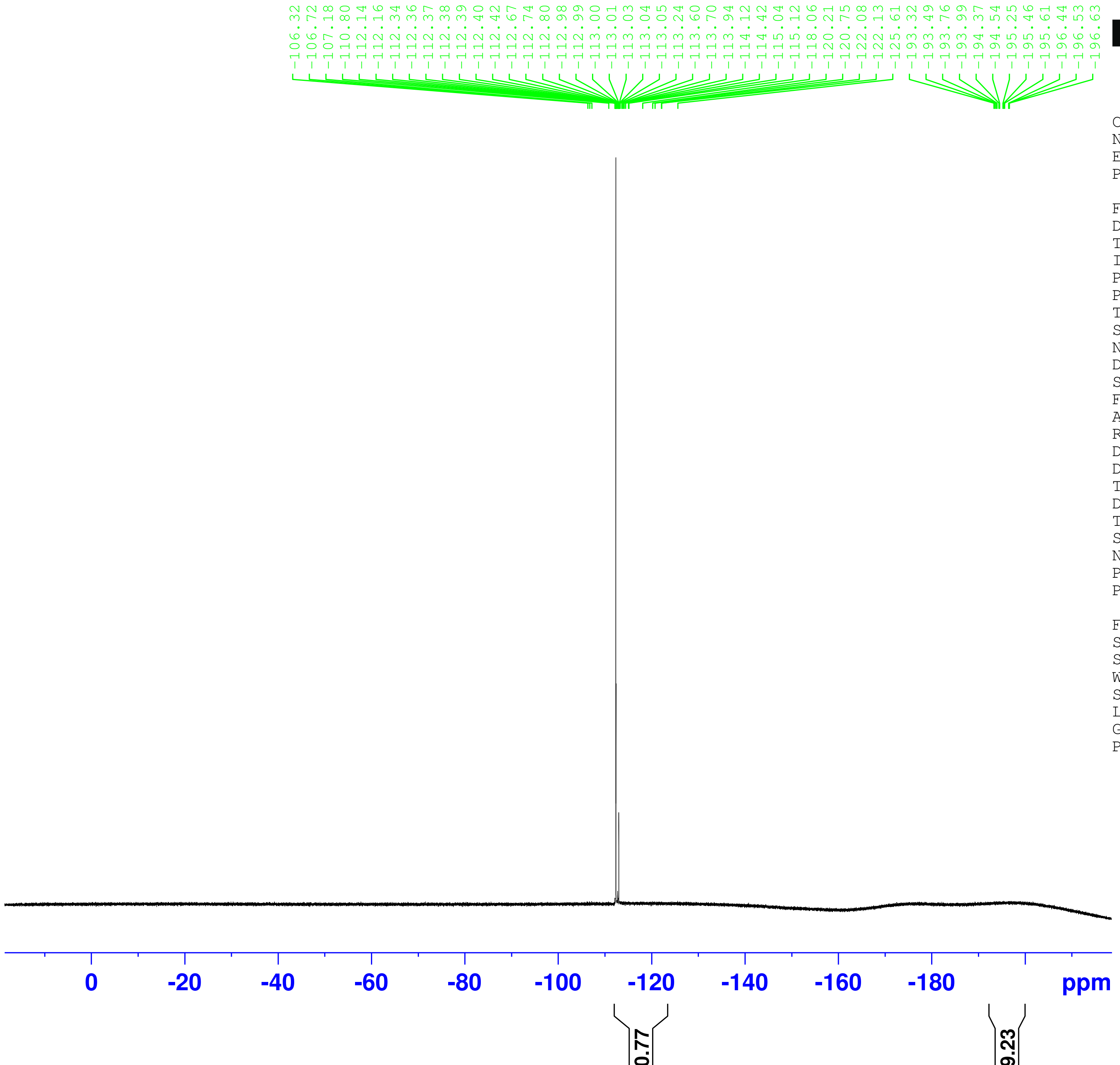

Current Data Parameters

NAME	sk-2-281-ac	
EXPNO	11	
PROCNO	1	

F2 - Acquisition Parameters

Date_	20230709	
Time	18.54	h
INSTRUM	spect	
PROBHD	Z116098_0222 (	
PULPROG	zgflqn	
TD	131072	
SOLVENT	CDCl3	
NS	16	
DS	4	
SWH	89285.711	Hz
FIDRES	1.362392	Hz
AQ	0.7340032	sec
RG	206.31	
DW	5.600	usec
DE	6.50	usec
TE	298.0	K
D1	1.00000000	sec
TD0	1	
SFO1	376.4795333	MHz
NUC1	19F	
P1	14.00	usec
PLW1	22.00000000	W

F2 - Processing parameters

SI	65536	
SF	376.5171850	MHz
WDW	EM	
SSB	0	
LB	0.30	Hz
GB	0	
PC	1.00	
